# Supplementary material for: A Network Analysis of Multiple Myeloma Related Gene Signatures
Source: Cancers (Basel). 2019 Sep 27;11(10):1452. doi: 10.3390/cancers11101452 (PMC6827160; doi:10.3390/cancers11101452)
Supplement: Supplementary file 1 [file cancers-11-01452-s001.pdf]

cancers

MDPI

Supplementary Materials

Network analysis of multiple myeloma related gene signatures

Yu Liu, Haocheng Yu, Seungyeul Yoo, Eunjee Lee, Alessandro Laganà, Samir Parekh, Eric E. Schadt, Li Wang, and Jun Zhu

Supplementary Tables

Supplementary Table 1a. Inconsistencies identified among gene expression, CNV profiles, and metadata (sex, immunoglobulin isotype, and hyperdiploidy).

samples	inconsistency
MMRC0021	sex, not self-aligned between mRNA and CNV
MMRC0039	Heavy chain isotype
MMRC0182	not self-aligned between mRNA and CNV
MMRC0197	sex, not self-aligned between mRNA and CNV
MMRC0198	not self-aligned between mRNA and CNV
MMRC0207	Light chain isotype
MMRC0220	not self-aligned between mRNA and CNV
MMRC0287	not self-aligned between mRNA and CNV
MMRC0312	sex, heavy chain isotype, not self-aligned between mRNA and CNV
MMRC0404	not self-aligned between mRNA and CNV
MMRC0441	not self-aligned between mRNA and CNV
MMRC0442	Hyperdiploidy, not self-aligned between mRNA and CNV

Supplementary Table 1b. Profile pairs pertaining to the same patients predicted by *pro*MODMatcher for mRNA expression and CNV data.

mRNA	CNV
MMRC0198	MMRC0197
MMRC0312	MMRC0404
MMRC0404	MMRC0312
MMRC0441	MMRC0442
MMRC0442	MMRC0441
MMRC0026	MMRC0021

Supplementary Table 2. Informative genes included in the construction of M3CN.

Gene Symbol	expression level	STD
A1BG	6.21469	0.645233
AACS	6.61147	0.564781
AAED1	7.05104	0.874472
AAGAB	6.29963	0.435014
AAR2	6.94667	0.423846
AARS	9.15425	0.618261
AASDH	6.01103	0.59334
AASDHPPT	6.97539	0.532538
AB074162	4.84621	0.714676
AB488780	5.65109	0.485305
ABCA3	6.17598	0.41056
ABCA5	5.80151	0.653924
ABCA7	6.13548	0.461047
ABCB10	6.2787	0.980178
ABCB7	6.62044	0.513687
ABCB9	6.05012	0.636314
ABCC1	6.1035	0.454518
ABCC10	7.00612	0.42682
ABCC5	5.18589	0.417402
ABCD3	6.07773	0.628576
ABCE1	7.90678	0.740026
ABCF1	7.92831	0.548434
ABCG2	6.65254	1.94523
ABHD10	7.35526	0.602539
ABHD12	6.59305	0.563493
ABHD14A	8.74165	0.72424
ABHD14A-ACY1	6.88479	0.551378
ABHD14B	7.85104	0.765494
ABHD15	5.45641	0.483958
ABHD17A	8.50276	0.443415
ABHD17B	5.28926	0.403444
ABHD17C	5.00905	0.8208
ABHD2	6.59107	0.811512
ABHD3	8.57408	0.625446
ABI1	6.7998	0.448589
ABLIM1	5.53644	1.05249
ABO	4.97759	0.457711
abParts	8.10755	2.18565
ABRACL	7.29928	1.19507
ABT1	6.91095	0.489185
AC004692.5	7.70727	0.585106
AC004941.5	6.49705	0.437025
AC004951.6	6.84272	0.694083

AC005523.2	5.22986	0.427741
AC006129.2	7.06059	1.185
AC007389.3	5.11648	0.407099
AC017002.2	6.56456	1.62597
AC018755.16	6.52041	0.491044
AC079305.10	6.66195	0.766249
AC128677.4	9.55253	1.98032
AC139100.2	5.39065	0.479016
ACACB	5.63552	0.472386
ACAD11	6.58554	0.471767
ACAD8	6.76451	0.784327
ACAD9	6.52338	0.44014
ACADM	8.59979	0.885793
ACADSB	5.29831	0.431916
ACADVL	9.33834	0.76671
ACAP3	5.66687	0.417952
ACAT2	6.66058	0.563566
ACBD3	8.66375	0.74114
ACBD5	6.0723	0.478785
ACBD6	7.38651	0.585484
ACCS	5.92939	0.581374
ACD	6.84607	0.458666
ACIN1	6.53644	0.50618
ACLY	7.64742	0.476034
ACN9	5.62084	0.906419
ACOT1	5.33142	0.586964
ACOT13	7.66427	0.725965
ACOT2	5.33142	0.586964
ACOT7	5.77529	0.449468
ACOT9	7.06488	0.485178
ACP2	7.68836	0.4872
ACP5	6.0214	1.11657
ACP6	5.68017	0.448787
ACRBP	4.97301	0.437166
ACRC	5.4339	0.5716
ACSF2	5.55085	0.428895
ACSL1	4.86792	1.19108
ACSL3	6.29393	0.60859
ACSL5	6.53092	0.513121
ACSM3	5.28669	0.782793
ACSS2	6.55799	0.472863
ACTB	12.1771	0.435442
ACTBP9	6.5358	0.443895
ACTG1	12.1635	0.537514
ACTG1P4	6.99143	0.708042
ACTL6A	7.43095	0.858214
ACTL8	5.81708	0.579202

ACTN1	4.87698	0.555225
ACTN4	7.35827	0.484709
ACTR10	8.58845	0.568121
ACTR2	7.22548	0.49788
ACTR3	8.45508	0.479007
ACTR3B	4.89437	0.480392
ACTR3C	5.01371	0.521285
ACTR5	6.2865	0.446308
ACTR6	5.57228	0.437642
ACVR1	7.10181	0.761939
ACVR1C	5.32689	1.03219
ACVR2A	5.51174	0.559663
ACY3	5.10701	0.544884
ACYP1	6.12255	0.728386
ACYP2	7.69582	0.848563
ADA	7.60427	0.955726
ADAM10	8.51815	0.767623
ADAM17	6.39348	0.453026
ADAM19	6.20078	0.49817
ADAM28	5.15794	1.07361
ADAM8	5.2593	0.406303
ADAM9	5.284	0.774956
ADAMTS14	5.5447	0.430223
ADAMTS15	4.94044	0.774629
ADAP2	5.70276	0.533955
ADAR	10.5869	0.572605
ADAT1	5.8357	0.563166
ADAT2	5.03424	0.523023
ADCK3	6.51744	0.443854
ADCY10P1	4.89948	0.467766
ADCY3	5.49733	0.438734
ADCY7	5.74165	0.458371
ADD3	8.77508	0.832795
ADH5	7.51931	0.522788
ADHFE1	6.60265	0.604957
ADIPOR1	9.10963	0.585401
ADIPOR2	7.24073	0.543495
ADIRF-AS1	5.31593	0.460596
ADK	5.81295	0.469949
ADM	9.6342	2.06007
ADNP	7.47229	0.518004
ADNP2	7.37325	0.585409
ADO	6.647	0.651958
ADORA2A	6.77997	0.634214
ADPGK	8.28243	0.496198
ADPRHL2	6.61158	0.49048
ADPRM	5.99994	0.506364

ADRA2C	5.51868	0.410731
ADRB2	6.18278	1.16656
ADRM1	7.97247	0.457206
ADSL	8.53999	0.647667
ADSS	5.13063	0.412394
ADSSL1	5.20372	0.438553
ADTRP	5.84309	1.45365
AEBP1	6.39757	0.587432
AEN	7.46616	0.450918
AES	8.19014	0.647554
AF007147	5.58092	0.620948
AF090939	5.81706	0.602326
AFAP1-AS1	5.08911	1.17308
AFF4	5.2236	0.4104
AFMID	5.34851	0.444393
AFTPH	8.19991	0.411735
AGA	8.8297	0.982531
AGAP1	4.80325	0.512319
AGFG1	5.523	0.408673
AGK	5.55843	0.484633
AGL	6.78223	0.858773
AGO2	5.28637	0.474342
AGO3	5.09244	0.485137
AGPAT1	7.29732	0.459153
AGPAT2	6.34612	0.401432
AGPAT5	5.82265	0.604327
AGPS	5.6101	0.671753
AGTPBP1	6.19783	0.453931
AHCTF1	5.40913	0.519342
AHCY	8.05794	0.852394
AHCYL1	6.37777	0.493916
AHI1	5.15126	0.446925
AHNAK	6.09742	0.743202
AHSA1	7.87063	0.633367
AHSA2	6.74601	0.464932
AHSP	5.44641	0.831006
AIDA	8.84338	0.696347
AIF1	5.70125	0.562981
AIFM1	7.20868	0.66465
AIG1	6.14741	0.46362
AIM2	8.86627	1.11861
AIMP1	7.30292	0.546699
AIMP2	7.45078	0.509681
AIP	7.65696	0.501349
AJ420595	5.40463	1.00493
AJAP1	5.24809	1.03963
AK022030	7.05124	0.724158

AK025288	6.09571	1.27022
AK026714	10.5975	1.26265
AK055981	5.97992	0.632038
AK057657	8.4207	0.585483
AK057978	5.96589	0.729114
AK090844	6.31803	0.597152
AK091028	4.95617	0.41532
AK096592	6.20061	0.598922
AK1	6.07842	0.415877
AK2	6.15022	0.429514
AK3	9.10327	0.586316
AK6	9.19068	0.546909
AKAP1	6.54482	0.557978
AKAP10	5.89243	0.470902
AKAP11	6.56788	0.681903
AKAP13	6.37867	0.458701
AKAP17A	6.65039	0.407718
AKAP2	8.78727	0.592077
AKAP7	5.89567	0.411719
AKAP9	6.03477	0.421876
AKIP1	6.62279	0.462198
AKIRIN1	7.12967	0.473345
AKIRIN2	6.65313	0.698994
AKNA	5.26104	0.485002
AKR1A1	9.39156	0.525935
AKR1B1	8.37229	0.979682
AKR7A2	7.34682	0.539264
AKT1	6.62199	0.539745
AKT1S1	5.49021	0.450108
AKTIP	6.83824	0.469216
AL022341.3	5.67603	0.474572
AL110181	5.46963	0.443827
AL832909	6.35139	0.734422
AL833181	5.20132	0.97366
AL928742.12	5.2176	0.417492
AL928768.3	7.80379	2.97053
ALAS1	6.99842	0.703665
ALCAM	6.84616	1.26862
ALDH16A1	6.99991	0.455843
ALDH18A1	8.54842	0.432165
ALDH1L2	6.13404	0.874968
ALDH2	7.09891	1.20799
ALDH3A2	7.50248	0.461442
ALDH5A1	5.56479	0.766158
ALDH6A1	6.19498	0.637871
ALDH9A1	9.35676	0.537841
ALDOA	9.57557	0.476895

ALDOC	6.66075	0.536795
ALG11	7.06049	0.662687
ALG13	6.82463	0.503028
ALG14	6.48792	0.508182
ALG2	7.43818	0.464924
ALG3	8.79263	0.542539
ALG5	6.16735	0.429734
ALG6	7.49857	0.637907
ALG8	9.25449	0.563772
ALG9	7.32992	0.486908
ALKBH2	6.40432	0.656888
ALKBH3	7.10349	0.577085
ALKBH5	7.33808	0.403266
ALKBH6	6.45975	0.556851
ALKBH8	4.96112	0.55148
ALMS1	6.48434	0.502521
ALOX5	6.44399	0.817457
ALOX5AP	7.70994	1.76865
ALYREF	7.96569	0.743032
AMD1	8.11388	0.60222
AMFR	5.71892	0.656205
AMICA1	5.3775	0.536264
AMIGO2	5.62525	1.56984
AMMECR1	5.41822	0.709339
AMMECR1L	6.80642	0.611023
AMN1	4.97015	0.55296
AMPD1	9.64709	1.54058
AMPD2	6.8356	0.515766
AMPD3	5.21745	0.60759
AMT	7.02061	0.541619
AMY1A	6.46368	0.896307
AMY1B	6.46368	0.896307
AMY1C	6.46368	0.896307
AMY2A	6.46368	0.896307
AMY2B	6.99143	0.708042
AMZ2	9.20754	0.404186
AMZ2P1	6.56584	0.497483
ANAPC10	5.63526	0.546252
ANAPC15	7.6403	0.40305
ANAPC2	6.90262	0.478128
ANAPC4	7.51114	0.467751
ANAPC5	8.0801	0.431267
ANAPC7	6.58153	0.507264
ANG	6.15292	1.21484
ANGEL2	5.73517	0.52518
ANKDD1A	6.82357	0.720324
ANKEF1	4.89678	0.488184

ANKH	4.90764	0.411884
ANKHD1-EIF4EBP3	7.82382	0.409598
ANKIB1	6.61877	0.434108
ANKMY2	7.48385	0.639528
ANKRA2	7.52516	0.506981
ANKRD10-IT1	7.39881	0.702631
ANKRD12	6.86911	0.649301
ANKRD13A	6.74569	0.559906
ANKRD13C	4.88777	0.482774
ANKRD16	6.75599	0.566863
ANKRD17	7.9121	0.551514
ANKRD20A1	5.2635	0.562934
ANKRD20A11P	8.76459	1.22445
ANKRD20A2	5.2635	0.562934
ANKRD20A3	5.2635	0.562934
ANKRD20A4	5.2635	0.562934
ANKRD20A5P	6.40151	0.951935
ANKRD20A9P	9.30384	1.34837
ANKRD27	7.13754	0.696251
ANKRD28	8.57062	0.761848
ANKRD33B	5.15224	0.776456
ANKRD36	5.11709	0.597865
ANKRD36B	9.22737	0.89438
ANKRD36BP2	7.39004	0.821715
ANKRD37	6.37518	0.609335
ANKRD44	5.19556	0.529591
ANKRD46	6.921	0.669486
ANKRD49	8.23266	0.696264
ANKRD6	5.18167	0.761349
ANKS1A	6.24824	0.536038
ANO10	6.32967	0.538522
ANO5	5.25767	0.837159
ANO9	5.91595	0.509519
ANP32A	8.03434	0.628266
ANP32B	11.2604	0.461767
ANP32E	8.44074	0.903811
ANTXR2	5.07924	0.692249
ANXA1	5.22026	1.77321
ANXA11	6.98358	0.496311
ANXA2	8.95301	0.465007
ANXA2P2	8.95991	0.52961
ANXA2R	7.01496	0.713163
ANXA4	6.2636	0.929043
ANXA5	9.92163	0.500996
ANXA6	6.76443	0.527613
ANXA7	9.40383	0.465859
AOAH	5.32827	0.565316

AP000525.9	6.34579	1.30938
AP001462.6	5.05024	0.471056
AP1G1	6.44149	0.550843
AP1M1	6.86859	0.458115
AP1S1	5.64573	0.41392
AP1S2	7.49139	1.04756
AP2B1	7.0053	0.550498
AP2M1	8.8074	0.436803
AP2S1	8.1521	0.415239
AP3M1	6.79424	0.514651
AP3S1	10.2634	0.579221
AP5B1	5.30371	0.439871
AP5M1	6.05875	0.466172
APBA3	6.26625	0.406505
APBB1IP	5.50797	0.677945
APBB3	6.47076	0.528358
APC	5.22195	0.403897
APEX1	10.1273	0.614892
APH1A	6.55342	0.436955
APH1B	6.75233	0.620882
API5	6.52784	0.488727
APITD1	5.94298	0.825303
APITD1-CORT	5.94298	0.825303
APLP2	6.70306	0.699882
APMAP	7.59185	0.510358
APOA1BP	9.44745	0.603323
APOBEC2	5.22801	0.464361
APOBEC3B	6.18036	1.49924
APOBEC3C	7.67422	0.476787
APOBEC3G	7.29852	0.501756
APOC1	6.24801	0.413761
APOE	6.21351	0.529286
APOL1	7.62601	0.771236
APOL3	6.44941	0.96055
APOL6	6.4442	0.660255
APOM	6.09665	0.459978
APOO	7.29018	0.577527
APOOL	5.86839	0.515704
APP	6.44612	1.55475
APPBP2	5.18028	0.52098
APPL1	6.09492	0.723688
APPL2	5.51064	0.630694
APRT	8.20599	0.565238
AQP3	8.53925	0.709937
AQP5	4.98747	0.448494
AQP9	4.80971	0.754081
AQR	5.95996	0.460875

ARCN1	9.35522	0.566712
ARF3	7.2849	0.494535
ARF4	10.6962	0.543002
ARF5	7.13586	0.405125
ARF6	8.29295	0.449952
ARFGAP3	8.86908	0.59879
ARFGEF1	6.4866	0.578255
ARFIP1	6.56766	0.745391
ARFIP2	8.28757	0.476898
ARGLU1	6.94722	0.504323
ARHGAP12	5.77932	0.681511
ARHGAP15	5.23271	0.633789
ARHGAP17	6.53923	0.664191
ARHGAP18	6.2923	0.799109
ARHGAP21	6.0585	0.522524
ARHGAP30	7.61911	0.445023
ARHGAP4	7.1281	0.603117
ARHGAP5	5.03106	0.515777
ARHGAP5-AS1	4.88892	0.460924
ARHGAP9	6.55759	0.78665
ARHGDIA	6.8489	0.529835
ARHGDIB	8.53232	0.681317
ARHGDIG	4.91273	0.444131
ARHGEF18	7.35574	0.5853
ARHGEF2	7.28968	0.473586
ARHGEF3	7.57587	1.07809
ARHGEF6	7.37405	1.03211
ARHGEF9	5.73714	0.508758
ARID2	6.43191	0.562704
ARID3A	6.66218	0.435378
ARID3B	7.09759	0.470822
ARID4A	5.57869	0.654358
ARID5A	6.51997	0.730237
ARID5B	5.41279	0.613535
ARIH1	6.77518	0.503579
ARIH2OS	4.91636	0.636801
ARL1	8.33533	0.611738
ARL14EP	7.22688	0.789869
ARL2	7.04965	0.462245
ARL2BP	7.69318	0.529481
ARL4A	7.51213	0.947673
ARL4C	5.47021	1.0304
ARL5A	7.64565	0.479051
ARL5B	5.7342	0.481439
ARL6IP1	8.24031	0.858559
ARL6IP5	9.72772	0.881155
ARL6IP6	5.81704	0.443979

ARL8A	6.37204	0.413591
ARL8B	7.88123	0.565675
ARMC1	8.46809	0.644011
ARMC10	7.79375	0.495441
ARMC2	6.45436	1.42674
ARMC2-AS1	10.5085	0.516945
ARMC5	6.56581	0.447146
ARMC8	5.70026	0.441779
ARMCX2	7.00315	1.244
ARMCX3	8.49011	0.668835
ARMCX5	5.98538	0.650835
ARMCX5-GPRASP2	4.8676	0.721292
ARNTL	5.14179	0.442487
ARPC1A	9.19478	0.614568
ARPC1B	8.64497	0.699697
ARPC2	9.90518	0.46461
ARPC3	9.74486	0.488096
ARPC5	7.36608	0.560273
ARPC5L	9.15025	0.733286
ARPP19	8.0637	0.65851
ARRB2	5.63339	0.48896
ARRDC3	8.53395	0.889334
ARRDC4	4.88175	1.03913
ARSG	5.80608	0.552719
ARTN	5.31939	0.549176
ARX	5.10314	0.669625
ASAH1	7.77987	0.458757
ASAP1	6.42638	0.58565
ASAP1-IT1	4.93888	0.581374
ASB16-AS1	5.15907	0.422984
ASB7	5.61937	0.552805
ASB8	6.45397	0.476286
ASCC1	6.89727	0.632972
ASCC2	6.25033	0.486869
ASCC3	6.95427	0.622113
ASF1A	6.68214	0.968232
ASF1B	5.87408	0.69073
ASH1L	7.29129	0.478417
ASH1L-AS1	6.7915	0.844079
ASH2L	7.49996	0.524876
ASL	6.77731	0.466755
ASMTL	6.99081	0.517673
ASNA1	7.75419	0.477042
ASNS	6.33405	0.499034
ASNSD1	8.29303	0.561232
ASPHD2	6.72472	0.798304
ASPSCR1	5.75312	0.428414

ASRGL1	5.76838	0.492945
ASS1	7.81284	2.18649
ASTE1	5.81705	0.537652
ASUN	8.61656	0.846503
ASXL1	6.85299	0.458481
ASXL2	5.73071	0.436315
ATAD1	7.09771	0.497149
ATF1	6.51027	0.634345
ATF2	5.56272	0.46499
ATF3	7.91046	1.13175
ATF4	11.6117	0.483245
ATF5	6.55911	0.732032
ATF6	7.87062	0.698729
ATF7IP2	5.84429	0.838921
ATG12	7.19136	0.462609
ATG14	6.34055	0.606595
ATG16L2	6.20141	0.597778
ATG2A	7.35431	0.410917
ATG2B	5.62945	0.44903
ATG3	6.79684	0.431816
ATG4A	7.79188	0.534873
ATG4C	6.55684	0.866879
ATG5	7.04545	0.541697
ATHL1	5.96	0.415749
ATIC	8.95051	0.585516
ATL1	5.40077	0.701119
ATL3	5.96573	0.446213
ATM	6.38158	0.407865
ATOX1	8.71226	0.598396
ATP10B	6.50477	1.67158
ATP10D	7.1957	1.13096
ATP11B	6.67282	0.603329
ATP11C	5.16749	0.782752
ATP13A1	9.00079	0.638484
ATP13A2	5.94822	0.578919
ATP13A3	6.14553	0.738256
ATP1A1	7.91662	0.54585
ATP1B1	6.78236	1.64613
ATP2A3	7.11657	0.592689
ATP2B1	6.18116	0.842299
ATP2B4	6.14453	0.685246
ATP2C1	5.81508	0.566854
ATP5B	10.4662	0.419596
ATP5D	8.55334	0.569127
ATP5G1	8.86966	0.513073
ATP5G3	9.05166	0.404437
ATP5I	10.0953	0.439247

ATP5J	10.8335	0.530657
ATP5L	11.3796	0.498815
ATP6	10.3977	0.409679
ATP6AP1	9.16664	0.710979
ATP6AP2	7.20404	0.428973
ATP6V0A1	6.71572	0.453242
ATP6V0B	9.18394	0.506962
ATP6V0C	9.0741	0.42797
ATP6V0D1	8.45215	0.506371
ATP6V0E1	7.93463	0.452541
ATP6V0E2	5.20495	0.690355
ATP6V1A	8.4931	0.675818
ATP6V1B2	6.98595	0.660149
ATP6V1C1	6.10082	0.616116
ATP6V1E1	8.55152	0.533766
ATP6V1E2	5.0861	0.44834
ATP6V1F	9.38528	0.449613
ATP6V1G1	8.69732	0.40574
ATP6V1G2-DDX39B	7.57275	0.550332
ATP7A	5.62575	0.527991
ATP8A2	4.85277	0.9021
ATP8B2	8.59937	0.588868
ATPAF1	7.08395	0.49234
ATPIF1	7.74908	0.452443
ATRAID	10.8553	0.521856
ATRN	6.45878	0.449088
ATRX	7.19125	0.749414
ATXN1	6.51422	0.591383
ATXN10	7.75545	0.436573
ATXN1L	7.43666	0.543988
ATXN2	6.60064	0.442451
ATXN2L	6.00373	0.414906
ATXN7L3B	7.27131	0.455185
AUH	8.38866	0.567669
AUP1	9.05857	0.412394
AURKAPS1	6.94	0.498016
AURKB	4.82296	0.581676
AVEN	8.05872	0.664079
AVPI1	6.55548	0.544256
AX746823	5.49637	0.932123
AX747182	6.84272	0.694083
AX747730	5.073	0.630952
AX747826	7.0886	1.03099
AX748157	5.2835	0.405749
AY940074	6.31599	0.456381
AZGP1	5.70804	1.88235
AZGP1P1	5.07074	0.527679

AZIN1	9.09398	0.640295
AZU1	4.89179	0.968608
B2M	11.7438	0.436909
B3GNT2	5.35168	0.472995
B3GNT8	4.89951	0.412622
B3GNT9	6.39215	0.535887
B3GNTL1	5.33611	0.415638
B4GALT1	5.31873	0.482391
B4GALT4	7.28688	0.425495
B4GALT5	6.5665	0.641947
B9D1	6.11163	0.52552
BABAM1	8.05118	0.431469
BACH2	5.17244	0.706941
BAG1	7.40568	0.470117
BAG3	6.61714	1.01181
BAG4	5.63895	0.567225
BAG5	5.96718	0.598635
BAGE2	5.04182	0.853082
BAGE3	4.81932	0.758077
BAGE4	5.26432	0.987105
BAGE5	4.81932	0.758077
BAI1	5.97721	0.430359
BAK1	6.47926	0.506634
BANF1	9.17699	0.746803
BANK1	6.40021	1.38553
BARD1	5.81051	0.591682
BARX2	4.87704	0.427105
BASP1	5.95325	1.14335
BATF	6.46426	0.459426
BATF3	5.64704	0.609114
BAX	6.11464	0.549966
BAZ1A	6.48275	0.819989
BAZ2B	7.70835	0.786646
BBC3	6.83255	0.438232
BBS2	5.42188	0.51745
BBS4	5.4059	0.525568
BBX	8.05477	0.613641
BC005081	5.52274	0.60478
BC017398	6.10777	2.2316
BC019672	5.46676	0.660106
BC022047	5.66359	0.723578
BC022568	5.65388	0.67572
BC030152	6.01016	0.436102
BC034319	5.13685	1.1988
BC036830	4.90834	0.919506
BC039681	5.99317	0.48171
BC041025	6.07232	0.871509

BC044596	5.71003	0.491431
BC047484	7.79432	0.475653
BC048103	5.31679	0.777497
BC062753	6.66154	0.622908
BC069804	4.82947	1.45895
BC070363	7.59733	0.740596
BCAP29	5.22403	0.434983
BCAP31	9.02637	0.693455
BCAR3	5.06532	0.853407
BCAS1	5.39423	0.489884
BCAS2	8.99879	0.485124
BCAS3	5.4713	0.442333
BCCIP	6.6991	0.482264
BCDIN3D	5.55729	0.554496
BCKDHB	6.47624	0.513157
BCKDK	7.10357	0.456412
BCL10	5.63162	0.628298
BCL11A	6.76815	1.29662
BCL2	6.59717	0.465981
BCL2A1	5.07236	1.20452
BCL2L1	6.35936	0.577636
BCL2L11	7.88012	0.798436
BCL2L2-PABPN1	8.05298	0.4262
BCL3	5.89018	0.472921
BCL6	4.93896	0.814012
BCL7B	6.89129	0.400931
BCL7C	6.65141	0.402071
BCLAF1	7.58988	0.602149
BCOR	5.27216	0.573995
BCR	6.2114	0.451361
BCS1L	7.19284	0.54583
BD495725	5.67733	0.729949
BDH2	5.14155	0.495487
BDP1	5.58252	0.462655
BEND3	6.26293	0.421092
BET1	8.41049	0.91145
BET1L	7.12512	0.407676
BEX2	8.02148	1.31124
BEX4	6.52785	1.7741
BEX5	8.08182	2.04933
BFAR	7.56789	0.530948
BFSP2	6.18534	0.894987
BGLAP	5.2343	0.958823
BHLHE40	5.22032	1.0804
BHLHE41	6.48984	0.845788
BICD1	6.20274	0.595607
BICD2	6.22381	0.44182

BID	6.78491	0.505135
BIK	7.49811	1.05571
BIN1	5.98067	0.527438
BIN2	8.41036	0.596097
BIRC2	8.7095	0.953465
BIRC3	6.92313	1.8033
BIRC5	4.87071	0.590653
BIRC6	7.48687	0.565481
BIRC7	6.23206	0.461013
BLCAP	7.33481	0.585521
BLK	4.82674	0.533618
BLM	6.59574	0.823088
BLMH	6.87841	0.532562
BLNK	8.79295	1.18466
BLOC1S1	8.00641	0.448128
BLOC1S2	7.99543	0.625105
BLOC1S4	6.73324	0.514262
BLOC1S5	6.6558	0.563247
BLOC1S6	8.58432	0.524051
BLVRA	5.27439	0.605106
BLVRB	7.78958	0.708105
BMF	6.89532	0.714294
BMI1	11.4923	0.689983
BMP4	5.82218	0.675302
BMP6	6.12983	0.433112
BMP8B	5.60872	0.671909
BMPR2	5.91416	0.671598
BMS1	7.16076	0.453001
BMS1P20	5.92982	0.502325
BMS1P6	6.76392	0.56058
BNIP2	7.53462	0.598053
BNIP3	5.84035	1.59358
BNIP3L	7.92318	0.862785
BOD1	7.51181	0.522127
BOD1L1	5.92301	0.656177
BOLA1	6.14759	0.652356
BOLA2	6.59554	0.404202
BOLA2B	9.76352	0.542828
BOLA3	9.38393	0.612274
BOP1	6.72807	0.575048
BORA	5.3332	0.669066
BPGM	5.68401	0.778263
BPNT1	4.94167	0.431432
BPTF	5.94382	0.404775
BRAF	5.84907	0.421386
BRAT1	5.88769	0.467619
BRCA1	5.10003	0.517153

BRCC3	5.97652	0.757807
BRD2	8.91775	0.57011
BRE-AS1	4.97065	0.41527
BRI3BP	6.23073	0.918316
BRIX1	6.56342	0.606133
BRMS1	7.45134	0.409055
BRMS1L	5.70356	0.533869
BROX	6.19813	0.545544
BRSK1	5.83412	0.556028
BSCL2	9.32044	0.728761
BSDC1	6.90744	0.411915
BSG	9.22606	0.678712
BSPRY	4.83038	0.677406
BST2	9.1981	0.656293
BTAF1	8.58077	0.687196
BTBD1	8.71004	0.506821
BTBD10	6.74239	0.566765
BTBD2	5.5645	0.431075
BTBD3	7.41989	1.87355
BTBD6	7.30424	0.584332
BTD	6.24596	0.483172
BTG1	9.71703	0.891238
BTG2	10.177	0.862782
BTG3	6.15651	0.448764
BTK	7.80094	0.712274
BTLA	7.78415	1.76253
BTN2A2	5.23536	0.543611
BTN3A1	8.36419	0.587035
BTN3A2	9.22997	0.813081
BTN3A3	8.75909	0.816974
BTNL3	6.08048	0.478392
BUB1B	6.09239	1.43846
BUB3	7.36863	0.442429
BUD13	6.15692	0.61516
BYSL	6.82802	0.680068
BZRAP1	5.09251	0.40736
BZW1	9.13204	0.482201
BZW2	9.30589	0.771555
C10orf10	6.70971	1.19097
C10orf2	5.88393	0.609895
C10orf32	7.60287	0.675059
C10orf35	5.40304	0.414539
C10orf54	6.67825	0.619076
C10orf88	5.15829	0.460229
C11orf1	6.8454	0.538069
C11orf24	8.10744	0.436108
C11orf48	8.39657	0.518503

C11orf54	6.04929	0.521583
C11orf57	5.12796	0.470256
C11orf68	7.33022	0.41696
C11orf71	7.26156	0.771021
C11orf73	8.0893	0.522415
C11orf74	5.16066	1.1623
C11orf80	7.04485	0.858065
C11orf83	6.55482	0.544026
C11orf96	6.70435	2.11985
C12orf10	7.03577	0.660322
C12orf29	6.68798	0.679561
C12orf4	6.09974	0.754452
C12orf43	5.83988	0.420722
C12orf45	8.25259	0.785422
C12orf5	6.04753	0.800992
C12orf57	10.4401	0.66463
C12orf66	5.15102	0.641949
C12orf73	5.75502	0.526337
C12orf75	5.1262	1.60708
C14orf119	8.23979	0.704087
C14orf142	6.75178	0.73036
C14orf166	10.6208	0.475498
C14orf169	6.48087	0.638606
C14orf2	9.39064	0.497224
C14orf28	5.45459	0.693312
C15orf37	4.98535	0.507878
C15orf39	6.30612	0.586704
C15orf57	6.11528	0.598597
C15orf61	9.59181	0.52854
C15orf65	6.80567	0.83032
C16orf52	5.44581	0.430726
C16orf54	7.78501	1.35046
C16orf72	6.45428	0.610823
C16orf74	6.14683	0.609787
C16orf80	7.90901	0.746916
C16orf91	6.92838	0.409112
C16orf93	5.06984	0.41105
C17orf49	9.16864	0.564148
C17orf58	7.02209	0.688076
C17orf62	7.11149	0.592152
C17orf75	5.38063	0.619888
C17orf96	6.1398	0.406279
C19orf10	10.3976	0.624328
C19orf12	6.83149	0.435204
C19orf24	7.36112	0.553138
C19orf43	8.11843	0.403252
C19orf48	7.28158	0.619069

C19orf52	6.66665	0.508617
C19orf53	10.0018	0.488413
C19orf54	7.05834	0.537179
C19orf60	8.13782	0.451055
C19orf66	6.86921	0.455857
C19orf68	5.91146	0.429746
C19orf70	9.37677	0.560299
C19orf81	4.87131	0.413005
C19orf83	6.04301	0.433195
C1D	7.67855	0.571846
C1GALT1	6.27255	0.600925
C1GALT1C1	7.28283	0.544435
C1orf106	6.5128	1.3744
C1orf109	4.84861	0.641891
C1orf122	7.95032	0.457115
C1orf131	6.70038	0.89522
C1orf174	5.87782	0.555995
C1orf186	5.56342	0.616479
C1orf198	5.57547	0.578209
C1orf21	4.87578	0.574239
C1orf216	5.81874	0.496919
C1orf27	6.604	0.448365
C1orf43	9.16674	0.619446
C1orf50	6.14745	0.429756
C1orf52	5.55318	0.442532
C1orf53	5.23385	0.66919
C1orf54	5.10763	0.604039
C1orf85	6.4533	0.700329
C1QA	5.1596	1.05878
C1QBP	9.21919	0.83275
C1QC	5.13515	1.10747
C1QTNF3-AMACR	5.35306	0.726348
C1R	6.30174	1.06787
C2	5.27843	0.767103
C20orf141	5.35522	0.402337
C20orf203	4.81463	0.431706
C20orf24	10.386	0.410734
C21orf119	5.38643	0.548854
C21orf15	5.72539	0.40164
C21orf33	7.8418	0.560829
C21orf91	8.28232	0.901514
C22orf39	5.8545	0.47741
C2CD2	6.42942	0.793924
C2CD5	8.43538	0.602663
C2orf42	6.27064	0.575757
C2orf43	6.38927	0.491338
C2orf47	7.23386	0.566237

C2orf49	5.29403	0.409301
C2orf57	5.14842	0.40769
C2orf68	5.05836	0.459567
C2orf69	8.16215	0.54569
C2orf74	6.80503	0.577647
C2orf76	6.61523	1.09445
C2orf88	7.58047	0.922098
C3	5.56847	0.406665
C3orf17	6.58704	0.426578
C3orf18	6.17173	0.402698
C3orf38	5.64779	0.504528
C3orf70	5.2862	1.81181
C4A	7.12099	1.31772
C4B	7.12099	1.31772
C4B_2	7.12099	1.31772
C4orf29	6.07328	0.405673
C4orf3	8.63899	0.609395
C4orf32	5.2247	0.585983
C4orf46	7.26459	0.570647
C4orf48	7.44645	0.816573
C5orf22	5.74005	0.444814
C5orf24	5.85277	0.450582
C5orf28	6.33478	0.69799
C5orf30	7.05332	0.845355
C5orf56	6.7987	0.475512
C6orf1	6.16184	0.418363
C6orf106	6.21069	0.415089
C6orf120	7.53169	0.770049
C6orf136	6.32005	0.565033
C6orf203	6.52983	0.677279
C6orf211	6.27178	1.07513
C6orf226	7.07501	0.577537
C6orf48	10.2412	0.848227
C6orf62	7.76123	0.640432
C7orf31	6.32778	0.690435
C7orf55	7.68248	0.68035
C7orf55-LUC7L2	7.5465	0.521967
C7orf60	5.35463	0.597866
C8orf33	6.44748	0.555747
C8orf44-SGK3	6.11444	0.671584
C8orf59	8.45158	0.542255
C8orf60	6.19028	0.664041
C8orf76	5.6282	0.685037
C8orf82	6.32491	0.621046
C9orf116	5.10189	0.433799
C9orf131	5.17268	0.451846
C9orf142	6.72587	0.516395

C9orf172	5.7219	0.414269
C9orf40	5.37955	0.471767
C9orf41	5.91112	0.66663
C9orf64	7.38407	0.537594
C9orf69	6.43648	0.476412
C9orf72	5.36308	0.623607
C9orf78	6.77309	0.421048
C9orf91	6.95721	0.75516
CA1	4.91149	0.570687
CA11	5.49905	0.568856
CA5BP1	5.46407	0.403327
CAAP1	6.75158	0.618158
CAB39	7.37737	0.561762
CABLES1	5.44327	0.579942
CABLES2	5.92867	0.526246
CACYBP	7.56963	0.574226
CADM1	6.4789	1.47723
CADPS2	8.54253	1.14018
CALCOCO1	7.62013	0.418301
CALM2	8.35078	0.410513
CALM3	8.35078	0.410513
CALML4	5.33903	0.695043
CALR	8.59552	0.632189
CALU	8.27446	0.526964
CAMK1D	6.57205	0.63555
CAMK2D	6.46437	0.874169
CAMLG	9.04329	0.640812
CAMP	6.84186	1.12205
CAMSAP2	5.80331	0.762118
CAND1	5.83303	0.418745
CAP1	9.49753	0.653105
CAPG	6.26559	0.784996
CAPN12	6.27585	0.553358
CAPN2	8.27521	0.639198
CAPN3	5.79292	0.61011
CAPN5	4.9032	0.407554
CAPN7	7.63955	0.51369
CAPNS1	9.16074	0.512072
CAPRIN1	6.90119	0.482904
CAPZA2	7.08108	0.479093
CARD16	5.78424	0.6597
CARKD	6.4177	0.703803
CARM1	6.53336	0.582457
CARNS1	5.08528	0.41032
CASC14	5.36703	0.423693
CASC3	7.22201	0.452167
CASP1	7.82416	1.13104

CASP10	6.46791	0.414419
CASP3	8.39408	0.6958
CASP4	6.63756	0.759815
CASP6	6.0104	0.527728
CASP7	6.82038	0.60955
CASP8	6.0676	0.41041
CAST	7.01707	0.439866
CAT	6.75076	0.574267
CATIP-AS1	5.03141	0.490483
CAV1	8.61711	1.22221
CAV2	5.5656	0.818981
CBFA2T2	5.85283	0.409157
CBFA2T3	6.37514	0.654708
CBFB	7.40018	0.564214
CBL	5.86664	0.484113
CBLL1	5.58911	0.592708
CBLN3	4.83816	0.498432
CBR1	6.52051	0.817885
CBR4	6.46066	0.548043
CBS	6.45116	0.860521
CBWD1	8.92557	0.638545
CBWD2	8.92557	0.638545
CBWD3	8.92557	0.638545
CBWD5	8.92557	0.638545
CBWD6	8.92557	0.638545
CBWD7	8.92557	0.638545
CBX1	7.1314	0.758294
CBX3	7.64279	0.574007
CBX4	8.20871	0.588237
CBX5	5.72329	0.520737
CBX6	7.4715	0.476882
CBX7	8.08286	0.88206
CCAR1	7.21056	0.555382
CCBL2	7.33453	0.73808
CCDC102A	5.31372	0.448245
CCDC104	6.03999	0.836956
CCDC105	5.27349	0.408636
CCDC107	7.05977	0.424903
CCDC109B	7.35421	0.739305
CCDC110	5.39403	1.00525
CCDC115	6.70434	0.632765
CCDC117	6.96495	0.693364
CCDC12	7.68488	0.431426
CCDC125	5.0762	0.521529
CCDC126	6.48283	0.607334
CCDC134	5.85894	0.438109
CCDC137	5.74911	0.465187

CCDC144B	6.32497	1.74973
CCDC144CP	4.83189	0.892837
CCDC151	5.57917	0.671696
CCDC167	7.87092	0.676893
CCDC186	6.12527	0.736562
CCDC23	6.15458	0.480993
CCDC25	6.90564	0.405329
CCDC28A	7.77064	0.69818
CCDC43	6.43163	0.644431
CCDC47	8.49551	0.490517
CCDC50	6.68267	0.780181
CCDC51	6.71408	0.613684
CCDC53	8.50862	0.622352
CCDC58	6.33773	0.969132
CCDC59	8.75942	0.488135
CCDC6	7.21708	0.982327
CCDC69	7.40668	0.81976
CCDC71L	5.76982	0.439473
CCDC84	6.98197	0.622134
CCDC85A	5.29632	0.933476
CCDC86	6.60022	0.79448
CCDC88A	5.47879	0.81118
CCDC88C	6.85792	0.647267
CCDC91	6.89001	0.6339
CCDC92	5.66738	0.529633
CCL21	5.29217	0.422918
CCL3	8.41626	2.59876
CCL3L1	8.41626	2.59876
CCL3L3	8.41626	2.59876
CCL4	6.93672	1.80043
CCL5	5.66536	0.977366
CCM2	8.06775	0.513328
CCNB1IP1	8.4053	0.860063
CCNB2	4.86542	0.467309
CCNC	7.47802	0.534066
CCND1	6.61684	2.50469
CCND2	5.66767	2.07575
CCND3	6.67651	0.686886
CCNDBP1	9.26909	0.522172
CCNE1	5.52288	0.406395
CCNE2	5.44409	1.22066
CCNG1	9.89381	0.858847
CCNG2	5.833	0.594296
CCNH	8.43477	0.508828
CCNI	8.80287	0.426936
CCNL1	9.5874	0.705219
CCNT1	7.23925	0.491902

CCNT2	5.45908	0.414239
CCNYL1	5.67847	0.627888
CCP110	5.02095	0.44094
CCPG1	9.00109	0.720334
CCR1	6.14127	1.64836
CCR10	6.9045	1.72354
CCR2	8.51237	1.82907
CCR5	5.68849	1.23535
CCRL2	5.62823	1.04357
CCSAP	5.11565	0.838584
CCT2	9.59579	0.763913
CCT3	9.68135	0.786487
CCT5	7.03129	0.558818
CCT6A	7.90833	0.865302
CCT6B	5.43335	0.519755
CCT6P1	6.71886	0.438478
CCT6P3	6.71886	0.438478
CCT8	7.62764	0.504259
CCZ1B	7.69691	0.457204
CD14	5.13231	0.879003
CD151	7.17079	0.418077
CD163L1	5.24715	0.904242
CD164	9.85951	0.562006
CD177	5.2403	0.592308
CD180	8.6055	1.29402
CD1D	7.12715	1.83292
CD200	7.7065	1.97204
CD27	7.27754	1.85015
CD274	5.42607	0.671515
CD300A	5.63991	0.801868
CD302	6.05847	1.06541
CD320	7.61738	0.675712
CD33	5.05221	1.00247
CD37	5.80365	0.629754
CD38	10.7495	0.748618
CD3E	5.25208	0.523954
CD3EAP	6.07016	0.655278
CD40	6.78741	0.611743
CD44	5.98365	1.39886
CD46	9.75704	0.820185
CD48	8.8771	0.94041
CD52	5.71837	1.56338
CD53	7.43724	0.441173
CD55	8.39824	0.845423
CD59	8.49628	0.684472
CD5L	5.85292	0.699251
CD63	11.4853	0.715181

CD69	5.05477	2.27824
CD74	7.53803	0.871911
CD79A	7.67381	1.55873
CD81	6.00178	1.58568
CD83	6.44298	0.773062
CD86	5.52451	0.963574
CD9	5.26753	0.894601
CD97	7.52977	0.907034
CD99	6.13522	1.55387
CDC123	7.61794	0.600175
CDC16	8.12107	0.658578
CDC20	5.21591	0.906031
CDC23	5.46113	0.740436
CDC25A	4.86089	0.481663
CDC25B	6.99881	0.713874
CDC26	8.57766	0.521025
CDC27	6.77417	0.491008
CDC37L1	5.14239	0.522402
CDC40	5.82747	0.590358
CDC42	7.46656	0.526306
CDC42-IT1	6.11661	0.975217
CDC42EP3	6.26926	0.683061
CDC42SE1	7.15371	0.578801
CDC42SE2	8.85096	0.749815
CDC45	5.31013	0.639958
CDC73	6.70914	0.682744
CDCA3	5.39212	0.636488
CDCA4	6.0218	0.517507
CDCA5	6.2691	0.819606
CDCA7L	7.17869	0.899623
CDCA8	5.93188	0.627461
CDH2	5.07994	1.0142
CDIP1	6.11527	0.446829
CDIPT	9.08854	0.477403
CDK1	5.1265	1.0655
CDK14	6.61506	0.477116
CDK17	7.17442	0.589074
CDK18	5.65892	0.516905
CDK2	5.01438	0.401588
CDK2AP1	7.44727	0.959436
CDK2AP2	8.60157	0.723924
CDK4	8.77658	0.476707
CDK5	6.90011	0.54106
CDK5RAP3	9.89061	0.439785
CDK6	5.66682	0.858562
CDK7	7.00213	0.702256
CDK8	5.20224	0.536312

CDKN1A	9.3159	0.911482
CDKN1B	9.68295	0.895101
CDKN1C	5.48965	0.843265
CDKN2A	6.01784	0.420978
CDKN2AIP	7.17507	0.677982
CDKN2C	6.11082	1.1992
CDKN3	6.04942	1.22101
CDR2	7.91905	0.577176
CDRT15	4.94058	0.427158
CDRT4	5.81023	0.723584
CDT1	5.16839	0.494644
CDV3	10.5054	0.564167
CDX1	6.47608	0.446197
CDYL	6.72459	0.612209
CEACAM1	5.58264	0.643
CEACAM21	5.76654	0.57209
CEBPA	4.84067	0.413374
CEBPB	9.60987	0.864884
CEBPE	5.51801	0.419791
CEBPG	6.99383	0.626708
CEBPZ	7.74255	0.500236
CEBPZOS	5.64169	0.420189
CECR1	9.87639	1.2919
CECR5	7.25919	0.596746
CELF2	6.53406	0.736405
CELP	4.87889	0.441511
CELSR3	5.35473	0.651122
CENPA	5.14026	0.711314
CENPB	5.82117	0.4342
CENPC	5.54016	0.649042
CENPE	4.91887	0.948478
CENPH	5.88372	0.946231
CENPJ	4.98296	0.604839
CENPM	5.98756	0.655995
CENPU	5.59931	0.677615
CENPW	6.54596	1.14098
CEP120	6.60802	0.570795
CEP128	5.30093	0.844906
CEP170	6.30818	0.657134
CEP170B	6.67722	0.44741
CEP170P1	6.30818	0.657134
CEP192	6.32835	0.580435
CEP290	5.00973	0.41701
CEP350	8.28597	0.574289
CEP57	7.29814	0.793145
CEP68	5.44088	0.509616
CEP70	5.21423	0.532973

CEP72	4.98119	0.488607
CEP76	5.08656	0.729661
CEP78	5.74537	0.548556
CEP85L	5.60105	0.544365
CEP95	5.65976	0.427391
CEP97	5.10425	0.645472
CEPT1	5.4832	0.517842
CERCAM	6.19849	0.476456
CERK	8.41125	0.69225
CERS2	8.81669	0.54998
CES4A	6.02213	0.415687
CETN2	8.19919	0.863946
CETN3	7.92655	0.974286
CFB	4.90362	0.492095
CFD	5.88552	0.826408
CFL2	7.03878	0.820421
CFLAR	7.82586	0.612219
CGGBP1	8.44759	0.425077
CGREF1	5.06006	0.763349
CGRRF1	6.26892	0.549774
CH17-132F21.1	9.55253	1.98032
CHAC1	6.44713	1.07873
CHAC2	8.13713	1.12761
CHAMP1	6.7274	0.835948
CHCHD1	8.58827	0.550549
CHCHD10	9.42378	0.707215
CHCHD2	11.2005	0.52269
CHCHD3	5.89031	0.40722
CHCHD4	7.49692	0.565044
CHCHD6	6.01119	0.426155
CHCHD7	7.84135	0.569675
CHD1	7.681	0.64277
CHD1L	6.37145	0.603626
CHD2	6.58594	0.51038
CHD7	6.43129	0.530984
CHD8	6.64103	0.488338
CHD9	6.04015	0.597936
CHEK1	5.45718	0.776643
CHEK2	5.90813	0.649625
CHFR	5.87104	0.546299
CHIC2	6.67319	0.698647
CHID1	8.65359	0.535339
CHKA	5.98257	0.410241
CHKB	7.88772	0.428083
CHKB-CPT1B	5.38067	0.636478
CHMP1B	7.04237	0.437619
CHMP2B	7.62137	0.484812

CHMP3	7.75013	0.519004
CHMP4A	7.85141	0.516489
CHMP4B	8.00323	0.661428
CHMP4C	5.36471	0.876462
CHMP5	8.96365	0.881375
CHMP7	6.29185	0.480022
CHN1	5.07744	0.429939
CHODL	5.26543	0.579827
CHORDC1	5.47844	0.479049
CHP1	8.4421	0.559562
CHPF	7.62699	0.50629
CHPF2	7.91079	0.448817
CHRAC1	6.48897	0.469409
CHRNB1	5.50799	0.43551
CHRNG	5.62803	0.408559
CHST11	7.03798	0.748047
CHST12	8.1618	0.508777
CHST14	6.59325	0.471041
CHST15	7.6367	0.466093
CHST2	5.34219	0.836646
CHST7	4.97044	0.708447
CHST8	5.49834	0.417894
CHSY1	8.68856	0.8022
CHSY3	6.12334	1.49719
CHTF18	5.85187	0.457766
CHTOP	7.68771	0.45153
CHUK	6.45294	0.682034
CHURC1	7.37219	0.699491
CIAPIN1	5.54757	0.400214
CIB1	8.07723	0.637352
CIDEB	6.61563	0.56468
CIPC	5.54153	0.793784
CIRBP	7.95585	0.445623
CIRH1A	7.12369	0.424922
CISD1	7.45203	0.699363
CISH	6.30803	0.7297
CITED2	8.84771	0.730379
CKAP2	6.4524	0.6445
CKAP4	7.97694	0.442141
CKAP5	6.87941	0.609809
CKB	6.14069	0.722339
CKLF	7.87597	0.834421
CKLF-CMTM1	8.81642	0.92933
CKMT2-AS1	5.71463	0.524239
CKS1B	7.81785	1.10388
CKS2	8.74284	1.38056
CLASP2	5.87615	0.467316

CLC	5.80506	1.6596
CLCC1	7.13114	0.624113
CLCN3	6.9588	0.596062
CLDN7	4.89041	0.50655
CLDND1	6.19059	0.405986
CLEC2B	5.24399	1.46459
CLEC2L	4.81875	0.411291
CLECL1	5.69034	0.807604
CLIC1	10.8593	0.568051
CLIC2	7.57217	1.79798
CLIC4	8.7951	0.705963
CLINT1	9.0136	0.635595
CLK1	9.26651	0.637612
CLK2	7.9292	0.583194
CLK4	6.37366	0.63052
CLMN	5.60317	0.673067
CLN3	6.97064	0.535837
CLN5	6.70098	0.744922
CLNS1A	7.76681	0.522466
CLOCK	6.49891	0.45038
CLPP	8.32845	0.446424
CLPTM1	8.02265	0.479234
CLPX	6.98746	0.697608
CLSTN1	7.83905	0.45097
CMAHP	6.12848	0.727751
CMC1	7.54356	0.861951
CMC2	8.6575	0.536033
CMC4	6.43135	0.549809
CMPK2	7.49782	1.93894
CMSS1	8.22079	0.733799
CMTM7	6.14665	0.656389
CMTR1	7.08042	0.478941
CMTR2	6.50581	0.850735
CNEP1R1	6.83056	0.710283
CNIH1	8.42916	0.702752
CNKSR1	5.38792	0.667909
CNN2	7.50858	0.421739
CNNM3	6.66077	0.540978
CNOT10	6.5581	0.50216
CNOT11	7.94383	0.405933
CNOT6	6.5049	0.596798
CNOT6L	5.61821	0.401247
CNOT7	7.43084	0.611874
CNOT8	7.08837	0.464473
CNPPD1	6.69569	0.45475
CNPY3	6.73555	0.48411
CNST	5.77475	0.487907

CNTD2	7.61173	0.455856
CNTN1	4.95581	1.11739
CNTNAP1	5.99982	0.478163
CNTRL	6.35727	0.555271
COA4	8.80971	0.55347
COA5	7.13459	0.453082
COA6	7.24705	0.83635
COA7	6.20497	0.524523
COASY	7.45989	0.444938
COBLL1	5.66609	0.949245
COCH	6.33045	1.12789
COG2	5.94356	0.401646
COG3	5.59874	0.570595
COG5	6.52052	0.51802
COG7	6.52049	0.451342
COIL	6.71054	0.534059
COL13A1	5.12429	0.453679
COL24A1	4.95951	1.10087
COL4A3BP	7.33317	0.475636
COL4A4	5.40952	0.536032
COL4A5	5.03131	0.912885
COL9A2	5.3988	0.443239
COL9A3	5.77618	0.924947
COLCA2	5.58115	1.37439
COLGALT1	5.87778	0.416122
COMMD1	8.82067	0.551848
COMMD10	6.25793	0.409507
COMMD2	5.56999	0.569152
COMMD3	11.5276	0.813518
COMMD3-BMI1	11.4923	0.689983
COMMD8	7.95815	0.776295
COMMD9	7.12474	0.524156
COMTD1	6.8701	0.526034
COPA	6.22605	0.445537
COPG1	8.59504	0.6312
COPRS	8.0619	0.50863
COPS2	7.79909	0.611239
COPS3	7.92076	0.518546
COPS5	8.63171	0.588351
COPS6	8.22948	0.466196
COPS7A	6.82911	0.527055
COPZ2	5.86241	0.55856
COQ10A	6.91328	0.509854
COQ2	5.03342	0.481373
COQ3	6.4323	0.636348
COQ4	7.23761	0.45515
COQ5	7.36802	0.637482

CORO1A	8.74273	1.11613
CORO1C	8.12615	0.877702
CORO7	5.53105	0.458598
CORT	5.03193	0.415174
COX14	8.19946	0.488039
COX15	6.25622	0.42942
COX16	7.14873	0.587179
COX17	6.62302	0.456947
COX20	7.69485	0.554117
COX3	7.94594	0.674098
COX5A	8.55578	0.471556
COX6B1	10.8371	0.487469
COX6C	11.2053	0.477644
COX7A2	11.5337	0.605021
COX7A2L	9.45666	0.488522
COX7A2P2	8.81101	0.441916
COX7B	7.34487	0.426252
COX7BP1	8.40262	0.455628
COX7C	11.1832	0.421809
COX8A	10.1405	0.469603
CPD	5.67691	0.616547
CPEB2	5.8207	0.542884
CPEB4	8.85902	0.839786
CPED1	5.54426	0.835078
CPNE1	7.80496	0.483085
CPNE3	8.57797	0.756433
CPNE5	9.45207	1.04188
CPNE7	6.41465	0.441178
CPOX	6.58609	0.672981
CPQ	6.73947	1.08629
CPSF2	6.57556	0.591416
CPSF3	7.85796	0.636214
CPSF6	6.826	0.539249
CPT1A	5.55631	0.435262
CPT1B	5.38067	0.636478
CPT2	6.12825	0.491013
CPTP	6.93891	0.438659
CPVL	5.44874	2.04941
CR936796	5.92421	1.68129
CRADD	6.78626	0.427458
CRB3	6.97435	0.41282
CRBN	8.67698	0.558452
CREB1	6.43525	0.470234
CREB3	8.09197	0.408305
CREB3L2	7.77489	0.481126
CREB3L4	6.24746	0.620383
CREBL2	8.13067	0.573061

CREBRF	7.15779	0.803023
CREBZF	6.5917	0.520478
CREG1	8.93377	0.840048
CRELD1	6.30872	0.454703
CRELD2	10.601	0.761127
CRIP1	5.95122	2.01249
CRIP2	4.98167	0.417226
CRIPAK	6.83428	0.508489
CRIPT	7.3636	0.500822
CRK	7.27876	0.481297
CRKL	6.00753	0.477549
CRLF1	5.29579	0.409082
CRLF3	7.86812	0.653368
CRNDE	6.51791	1.76066
CRNKL1	6.46604	1.01098
CRTAP	7.53413	0.495427
CRTC3	5.62199	0.592812
CRY1	6.12909	1.01717
CRYBB1	5.25223	0.556302
CRYBB2	5.52405	0.424872
CRYBG3	7.34729	0.868949
CRYL1	6.34819	0.63348
CRYM	5.79696	0.467968
CRYZ	7.1429	1.12079
CRYZL1	5.93759	0.458875
CS	9.04619	0.446714
CSAG2	5.13363	0.778547
CSAG3	5.13363	0.778547
CSDE1	10.0367	0.63594
CSE1L	6.92247	0.606419
CSF2RB	8.30917	1.9121
CSF3R	5.15613	0.577491
CSGALNACT1	6.62367	1.08233
CSGALNACT2	6.54321	0.560554
CSK	8.16024	0.603906
CSNK1A1	6.86526	0.422856
CSNK1G3	7.20516	0.663269
CSNK2A1	6.88795	0.402124
CSNK2A2	6.59393	0.459153
CSPP1	5.78907	0.450528
CSRNP1	8.07643	0.833546
CSRP1	8.97906	0.502896
CST3	5.25548	0.716511
CSTA	5.18388	1.29038
CSTF1	5.86154	0.514947
CSTF2	5.15252	0.49681
CSTF2T	6.46097	0.741273

CSTF3	6.48859	0.622851
CTA-250D10.23	5.84306	0.87376
CTA-292E10.6	6.88097	1.43373
CTA-29F11.1	7.42458	0.842596
CTA-445C9.15	4.95886	0.51874
CTAGE15	7.26242	0.424784
CTB-12A17.3	5.93255	0.553751
CTB-31O20.2	6.5852	0.767697
CTB-50L17.7	5.71649	0.474415
CTBS	7.463	0.672647
CTC-265F19.1	5.65388	0.67572
CTC-338M12.4	5.18418	0.455792
CTC-425F1.4	8.44144	0.453516
CTC-428G20.3	6.09674	0.53523
CTC-429P9.3	6.60552	0.43879
CTC-444N24.11	6.54257	0.726882
CTC-459F4.3	6.48513	0.482175
CTCF	8.10324	0.479847
CTD-2314B22.3	6.10777	2.2316
CTD-2553C6.1	5.72972	0.495604
CTD-3092A11.2	5.43621	1.00573
CTD-3126B10.1	5.54117	0.416754
CTD-3222D19.5	6.06447	0.748345
CTDNEP1	8.16317	0.42226
CTDSP1	6.98591	0.427078
CTDSP2	7.7155	0.425974
CTDSPL2	6.09893	0.661074
CTF1	5.3257	0.40822
CTGF	6.20584	2.09031
CTH	5.832	1.01204
CTHRC1	7.77256	2.73125
CTNNA2	5.12631	1.357
CTNNAL1	6.17453	1.01172
CTNNB1	6.33109	0.447173
CTNS	5.45679	0.473193
CTPS1	5.93528	0.886352
CTR9	7.93347	0.852389
CTSA	8.28399	0.667374
CTSB	7.09988	0.76358
CTSC	6.84739	0.718393
CTSD	6.87817	0.46456
CTSF	7.62646	0.76757
CTSG	6.09317	0.966785
CTSH	5.75533	1.56867
CTSK	5.14852	0.540484
CTSL	6.02695	0.727469
CTSO	8.7113	0.869385

CTSS	9.36416	0.715557
CTSW	6.10502	1.27091
CTSZ	6.85562	0.417036
CUL2	5.72454	0.486609
CUL4B	6.85452	0.73812
CUL5	6.35576	0.498768
CUTA	11.1498	0.514545
CUTC	6.52676	0.64702
CUX1	6.75561	0.528371
CWC15	9.05321	0.501554
CWC22	6.52677	0.538973
CWC27	6.98482	0.560087
CXCL12	5.57354	1.5299
CXCL16	5.20989	0.436985
CXCL8	6.27289	1.96705
CXCR4	10.2752	1.6264
CXorf21	6.59347	0.649721
CXorf38	5.22944	0.462255
CXorf40A	7.6183	0.553062
CXorf40B	7.62756	0.547286
CXXC1	8.36031	0.505982
CXXC4	6.28284	0.809585
CXXC5	9.53706	0.713728
CYAT1	6.95597	0.508545
CYB561	6.47073	0.409023
CYB561A3	7.09014	0.481824
CYB5A	6.13424	0.688441
CYB5B	4.98049	0.408297
CYB5D1	6.29206	0.609646
CYB5D2	7.23711	0.542162
CYB5R3	7.6844	0.449463
CYB5R4	5.5182	0.498911
CYBA	9.17936	0.420701
CYBB	5.23631	0.876022
CYC1	8.99624	0.553984
CYCS	8.26782	0.650151
CYFIP1	8.11148	0.771609
CYFIP2	5.95935	1.03027
CYLD	6.13638	0.683757
CYP11B2	5.54461	0.508062
CYP20A1	7.50609	0.675674
CYP27B1	5.37941	0.734543
CYP2E1	5.27572	0.443793
CYP2J2	5.91815	1.15128
CYP2R1	6.04632	0.495435
CYP2W1	5.73354	0.470021
CYP51A1	8.29223	0.674306

CYSLTR1	6.97116	0.738813
CYSTM1	8.35838	0.671811
CYTH1	7.00242	0.580536
CYTH4	6.55371	0.505001
CYTIP	10.2984	0.601324
DAAM1	5.39237	0.64509
DAAM2	4.83509	0.443344
DAD1	10.2827	0.546881
DALRD3	6.38345	0.401467
DANCR	10.5561	0.664532
DAP	8.72428	0.522654
DAPK1	6.37064	1.181
DAPK1-IT1	5.6852	1.04359
DAPP1	6.59797	0.603166
DARS	8.49736	0.540672
DARS2	6.06346	0.622618
DBF4	8.28953	0.69016
DBH-AS1	5.23021	0.447153
DBI	10.0817	0.596646
DBN1	6.27447	0.689772
DBNL	6.66954	0.400197
DCAF10	5.5719	0.447835
DCAF12	7.146	0.683725
DCAF13	5.40739	0.50398
DCAF16	6.1387	0.522746
DCAF17	5.33753	0.597513
DCBLD1	6.26679	0.759257
DCK	5.6673	1.18404
DCLRE1A	5.70248	0.576317
DCP1A	6.99685	0.445547
DCP1B	6.35877	0.611909
DCP2	4.95178	0.419305
DCPS	7.97802	0.63571
DCTD	7.78313	0.41575
DCTN1	6.95012	0.40393
DCTN3	9.14125	0.494122
DCTN4	6.30893	0.43973
DCTN6	8.41168	0.503919
DCTPP1	7.65169	0.59065
DCUN1D1	5.91167	0.486783
DCUN1D2	5.67309	0.408495
DCUN1D4	5.82096	0.602662
DCUN1D5	5.55242	0.438319
DCXR	8.20818	0.588639
DDAH2	6.5845	0.464442
DDB2	6.35567	0.648728
DDHD1	6.23671	0.573053

DDHD2	6.28384	0.818343
DDIAS	5.86732	0.808157
DDIT3	8.05329	0.868526
DDIT4	9.74996	1.33176
DDN	6.22377	0.414326
DDOST	10.8796	0.465706
DDR1	5.60781	0.473182
DDR2	5.7334	0.580036
DDRGK1	8.30054	0.49395
DDT	7.93742	0.605018
DDTL	7.93742	0.605018
DDX1	9.45401	0.454694
DDX11	6.8517	0.534442
DDX11L2	5.26276	0.458819
DDX12P	6.73377	0.666891
DDX17	8.49421	0.892571
DDX18	8.42365	0.507776
DDX20	4.89591	0.513548
DDX21	9.34458	0.498111
DDX23	6.83273	0.483036
DDX24	8.38722	0.544662
DDX26B	6.43559	0.810393
DDX39A	9.54999	0.655071
DDX39B	7.57275	0.550332
DDX3Y	5.19556	1.49035
DDX41	7.62186	0.471927
DDX46	7.72428	0.438067
DDX47	8.9057	0.485314
DDX5	9.72152	0.44455
DDX50	6.39182	0.403942
DDX55	5.83156	0.549056
DDX58	5.84998	0.702772
DDX59	6.60482	0.428462
DDX6	6.72039	0.464558
DECR1	8.45687	0.539235
DEDD	6.34584	0.430903
DEDD2	8.40324	0.434152
DEF6	7.2603	0.423326
DEF8	7.2016	0.468277
DEFA1	8.56391	2.35235
DEFA1B	8.56391	2.35235
DEFA3	8.56391	2.35235
DEFA4	6.21207	1.23505
DEFB4A	4.83981	0.553085
DEFB4B	4.83981	0.553085
DEGS1	8.57118	0.584506
DEGS2	4.89567	0.463834

DEK	5.40749	1.49464
DENND1B	6.65421	0.752989
DENND1C	6.69064	0.634514
DENND2C	6.1765	0.860035
DENND2D	5.43299	0.506002
DENND3	6.77777	0.834775
DENND4A	6.2582	0.750419
DENND4B	7.52212	0.568625
DENND4C	5.42855	0.762779
DENND5B	6.4135	0.474677
DENND6A	7.8602	0.593312
DENND6B	5.74469	0.61663
DEPTOR	6.23163	0.999528
DERA	7.36527	0.706587
DERL1	9.1369	0.414499
DERL2	8.51356	0.500293
DERL3	8.00361	0.691339
DESI1	7.13822	0.543055
DET1	5.42017	0.478814
DEXI	7.10061	0.432955
DFFB	5.50303	0.564834
DFNB31	6.19501	0.773951
DGAT1	6.52148	0.52621
DGAT2	6.13137	0.71736
DGCR6	7.26562	0.526267
DGKA	5.67988	0.569828
DGKD	7.35754	0.611825
DGKQ	5.00058	0.533189
DHCR7	6.54711	0.529887
DHFR	6.15504	0.734969
DHFRL1	5.52613	0.56639
DHPS	8.0607	0.512915
DHRS1	7.00454	0.542058
DHRS11	5.25882	0.598715
DHRS13	7.06685	0.509322
DHRS3	5.40289	0.478833
DHRS4	7.41091	0.442677
DHRS4L2	7.41091	0.442677
DHRS7B	6.80916	0.743367
DHRS9	6.44074	2.08065
DHTKD1	6.2614	0.602465
DHX15	9.62035	0.473181
DHX16	7.54561	0.453095
DHX29	7.82151	0.732802
DHX32	7.72068	0.487334
DHX33	6.86714	0.542418
DHX40	7.16838	0.54587

DHX9	7.21131	0.602306
DIABLO	8.10432	0.423793
DIEXF	5.33499	0.753842
DIMT1	7.14638	0.482472
DIS3	5.28539	0.451635
DIS3L	7.39677	0.504514
DIS3L2	5.75673	0.422759
DKC1	8.43967	0.685712
DKFZP434I0714	6.97683	0.972807
DKFZP586I1420	8.33018	0.598234
DKFZp667J0810	6.78241	1.93477
DKK1	8.44938	2.33703
DLAT	7.03673	0.72658
DLD	7.11138	0.540713
DLEC1	5.68528	0.442832
DLEU1	7.54305	0.825565
DLG1	6.36009	0.498223
DLST	6.70114	0.549828
DMAP1	6.05324	0.422116
DMRT2	5.50828	1.14559
DMTF1	8.81884	0.444765
DMXL1	6.91016	0.748669
DMXL2	4.92891	0.705907
DNA2	5.4403	0.515129
DNAAF1	6.15593	0.845865
DNAAF2	6.91834	0.650153
DNAJA1	9.60216	0.459981
DNAJA2	7.34161	0.561217
DNAJA3	7.46882	0.554917
DNAJB1	7.99568	0.634176
DNAJB11	10.8922	0.530572
DNAJB14	7.76415	0.695754
DNAJB2	5.92587	0.480173
DNAJB4	4.87217	0.531153
DNAJB6	7.11667	0.576813
DNAJB9	10.4138	0.664784
DNAJC1	11.0412	0.500972
DNAJC10	7.82062	0.650554
DNAJC12	5.67918	1.05356
DNAJC14	6.55477	0.509747
DNAJC15	7.21105	0.650715
DNAJC17	5.75728	0.408389
DNAJC18	5.0929	0.400853
DNAJC19	7.57068	0.468169
DNAJC24	5.57763	0.474209
DNAJC25	7.4059	0.648782
DNAJC25-GNG10	7.4059	0.648782

DNAJC3	8.72254	0.753379
DNASE1L1	7.22758	0.636655
DNASE2	7.64358	0.596478
DNMT1	7.41195	0.786221
DNMT3B	5.03862	0.70418
DNPH1	7.38083	0.512061
DNTTIP1	6.8747	0.464227
DNTTIP2	8.59534	0.602356
DOCK10	5.03745	0.811844
DOCK11	5.69003	1.0937
DOCK7	4.88281	0.431552
DOCK8	6.85183	0.553901
DOK3	6.7056	0.769586
DOLK	6.68758	0.63871
DONSON	7.10254	0.824283
DPAGT1	8.23443	0.60151
DPCD	6.70715	0.677469
DPEP1	6.67841	1.11616
DPEP3	4.91501	0.504579
DPF2	7.81971	0.660698
DPH2	7.22128	0.43122
DPH3	7.28374	0.594907
DPH5	7.15659	0.490318
DPH7	7.63131	0.430471
DPM1	9.35555	0.569058
DPM2	7.75218	0.400957
DPM3	8.28463	0.948068
DPP3	7.1648	0.620134
DPP7	6.50468	0.481705
DPP8	7.20561	0.485971
DPY19L3	7.23112	0.653343
DPY19L4	4.83712	0.521554
DPY30	7.68752	0.451638
DQ592230	5.84182	1.45981
DQ592442	4.88537	0.854053
DQ597730	5.02745	0.622469
DQ786293	6.10777	2.2316
DRAM1	4.96325	0.634489
DRAM2	7.28082	0.760235
DRAP1	7.86467	0.536285
DRG1	8.53347	0.488952
DROSHA	7.73101	0.525923
DSERG1	6.78325	0.751257
DSN1	5.36046	0.494517
DST	5.47487	0.558279
DTD2	6.24385	0.615292
DTL	5.77384	1.46501

DTNBP1	6.46205	0.426224
DTWD1	5.54514	0.421306
DTX1	5.66706	0.512262
DTX3L	8.17739	0.97657
DUS1L	8.50258	0.47185
DUS4L	5.18269	0.438347
DUSP1	9.3596	0.574146
DUSP10	5.93209	1.10403
DUSP11	7.51993	0.548285
DUSP12	8.14922	0.562828
DUSP13	5.28185	0.56582
DUSP14	5.31426	0.583219
DUSP2	7.73459	1.58695
DUSP22	9.61901	0.818201
DUSP23	6.62515	0.661481
DUSP26	7.05375	0.783009
DUSP28	7.53263	0.486092
DUSP4	6.75598	1.75827
DUSP5	10.6195	1.07955
DUSP6	8.07572	1.66394
DUSP7	5.18462	0.429354
DUT	8.77358	0.56404
DUXAP10	4.82007	1.29036
DVL1	7.35657	0.477885
DXO	7.12731	0.523544
DYNC1LI1	7.45013	0.490142
DYNLL1	10.8112	0.499365
DYNLT1	9.07316	0.696862
DYNLT3	7.09671	0.772117
DYRK2	5.16468	0.622579
DYRK4	7.05531	0.537547
DYSF	6.48478	0.43829
DYX1C1-CCPG1	8.31804	0.643356
DZIP3	6.01442	0.590293
E2F2	5.60328	0.420694
E2F3	7.08377	0.551853
E2F5	7.45423	0.766926
E2F6	6.3339	0.720551
EAF1	6.09007	0.642513
EAF2	6.35033	0.736867
EAPP	8.37481	0.59893
EARS2	5.80873	0.408788
EBAG9	9.01894	0.519922
EBLN2	6.67281	0.500925
EBLN3	9.22634	0.505004
EBNA1BP2	7.74088	0.6178
EBPL	8.58621	0.836977

ECD	6.93008	0.609374
ECHDC1	7.84423	0.684761
ECHDC2	6.09281	0.605718
ECHDC3	5.44461	0.683463
ECHS1	8.39041	0.531242
ECI1	7.6276	0.449118
ECI2	8.43202	0.524124
ECSIT	6.97922	0.52098
EDEM1	8.94147	0.777278
EDEM2	8.34398	0.714904
EDEM3	8.13338	0.957062
EDN1	5.09307	0.648955
EDNRB	7.3888	3.23693
EDNRB-AS1	5.13202	1.17836
EDRF1	5.09234	0.48722
EED	5.53762	0.602622
EEF1D	8.90299	0.485001
EEF1DP5	5.25406	0.409895
EEF1E1	6.32645	0.579305
EEF1E1-BLOC1S5	6.6558	0.563247
EEF2	12.5872	0.516119
EEF2K	6.57483	0.55091
EFCAB4A	6.52852	0.525994
EFCAB7	5.52627	0.827472
EFHC1	5.58054	0.687311
EFHD2	5.87073	0.513144
EFNA4	5.32717	0.485495
EFR3A	7.59884	0.680239
EFTUD1	6.40128	0.635047
EGFL7	5.61066	0.410622
EGLN1	6.32172	0.411221
EGR1	9.93712	1.5377
EGR2	6.10933	1.19648
EHBP1	6.54041	0.533661
EHBP1L1	6.21141	0.509337
EHD1	6.02696	0.634625
EHD3	5.22564	0.692913
EHD4	5.26083	0.556205
EI24	7.59118	0.589043
EID1	9.32262	0.606539
EID2	6.30036	0.714573
EIF1AD	6.59313	0.446786
EIF1AX	8.48808	0.540932
EIF1AY	5.40482	2.22228
EIF1B	6.52447	0.451832
EIF2A	10.9812	0.624772
EIF2AK1	7.17639	0.44141

EIF2AK2	6.8846	0.614434
EIF2AK3	7.25088	0.461301
EIF2AK4	6.1152	0.569605
EIF2B2	7.13821	0.548238
EIF2B3	7.49323	0.491947
EIF2D	8.90027	0.576882
EIF2S1	8.08329	0.587936
EIF2S3	8.92536	0.691035
EIF3A	8.8452	0.421944
EIF3B	7.0726	0.455614
EIF3C	8.98027	0.467174
EIF3CL	10.4597	0.544303
EIF3D	10.6634	0.536544
EIF3E	8.68262	0.52101
EIF3F	6.87184	0.483937
EIF3G	10.2324	0.54464
EIF3H	7.55196	0.507121
EIF3I	9.25526	0.42549
EIF3J	8.24177	0.667418
EIF3J-AS1	5.5684	0.632042
EIF3L	12.0696	0.530266
EIF3LP3	4.88526	0.413081
EIF3M	6.59499	0.432119
EIF4A1	10.5157	0.552191
EIF4A3	8.55739	0.734564
EIF4B	9.71679	0.604999
EIF4E3	6.33751	0.644265
EIF4EBP1	7.14892	0.932248
EIF4EBP3	7.188	0.694718
EIF4G1	7.77427	0.567389
EIF4G3	5.72079	0.447236
EIF5	8.81462	0.655325
EIF5A	8.74668	0.756137
EIF6	8.39942	0.476899
ELANE	5.04717	1.0698
ELF1	8.24777	0.696164
ELF2	6.80568	0.477632
ELF4	5.32863	0.534344
ELFN1-AS1	5.34655	0.626484
ELK3	6.896	0.580724
ELL2	8.48476	0.752392
ELMO1	7.18734	0.700969
ELOVL1	7.39797	0.427483
ELOVL7	6.63356	1.7234
ELP3	6.48088	0.559742
ELP4	7.0134	0.645121
ELP5	6.98825	0.434929

EMB	7.14399	0.4876
EMC2	7.77372	0.792613
EMC6	8.41919	0.455839
EMC7	7.61768	0.515339
EMC8	6.82742	0.427537
EMC9	7.03075	0.457302
EMD	7.07721	0.45109
EME1	6.3206	0.49427
EMG1	7.675	0.648791
EML4	6.46443	0.579802
EML6	6.1825	0.940875
EMP3	9.58069	1.09212
ENC1	4.84663	0.931755
ENDOD1	5.07063	0.636147
ENDOG	6.85139	0.608197
ENKD1	5.71906	0.424609
ENO1	7.0426	0.461571
ENO2	6.11552	1.19647
ENOPH1	8.94362	0.57797
ENOSF1	6.70777	0.772123
ENPP4	5.78576	0.975757
ENPP7	5.42289	0.481977
ENSA	7.58447	0.588599
ENTPD1	4.90761	1.13115
ENTPD4	7.10846	0.632409
ENY2	6.03522	0.478534
EP300	5.88169	0.587441
EPAS1	5.33301	0.749628
EPB41L2	4.82799	0.6038
EPB41L4A	5.26039	0.573457
EPB41L4A-AS1	7.45807	0.63665
EPC1	7.32048	0.480383
EPC2	5.79485	0.648277
EPHB6	4.9822	0.453256
EPHX1	7.11685	0.888509
EPHX2	6.11963	1.09973
EPM2AIP1	7.47361	0.561406
EPRS	9.56004	0.56593
EPS15	7.85543	0.700649
EPS8	4.97483	1.21901
EPSTI1	6.42984	1.07776
EPT1	6.30715	0.429317
EPX	5.08242	0.777827
ERAL1	6.37585	0.480614
ERAP2	6.27616	1.11435
ERBB2IP	6.77364	0.556705
ERCC1	7.2936	0.469655

ERCC3	6.55653	0.433892
ERCC4	5.14303	0.504249
ERCC5	7.16542	0.619361
ERGIC1	7.00278	0.471579
ERGIC2	7.83695	0.519636
ERH	9.89923	0.565153
ERI3	6.41547	0.410089
ERLEC1	8.4938	0.515443
ERLIN1	6.01199	0.662151
ERLIN2	5.49963	0.61878
ERMARD	6.79538	0.498449
ERMP1	5.54986	0.597177
ERN1	7.84234	0.672601
ERO1L	7.61859	0.558153
ERO1LB	6.69589	0.933389
ERP29	10.7014	0.475156
ERV3-2	7.06403	1.11465
ERVK3-1	6.58319	0.584436
ESAM	5.42157	0.474386
ESCO1	6.47872	0.825878
ESF1	5.78339	0.612392
ESPL1	5.26769	0.489312
ESRRG	6.42626	1.48721
ESYT1	7.21329	0.600542
ESYT2	5.89677	0.453214
ETAA1	5.36404	0.435192
ETF1	8.27058	0.521223
ETFA	9.21076	0.844464
ETFB	7.44697	0.905411
ETFDH	5.78243	0.679276
ETHE1	7.51569	0.560867
ETNK1	6.31055	0.529287
ETNK2	4.82157	0.42137
ETS1	4.97228	1.00948
ETV1	5.16796	1.206
ETV3	5.85526	0.436985
ETV5	5.5345	0.531887
ETV6	6.24483	0.578158
EVI2A	9.72231	0.930427
EVI2B	11.288	0.777854
EVL	6.02144	0.713586
EWSR1	6.89658	0.422897
EXD2	5.83795	0.422209
EXO1	5.33915	0.852771
EXOC1	7.82283	0.574772
EXOC2	5.55233	0.529093
EXOC5	5.62271	0.585071

EXOC6	5.3691	0.425126
EXOC8	6.26163	0.907579
EXOSC3	5.40373	0.420265
EXOSC4	7.28015	0.510566
EXOSC5	7.83628	0.605245
EXOSC6	6.19776	0.422359
EXOSC8	7.20178	0.80328
EXOSC9	7.5831	0.415449
EXT1	5.01804	0.407177
EXT2	6.8554	0.574169
EXTL2	6.89434	1.01412
EYA2	4.98309	1.0423
EZH2	5.94859	1.11164
EZR	8.08542	0.802236
F11R	6.61233	0.458326
F12	5.41894	0.537001
F13A1	4.86015	0.968302
F2R	4.83563	1.06131
F8	5.65844	0.810208
F8A1	8.56066	0.735448
F8A2	8.56066	0.735448
F8A3	8.56066	0.735448
FAAH	5.68546	0.477091
FAAH2	6.83014	0.842891
FABP5	6.20016	1.41667
FADD	6.3234	0.679729
FADS3	6.14275	0.542603
FAF2	7.84708	0.441026
FAHD1	5.35789	0.59857
FAIM	6.88988	0.784374
FAIM3	6.09125	1.24442
FAM101B	7.43246	1.71453
FAM102B	5.41961	0.896771
FAM103A1	7.78726	0.579879
FAM104A	7.66952	0.521421
FAM106A	5.38465	1.11908
FAM107B	7.34407	1.8214
FAM109B	5.42806	0.425183
FAM111A	5.83391	0.486891
FAM114A1	5.95158	0.679502
FAM114A2	7.30874	0.525109
FAM117A	8.41963	0.695919
FAM118A	5.47249	0.630452
FAM118B	4.94844	0.402461
FAM120B	6.08756	0.406898
FAM120C	5.01463	0.42218
FAM122A	6.06992	0.556815

FAM122B	7.11544	0.7929
FAM126A	5.42417	0.81778
FAM126B	6.04056	0.619941
FAM127A	7.8858	0.602476
FAM127B	5.74927	0.557609
FAM129A	6.33604	1.76062
FAM133A	6.63284	1.74158
FAM133B	6.05607	0.684055
FAM133DP	6.05607	0.684055
FAM134A	6.9513	0.402732
FAM134C	8.69852	0.541846
FAM136A	8.34869	0.417132
FAM13A	6.43598	0.820992
FAM13A-AS1	5.59451	0.644575
FAM13B	6.84989	0.7554
FAM149A	5.54274	0.791139
FAM160A2	6.54275	0.440414
FAM162A	7.54929	0.448649
FAM171A1	5.82408	1.49197
FAM172A	6.11079	0.542446
FAM173A	7.31565	0.576052
FAM173B	5.68407	0.436561
FAM174A	9.29616	0.991228
FAM175A	5.30087	0.745716
FAM175B	6.37814	0.546554
FAM177A1	6.07812	0.581604
FAM178A	5.15299	0.411206
FAM179B	6.58445	0.812304
FAM186B	4.92693	0.454075
FAM188A	6.97824	0.922641
FAM189A2	4.96454	0.545629
FAM193A	6.34123	0.474045
FAM195A	7.46808	0.546019
FAM199X	6.52038	0.514822
FAM19A5	5.37436	1.38062
FAM206A	8.06645	0.501724
FAM208A	5.98886	0.418297
FAM20B	6.73127	0.642987
FAM210A	5.10597	0.431918
FAM210B	6.64451	0.653375
FAM213A	6.45713	0.859367
FAM213B	6.15802	0.584062
FAM214A	8.51869	0.758102
FAM216A	5.93897	0.918797
FAM217B	6.9699	0.792937
FAM219A	6.52885	0.431255
FAM21A	7.74507	0.408493

FAM21C	7.86529	0.416836
FAM220A	8.07663	0.720302
FAM221A	6.92172	0.692121
FAM228B	6.17449	0.549672
FAM229B	5.26015	0.749943
FAM24B	5.22159	0.479618
FAM26F	5.14632	0.997701
FAM32A	8.07592	0.43884
FAM35A	8.55957	0.616367
FAM3C	6.99071	0.734947
FAM43A	4.89865	0.447271
FAM43B	5.26217	0.400788
FAM45B	6.89384	0.524719
FAM46A	5.08729	0.661358
FAM46C	10.5764	0.670145
FAM47E-STBD1	5.71479	0.890259
FAM49A	6.49724	1.16173
FAM50A	7.66247	0.613164
FAM50B	5.92691	0.727222
FAM53C	7.436	0.437661
FAM57B	6.22167	0.40505
FAM58A	7.20362	0.517325
FAM60A	7.86961	0.935407
FAM63B	5.89229	0.605091
FAM65A	6.22479	0.413152
FAM69A	7.33758	0.864198
FAM71E1	4.86339	0.462417
FAM71F2	4.83187	0.403481
FAM72A	5.96663	1.60338
FAM72B	5.96663	1.60338
FAM72C	5.96663	1.60338
FAM72D	5.96663	1.60338
FAM73B	6.63841	0.415099
FAM76B	5.83783	0.622388
FAM78A	5.93034	0.632089
FAM84B	5.4487	0.834635
FAM86B1	5.50515	0.614881
FAM86B2	5.50515	0.614881
FAM86C1	5.59205	0.472575
FAM86DP	5.50515	0.614881
FAM86FP	5.50515	0.614881
FAM89A	5.88319	0.801767
FAM89B	7.61368	0.511916
FAM8A1	8.68639	0.601657
FAM96A	10.3364	0.619347
FAM96B	8.73357	0.475113
FAM98A	7.47071	0.488988

FAM98B	5.48927	0.630272
FAM98C	6.80256	0.487175
FANCE	6.40948	0.409778
FANCF	7.10868	0.824897
FANCG	6.64541	0.580449
FANCI	5.56046	0.943454
FANCL	6.73076	0.902913
FAR1	6.23661	0.753259
FAR2	5.44757	1.02717
FARSA	7.03026	0.563872
FARSB	6.2616	0.546691
FASTKD1	7.77407	0.82041
FASTKD3	6.81462	0.892864
FASTKD5	6.78278	0.469362
FATE1	6.10413	0.481363
FBL	10.2515	0.706633
FBLN2	5.71949	1.00107
FBP1	7.36996	1.83643
FBXL16	5.37062	0.562819
FBXL20	5.24456	0.410221
FBXL4	5.19954	0.656922
FBXL5	7.69399	0.574656
FBXL7	5.03532	0.565186
FBXO11	6.28465	0.459954
FBXO16	6.54879	0.659948
FBXO22	5.57906	0.513626
FBXO25	7.8756	0.523357
FBXO28	6.12888	0.523125
FBXO30	4.94286	0.495678
FBXO32	5.11617	0.656045
FBXO33	7.30814	0.759489
FBXO34	7.01524	0.560384
FBXO38	6.53886	0.500563
FBXO45	5.61406	0.47482
FBXO46	7.57728	0.482364
FBXO5	5.27873	0.799525
FBXO6	5.58422	0.808707
FBXO7	7.35245	0.453017
FBXO8	6.58602	0.710212
FBXW11	6.64832	0.456213
FBXW7	8.72614	0.733987
FCER1G	5.23863	1.00695
FCER2	5.22024	1.20372
FCF1	5.06044	0.457399
FCGBP	5.46035	0.591998
FCGR2B	8.43264	1.76425
FCGR2C	6.35854	0.705176

FCGR3A	5.27437	1.26846
FCGR3B	5.61974	1.3091
FCGRT	7.50887	0.818462
FCHO1	6.02185	0.435095
FCHSD2	7.55239	0.858627
FCN1	5.21062	1.19767
FCRL2	6.37738	1.20988
FCRL3	4.88154	1.0184
FCRL5	7.73056	0.804889
FCRLA	8.27882	1.95253
FCRLB	7.17584	1.32854
FDFT1	7.28447	0.637314
FDPS	8.89577	0.608623
FDX1	7.6833	0.44043
FDXACB1	6.35928	0.614457
FDXR	5.0593	0.457853
FECH	5.49865	0.470695
FEM1B	6.28979	0.716557
FEM1C	6.81827	0.892966
FEN1	7.14448	0.918424
FER	6.165	0.763122
FER1L4	6.06533	0.72819
FERMT3	7.0704	0.526856
FGD2	5.87203	0.443817
FGD3	5.89301	0.400075
FGF13	5.58187	1.69781
FGFR1OP2	6.0763	0.485632
FGL2	5.52923	1.48224
FH	7.80671	0.706753
FHDC1	5.70657	0.48516
FHIT	5.61605	0.738996
FHL1	6.65897	1.59388
FHOD1	5.79772	0.469392
FIBP	8.07022	0.618521
FICD	8.38588	0.719291
FIG4	7.04468	0.654095
FIGNL1	4.96709	1.02818
FIP1L1	5.79151	0.405637
FIS1	9.55431	0.480606
FJX1	4.91542	0.588125
FKBP11	10.7146	0.666095
FKBP14	6.82693	0.733132
FKBP1A	7.03946	0.499885
FKBP2	10.4963	0.559975
FKBP5	7.74165	1.2687
FKBP9	5.44649	0.680216
FKTN	5.36915	0.721329

FLAD1	7.25956	0.567011
FLI1	7.09804	0.553133
FLJ10038	5.2994	0.686229
FLJ11710	5.49471	0.409592
FLJ16734	5.01954	0.568077
FLJ20021	7.29983	0.495524
FLJ22184	5.32432	0.427745
FLJ25758	5.33509	0.450248
FLJ30901	5.43501	0.457334
FLJ31306	6.21766	0.751026
FLJ32255	6.01697	0.806462
FLJ38717	5.79075	0.717142
FLJ46875	6.27104	0.478504
FLNA	6.78705	0.704816
FLNB	5.97363	0.573896
FLVCR1	4.91665	0.740935
FLVCR1-AS1	6.1213	0.67882
FMNL1	6.06718	0.447711
FMO4	5.55818	0.439606
FMR1	7.25034	0.968699
FN3KRP	7.41495	0.535778
FNBP1	6.31153	1.14129
FNBP4	5.71144	0.450632
FNDC3A	6.72596	0.439627
FNDC3B	8.06985	0.644222
FNIP1	4.94314	0.414441
FNIP2	5.8045	0.770978
FNTA	7.04843	0.458885
FOPNL	7.24132	0.595863
FOS	11.5394	1.23736
FOSB	10.2218	1.36052
FOXM1	4.83347	0.706327
FOXN2	6.16814	0.446253
FOXO1	5.6429	0.46598
FOXO3	8.58289	0.605705
FOXO3B	8.08138	0.654067
FOXO6	5.91846	0.513916
FOXP1	5.58672	0.695799
FOXP1-IT1	5.78314	0.510584
FOXRED1	6.93665	0.558251
FPR1	4.81835	0.690078
FRAT1	5.43159	0.530962
FRAT2	6.20027	0.61696
FRG1	7.07294	0.5241
FRG1B	5.75335	0.540881
FRMD6	6.25338	1.63563
FRZB	7.19459	1.91874

FTH1	9.61613	0.743993
FTH1P5	10.7673	0.525262
FTL	12.7207	0.461583
FTO	7.02057	0.504413
FTSJ2	6.96198	0.568066
FTSJ3	7.55938	0.501743
FTX	6.67568	0.890742
FUBP1	6.51466	0.516445
FUBP3	7.05506	0.451764
FUCA2	7.97385	0.95723
FUK	6.44611	0.443454
FUNDC1	6.12755	0.746721
FUOM	6.32804	0.647326
FUS	6.95518	0.449272
FUT11	5.25556	0.487685
FUT8	7.56195	1.34537
FUT8-AS1	5.25759	0.560547
FXN	5.69376	0.440046
FXYD5	10.0042	0.949949
FYN	4.84287	0.84056
FYTTD1	8.80163	0.512914
FZD2	5.46137	0.468414
FZD3	6.00208	0.821129
FZD6	7.15803	1.28251
FZD7	5.23805	1.21715
G0S2	7.21919	1.50602
G2E3	5.43836	0.652037
G3BP1	6.66097	0.414813
G3BP2	8.02594	0.512611
G6PC3	7.92598	0.497753
G6PD	6.43059	0.593614
GAA	6.59182	0.47325
GAB1	6.05433	0.470146
GAB2	7.06446	0.701377
GABARAP	10.4435	0.453058
GABARAPL1	7.38679	0.990559
GABARAPL2	9.64996	0.534147
GABARAPL3	6.00838	0.688493
GABPA	6.00332	0.583264
GABPB1	6.14741	0.588822
GABPB1-AS1	6.02185	0.741471
GABPB2	6.1613	0.513412
GADD45A	10.3331	1.4301
GADD45B	8.32278	0.987743
GAGE12B	4.96109	1.87345
GAGE12C	5.0126	1.83063
GAGE12D	5.0126	1.83063

GAGE12E	5.0126	1.83063
GAGE12F	4.96109	1.87345
GAGE12G	4.96109	1.87345
GAGE12H	5.0126	1.83063
GAGE12I	4.96109	1.87345
GAGE12J	5.04984	1.76431
GAGE13	5.04984	1.76431
GAGE2A	5.07514	1.84391
GAGE2B	5.51683	1.72929
GAGE2C	5.07514	1.84391
GAGE2D	5.04984	1.76431
GAGE2E	5.16227	1.75235
GAGE3	5.06576	1.62791
GAGE4	4.96109	1.87345
GAGE5	4.96109	1.87345
GAGE6	4.96109	1.87345
GAGE7	4.96109	1.87345
GAGE8	5.16227	1.75235
GALC	5.65227	0.601402
GALK2	6.84984	0.56156
GALM	6.32264	0.515759
GALNT1	7.79107	0.83211
GALNT10	5.66255	0.620725
GALNT11	6.48884	0.912391
GALNT12	5.51694	0.924806
GALNT2	7.54825	0.505887
GALNT3	5.59712	0.64632
GALNT4	5.43221	0.585714
GALNT6	5.29908	0.504675
GALNT7	6.16087	0.902163
GALNT9	6.28928	0.410387
GANAB	9.56795	0.434492
GAPDH	12.1396	0.55743
GAPDHP62	6.80704	0.659094
GAPDHP73	5.92698	0.45861
GAPVD1	6.53383	0.444483
GAR1	8.04606	0.571651
GARS	10.6004	0.63235
GART	5.42704	0.555511
GAS2	6.71166	2.31598
GAS5	8.93023	0.677035
GAS6	8.96652	0.996719
GATAD1	5.82091	0.419628
GATAD2B	5.69539	0.439171
GATSL2	6.6687	0.437454
GBA	7.01102	0.622781
GBA3	5.94553	2.2431

GBAP1	6.04172	0.40677
GBAS	7.85938	0.754957
GBE1	6.98494	0.772392
GBF1	6.75831	0.497018
GBP1	5.61053	1.06822
GBP2	5.43168	1.15273
GBP3	7.02512	1.2846
GBP5	6.03978	1.04532
GCA	6.40523	0.943426
GCAT	5.78994	0.603525
GCDH	7.34975	0.413099
GCFC2	5.72913	0.540607
GCH1	6.20422	1.20472
GCHFR	6.22078	0.595204
GCLC	5.67677	0.452674
GCN1L1	7.2427	0.455756
GCNT1	4.96135	0.582062
GCNT3	4.9786	0.48012
GCOM1	6.19508	0.417484
GCSH	7.74551	0.746718
GDAP2	5.89347	0.431374
GDE1	8.10002	0.434657
GDI1	8.15539	0.546537
GDI2	10.0458	0.464743
GEMIN2	5.49725	0.699746
GEMIN4	6.00343	0.505723
GEMIN5	6.57974	0.708096
GEMIN6	6.04141	0.419765
GFI1	6.10743	0.669577
GFM1	6.17525	0.479715
GFM2	6.57588	0.54585
GFOD1	5.06643	0.614503
GFPT1	7.41849	0.880511
GGCT	7.83905	0.784189
GGCX	6.17621	0.403388
GGH	8.29975	1.35015
GGNBP2	6.18339	0.430936
GGPS1	6.59994	0.637815
GGT1	6.22664	0.493767
GGT2	6.34417	0.572665
GGT3P	5.68224	0.426046
GGTLC1	6.26741	0.562732
GGTLC2	6.31625	0.496318
GHDC	6.71089	0.615982
GHITM	9.71332	0.426724
GID4	5.34078	0.414264
GID8	6.98291	0.410119

GIMAP2	6.28518	1.15639
GIN1	5.73799	0.694032
GINM1	8.42989	0.820074
GINS1	5.15395	1.32422
GIPC1	6.6353	0.462721
GKAP1	5.62454	0.427136
GLA	8.04825	0.678633
GLB1	8.82448	0.68948
GLB1L2	5.71399	0.404455
GLCCI1	8.87676	0.72538
GLDC	5.54265	1.49262
GLE1	6.14002	0.420706
GLG1	8.08951	0.451801
GLI3	4.80111	0.429021
GLIDR	6.79375	0.710276
GLIPR1	5.34068	1.0063
GLIPR2	6.01904	0.840858
GLIS1	4.89638	0.48783
GLIS3	4.83139	1.25279
GLMN	5.53638	0.631633
GLO1	10.3768	0.71463
GLOD4	6.92558	0.699974
GLRX	10.0067	0.872147
GLRX2	7.95264	0.941298
GLRX5	8.55732	0.573028
GLS	6.44541	0.400997
GLT1D1	5.24263	0.430811
GLT8D1	9.31418	0.667596
GLTP	7.28402	0.482286
GLTSCR1L	6.83861	0.587028
GLTSCR2	9.52003	0.468529
GLUD1	7.50007	0.623361
GLUL	5.9958	0.923967
GLYR1	6.25691	0.405305
GM2A	6.82664	0.567883
GMCL1	6.23816	0.674793
GMCL1P1	5.5575	0.661559
GMDS	5.70618	0.427815
GMFB	6.63521	0.729129
GMFG	9.04697	0.86039
GMNN	7.2583	1.03679
GMPPA	7.28233	0.488192
GMPR	5.76111	0.811689
GMPR2	7.79943	0.515503
GMPS	5.35015	0.414552
GNA11	5.57941	0.415197
GNA13	6.86469	0.53588

GNAI2	7.9861	0.46249
GNAI3	7.09655	0.578564
GNAZ	5.38586	0.449347
GNB1	8.81212	0.458585
GNB2	7.58615	0.49909
GNB2L1	9.77596	0.472575
GNB3	6.02783	0.401987
GNB5	5.73424	0.401898
GNE	7.44443	0.644603
GNG10	9.07037	0.696531
GNG5	10.3512	0.48226
GNG7	7.61671	0.436675
GNL2	7.7677	0.587362
GNL3	7.52783	0.440262
GNPAT	8.69095	0.60099
GNPDA1	6.90839	0.684521
GNPDA2	6.26842	0.709461
GNPNAT1	6.36493	0.75885
GNPTAB	5.97368	0.40183
GNPTG	8.12646	0.575048
GNRH2	5.50897	0.408998
GNS	7.31093	0.624895
GOLGA1	6.45707	0.450022
GOLGA2P5	5.4613	0.451549
GOLGA2P7	6.58855	0.404781
GOLGA4	8.42606	0.478298
GOLGA5	8.46328	0.649711
GOLGA6L11P	5.3029	0.434598
GOLGA6L16P	5.3029	0.434598
GOLGA6L4	6.62875	0.838701
GOLGA6L5P	6.62875	0.838701
GOLGA6L9	6.62875	0.838701
GOLGA7	7.97743	0.524243
GOLGA8A	7.90428	0.687341
GOLGA8B	7.80165	0.680691
GOLGA8CP	7.39884	0.44611
GOLGA8DP	7.39884	0.44611
GOLGA8EP	7.39884	0.44611
GOLGA8F	7.39884	0.44611
GOLGA8G	7.39884	0.44611
GOLGA8I	6.89271	0.438072
GOLGA8M	6.89271	0.438072
GOLGA8N	9.79361	0.893131
GOLGB1	7.89478	0.4245
GOLIM4	6.4668	0.406713
GOLM1	4.92396	0.741847
GOLPH3	9.66279	0.638057

GOLPH3L	8.32087	0.862843
GOLT1B	8.72552	0.535119
GON4L	6.27476	0.437326
GOPC	6.11189	0.492022
GORAB	7.161	0.749129
GORASP2	9.80637	0.483304
GOT1	7.52606	0.557439
GOT2	8.23726	0.509359
GP1BB	5.01982	0.44952
GPAA1	8.24088	0.562664
GPALPP1	5.02004	0.57098
GPANK1	7.71539	0.42101
GPATCH1	6.43697	0.496465
GPATCH11	5.13459	0.622643
GPATCH8	7.17142	0.454504
GPBP1	8.94773	0.48795
GPBP1L1	7.35632	0.436565
GPCPD1	7.56594	0.714592
GPD1L	7.42203	0.71748
GPHN	5.52883	0.578599
GPI	9.23218	0.634084
GPN1	7.83425	0.437213
GPN3	7.14574	0.668893
GPR108	8.49797	0.475272
GPR114	5.51834	0.506305
GPR155	5.68841	0.820622
GPR160	10.3012	0.873707
GPR75-ASB3	8.87764	0.604872
GPR89A	8.35257	0.621802
GPR89B	8.35257	0.621802
GPRASP1	4.93145	1.26922
GPRASP2	4.8676	0.721292
GPRC5D	9.58581	1.4939
GPS2	7.65444	0.461748
GPSM3	7.39154	0.567268
GPT2	5.40848	0.731487
GPX1	9.83973	0.991587
GPX3	5.02333	0.974235
GPX4	10.1009	0.526755
GPX7	7.38692	0.741469
GRAMD1C	6.13027	1.35419
GRASP	6.4615	0.465601
GRHL1	5.49408	0.643526
GRHPR	8.28538	0.45064
GRINA	6.65144	0.537107
GRK6	7.46924	0.500822
GRN	8.57739	0.781254

GRPEL1	7.45862	0.573465
GRPEL2	4.88717	0.465939
GS1-111G14.1	9.59777	0.627386
GSAP	7.35599	0.866371
GSE1	6.61994	0.410934
GSKIP	8.01231	0.72679
GSPT1	6.6717	0.507581
GSPT2	6.50546	0.869957
GSR	5.68623	0.539404
GSS	6.89959	0.434454
GSTA4	5.83982	0.965917
GSTK1	8.82502	0.468059
GSTM1	7.48663	0.677657
GSTM2	7.99051	0.781554
GSTM3	4.88351	0.77421
GSTM4	5.06572	0.511179
GSTO1	8.88609	0.706128
GSTP1	8.86821	1.21629
GSTT1	5.14382	0.721012
GSTZ1	6.14818	0.573794
GTF2A1	7.09029	0.4137
GTF2A2	6.58796	0.438684
GTF2B	8.38929	0.610487
GTF2E1	6.70224	0.733244
GTF2E2	7.4075	0.611634
GTF2F1	7.03116	0.414824
GTF2F2	6.27763	0.688369
GTF2H1	5.70253	0.5513
GTF2H2	7.13844	0.573914
GTF2H2B	6.80172	0.574766
GTF2H2C	7.90046	0.570876
GTF2H2C_2	8.54117	0.579925
GTF2H3	6.32451	0.58653
GTF2H4	5.77077	0.54044
GTF2I	8.51191	0.484721
GTF2IP1	9.48927	0.501878
GTF3A	9.09617	0.616446
GTF3C3	5.19897	0.401698
GTF3C6	9.65501	0.61702
GTPBP6	7.23557	0.488869
GTPBP8	6.07867	0.525141
GTSF1	5.62057	2.04025
GUCD1	7.79031	0.457155
GUF1	4.95136	0.605986
GUSBP11	7.17189	0.404983
GUSBP3	5.98595	0.905579
GUSBP9	6.10095	0.984453

GVINP1	5.38211	0.720251
GXYLT1	5.31626	0.619019
GYG1	9.47107	0.756148
GYPC	9.58333	0.621304
GYS1	6.74624	0.505131
GZF1	6.23694	0.447965
H1F0	6.38668	1.43295
H1FX	10.4829	0.80632
H2AFJ	6.51005	0.463076
H2AFV	8.26786	0.502976
H2AFY	6.58603	0.406809
H2AFZ	10.804	0.535063
H2BFS	10.9103	0.900827
H3F3A	11.5213	0.408598
H3F3AP4	12.3752	0.430135
H3F3B	12.2389	0.440933
HAB1	6.20905	0.402378
HACL1	7.89204	0.568725
HADH	6.46592	0.637934
HADHB	8.77555	0.434193
HAGH	8.07909	0.485505
HARS	8.39635	0.470137
HARS2	6.92263	0.492345
HAT1	8.04681	0.705807
HAUS1	6.92146	0.860028
HAUS3	8.25841	0.867565
HAUS4	6.62472	0.638197
HAUS6	5.05236	0.72422
HAUS7	5.21869	0.407488
HBA1	8.91084	2.02695
HBA2	8.91084	2.02695
HBB	8.87642	1.42587
HBD	7.54299	1.74072
HBG1	6.57174	1.32946
HBG2	6.57174	1.32946
HBP1	6.47726	0.641013
HBS1L	6.00227	0.608978
HCCS	7.168	0.539281
HCFC2	4.85094	0.437368
HCG26	5.9569	0.472899
HCK	5.07705	0.787325
HCLS1	9.95068	0.475992
HCP5	6.47497	0.953398
HCST	6.1481	0.583972
HDAC1	8.17343	0.571658
HDAC9	4.89956	0.419056
HDDC2	6.86133	0.51601

HDDC3	8.01632	0.442587
HDGF	8.51745	0.643744
HDHD1	7.02394	0.613802
HDHD2	8.02303	0.648408
HDHD3	5.95432	0.485191
HDLBP	7.30163	0.435872
HEATR1	5.5298	0.548259
HEATR2	5.94326	0.40852
HEATR3	5.40222	0.523153
HEATR5A	5.11768	0.819654
HEATR5B	5.03241	0.401009
HEBP1	6.61418	1.00249
HECA	7.28638	0.452841
HECTD1	5.72439	0.476981
HECTD3	6.1116	0.523858
HEG1	4.96549	0.554124
HEIH	8.5464	0.478775
HELB	5.33466	0.460636
HENMT1	6.53995	0.697189
HERC1	5.09139	0.406683
HERC2P2	8.81124	0.766158
HERC2P9	8.81124	0.766158
HERC5	7.98439	1.30138
HERPUD1	9.60054	0.55641
HERPUD2	7.44279	0.505601
HES4	5.42359	0.506746
HES6	4.95384	0.447959
HESX1	4.92902	0.402706
HEXB	10.4267	0.69964
HEXIM1	6.96788	0.616265
HEXIM2	5.83209	0.476811
HEY1	6.65616	1.31756
HEY2	6.82841	1.01319
HGF	6.35678	1.7803
HHIP-AS1	5.37851	0.538117
HIAT1	8.89918	0.741038
HIBADH	5.66875	0.435183
HIBCH	6.72607	0.863173
HID1	6.22699	0.512651
HIF1A	7.35222	1.94062
HIGD1A	7.73701	0.562332
HIGD2A	8.76773	0.498476
HILPDA	5.93117	0.496586
HINFP	6.43122	0.519398
HINT1	10.3556	0.496107
HINT2	9.33094	0.400207
HIPK2	7.40903	0.598871

HIPK3	5.82666	0.41623
HIST1H1C	11.502	1.15586
HIST1H1D	8.20971	1.16095
HIST1H1E	6.57827	1.09843
HIST1H1T	6.02109	0.88815
HIST1H2AB	5.30181	0.730374
HIST1H2AC	10.6501	1.27331
HIST1H2AD	7.65604	1.37718
HIST1H2AE	8.726	1.84457
HIST1H2AG	7.03915	0.867757
HIST1H2AH	7.03915	0.867757
HIST1H2AI	7.03915	0.867757
HIST1H2AJ	8.82308	0.895574
HIST1H2AK	5.96822	0.633905
HIST1H2AL	6.2559	0.88357
HIST1H2AM	7.08264	1.10364
HIST1H2BB	5.87303	1.38265
HIST1H2BC	9.05889	1.24267
HIST1H2BD	10.3821	0.946622
HIST1H2BE	9.229	1.0677
HIST1H2BF	9.15146	1.13988
HIST1H2BG	9.53608	1.27927
HIST1H2BH	9.27268	0.917503
HIST1H2BI	9.15146	1.13988
HIST1H2BJ	8.15204	1.38317
HIST1H2BK	11.6653	0.834669
HIST1H2BM	5.14442	0.779388
HIST1H2BN	6.07094	0.667996
HIST1H3A	5.71237	1.0401
HIST1H3B	5.82373	1.02366
HIST1H3C	5.90771	0.954464
HIST1H3D	6.56008	1.11502
HIST1H3E	5.24018	0.718055
HIST1H3F	5.54721	0.969432
HIST1H3G	6.37475	0.946415
HIST1H3H	6.13947	1.14745
HIST1H3I	5.48306	0.888071
HIST1H3J	6.13947	1.14745
HIST1H4A	6.69647	0.945315
HIST1H4B	6.96988	0.966413
HIST1H4C	6.96988	0.966413
HIST1H4D	6.60214	0.937764
HIST1H4E	6.98761	0.970531
HIST1H4F	6.64513	0.974561
HIST1H4H	6.94167	0.946555
HIST1H4I	6.82807	0.832972
HIST1H4J	7.08636	0.945266

HIST1H4K	6.96988	0.966413
HIST1H4L	6.54918	0.825059
HIST2H2AA3	12.222	1.09073
HIST2H2AA4	12.222	1.09073
HIST2H2BE	9.56707	1.52682
HIST2H4A	6.96988	0.966413
HIST2H4B	6.96988	0.966413
HIST3H2A	5.82188	0.670271
HIST4H4	6.96988	0.966413
HIVEP1	5.23298	0.575055
HJURP	5.24935	0.796634
HK1	7.14786	0.636119
HK2	5.00657	0.542225
HK3	5.90232	0.543023
HLA-A	12.8908	0.534297
HLA-B	12.8045	0.65387
HLA-C	12.5582	0.525635
HLA-DMA	5.85603	1.43799
HLA-DMB	6.44748	0.653153
HLA-DOB	7.67099	0.847097
HLA-DPA1	5.37861	1.11558
HLA-DQB1	5.04868	0.564433
HLA-DRA	6.74464	1.73066
HLA-DRB1	5.72816	0.796336
HLA-DRB4	4.97874	0.575399
HLA-E	10.9785	0.537808
HLA-F	9.43523	0.642635
HLA-G	10.9582	0.496605
HLA-J	10.663	0.618227
HLTF	8.03192	0.869316
HLX	5.33494	0.492727
HMBOX1	5.98726	0.409447
HMBS	7.91134	0.535464
HMCES	7.46134	0.519709
HMG20A	7.40088	0.738404
HMGA1	6.66228	0.583008
HMGA1P4	6.47826	0.418653
HMGB1	10.0872	0.535895
HMGB1P4	9.02182	0.457839
HMGB2	7.59764	0.528818
HMGB3	6.11327	0.94051
HMGB3P1	5.56341	0.58942
HMGCR	6.36282	0.69359
HMGCS1	6.20886	0.526584
HMGN1	10.5391	0.484806
HMGN2	11.4977	0.524192
HMGN3	10.6866	0.58985

HMGN4	9.56625	0.553375
HMGXB4	6.57613	0.530997
HMMR	5.7866	1.2402
HMOX1	5.63083	0.909903
HMX1	6.64443	0.465289
HN1	6.81062	0.732495
HNRNPA0	7.38855	0.42727
HNRNPA1	10.0499	0.412956
HNRNPA1P3	8.96222	0.445585
HNRNPA3	8.65961	0.503418
HNRNPAB	9.40371	0.612988
HNRNPF	7.82967	0.519034
HNRNPH1	8.34795	0.775377
HNRNPH2	8.23162	0.895592
HNRNPL	7.78058	0.493532
HNRNPLL	5.01614	0.42652
HNRNPR	8.20617	0.460442
HNRNPU	7.59104	0.507506
HNRNPU-AS1	6.56799	1.10225
HNRNPUL2-BSCL2	7.1483	0.403729
HOMER1	6.39786	1.51617
HOMER3	6.6819	0.9446
HOOK1	5.89193	1.33505
HOOK2	6.09304	0.50441
HOXA6	5.32459	0.412628
HOXC4	5.9982	0.571268
HP	5.27472	0.680036
HP1BP3	8.2566	0.407779
HPCAL1	7.27033	0.573506
HPRT1	5.44352	0.488223
HPS3	5.68045	0.50155
HPS5	6.64349	0.770036
HRAS	6.32631	0.509455
HRASLS2	5.60437	0.531564
HRSP12	5.72263	0.401919
HS2ST1	6.14176	0.807021
HSBP1	7.84324	0.554743
HSBP1L1	5.52422	0.711094
HSCB	7.56099	0.555432
HSD17B10	8.79855	0.537329
HSD17B11	8.18291	0.921376
HSD17B7	8.23781	0.425306
HSD17B8	8.66566	0.601158
HSD3B7	5.83282	0.482535
HSDL1	5.44301	0.419857
HSF2	4.86127	0.566388
HSH2D	6.9196	0.868336

HSP90AA1	11.0907	0.565778
HSP90AB1	10.7747	0.460299
HSP90B1	11.4442	0.500212
HSPA13	10.173	0.736189
HSPA14	6.16502	0.466321
HSPA1A	7.1768	1.49632
HSPA1B	7.1768	1.49632
HSPA1L	5.92029	0.545554
HSPA4	7.19709	0.499619
HSPA5	10.0601	0.597361
HSPA6	5.74776	0.802231
HSPA9	7.49743	0.409719
HSPB1	9.59166	1.19165
HSPB11	7.06695	0.551995
HSPBAP1	6.36756	0.681104
HSPBP1	6.97481	0.404692
HSPD1	7.24997	0.427541
HSPE1	8.94087	0.700597
HSPE1-MOB4	8.31259	0.760818
HSPH1	6.68389	0.628836
HTATIP2	6.28981	0.471235
HTATSF1	7.31098	0.668605
HTRA2	7.54454	0.469889
HVCN1	7.1502	0.732519
HYI	6.98101	0.592904
HYKK	5.62317	0.84784
HYLS1	6.28077	0.954688
HYOU1	10.5015	0.661829
IARS	10.7845	0.5884
IARS2	8.80057	0.693033
IBTK	5.52991	0.504859
ICAM1	6.11306	0.597898
ICAM2	10.0007	0.628785
ICAM3	10.3938	0.849756
ICAM4	5.61853	1.4767
ICE1	7.98148	0.584002
ICE2	6.00859	0.540458
ICT1	7.35861	0.59823
ID1	7.28931	1.99484
ID2	7.94142	1.48674
ID2B	5.72726	0.918443
ID3	6.26082	1.44019
IDE	7.76156	0.572247
IDH1	6.46374	0.639542
IDH2	7.80607	0.685073
IDH3A	7.11069	0.551339
IDH3B	7.27368	0.41012

IDH3G	7.84274	0.452266
IDNK	6.51389	0.475392
IDS	6.18776	0.558118
IDUA	6.27388	0.563202
IER2	11.3598	1.29504
IER3	7.55228	1.56579
IER3IP1	8.14248	0.572595
IER5	10.0425	0.955028
IFFO2	5.91297	0.690208
IFI16	9.3488	0.839221
IFI27	7.48572	2.48248
IFI27L1	6.88362	0.441082
IFI27L2	7.38412	0.451608
IFI30	8.58705	1.25108
IFI35	8.2407	0.757162
IFI44	5.44742	1.49902
IFI44L	5.4555	2.05623
IFI6	8.9132	1.75136
IFIH1	5.68825	0.672895
IFIT1	6.96787	1.92855
IFIT3	6.73901	1.79181
IFIT5	7.18247	0.971639
IFITM1	10.7112	1.54902
IFITM2	9.7317	1.25346
IFITM3	9.70693	0.927792
IFNAR1	6.76953	0.487754
IFNAR2	8.47426	0.581605
IFNGR1	7.88336	0.992693
IFNGR2	8.84657	0.468788
IFNLR1	6.46951	0.441327
IFRD1	8.14325	1.03983
IFRD2	7.91443	0.608086
IFT172	5.24175	0.445572
IFT20	8.88761	0.476193
IFT22	4.9689	0.448543
IFT46	6.50497	0.510194
IFT57	6.56133	0.564402
IFT80	5.65769	0.506371
IFT88	5.14638	0.461603
Ig alpha 1-[alpha]2m	7.80379	2.97053
IGBP1	9.219	0.545137
IGF1	7.18034	0.659504
IGF2-AS	5.38461	0.436819
IGF2R	7.08677	0.771185
IGFALS	6.24923	0.471843
IGFBP3	5.29976	0.636568
IGFBP4	5.16323	0.42586

IGFBP6	5.11061	0.431204
IGFLR1	7.70725	0.632813
IGH	6.6377	0.697453
IGHA1	6.11154	0.638791
IGHA2	6.64923	0.732158
IGHD	6.04007	0.626763
IGHD3-16	5.06673	0.458051
IGHE	5.61536	0.870388
IGHG1	5.86062	0.524207
IGHG2	7.17735	0.858872
IGHG3	6.44461	0.729891
IGHG4	6.06781	0.755329
IGHM	6.27834	0.630896
IGHV3-23	6.2917	1.00077
IGHV3-54	4.92529	0.620676
IGHV4-31	6.37216	0.684804
IGIP	5.72223	0.568285
IGJ	12.3758	1.71627
Igk	5.4913	0.485371
IGK	9.47732	1.35529
IGKC	8.72505	1.17921
IGKV1-17	8.33304	2.24123
IGKV1-37	9.09595	2.0304
IGKV1-5	5.60575	0.586006
IGKV1D-37	9.09595	2.0304
IGKV1OR-1	9.55253	1.98032
IGKV1OR10-1	9.55253	1.98032
IGKV1OR2-108	9.81061	2.06433
IGKV1OR2-2	9.55253	1.98032
IGKV2-28	7.43906	2.08511
IGKV2D-28	7.43906	2.08511
IGKV4-1	8.10755	2.18565
IGL@	5.60575	0.586006
IGLC1	7.15174	0.798385
IGLJ2	7.00311	1.29875
IGLJ3	6.94613	1.08343
IGLL3P	9.32588	1.0804
IGLL5	7.81534	1.88201
IGLV@	7.35844	1.34298
IGLV1-36	6.78241	1.93477
IGLV1-40	6.12663	0.973705
IGLV1-44	7.10634	0.853136
IGLV1-50	6.45452	1.35281
IGLV2-14	7.60196	1.98727
IGLV3-1	7.40294	1.41324
IGLV3-10	7.42729	2.01388
IGLV3-19	6.19823	0.866162

IGLV3-25	6.4054	1.3249
IGLV4-3	6.66341	0.457741
IGLV4-60	5.14495	0.631487
IGLV6-57	6.773	0.99672
IGSF22	6.20053	0.436245
IGSF8	6.32406	0.552757
IK	7.48637	0.425819
IKBKB	5.33638	0.452834
IKZF3	6.94919	0.759598
IKZF5	6.12048	0.515157
IL10RA	5.96512	1.21241
IL10RB	7.50108	0.54292
IL12A	5.2767	0.716049
IL13RA1	4.818	0.980208
IL15	5.87892	1.09253
IL15RA	6.94572	0.504388
IL16	6.70214	0.617693
IL17RA	5.88769	0.400004
IL17RB	4.89357	0.679555
IL18RAP	5.00576	0.639672
IL27RA	5.57771	0.651822
IL2RB	5.51341	0.477812
IL2RG	9.69964	1.258
IL32	4.93417	0.959813
IL4R	5.07778	0.522297
IL5RA	5.54901	0.902748
IL6	4.89597	1.11419
IL6R	6.95571	1.31262
IL6ST	7.15519	0.658686
ILF2	9.21441	0.67652
ILF3	7.6232	0.468775
ILF3-AS1	6.7622	0.808066
ILK	6.88268	0.473356
IMMP1L	6.05802	0.610598
IMMP2L	6.16817	0.669385
IMMT	8.12316	0.472168
IMP3	8.54505	0.697998
IMP4	8.91757	0.520477
IMPA1	8.02181	0.714642
IMPA2	5.79182	0.821344
IMPACT	5.50287	0.823263
IMPAD1	6.32805	0.412621
IMPDH1	6.76244	0.425017
IMPDH2	9.87489	0.856236
ING2	5.63178	0.438135
ING3	6.14488	0.407823
INO80B-WBP1	6.94489	0.406241

INO80E	6.6097	0.470132
INPP1	6.85212	0.716297
INPP4A	7.15726	0.483845
INPP5A	5.01437	0.41721
INPP5D	5.30047	0.437039
INPP5E	7.06134	0.505689
INSIG1	6.66748	0.869327
INSIG2	7.29429	0.760994
INSR	6.44416	0.586549
INTS12	7.95751	0.496863
INTS2	4.98707	0.80759
INTS5	6.74876	0.47504
INTS6	5.20108	0.463138
INTS6-AS1	4.98234	0.42143
INTS8	8.6852	0.554106
IP6K2	7.582	0.473207
IPO11-LRRC70	5.642	0.659267
IPO4	7.51982	0.506295
IPO5	6.49428	0.539995
IPO7	8.752	0.659361
IPO8	6.80337	0.43002
IQCB1	8.40187	0.613828
IQCH-AS1	6.56914	0.615379
IQGAP1	8.19553	0.611095
IQGAP2	5.87065	1.08298
IQSEC1	7.11882	0.584197
IRAK1	8.21106	0.504635
IRAK4	6.58655	0.41859
IREB2	5.3795	0.464432
IRF1	8.50921	0.91957
IRF2	6.99106	0.91082
IRF2BP2	7.86102	0.656308
IRF2BPL	7.8202	0.84619
IRF3	8.39179	0.457743
IRF4	7.38064	0.709952
IRF5	6.3009	0.450756
IRF7	7.85226	0.836556
IRF8	5.2818	0.519526
IRF9	7.05536	0.512796
IRS1	4.96641	0.603065
IRS2	5.57771	1.11621
ISCA1	8.43089	0.57776
ISCA2	7.54274	0.661762
ISCU	11.4867	0.577444
ISG15	9.3466	1.6513
ISG20	10.7562	0.764701
ISG20L2	5.96388	0.423572

ISL2	6.53352	1.46803
ISLR	5.22423	1.19039
ISOC1	7.98958	0.659158
ISOC2	6.99282	0.450217
ISY1-RAB43	6.41469	0.449713
ITCH	6.06666	0.465239
ITGA3	5.72508	0.448099
ITGA4	8.89582	0.91782
ITGA6	7.61638	1.18834
ITGA8	6.91835	1.16671
ITGAE	8.93604	0.61589
ITGAL	6.42023	0.922512
ITGAV	7.42366	0.71491
ITGB1	4.86054	0.543956
ITGB2	4.9997	0.68044
ITGB3BP	7.87725	0.742438
ITGB7	8.35823	1.3356
ITM2A	9.32649	1.40987
ITM2B	11.1489	0.619589
ITM2C	11.1861	1.12623
ITPR2	5.78765	0.452572
ITPRIP	7.20432	1.01925
IVNS1ABP	6.14646	0.577855
IZUMO4	5.97128	0.567005
JADE1	6.97898	0.546384
JADE2	5.87566	0.49965
JAG1	5.54633	0.497951
JAGN1	8.39348	0.476132
JAK1	6.7677	0.616264
JAK2	5.74027	0.660369
JAZF1	4.98674	0.855134
JDP2	5.46851	0.45532
JHDM1D-AS1	5.27969	0.447918
JKAMP	7.38166	0.580526
JMJD1C	6.41722	0.594613
JMJD7	6.43124	0.43058
JMJD8	7.25086	0.436859
JMY	8.19706	0.925918
JOSD1	7.78912	0.526965
JOSD2	6.08777	0.459722
JRKL	5.78891	0.402722
JSRP1	6.33299	0.876958
JTB	11.761	0.43239
JUN	10.6044	0.804857
JUNB	9.82046	1.02031
JUND	8.23058	0.599253
KANSL1	7.43965	0.67758

KANSL1-AS1	5.34429	0.683296
KANSL2	6.89336	0.51572
KARS	10.3632	0.467087
KAT2A	7.6153	0.502111
KAT2B	7.95002	0.732502
KAT5	7.30991	0.419395
KAT6B	6.85498	0.52001
KAT7	6.42438	0.445716
KATNA1	5.95037	0.598242
KATNBL1	5.45419	0.477214
KBTBD2	6.54151	0.510806
KBTBD3	6.01125	0.549184
KBTBD4	6.30988	0.524782
KBTBD8	7.43328	1.01991
KCNA3	7.68056	0.833291
KCNA5	5.00548	1.00697
KCNF1	6.39974	0.504146
KCNG2	4.87091	0.534209
KCNH2	5.84299	0.777287
KCNH3	5.66909	0.439214
KCNK12	6.21032	1.02525
KCNK6	6.04618	0.66567
KCNK9	5.15725	0.525487
KCNMB3	5.12348	0.679565
KCNN3	6.75646	0.858021
KCNN4	6.09885	1.01297
KCNQ1	6.67085	0.565485
KCNQ1DN	5.67134	0.426506
KCNS1	5.74594	0.406659
KCNS3	6.64307	2.24954
KCTD1	5.10101	0.407064
KCTD12	5.08046	1.46872
KCTD13	5.29451	0.447548
KCTD20	7.24711	0.565544
KCTD21	4.93319	0.525341
KCTD3	5.50867	0.94904
KCTD6	5.86676	0.520558
KCTD9	4.89313	0.840963
KDELC2	6.58957	0.83027
KDELR1	9.16515	0.603707
KDELR2	8.94225	0.634975
KDELR3	6.94295	1.30899
KDM1A	7.13397	0.408923
KDM2B	6.39225	0.578186
KDM3B	7.51963	0.568763
KDM4C	5.7907	0.504855
KDM5A	5.32848	0.412102

KDM5B	5.03536	0.444097
KDM5D	6.22018	2.37413
KDM6A	5.09336	0.605168
KDM6B	6.00663	0.408263
KDM7A	5.88806	0.739188
KEAP1	7.17217	0.627724
KHNYN	7.16885	0.463597
KIAA0040	7.48387	0.644002
KIAA0101	6.41297	1.05257
KIAA0125	6.81072	2.03222
KIAA0196	7.31021	0.55248
KIAA0232	5.51716	0.430273
KIAA0247	7.77313	0.665088
KIAA0355	6.04135	0.490954
KIAA0430	5.48881	0.482362
KIAA0513	5.34146	0.42974
KIAA0753	6.19831	0.405562
KIAA0907	7.23171	0.731997
KIAA0930	6.39345	0.502069
KIAA1024	4.97154	0.423907
KIAA1033	8.10852	0.590764
KIAA1143	8.26469	0.604278
KIAA1147	6.61324	0.580888
KIAA1191	8.81455	0.544485
KIAA1217	6.13302	0.919538
KIAA1279	6.13579	0.879132
KIAA1324L	5.19772	0.679263
KIAA1407	5.49667	0.867353
KIAA1432	6.13658	0.429146
KIAA1468	6.91936	0.602769
KIAA1551	7.04943	0.879244
KIAA1715	5.45037	0.513993
KIAA1731	5.38012	0.414808
KIAA1804	5.03583	0.831991
KIF12	5.92496	0.436583
KIF13B	6.59235	0.57972
KIF18B	5.37295	0.782922
KIF21A	5.57404	0.713442
KIF21B	6.18012	1.01124
KIF22	5.7866	0.507372
KIF2A	6.46255	0.68497
KIF2C	5.38524	0.646881
KIF3A	5.07968	0.640879
KIF3B	6.95741	0.462691
KIF4A	4.92458	0.895394
KIFAP3	7.25254	0.74643
KIFC1	6.53559	0.461665

KIFC2	6.39625	0.414861
KISS1R	5.70486	1.52352
KIT	6.63767	2.09189
KIZ	6.27996	0.45587
KLC2	6.40415	0.491719
KLF10	9.45212	1.11781
KLF11	5.27546	0.821626
KLF2	8.86617	0.900146
KLF3	6.18402	0.833078
KLF4	6.4399	1.86899
KLF5	4.99707	0.983805
KLF6	9.87726	1.32231
KLF7	5.06851	0.741561
KLF9	5.6331	0.745817
KLHDC2	8.86052	0.51115
KLHDC3	7.01126	0.437978
KLHDC7B	5.67281	0.566625
KLHDC8A	5.08017	0.432525
KLHDC8B	5.36556	0.402293
KLHDC9	6.50699	0.700562
KLHL12	6.39939	0.543365
KLHL14	4.83695	0.969106
KLHL15	6.98393	0.758958
KLHL2	5.55162	0.710861
KLHL20	5.75338	0.463751
KLHL21	6.00974	0.592916
KLHL24	6.70442	0.631827
KLHL26	5.49659	0.425844
KLHL28	5.98647	0.564201
KLHL42	6.04864	0.665989
KLHL5	6.42834	1.00288
KLHL6	7.76119	0.799883
KLHL7	5.61665	0.622801
KLHL8	5.58566	0.523063
KMO	5.31436	0.954393
KMT2A	5.92624	0.515913
KMT2C	6.18767	0.437912
KMT2E	8.14581	0.60646
KMT2E-AS1	6.39974	0.408221
KNSTRN	6.53715	0.779475
KNTC1	7.57051	0.857192
KPNA1	5.97364	0.454479
KPNA2	8.74028	0.814177
KPNA3	6.58218	0.667501
KPNA4	6.17403	0.440915
KPNA5	5.40075	0.624324
KPNB1	7.54991	0.543239

KPTN	6.39007	0.430487
KRAS	7.0457	0.427597
KRBOX4	4.93568	0.463649
KRCC1	7.24057	0.773784
KRI1	6.37557	0.407658
KRIT1	6.36273	0.4313
KRT10	8.9978	0.633674
KRT18	5.87096	0.975865
KRT81	5.54405	0.409574
KRT8P11	5.57571	0.405663
KRTCAP3	5.05221	0.521347
KTI12	5.94727	0.666274
KTN1	9.34674	0.592688
L3HYPDH	5.41685	0.653772
LACTB	5.79187	0.698627
LACTB2	7.16871	0.985704
LAG3	5.04216	0.955475
LAGE3	7.93708	0.663497
LAIR1	5.44307	0.538571
LAIR2	4.93093	0.416265
LAMA5	6.14243	0.703654
LAMA5-AS1	6.09786	0.593131
LAMC1	7.97442	0.892915
LAMP1	7.78373	0.580461
LAMP2	7.7871	0.649214
LAMP3	8.16218	1.48885
LAMP5	9.43903	2.82095
LAMTOR1	9.01813	0.424427
LAMTOR2	8.11039	0.650338
LAMTOR3	6.49019	0.615212
LAMTOR4	8.30165	0.453683
LAMTOR5	9.06021	0.427657
LANCL1	7.203	0.454537
LAP3	9.68917	0.702612
LAPTM4A	10.7537	0.47704
LAPTM4B	6.41461	1.84536
LAPTM5	8.52555	2.30964
LARP1B	6.17966	0.527547
LARP4	6.55792	0.537053
LARS	8.06102	0.48417
LARS2	5.10762	0.549527
LASP1	8.08707	0.567707
LAT2	6.17009	0.664211
LATS2	5.67821	0.64502
LAX1	9.42415	0.985531
LBH	4.87676	0.752372
LBR	8.02028	0.958068

LCLAT1	6.48359	0.766049
LCMT1	7.91959	0.432767
LCMT2	5.60552	0.481849
LCN2	5.89219	1.16197
LCOR	6.97626	0.742393
LCORL	7.05236	0.697109
LCP1	7.22849	1.54079
LDHA	11.4694	0.676154
LDHB	11.6603	0.579678
LDLR	5.49584	0.567432
LDLRAD4	5.14827	0.494138
LDLRAP1	7.31959	0.563727
LDOC1	5.03477	1.02587
LDOC1L	5.34326	0.865915
LEAP2	5.79986	0.493289
LEMD2	5.4839	0.432728
LEMD3	9.14723	0.477576
LENG8	6.55789	0.851352
LEO1	5.93789	0.793194
LEPREL1	5.34445	0.615376
LEPREL2	5.52847	0.446652
LEPREL4	5.22336	0.475244
LEPROTL1	6.59217	0.54264
LETMD1	8.07775	0.631221
LGALS1	10.3177	0.993801
LGALS3	6.32764	0.631742
LGALS3BP	6.28366	1.23984
LGALS8	5.50601	0.719271
LGALS9	6.50483	0.539805
LGALSL	6.22397	0.637469
LGMN	6.5571	1.27724
LHFPL2	5.19302	0.680763
LHPP	5.85693	0.577793
LHX4-AS1	5.05361	0.451614
LIAS	5.35954	0.62564
LIG1	7.00165	0.465791
LIG4	4.94397	0.535721
LILRA2	4.82586	0.432002
LILRA3	4.91459	0.494077
LILRA4	5.44414	0.439982
LILRB1	6.86034	0.805401
LILRB3	6.87826	0.421199
LIMD1	5.86331	0.407773
LIMD2	6.12597	0.565375
LIME1	7.50897	0.527417
LIMS1	5.59066	0.419771
LIMS2	6.10144	0.458135

LIMS3	6.02113	0.556267
LIMS3L	6.62914	0.581755
LIN52	6.75092	0.593101
LIN7C	7.93285	0.632268
LINC-PINT	6.97796	0.568107
LINC00094	5.65216	0.692029
LINC00115	4.84862	0.590807
LINC00116	6.13525	0.794362
LINC00152	7.6673	0.865069
LINC00263	5.00326	0.450312
LINC00294	6.58973	0.607736
LINC00324	6.317	0.877677
LINC00339	6.58119	0.824669
LINC00341	7.80041	1.10294
LINC00342	7.7965	0.575247
LINC00467	7.06181	0.842393
LINC00482	6.20454	0.411697
LINC00493	10.0174	0.567907
LINC00494	5.85823	0.720446
LINC00520	7.21429	0.433789
LINC00525	6.26912	1.74225
LINC00526	6.15628	0.52586
LINC00528	6.00701	0.739833
LINC00537	7.05613	0.647231
LINC00582	8.1534	1.1079
LINC00621	8.43651	0.687507
LINC00623	8.71927	0.775727
LINC00657	9.05782	0.724852
LINC00667	5.95671	0.422715
LINC00685	5.04789	0.422375
LINC00847	6.91205	0.62875
LINC00849	5.75042	0.707323
LINC00869	5.71171	0.4832
LINC00886	5.45627	0.467004
LINC00888	8.06206	0.634772
LINC00893	4.95192	0.638509
LINC00909	6.75966	0.749965
LINC00936	7.66494	1.15686
LINC00938	5.78729	0.504928
LINC00959	5.85803	0.663723
LINC00969	6.23618	0.511644
LINC00984	6.69608	0.683963
LINC00992	5.21743	1.05035
LINC00998	6.545	0.595638
LINC01000	6.9544	0.532305
LINC01001	5.79212	0.493729
LINC01002	7.0353	0.745539

LINC01003	6.82606	0.95969
LINC01023	5.70528	0.555372
LINC01061	5.1162	0.536895
LINC01125	5.24944	0.455255
LINC01126	4.91492	0.474082
LINC01296	5.04926	0.885677
LINC01410	6.44538	0.965768
LINC01420	7.11266	0.545263
LINS	5.9365	0.578272
LIPT1	6.60126	0.780035
LITAF	6.96651	0.477661
LIX1L	6.02557	0.607971
LL22NC03-N14H11.1	6.43517	0.939656
LL22NC03-N64E9.1	6.10777	2.2316
LLGL2	5.5237	0.421699
LLPH	5.3214	0.415432
LMAN1	9.06362	0.732724
LMAN2L	6.70809	0.449901
LMBRD1	6.6869	0.487961
LMBRD2	5.47845	0.443085
LMF1	5.55399	0.431401
LMNA	6.7582	0.520746
LMNB1	5.82257	0.857043
LMNB2	6.44667	0.553166
LMO4	6.07236	0.544821
LNP1	7.11889	0.606521
LNPEP	5.71754	0.445482
LNX2	5.61945	0.431527
LOC100049716	4.8613	0.403239
LOC100093631	9.48927	0.501878
LOC100127972	4.85428	0.728712
LOC100128325	5.32126	0.532742
LOC100129034	5.71613	0.423778
LOC100129518	5.69226	0.632845
LOC100129550	5.46908	0.644973
LOC100129924	6.18987	0.416191
LOC100130093	7.65759	0.630506
LOC100130429	6.07891	0.556013
LOC100130872	5.66894	0.431773
LOC100131043	6.85233	0.82483
LOC100131541	5.05148	0.941227
LOC100131564	7.44178	0.446162
LOC100132062	7.0353	0.745539
LOC100132167	5.24533	0.419349
LOC100132352	6.15596	0.623982
LOC100132354	5.56359	0.446506
LOC100132705	5.68224	0.426046

LOC100133130	6.49641	0.451534
LOC100133182	7.0353	0.745539
LOC100133315	5.21219	0.697266
LOC100133331	7.0353	0.745539
LOC100134091	5.90969	0.671481
LOC100134937	4.80578	0.480201
LOC100147773	5.11507	0.676982
LOC100190986	7.82975	0.813682
LOC100233156	6.78521	0.531095
LOC100270804	6.46871	0.65649
LOC100272216	7.85894	0.898312
LOC100287497	5.81775	0.89164
LOC100287852	8.34869	0.417132
LOC100288152	6.05164	0.612298
LOC100288974	4.9008	0.421455
LOC100289019	6.16368	0.608984
LOC100289092	5.32209	0.436263
LOC100289097	6.49167	0.465221
LOC100289098	7.22635	0.607739
LOC100289333	6.78007	0.479168
LOC100289361	6.28101	0.597809
LOC100310756	6.30116	0.502823
LOC100505478	5.78225	0.403921
LOC100505650	5.18398	0.788912
LOC100505715	6.22929	0.722843
LOC100505812	5.22656	0.684059
LOC100505915	5.63107	0.472108
LOC100506076	7.78973	0.820894
LOC100506098	7.07093	1.01641
LOC100506100	6.52063	0.683971
LOC100506123	7.78973	0.820894
LOC100506248	11.4907	0.452525
LOC100506302	6.01842	0.639908
LOC100506325	5.3958	0.443353
LOC100506473	6.10602	0.526361
LOC100506548	6.25549	0.77898
LOC100506797	5.00405	0.447027
LOC100506844	5.06457	0.478474
LOC100506922	5.9893	0.874538
LOC100506990	5.36332	0.613468
LOC100507006	7.34682	0.794665
LOC100507022	4.98566	0.523336
LOC100507217	5.8734	0.558037
LOC100507431	4.83813	0.660165
LOC100507487	4.96049	0.540344
LOC100507535	7.74712	0.762077
LOC100507577	6.50883	0.484843

LOC100507670	5.6452	0.42755
LOC100509445	7.89465	1.80851
LOC100653061	5.22682	0.433652
LOC100653149	5.21019	0.664532
LOC100653247	9.2656	0.834441
LOC100996332	6.22167	0.40505
LOC100996412	6.71573	1.15393
LOC100996425	5.83723	0.407257
LOC100996517	7.83898	0.64251
LOC100996579	5.25191	0.648022
LOC100996643	5.39179	0.423138
LOC100996668	5.43129	0.465939
LOC100996696	10.6601	0.707292
LOC100996717	7.3831	1.26053
LOC100996724	5.77941	0.459395
LOC100996732	5.3104	0.591297
LOC100996735	5.3104	0.591297
LOC100996740	8.27248	1.06105
LOC100996741	5.73898	0.834982
LOC100996747	11.8815	0.696551
LOC100996756	8.53662	1.38235
LOC100996761	5.85355	0.521299
LOC100996763	7.3831	1.26053
LOC100996792	6.7227	0.585743
LOC100996809	5.32188	0.754632
LOC101059949	5.84505	1.3368
LOC101060275	7.92019	0.693349
LOC101060321	7.00544	0.473434
LOC101060351	7.00544	0.473434
LOC101060363	10.4416	0.46016
LOC101060373	10.0789	0.712953
LOC101060376	7.00544	0.473434
LOC101060386	6.20894	0.650432
LOC101060389	7.00544	0.473434
LOC101060399	7.91905	0.577176
LOC101060405	6.63413	0.508255
LOC101060443	4.97523	0.47712
LOC101060510	4.98706	0.511078
LOC101060578	8.92557	0.638545
LOC101060596	5.98226	0.909193
LOC101060604	5.36874	0.4194
LOC101060632	5.84505	1.3368
LOC101060691	4.92277	0.411168
LOC101060817	7.74551	0.746718
LOC101060835	5.2343	0.678956
LOC101243545	8.45735	0.608232
LOC101926963	5.84288	0.491742

LOC101927144	7.69582	0.848563
LOC101927178	6.07812	0.581604
LOC101927180	9.38528	0.449613
LOC101927184	4.96049	0.540344
LOC101927204	5.17955	0.560984
LOC101927266	5.76082	0.40971
LOC101927268	4.92715	0.740188
LOC101927330	5.51473	0.668761
LOC101927345	5.84505	1.3368
LOC101927420	5.43818	0.427327
LOC101927451	6.20333	0.895913
LOC101927507	5.26452	0.430629
LOC101927705	4.97225	0.630744
LOC101927733	10.4668	0.510542
LOC101927811	6.44125	1.16419
LOC101927933	5.4352	0.442148
LOC101927974	5.34812	0.464343
LOC101928000	7.31069	0.584115
LOC101928061	9.82688	0.446511
LOC101928099	4.89199	0.404346
LOC101928102	5.56787	1.12245
LOC101928111	4.88289	0.419556
LOC101928143	5.31958	0.510468
LOC101928152	5.41386	1.31298
LOC101928189	8.87125	0.666845
LOC101928195	5.39179	0.423138
LOC101928198	4.93702	0.888988
LOC101928230	5.87667	0.475959
LOC101928361	5.23271	0.633789
LOC101928371	5.23366	0.41478
LOC101928403	5.19235	0.499578
LOC101928433	5.92026	0.766221
LOC101928589	6.14292	0.585681
LOC101928615	8.63912	0.655962
LOC101928625	6.31091	0.803783
LOC101928647	5.12669	0.425383
LOC101928676	8.1938	0.545548
LOC101928789	7.14699	0.723462
LOC101928806	5.348	0.547929
LOC101928830	10.0062	0.579837
LOC101928852	4.98599	0.4173
LOC101928879	6.77418	0.520579
LOC101928927	7.70465	0.577097
LOC101929072	5.93207	0.525868
LOC101929112	5.89333	0.536711
LOC101929115	5.37135	0.601436
LOC101929141	6.32497	1.74973

LOC101929177	6.16747	0.434866
LOC101929219	5.56342	0.616479
LOC101929243	6.77805	0.807529
LOC101929272	7.82886	1.81663
LOC101929356	7.64565	0.479051
LOC101929368	7.03946	0.499885
LOC101929479	6.58855	0.404781
LOC101929612	7.94594	0.674098
LOC101929655	6.22307	0.400978
LOC101929740	5.72669	0.637281
LOC101929747	4.82353	0.448461
LOC101929759	5.59935	0.96734
LOC101929787	5.99214	0.817109
LOC101929792	5.85355	0.521299
LOC101929796	7.3831	1.26053
LOC101929819	5.79212	0.493729
LOC101929841	6.39493	0.484498
LOC101929855	5.73491	0.866852
LOC101929876	11.8815	0.696551
LOC101929880	6.90608	0.707805
LOC101929886	6.48709	0.795455
LOC101929889	7.12343	0.54327
LOC101929897	6.173	0.409908
LOC101929910	6.56894	0.522603
LOC101930026	6.4503	0.414484
LOC101930052	6.08891	0.544274
LOC101930059	6.13759	0.757544
LOC101930100	4.85344	0.508494
LOC101930107	7.22803	0.546222
LOC101930112	5.93723	0.419994
LOC101930115	6.05901	0.48864
LOC101930123	6.57483	0.55091
LOC101930154	7.712	0.492907
LOC101930278	7.07294	0.5241
LOC101930306	5.22741	0.413696
LOC101930324	8.83194	0.507183
LOC101930388	5.31958	0.510468
LOC101930415	7.62892	0.957947
LOC101930416	5.85355	0.521299
LOC101930489	7.6673	0.865069
LOC101930531	6.10648	0.705418
LOC101930583	7.80165	0.680691
LOC101930591	7.86529	0.416836
LOC101930595	5.00962	0.435406
LOC102288414	8.39657	0.518503
LOC102606465	4.98019	0.591681
LOC102723678	5.42737	0.647017

LOC102723692	5.06525	0.493147
LOC102723709	5.42737	0.647017
LOC102723773	5.98226	0.909193
LOC102723779	6.02613	0.80529
LOC102723847	5.1887	0.40911
LOC102723859	7.00544	0.473434
LOC102723891	9.30384	1.34837
LOC102723897	7.75284	0.529578
LOC102724017	6.62201	0.712232
LOC102724093	6.62875	0.838701
LOC102724112	5.83966	0.593344
LOC102724197	5.68224	0.426046
LOC102724229	5.52328	0.998422
LOC102724250	6.23104	0.495644
LOC102724356	7.21508	0.920961
LOC102724362	6.07532	0.642253
LOC102724364	7.89724	0.609519
LOC102724517	6.09991	1.14299
LOC102724532	6.72759	0.565457
LOC102724562	6.08891	0.544274
LOC102724614	8.09703	0.651593
LOC102724782	5.45165	0.409442
LOC102724788	5.85032	0.491324
LOC102724814	5.69304	0.459252
LOC102724851	5.33103	0.740908
LOC102724862	7.00544	0.473434
LOC102724870	6.52083	0.413677
LOC102724884	8.51587	0.725877
LOC102724923	5.90969	0.671481
LOC102724967	6.24984	0.442499
LOC102724984	8.38483	0.422595
LOC102724985	6.59686	0.486068
LOC102724993	9.27647	0.529879
LOC102725051	9.30384	1.34837
LOC102725125	7.53614	0.945283
LOC102725127	5.21019	0.664532
LOC102725188	9.24893	1.0002
LOC102725198	6.3396	0.623429
LOC102725426	8.90517	2.13372
LOC102725526	5.654	0.97376
LOC103091866	8.73012	1.04582
LOC113230	5.67116	0.435449
LOC150776	6.54243	0.478868
LOC153546	6.99462	0.586668
LOC153684	6.77651	0.724473
LOC154761	5.96884	0.793888
LOC157562	5.28608	0.448097

LOC158960	6.97429	0.505232
LOC202025	4.87934	0.614477
LOC220729	7.05538	1.07369
LOC254896	5.5243	0.45626
LOC283070	6.84711	0.789398
LOC283278	5.03278	0.714926
LOC284454	7.08222	0.689553
LOC284889	4.99422	0.406508
LOC285074	7.34927	0.542157
LOC285812	5.45253	1.06767
LOC285835	5.93207	0.525868
LOC286052	5.73314	0.977323
LOC286367	5.12671	0.662599
LOC286437	6.1222	0.51839
LOC374443	4.83303	0.69788
LOC389765	5.08458	0.662007
LOC389834	5.41382	0.803776
LOC439994	7.1412	0.636979
LOC440354	7.47835	0.753797
LOC440434	7.51473	0.424287
LOC441155	7.74884	0.553931
LOC441259	5.91933	0.459188
LOC613037	6.4516	0.573535
LOC642236	5.03655	0.505421
LOC642361	7.1412	0.636979
LOC642423	6.58855	0.404781
LOC642846	6.73377	0.666891
LOC643072	5.21434	0.641686
LOC643733	5.06801	0.848467
LOC644656	6.7043	0.863884
LOC645166	4.85361	0.440867
LOC646214	5.62885	0.517241
LOC646743	4.92248	0.429384
LOC646762	6.22241	0.829825
LOC727751	6.58855	0.404781
LOC727820	7.62939	0.79611
LOC728026	11.4907	0.452525
LOC728392	6.82415	0.586136
LOC728554	7.14163	0.724943
LOC728613	5.67752	0.49628
LOC728715	7.89465	1.80851
LOC728730	5.13457	0.487995
LOC728903	6.50366	0.743343
LOC729218	5.1162	0.536895
LOC729737	7.0353	0.745539
LOC729887	5.26646	0.468839
LOC730098	5.68082	0.405114

LOC730101	7.27583	1.36674
LOC732360	6.5954	0.635298
LOC93622	5.60403	0.689477
LOH12CR1	5.42636	0.451976
LONP1	7.86948	0.562099
LONP2	6.50883	0.484843
LONRF1	5.8165	0.674742
LPCAT1	7.00887	0.560273
LPIN1	7.60749	0.713862
LPIN2	4.96623	0.4401
LPPR3	5.93244	0.456336
LPXN	7.45536	0.677732
LRBA	6.54923	0.636869
LRCH1	4.84082	0.47024
LRG1	5.34225	0.453569
LRIF1	6.95259	0.967721
LRMP	8.44339	1.19498
LRP10	7.28221	0.41058
LRP11	4.90937	1.14015
LRP6	5.21895	0.428335
LRP8	5.33857	0.761076
LRPPRC	7.00692	0.481199
LRR1	6.06857	0.528668
LRRC16A	6.74728	0.562555
LRRC16B	6.23976	0.467583
LRRC25	5.72108	0.560226
LRRC37B	6.39493	0.484498
LRRC40	7.2744	0.413627
LRRC42	5.61057	0.460114
LRRC47	8.57964	0.449762
LRRC58	6.08503	0.520667
LRRC69	4.92825	0.578233
LRRC8C	5.4352	0.442148
LRRC8D	8.30358	0.614941
LRRD1	6.55335	0.442508
LRRFIP1	6.37189	0.522419
LRRK1	6.15557	0.706326
LRRK2	5.07709	1.38563
LRRN1	6.92136	1.19824
LRRN2	5.03395	0.427669
LRRTM2	4.95703	0.726153
LRTM2	5.608	0.557796
LRWD1	7.11589	0.506725
LSAMP	5.48249	1.09246
LSG1	6.75649	0.494184
LSM1	8.97221	0.46591
LSM10	8.47447	0.465412

LSM12	6.99128	0.488363
LSM14A	8.00141	0.403233
LSM2	7.11306	0.664985
LSM4	6.95675	0.431412
LSM5	7.04451	0.709616
LSM6	7.53881	0.563738
LSM7	9.96135	0.626409
LSM8	5.91475	0.48753
LSP1	7.93698	1.17814
LSR	7.70557	0.877323
LST1	5.77748	0.70217
LTA4H	10.0062	0.579837
LTB	5.38903	0.446427
LTB4R2	5.08573	0.459929
LTBP1	6.85697	0.811486
LTF	6.19979	1.67365
LTK	5.47019	0.604213
LTN1	6.17212	0.546819
LTV1	6.253	0.539376
LUC7L2	7.55318	0.434984
LUC7L3	7.78791	0.530514
LUZP6	9.85691	0.5203
LXN	6.50519	0.975961
LY6E	7.54872	1.20776
LY75	6.61247	0.803525
LY75-CD302	6.05847	1.06541
LY86	7.04044	1.342
LY9	8.2133	0.975694
LY96	9.6142	1.16301
LYAR	6.88252	0.585382
LYL1	6.5712	0.457967
LYN	7.53307	0.640023
LYPD6B	6.29982	1.42556
LYPLA1	9.23994	0.641435
LYPLAL1	7.06342	0.833558
LYRM1	8.65635	0.548534
LYRM4	6.8848	0.606366
LYRM5	8.79912	0.65631
LYRM7	6.21829	0.637295
LYSMD1	5.00746	0.53934
LYSMD2	6.86198	1.72327
LYSMD3	8.12143	0.847073
LYSMD4	5.80303	0.548365
LYZ	6.60601	1.92607
LZIC	6.42938	0.472962
LZTFL1	5.90511	0.530386
LZTS3	5.4394	0.518643

M6PR	8.07451	0.54505
MAD2L1	5.29313	0.937023
MAD2L1BP	6.57011	0.464493
MAD2L2	7.01334	0.471327
MAEA	8.1938	0.545548
MAF1	7.34165	0.453101
MAFB	5.9537	1.28146
MAFF	7.46077	1.32682
MAFIP	7.04277	0.694091
MAGEA12	4.88299	1.46114
MAGEA3	6.48994	2.4215
MAGEA6	6.43185	2.38272
MAGEA9	5.0042	0.658862
MAGEA9B	5.0042	0.658862
MAGEC1	7.10097	1.82016
MAGEC2	4.90349	0.568722
MAGED1	10.2667	0.695488
MAGED2	7.55597	0.534802
MAGED4	5.37218	1.05267
MAGED4B	5.37218	1.05267
MAGEE1	5.62739	0.451632
MAGEF1	6.49616	0.687763
MAGEH1	8.21239	0.725046
MAGI2	5.03272	1.29072
MAGOH	6.96994	0.561932
MAGOHB	5.84245	0.40646
MAGT1	9.15638	0.565629
MAK16	6.80421	0.710708
MAL	4.9689	1.41301
MALAT1	10.8176	0.572332
MALT1	6.87819	0.850678
MAMDC4	5.46109	0.440487
MAML1	6.88791	0.619787
MAML2	5.65881	0.986173
MAN1A1	9.69397	1.10433
MAN1A2	6.11889	0.419784
MAN1B1	7.39124	0.479472
MAN1C1	5.23385	0.941244
MAN2A1	9.74437	0.738348
MAN2A2	6.20217	0.454271
MAN2B1	8.57256	0.641006
MAN2B2	7.09924	0.617735
MANBA	6.75573	0.525467
MANBAL	7.6673	0.50816
MANEA	7.75743	1.06221
MANEAL	6.51214	0.63191
MANF	10.5939	0.739834

MANSC1	6.2717	1.32486
MAP1LC3B	9.07924	0.693373
MAP1S	5.83189	0.418141
MAP2	5.09805	1.06212
MAP2K1	8.22583	0.53569
MAP2K3	6.7227	0.585743
MAP2K4	6.44433	0.492925
MAP2K6	4.97072	0.529901
MAP3K1	7.99441	0.76824
MAP3K13	5.88382	0.448087
MAP3K14	5.53317	0.876468
MAP3K4	7.11817	0.538452
MAP3K5	7.51523	0.902998
MAP3K7	6.60174	0.586352
MAP3K8	5.18334	1.18148
MAP4K1	6.43638	0.414145
MAP4K3	6.4692	0.835338
MAP4K5	5.76848	0.543831
MAP7	5.00584	0.613024
MAP7D1	6.4515	0.564489
MAPK1	5.81841	0.40579
MAPK13	6.73651	0.793615
MAPK1IP1L	7.7815	0.440233
MAPK8	5.61335	0.446062
MAPKAPK2	7.72452	0.729992
MAPKAPK5	6.0638	0.514874
MAPRE1	8.83679	0.434967
1-Mar	6.09653	0.592273
2-Mar	5.9237	0.826797
2-Mar	6.40276	0.502431
3-Mar	5.07426	0.475564
5-Mar	5.8599	0.469769
MARCKS	7.17726	1.65011
MARCKSL1	7.43861	1.11323
MARS	8.27024	0.479879
MARS2	5.32813	0.729438
MARVELD2	4.90312	0.585908
MAST1	6.03682	0.576163
MAST3	6.48966	0.446259
MAST4	5.24287	0.514461
MASTL	5.15585	0.698848
MAT1A	5.61727	0.400797
MAT2A	7.69056	0.733992
MAT2B	7.20341	0.435777
MATN1-AS1	4.88635	0.643223
MAX	6.69201	0.417507
MAZ	6.46415	0.422998

MB21D2	7.01643	1.09182
MBD4	8.35798	0.588187
MBIP	5.25737	0.549412
MBL1P	4.9008	0.421455
MBNL1	7.38957	0.613476
MBNL2	7.14101	0.760053
MBNL3	5.52645	0.677487
MBTD1	5.52891	0.457331
MBTPS1	7.54901	0.435596
MC4R	6.22461	1.83765
MCAM	5.42334	0.41859
MCAT	6.47352	0.481449
MCC	7.60827	1.02758
MCCC1	7.60886	0.502256
MCEE	8.27825	0.495193
MCEMP1	5.4839	0.742839
MCF2L-AS1	5.04487	0.711493
MCFD2	6.59372	0.456606
MCL1	8.86932	0.570584
MCM2	7.01286	1.04487
MCM3	7.07921	0.826195
MCM4	5.30397	0.540845
MCM5	6.53083	0.774854
MCM6	5.8057	0.620693
MCM7	7.38812	0.733098
MCOLN1	6.90248	0.472291
MCPH1	4.98575	0.456674
MCRS1	6.7513	0.430958
MCTP2	6.72964	0.725428
MCU	6.43067	0.444068
MCUR1	7.55937	0.678051
MDC1	5.86471	0.402013
MDFIC	7.87337	0.688712
MDH1	9.96169	0.656278
MDH2	7.96933	0.469301
MDK	7.36076	1.17583
MDM1	5.0536	0.526281
MDM2	5.86033	0.549198
MDM4	6.887	0.593826
MDN1	5.15272	0.418319
MDP1	7.12626	0.551959
ME2	8.17049	0.910161
MEA1	8.67728	0.449016
MECP2	5.91581	0.41431
MECR	6.55838	0.413785
MED1	6.69633	0.557274
MED10	7.19979	0.40331

MED11	7.06128	0.516262
MED13	6.65097	0.414084
MED13L	7.34143	0.601478
MED17	5.87488	0.5099
MED19	7.59429	0.437372
MED20	5.65667	0.468739
MED21	6.31091	0.803783
MED22	5.89292	0.425599
MED23	5.90322	0.41951
MED26	6.08142	0.406301
MED29	6.25287	0.510496
MED30	5.22449	0.459113
MED31	5.8858	0.490848
MED4	5.31065	0.575663
MED6	6.68158	0.611435
MED7	6.47324	0.467979
MED8	6.95691	0.482527
MEF2A	6.49562	0.616372
MEF2B	5.72371	0.863846
MEF2BNB-MEF2B	5.72371	0.863846
MEF2C	9.46416	0.767027
MEGF9	4.81155	0.626959
MEI1	7.44673	0.569477
MEIS2	5.63998	2.46003
MEIS3P1	6.10905	0.626216
MELK	6.56465	1.14163
MEMO1	7.19772	0.514973
MEN1	6.96101	0.51887
MEPCE	7.88741	0.489649
MERTK	5.7362	0.705544
MESDC1	6.65943	0.517491
MEST	5.90177	1.35548
MET	5.15941	0.477844
METAP1	7.62849	0.478365
METAP2	7.36716	0.527933
METRN	5.81738	0.423483
METRNL	5.42506	1.15933
METTL1	6.83212	0.509357
METTL13	6.65741	0.61308
METTL18	6.55007	0.918406
METTL21A	5.04539	0.548184
METTL21B	5.86656	0.707528
METTL23	9.21654	0.470651
METTL25	6.84638	0.534211
METTL2A	6.64138	0.625457
METTL2B	5.7484	0.473443
METTL3	7.09059	0.464576

METTL4	5.16147	0.575642
METTL5	6.84709	0.422282
METTL8	5.97136	0.655308
METTL9	7.1877	0.420147
MEX3C	5.71332	0.45183
MFAP1	7.48247	0.985374
MFAP2	5.54037	0.559039
MFAP3	5.28666	0.517581
MFAP3L	4.93702	0.888988
MFAP4	5.04431	0.446182
MFF	8.03062	0.48749
MFGE8	6.0705	0.446556
MFN1	7.0009	0.592594
MFNG	6.45917	0.709012
MFSD1	9.55183	0.628677
MFSD10	6.44407	0.466277
MFSD3	5.89624	0.511489
MFSD5	6.63496	0.580155
MFSD6	6.85044	0.868291
MFSD8	5.58394	0.557562
MGA	5.17583	0.488944
MGAT1	8.33965	0.541793
MGAT2	8.37017	0.514772
MGAT4B	6.70952	0.444384
MGAT5	5.04201	0.50089
MGC12488	5.41215	0.688602
MGC50722	5.16465	0.423374
MGEA5	7.19428	0.527852
MGLL	5.93346	1.07781
MGME1	6.85099	0.870157
MGMT	6.84098	0.696023
MGRN1	6.80332	0.428042
MGST2	6.69629	0.885328
MIA-RAB4B	6.67476	0.486806
MIA3	7.82101	0.587264
MIAT	6.4238	1.28893
MIATNB	5.5636	0.506565
MIB1	6.97582	0.794006
MICA	6.42473	0.419511
MICAL1	6.60816	0.58965
MICB	7.23388	0.515408
MICU1	6.86156	0.424815
MICU2	7.69526	0.747832
MID1IP1	5.70777	1.06092
MID2	5.73933	0.603619
MIEF1	7.21591	0.56019
MIER1	5.26415	0.424768

MIER3	5.05232	0.534069
MIF	11.609	0.591396
MIF4GD	6.51721	0.52916
MINA	7.10338	0.564566
MINPP1	6.69414	0.895202
MIOS	6.45172	0.443131
MIR101-1	5.43338	0.692453
MIR1182	5.88319	0.801767
MIR1199	5.67116	0.435449
MIR1234	7.46304	0.467638
MIR1236	8.93863	0.518316
MIR1244-1	11.4907	0.452525
MIR1244-2	11.4907	0.452525
MIR1248	10.3277	0.543255
MIR1292	7.98352	0.525796
MIR1304	8.83222	0.80499
MIR142	6.62001	0.503136
MIR155	6.01022	2.37587
MIR155HG	6.01022	2.37587
MIR181A2HG	4.84621	0.714676
MIR186	5.54974	0.654487
MIR21	7.99565	1.14785
MIR22	7.66417	0.9239
MIR22HG	7.66417	0.9239
MIR23A	7.08222	0.689553
MIR24-2	7.08222	0.689553
MIR29B2	8.70778	0.9096
MIR29C	8.70778	0.9096
MIR3190	6.83255	0.438232
MIR3191	6.83255	0.438232
MIR3620	8.91579	0.405411
MIR3652	12.5726	0.491153
MIR3656	6.68084	0.522342
MIR3658	6.66102	0.622971
MIR3671	5.43338	0.692453
MIR3916	5.29917	0.98394
MIR3917	6.61334	0.529908
MIR4435-1HG	5.4429	0.414207
MIR4453	6.97683	0.972807
MIR4467	7.11589	0.506725
MIR4640	5.60781	0.473182
MIR4647	7.38075	0.49095
MIR4657	6.7739	0.523664
MIR4680	8.4276	1.00784
MIR4707	6.62472	0.638197
MIR4738	12.1026	0.767475
MIR4784	9.37121	0.573565

MIR5193	7.42395	0.634991
MIR600	5.65054	0.400128
MIR600HG	5.65054	0.400128
MIR612	8.61682	0.706594
MIR6125	6.30357	0.42135
MIR6132	6.36975	0.442949
MIR631	5.6485	0.443436
MIR636	10.1927	0.425978
MIR6513	6.90224	0.46861
MIR6516	5.28603	0.687087
MIR664B	8.43967	0.685712
MIR6732	7.27017	1.0192
MIR6733	7.74088	0.6178
MIR6734	7.39797	0.427483
MIR6741	7.57459	0.647642
MIR6748	8.64465	0.436963
MIR6751	8.89844	0.542338
MIR6756	5.46686	0.412881
MIR6758	8.27024	0.479879
MIR6778	6.48401	0.601095
MIR6787	5.77719	0.517674
MIR6791	8.49797	0.475272
MIR6800	7.91837	0.535669
MIR6805	10.0048	0.44455
MIR6824	5.40884	0.44177
MIR6831	6.47076	0.528358
MIR6837	6.66954	0.400197
MIR6840	7.8853	0.714721
MIR6845	6.18235	0.577309
MIR6849	7.46304	0.467638
MIR6859-1	7.712	0.492907
MIR6859-2	7.712	0.492907
MIR6866	7.22201	0.452167
MIR6878	6.8063	0.424864
MIR6883	6.93696	0.662275
MIR6884	6.06708	0.5131
MIR6890	10.0963	0.584079
MIR7110	9.35822	0.682919
MIR7112	6.72807	0.575048
MIR7114	5.53884	0.505435
MIR7703	11.2072	0.649592
MIR8071-1	11.0851	2.17286
MIR8071-2	11.0851	2.17286
MIR8085	5.89018	0.472921
MIR939	7.46304	0.467638
MIRLET7D	6.17605	0.545712
MIS12	8.22515	0.691094

MIS18A	5.33812	0.653362
MIS18BP1	5.13306	0.599239
MITD1	6.83489	0.777289
MIXL1	6.32791	0.645703
MKI67	5.19774	0.730845
MKKS	7.41892	0.488941
MKLN1	5.72582	0.473413
MKNK2	9.20154	0.593899
MKRN1	7.98198	0.623398
MLEC	9.00423	0.534041
MLF2	7.4324	0.510362
MLH1	8.68819	0.458218
MLKL	7.70393	1.08922
MLLT11	6.22414	1.10605
MLLT3	7.60432	1.2001
MLN	4.88405	0.463104
MLST8	6.12018	0.464506
MMACHC	6.479	0.470607
MMADHC	10.5508	0.454913
MMD	5.36385	0.54073
MMGT1	9.29034	0.601845
MMP17	5.69988	0.400628
MMP9	5.88682	1.03352
MNAT1	6.44737	0.593952
MNDA	5.36556	1.71934
MOAP1	8.00927	0.609261
MOB1A	7.57333	0.571068
MOB1B	5.87116	0.835718
MOB3A	7.26829	0.6387
MOB4	8.07892	0.545811
MOCS2	5.14801	0.409556
MOGS	7.97619	0.483247
MON1A	6.5322	0.485723
MON1B	6.69012	0.542143
MON2	5.74159	0.437318
MORC1	4.85145	1.51907
MORC3	6.02871	0.616914
MORC4	6.15436	0.811981
MORF4L1	10.0865	0.525157
MORF4L2	8.73215	0.744454
MORN2	5.22858	0.57799
MOSPD1	7.16474	0.662769
MOSPD2	5.69049	0.526152
MOXD1	6.21294	1.08439
MPC1	6.50819	0.551229
MPC2	7.4834	0.570949
MPG	7.31701	0.442337

MPHOSPH10	7.43408	0.632703
MPHOSPH6	5.25022	0.406487
MPHOSPH8	6.46763	0.534265
MPHOSPH9	5.90735	0.580515
MPLKIP	8.16246	0.750119
MPND	6.54823	0.590546
MPO	4.81619	0.964527
MPP1	6.18517	0.696015
MPP5	5.4663	0.6346
MPST	7.71867	0.484798
MPV17	7.10504	0.468954
MPV17L2	7.18812	0.455613
MPZL1	6.48928	0.416142
MR1	5.92559	0.459484
MRFAP1	10.7846	0.55983
MRFAP1L1	8.1947	0.758902
MRGBP	6.19403	0.400047
MRI1	7.45371	0.408553
MROH7-TTC4	6.56649	0.614999
MRPL1	7.70017	0.735597
MRPL10	7.17343	0.573084
MRPL11	7.94452	0.464671
MRPL13	7.16215	0.886755
MRPL14	8.27824	0.589793
MRPL15	8.45389	0.739801
MRPL16	7.84458	0.593472
MRPL17	8.24289	0.59245
MRPL18	8.79815	0.562096
MRPL2	6.57141	0.432493
MRPL21	8.19934	0.486103
MRPL22	7.97764	0.617943
MRPL24	8.70438	0.559551
MRPL27	8.25541	0.64201
MRPL3	10.3985	0.655296
MRPL30	6.00926	0.455927
MRPL32	9.68316	0.632555
MRPL34	8.33489	0.584752
MRPL36	9.10941	0.64162
MRPL37	7.97576	0.609749
MRPL39	5.50583	0.527853
MRPL4	6.73118	0.537581
MRPL40	9.11704	0.462687
MRPL42	6.86767	0.568381
MRPL43	7.01622	0.448717
MRPL44	6.29064	0.522589
MRPL45	7.74845	0.631463
MRPL46	7.21189	0.585738

MRPL48	8.13204	0.53193
MRPL49	7.91648	0.586196
MRPL50	6.25517	0.655478
MRPL51	8.77865	0.432703
MRPL53	9.40064	0.492665
MRPL54	9.02524	0.468555
MRPL57	7.98013	0.462092
MRPL9	8.38454	0.577029
MRPS10	7.20232	0.597642
MRPS14	7.39066	0.634341
MRPS17	7.43484	0.783062
MRPS18A	7.21914	0.415888
MRPS18B	8.6569	0.57042
MRPS2	7.50493	0.839179
MRPS21	9.79062	0.786753
MRPS22	8.0478	0.480848
MRPS24	10.6221	0.674739
MRPS25	6.18603	0.448502
MRPS28	7.49286	0.736694
MRPS30	6.14836	0.583564
MRPS31	7.19818	0.518921
MRPS31P5	5.34423	0.568436
MRPS33	9.40595	0.584787
MRPS34	7.5956	0.414155
MRPS35	7.67261	0.778909
MRPS36	8.86838	0.628676
MRPS6	7.65809	0.669015
MRPS7	7.52745	0.461722
MRPS9	7.23998	0.550241
MRRF	7.17667	0.717586
MRS2	5.48825	0.415404
MRTO4	6.42466	0.483302
MS4A1	5.20544	2.4292
MSANTD2	5.99554	0.865871
MSANTD3	6.76507	0.618164
MSANTD3-TMEFF1	4.84889	1.59101
MSANTD4	4.96613	0.555946
MSH2	5.425	1.09135
MSH5	5.76652	0.710606
MSH5-SAPCD1	5.25198	0.484336
MSI2	7.13332	0.579279
MSL2	7.47702	0.776174
MSL3	6.47375	0.542356
MSMO1	7.74893	1.01929
MSRB1	6.76351	0.675061
MST1	6.80433	0.470362
MST1P2	6.08891	0.544274

MST4	6.71374	0.900637
MSTO1	7.07936	0.655462
MSTO2P	7.48057	0.622357
MT1E	6.78414	0.75909
MT1F	6.53727	0.723324
MT1G	5.48051	0.600458
MT1H	6.80939	0.712637
MT1HL1	7.62911	0.68674
MT1X	6.86754	0.953772
MT2A	7.90638	0.867556
MTA1	6.37498	0.537295
MTA2	4.84393	0.410293
MTAP	4.85516	0.408351
MTCH1	9.56401	0.428469
MTCH2	7.59889	0.598243
MTDH	10.4289	0.714724
MTERF1	5.61102	0.48048
MTERF2	5.32903	0.554248
MTERF3	7.13466	0.65981
MTF2	5.91441	0.575342
MTFMT	6.58136	0.688939
MTFP1	6.79114	0.449571
MTFR1	5.60658	0.430728
MTG1	6.13838	0.478062
MTHFD1	5.9478	0.42949
MTHFD1L	5.44526	0.575827
MTHFD2	7.17581	0.723787
MTIF2	8.02182	0.66962
MTMR1	6.57815	0.516907
MTMR12	6.94038	0.545481
MTMR2	5.99676	0.489713
MTMR4	7.04725	0.652484
MTMR9	5.93363	0.400177
MTO1	6.67679	0.60927
MTPAP	6.77188	0.550774
MTR	6.12566	0.663342
MTRR	8.09848	0.650307
MTURN	5.25814	0.539219
MTUS1	6.57752	0.818351
MTX1	7.9377	0.586148
MTX2	8.07749	0.539012
MTX3	6.11493	0.482705
MUC1	5.40348	0.40625
MUC20	5.03275	0.538986
MUS81	7.43756	0.401796
MUT	7.0797	0.583928
MVB12A	6.08655	0.6417

MVD	5.81315	0.400608
MVP	7.57721	0.515378
MX1	9.26312	1.48381
MX2	7.02254	1.14798
MXD1	6.37408	0.751958
MXI1	9.65072	0.719859
MXRA7	4.87619	0.537383
MYADM	6.21639	1.33979
MYBL1	5.33161	0.836052
MYBL2	6.55523	0.593215
MYC	10.3563	1.767
MYCBP2	9.04542	0.644927
MYD88	8.36059	0.536
MYEF2	5.15373	0.578996
MYEOV	7.70358	1.21918
MYF6	5.17728	0.913285
MYH9	7.06013	0.58966
MYL12A	7.3451	0.695302
MYL12B	9.68381	0.492965
MYL5	5.67917	0.463075
MYL6B	7.63368	0.510693
MYLIP	6.652	0.817042
MYO18A	6.25857	0.580102
MYO1F	5.01525	0.70629
MYO1G	5.77162	0.477522
MYO5A	5.54463	0.712615
MYO5C	6.94328	1.27526
MYO6	6.9052	1.0228
MYPOP	5.69963	0.446453
MZB1	11.9767	0.701199
MZF1	7.13847	0.407238
MZT1	5.55707	0.484817
MZT2A	9.33191	0.514363
N4BP1	6.76979	0.437378
N4BP2	6.67376	0.814957
N4BP2L2	6.45921	0.574391
N6AMT2	6.26408	0.431557
NAA10	7.6355	0.53901
NAA16	6.07172	0.642619
NAA20	8.49916	0.419555
NAA25	6.09252	0.603574
NAA35	7.23139	0.534049
NAA38	8.44482	0.558944
NAA40	5.93398	0.560601
NAA50	6.88163	0.536419
NAAA	6.47233	0.546534
NABP1	5.95396	0.634081

NABP2	5.44406	0.455103
NADK2	6.72804	0.653294
NAE1	8.52871	0.513714
NAF1	4.91999	0.530436
NAGA	6.65484	0.614069
NAGK	8.23159	0.735224
NAGPA	6.32602	0.492245
NAIP	5.53854	0.717031
NAMPT	8.81235	1.06562
NANP	5.27283	0.670192
NAP1L1	8.83176	0.564855
NAP1L2	5.7264	1.658
NAP1L5	5.74338	0.882323
NAPA	7.42677	0.488188
NAPB	5.24072	0.462608
NAPG	6.37075	0.593306
NAPRT	6.8273	0.61123
NARR	6.11464	1.29391
NARS	10.3136	0.609352
NARS2	7.96842	0.623755
NASP	5.46141	0.719322
NAT1	5.50354	0.875553
NAT10	7.24308	0.576784
NAT9	7.25583	0.415004
NBEA	6.75801	1.29979
NBN	6.63654	0.545144
NBPF1	6.37337	0.400914
NBPF10	8.55934	0.500521
NBPF11	7.74775	0.453543
NBPF12	7.99512	0.484897
NBPF14	8.41907	0.507866
NBPF15	7.74881	0.457069
NBPF19	8.18495	0.492183
NBPF20	7.84736	0.429853
NBPF25P	8.77177	0.522831
NBPF26	7.98111	0.530451
NBPF8	7.57912	0.408964
NBPF9	8.33044	0.523126
NCAM1	5.39171	0.797966
NCAPD2	5.65062	0.693037
NCAPD3	6.76886	0.717255
NCAPG	4.96769	1.09896
NCAPG2	5.40952	1.05622
NCBP1	5.03953	0.458281
NCBP2	7.82145	0.58683
NCBP2-AS2	7.98721	0.498127
NCF1	7.11103	0.640005

NCF1B	7.10688	0.690347
NCF1C	7.10688	0.690347
NCF2	5.26235	1.23593
NCF4	7.09822	1.10823
NCK1	6.01668	0.530625
NCK1-AS1	6.22018	0.588837
NCK2	7.71927	0.690041
NCKAP1	5.41607	1.06428
NCKAP1L	6.99621	0.528739
NCOA1	6.82149	0.509625
NCOA2	6.47523	0.536196
NCOA3	7.77502	0.661183
NCOA4	10.0536	0.626198
NCOA5	5.71132	0.481576
NCOA7	6.08638	0.515954
ND2	13.5158	0.414151
ND6	8.5883	0.783739
NDC1	6.33389	0.714724
NDC80	5.58773	1.30695
NDFIP1	8.17705	0.512942
NDFIP2	5.89014	0.532178
NDN	5.17922	1.15171
NDNF	7.84769	1.93849
NDNL2	6.52834	0.568013
NDRG1	7.58142	0.797836
NDRG3	5.77547	0.530127
NDUFA1	11.0563	0.481559
NDUFA12	9.8663	0.482109
NDUFA13	10.4176	0.500962
NDUFA3	10.3641	0.442301
NDUFA4	11.3163	0.501397
NDUFA5	6.86204	0.519465
NDUFA6	8.07419	0.47827
NDUFA8	9.26345	0.601533
NDUFA9	8.85515	0.494765
NDUFAB1	10.8865	0.441173
NDUFAF1	8.15422	0.803451
NDUFAF2	8.37002	0.551514
NDUFAF3	8.42594	0.446742
NDUFAF4	7.38854	0.717
NDUFAF6	5.4701	0.41573
NDUFB10	8.97104	0.430211
NDUFB11	10.2882	0.405772
NDUFB3	9.71298	0.460283
NDUFB5	10.0901	0.580331
NDUFB6	7.00849	0.406018
NDUFB9	10.4644	0.492927

NDUFC1	7.65644	0.456967
NDUFC2-KCTD14	6.34622	0.444056
NDUFS2	8.91175	0.615525
NDUFS3	8.31611	0.426299
NDUFS4	6.23167	0.479281
NDUFS5	10.038	0.47477
NDUFS6	9.29563	0.553237
NDUFV2	9.47864	0.567376
NDUFV3	7.59544	0.545931
NEAT1	8.61682	0.706594
NEB	5.6146	1.27833
NECAP1	7.61036	0.555066
NECAP2	6.41024	0.518471
NEDD1	5.97671	0.607992
NEDD4L	5.00087	0.510028
NEDD8	9.58897	0.437655
NEDD8-MDP1	7.12626	0.551959
NEDD9	5.977	0.741484
NEIL1	5.6485	0.443436
NEK3	5.27984	0.556617
NEK4	5.52779	0.41308
NEK6	5.03116	0.425738
NEK7	9.02103	0.936673
NEK8	6.31601	0.448039
NELFB	7.41662	0.512537
NELFCD	8.20055	0.556463
NELFE	8.93863	0.518316
NELL2	4.82339	0.743878
NEMF	6.93412	0.585023
NEO1	5.33622	0.433507
NES	5.84239	1.08398
NET1	6.66034	1.05575
NETO2	5.07866	1.02514
NEU1	8.98043	0.584669
NFAT5	5.99191	0.709744
NFATC2IP	7.008	0.536837
NFE2L1	8.04549	0.672671
NFE2L3	5.79734	1.17806
NFIC	5.39831	0.639106
NFIL3	8.43149	1.00514
NFKB1	7.5303	0.613558
NFKB2	5.59423	0.403833
NFKBIA	8.64078	0.776027
NFKBIE	6.90185	0.787414
NFKBIZ	10.1131	1.09344
NFS1	5.5241	0.439736
NFU1	8.14229	0.501803

NFX1	6.74378	0.492933
NFXL1	6.66205	0.704597
NFYB	5.48296	0.535466
NGFRAP1	6.93193	1.61699
NGLY1	8.54849	0.484404
NGRN	7.99709	0.445803
NHEJ1	6.14514	0.441655
NHLRC3	5.67345	0.625523
NHP2	10.5637	0.583787
NHP2P2	7.75675	0.484822
NICN1	6.5384	0.450278
NID2	5.02484	1.97226
NIF3L1	7.44472	0.705926
NIFK	6.76261	0.462276
NIN	4.91557	0.446011
NINJ1	6.76345	0.601566
NINJ2	5.1067	0.627232
NINL	5.31109	0.613504
NIP7	5.68729	0.559813
NIPA1	5.50924	0.506937
NIPA2	8.90275	0.453409
NIPAL2	5.24435	0.520765
NIPAL4	5.40305	0.557092
NIPBL	7.08842	0.579445
NIPSNAP3A	6.03759	1.1008
NIT2	8.55051	0.598827
NKAP	6.40285	0.651756
NKIRAS1	6.16213	0.812972
NKRF	6.66301	0.56198
NKTR	6.37561	0.51638
NKX1-1	5.62095	0.418773
NKX6-3	5.57563	0.430888
NLE1	5.24553	0.410918
NLGN4X	5.8906	1.78652
NLK	6.51212	0.845223
NLRC5	6.98434	0.805505
NLRP11	4.86192	1.24293
NMB	6.86516	0.620026
NMD3	8.43221	0.522368
NME1	10.0691	0.725961
NME1-NME2	11.4652	0.547084
NME2	11.4652	0.547084
NME3	7.30498	0.440978
NME4	7.41102	0.817396
NME6	5.60471	0.530562
NME7	6.10525	0.846177
NMI	7.33063	0.806669

NMRAL1	6.69898	0.639475
NMRK1	6.70468	0.562449
NNT	6.21307	0.460573
NNT-AS1	6.38948	0.771891
NOA1	8.37126	0.619769
NOB1	7.64952	0.727349
NOC3L	7.65457	0.829035
NOL11	7.71649	0.662206
NOL4	4.9312	1.54527
NOL7	9.27561	0.435652
NOL8	8.37555	0.577783
NOLC1	8.08961	0.494536
NOM1	5.72183	0.498534
NOMO1	10.0789	0.712953
NOMO2	10.0789	0.712953
NOMO3	8.98865	0.562616
NONO	9.29576	0.456143
NOP10	10.6954	0.503859
NOP14	7.17435	0.525547
NOP16	6.16578	0.415712
NOP2	7.44996	0.578495
NOP56	7.98352	0.525796
NOP58	10.4349	0.553616
NOSIP	7.80147	0.443388
NOTCH2	6.52217	0.716799
NOTCH2NL	7.74076	0.954125
NPAT	5.82705	0.480778
NPC2	10.198	0.978709
NPDC1	5.98775	0.709236
NPEPPS	6.34621	0.497243
NPHP3	5.72073	0.478734
NPHP3-ACAD11	6.58554	0.471767
NPIPB11	5.72441	0.420936
NPIPB15	6.13302	0.495379
NPIPB3	6.10631	0.427282
NPIPB4	6.4516	0.573535
NPIPB5	6.4516	0.573535
NPIPB6	5.18105	0.406342
NPLOC4	7.58781	0.426421
NPM1	11.4948	0.62017
NPM1P22	6.87644	0.459498
NPM3	6.30462	0.701423
NPPA	6.34611	0.402167
NPTN	8.79028	0.887119
NPTN-IT1	5.19018	0.660221
NQO2	5.68301	0.424726
NR0B2	6.16723	0.402284

NR1D1	6.47481	0.520906
NR1D2	7.6895	0.84383
NR1H2	7.17495	0.424426
NR2C2	5.87897	0.467491
NR3C1	8.88085	0.848008
NR4A1	6.75436	0.526414
NR4A2	8.12339	1.20061
NRAS	7.77947	0.804407
NRBF2	6.54698	0.613079
NRBP1	7.06239	0.409073
NRBP2	6.18235	0.577309
NRD1	7.9511	0.586182
NRG2	5.13228	0.672499
NRG3	6.51643	1.35131
NRGN	5.17042	0.750079
NRM	6.66115	0.412389
NRROS	6.33169	0.679541
NSA2	11.1859	0.650011
NSDHL	6.08101	0.633764
NSMAF	5.53667	0.593035
NSMCE1	8.03901	0.763559
NSMCE2	8.23525	0.625968
NSRP1	7.51971	0.484348
NSUN2	8.03954	0.542864
NSUN5	8.10073	0.462247
NSUN5P1	8.7269	0.510139
NSUN5P2	8.89817	0.522267
NSUN6	6.47893	0.594468
NT5C2	7.64848	0.592232
NT5C3A	9.32741	0.734744
NT5C3B	8.3789	0.599839
NT5DC1	5.89938	0.483731
NT5DC2	5.33672	0.483183
NTAN1	7.44359	0.936443
NTHL1	6.28148	0.636558
NTPCR	6.20308	0.413973
NTRK3	4.81385	0.474483
NUAK2	7.55488	0.89986
NUBP1	7.0089	0.503599
NUBPL	5.82948	0.528548
NUCB1	8.03205	0.577978
NUCB2	7.88531	0.787697
NUCKS1	7.41	0.633172
NUDCD1	5.50724	0.577116
NUDT1	6.49295	0.71588
NUDT15	5.3563	0.660795
NUDT18	5.95688	0.437295

NUDT19	6.91832	0.760965
NUDT2	7.1847	0.499359
NUDT21	6.75256	0.702572
NUDT3	6.61804	0.442867
NUDT4P1	7.99093	0.556363
NUDT4P2	8.2909	0.538674
NUDT5	7.41969	0.583862
NUDT6	5.88068	0.55814
NUDT9	7.66977	0.602892
NUFIP2	7.243	0.537081
NUMB	5.30655	0.535772
NUP107	7.31992	0.741613
NUP133	5.87724	0.611936
NUP153	5.4936	0.486006
NUP155	6.36505	0.688291
NUP160	5.74614	0.575524
NUP205	5.63937	0.495991
NUP210	7.25974	0.438447
NUP35	7.49583	0.852151
NUP37	6.48683	0.470366
NUP43	6.10284	0.687547
NUP85	6.59646	0.72658
NUP88	6.73488	0.418071
NUP98	6.16364	0.458902
NUPL1	5.20035	0.529
NUPL2	7.13221	0.564887
NUPR1	5.21056	0.457608
NUS1	7.47027	0.587028
NUS1P3	5.85006	0.546283
NUSAP1	6.40948	1.44189
NVL	6.95978	0.613066
NXF1	8.64099	0.649797
NXPE3	7.04379	1.14719
NXPE4	6.08885	0.931804
NXT1	7.99791	0.471447
NXT2	6.73302	0.944148
OARD1	7.04942	0.539487
OAS1	7.31051	0.971016
OAS2	6.88958	0.727138
OAS3	5.2083	0.508508
OASL	5.28874	0.487799
OAT	9.34029	0.994185
OBP2B	4.83288	0.421341
OCA2	5.52984	0.437511
OCIAD2	8.53375	0.436469
OCLN	5.27134	0.606242
OCRL	5.86872	0.459843

ODC1	11.3758	0.482927
ODF2	6.04926	0.459816
OFD1	6.87558	0.645195
OGFOD1	6.33296	0.401265
OGFOD3	7.31397	0.483437
OGFRL1	5.58488	0.769897
OGT	8.79607	0.567604
OIP5	4.8627	1.14552
OIP5-AS1	8.03539	0.577443
OMA1	7.74972	0.622633
OPA1	5.09251	0.490606
OPHN1	9.62906	0.468874
OPN3	6.49989	1.00642
OPTN	6.22158	0.613444
OR7E12P	5.53601	0.431914
OR7E37P	6.91199	0.600376
OR7E47P	5.18288	0.432811
ORAI1	6.14751	0.444939
ORAI3	7.05254	0.579912
ORC2	5.99823	0.644829
ORC3	7.08923	0.663453
ORC4	6.02106	0.48262
ORC5	5.07183	0.522899
ORC6	6.43149	0.557513
ORMDL1	8.10265	0.47367
ORMDL2	9.10408	0.55379
ORMDL3	7.69698	0.485061
OS9	9.69512	0.53251
OSBP	7.39242	0.439725
OSBPL10	5.89397	0.585366
OSBPL11	5.98863	0.640804
OSBPL1A	4.82939	0.945239
OSBPL3	5.5788	1.16991
OSBPL8	6.50527	0.504781
OSBPL9	9.08553	0.520587
OSER1	7.22275	0.485621
OSGEP	6.52805	0.437884
OSGEPL1	6.41798	0.614645
OST4	11.3242	0.410692
OSTC	10.7907	0.461199
OSTF1	7.77972	0.597706
OSTM1	6.12007	0.648289
OTUD1	8.10225	0.830443
OTUD4	5.51881	0.434144
OTUD6B	6.62447	0.81636
OTUD6B-AS1	6.90456	0.571556
OVAAL	4.90834	0.919506

OVGP1	6.52161	0.440698
OVOL1	5.20388	0.644252
OVOL3	5.1636	0.524181
OVOS	7.89465	1.80851
OVOS2	7.89465	1.80851
OXA1L	9.56729	0.509804
OXCT1	7.74615	0.584504
OXLD1	6.93792	0.443167
OXNAD1	7.26225	0.550123
OXR1	7.18642	0.54439
OXSM	7.74616	0.601803
OXSR1	7.07359	0.540567
P2RX1	7.66377	1.33261
P2RX4	7.81113	0.593319
P2RX5	6.92247	0.725322
P2RX5-TAX1BP3	5.92055	0.606799
P2RX7	5.7226	0.964851
P2RY10	6.32241	1.09384
P2RY6	5.39289	0.882121
P2RY8	8.8742	0.828233
P4HA1	5.76704	0.481994
P4HA2	4.97225	0.630744
P4HB	10.4312	0.507432
P4HTM	8.38665	0.707955
PA2G4	7.05336	0.466808
PAAF1	6.87849	0.41898
PABPC1L	5.43577	0.446203
PABPC4	10.6601	0.707292
PABPN1	7.52931	0.490364
PACSIN1	5.00629	0.405947
PACSIN3	4.93952	0.407897
PAFAH1B1	6.43372	0.542513
PAFAH1B3	7.22649	0.568191
PAGE5	5.18019	0.425679
PAICS	6.9681	0.485416
PAIP1	7.52972	0.534304
PAIP2B	7.24766	0.959668
PAK1	5.66386	0.617736
PAK1IP1	7.02638	0.666866
PAK2	5.75983	0.440392
PALB2	6.06915	0.663627
PALD1	6.30451	0.912398
PALM2-AKAP2	8.78727	0.592077
PAM	8.4997	0.73689
PAM16	7.51656	0.482488
PAN2	7.30158	0.632773
PAN3	8.13498	0.767484

PANK3	6.07319	0.469524
PANK4	6.45343	0.48781
PANX1	5.35129	0.518211
PAPD4	6.78281	0.696777
PAPD5	6.46395	0.657902
PAPD7	8.14062	0.745272
PAPOLA	7.87962	0.505968
PAPSS1	9.61605	0.596801
PAPSS2	5.5581	1.11331
PAQR6	6.61626	0.618741
PARD3	5.96741	0.891084
PARG	6.48947	0.41464
PARK7	11.4736	0.419678
PARM1	7.4339	1.30141
PARN	7.48698	0.509871
PARP1	8.46299	0.661716
PARP12	7.52664	0.814764
PARP14	5.81723	0.528323
PARP16	7.30183	0.402283
PARP2	7.5213	0.515913
PARP3	6.01262	0.43649
PARP4	7.65039	0.665028
PARP8	6.35606	0.861755
PARP9	6.44637	0.847856
PARS2	5.44475	0.442442
PARVB	5.46844	0.428557
PARVG	5.41363	0.492322
PATL2	6.03637	0.4569
PAWR	5.05674	0.478304
PAX5	5.96162	0.836797
PAXBP1	6.51665	0.583491
PAXIP1	6.77027	0.735955
PAXIP1-AS1	7.28283	0.743712
PBDC1	6.47271	0.571206
PBK	5.05936	1.44673
PBX3	4.85806	0.720619
PBXIP1	7.76902	0.893095
PC	4.82418	0.472837
PCAT6	5.52216	0.780045
PCBD1	6.80084	0.534082
PCBP1	9.36248	0.509724
PCBP2	7.27484	0.520353
PCBP3	5.95499	0.509567
PCBP4	6.72275	0.483631
PCDHGA1	5.89027	0.518407
PCDHGA10	5.68721	0.502669
PCDHGA11	5.47551	0.46324

PCDHGA12	5.83864	0.541544
PCDHGA2	6.24984	0.618852
PCDHGA3	5.51554	0.44255
PCDHGA4	6.09252	0.556796
PCDHGA5	5.83864	0.541544
PCDHGA6	5.83864	0.541544
PCDHGA7	6.24984	0.618852
PCDHGA8	5.94204	0.519614
PCDHGA9	5.93155	0.513882
PCDHGB1	6.24984	0.618852
PCDHGB2	6.24984	0.618852
PCDHGB3	6.24984	0.618852
PCDHGB4	6.24984	0.618852
PCDHGB5	6.15884	0.516403
PCDHGB6	5.69603	0.506516
PCDHGB7	5.34088	0.451489
PCDHGC4	5.4969	0.429308
PCDHGC5	5.40178	0.428276
PCED1B	6.26342	1.07141
PCF11	7.70608	0.529854
PCGF1	6.96248	0.464882
PCGF5	6.93579	0.65186
PCGF6	5.62581	0.492865
PCID2	6.32542	0.495377
PCK2	7.28524	0.813065
PCM1	6.16522	0.457915
PCMT1	8.09378	0.716255
PCMTD1	7.83215	0.580944
PCNA	8.22805	1.22888
PCNP	7.81102	0.434247
PCNXL3	5.89163	0.435851
PCOLCE-AS1	6.16288	0.541764
PCSK1N	4.88518	0.631244
PCTP	6.91439	0.607784
PCYOX1	7.58265	0.769483
PCYOX1L	5.42759	0.789248
PDCD10	9.32784	0.669904
PDCD11	6.56674	0.412636
PDCD2	5.63651	0.414982
PDCD2L	7.38668	0.853882
PDCD4	8.03146	1.00137
PDCD4-AS1	5.04694	0.608828
PDCD5	6.51503	0.501102
PDCD6IP	7.5624	0.557837
PDCD7	6.29301	0.431129
PDCL	6.54032	0.472471
PDCL3	7.50006	0.749816

PDCL3P4	7.50006	0.749816
PDE4B	8.07764	1.08568
PDE7A	4.94586	0.50337
PDE8A	5.86505	0.417244
PDF	7.05945	0.506322
PDHA1	7.35431	0.453792
PDHB	8.68958	0.619405
PDHX	6.86242	0.693858
PDIA3	8.0965	0.712721
PDIA4	9.36727	0.725424
PDIA5	9.35822	0.682919
PDIA6	11.1626	0.523971
PDIK1L	6.64633	0.859684
PDK1	9.42347	0.929583
PDLIM1	8.69569	1.26844
PDLIM3	4.89549	0.852044
PDP1	6.50743	0.711679
PDP2	5.177	0.467289
PDS5A	6.75822	0.412518
PDSS1	5.02612	0.428191
PDSS2	5.70012	0.548906
PDXDC2P	5.35839	0.469753
PDXP	6.02154	0.487561
PDZD11	8.42258	0.47182
PDZD8	5.12775	0.483122
PDZRN4	5.90131	1.59446
PEA15	6.92836	0.889869
PEAK1	6.3448	0.41784
PEAR1	5.21205	0.418453
PEBP1	9.51241	0.623075
PECAM1	8.38598	0.945244
PEF1	7.61359	0.507994
PELI1	10.6137	1.18535
PEMT	6.78099	0.531548
PER1	6.93696	0.662275
PER2	5.35017	0.459767
PERP	5.69546	0.795352
PET100	9.68633	0.43413
PET112	6.18852	0.430688
PEX1	5.71292	0.514458
PEX11B	7.69113	0.722716
PEX12	6.31825	0.720718
PEX19	6.81677	0.542299
PEX2	6.13507	0.513774
PEX3	5.79802	0.597091
PEX6	5.22827	0.499327
PEX7	5.49763	0.539921

PF4	6.93444	1.41844
PFAS	6.42008	0.789495
PFDN2	8.38327	0.530849
PFDN5	10.8793	0.437308
PFKFB2	4.8515	0.492962
PFKFB3	4.90945	0.982809
PFKM	7.58899	0.798671
PFN1	9.26533	0.691708
PFN2	7.46697	1.3705
PGAP2	7.26224	0.494234
PGGT1B	5.97628	0.579452
PGK1	7.56804	0.45142
PGLYRP1	5.75131	0.648164
PGM1	8.01973	0.528617
PGM2	5.89867	0.53268
PGM3	7.02018	0.783053
PGM5-AS1	5.5084	0.418075
PGRMC1	7.67587	0.650159
PGRMC2	8.18857	0.673226
PHACTR1	6.01923	1.372
PHACTR4	6.30618	0.529636
PHAX	6.86367	0.67568
PHB	7.99045	0.442704
PHB2	10.7972	0.532148
PHC3	5.99624	0.406006
PHF1	7.7188	0.523332
PHF10	6.69773	0.587628
PHF11	6.09372	0.511951
PHF13	6.59052	0.669211
PHF14	5.3331	0.486581
PHF19	6.29818	0.862299
PHF23	6.24715	0.528657
PHF5A	7.93433	0.482879
PHF6	6.2013	0.475891
PHGDH	8.37395	0.618246
PHKB	5.29804	0.515993
PHLDA1	5.68076	1.3128
PHLDA3	6.07909	0.408694
PHLPP2	5.04138	0.506739
PHPT1	8.47444	0.460702
PHTF1	5.05955	0.565136
PHTF2	5.08425	0.436387
PHYH	6.97903	1.05333
PHYHD1	4.93896	0.81315
PI16	6.00266	0.415117
PI4K2B	7.9252	0.563031
PI4KA	7.19336	0.496392

PI4KAP1	8.20654	0.505611
PI4KAP2	8.20654	0.505611
PIAS1	8.17583	0.661461
PIAS3	6.32143	0.472762
PIBF1	5.35096	0.751013
PICALM	6.69831	0.527659
PIGA	5.97612	0.860234
PIGB	5.77155	0.640903
PIGC	6.08403	0.40995
PIGF	8.05097	0.520043
PIGH	6.26509	0.704977
PIGK	6.4821	0.468789
PIGM	5.86728	0.685974
PIGN	5.69627	0.667911
PIGP	10.0045	0.585734
PIGU	6.88677	0.616748
PIGV	6.48533	0.490594
PIGW	6.35029	0.813133
PIGY	8.96235	0.483837
PIK3AP1	6.0139	1.08962
PIK3C2A	6.88888	0.598284
PIK3C2B	6.6892	0.537917
PIK3CA	5.8202	0.532797
PIK3CB	5.67649	0.478402
PIK3CD	5.52662	0.41367
PIK3CG	6.1206	0.919192
PIK3IP1	6.1829	0.446952
PIK3R1	6.53225	0.661444
PIK3R2	6.67003	0.404942
PIK3R3	4.94111	0.775958
PIKFYVE	5.77163	0.410125
PILRA	4.84238	0.412607
PILRB	7.8853	0.714721
PIM1	7.18823	0.767284
PIM2	9.79789	0.81871
PIM3	7.64894	0.784584
PIN1	7.08649	0.467337
PINK1	7.17696	0.518917
PINLYP	6.3674	0.800082
PINX1	6.5544	0.447538
PIP4K2A	6.64143	0.611632
PIP5K1A	6.30989	0.413007
PIP5K1B	6.54558	0.570822
PIP5K1C	6.96862	0.418534
PIP5KL1	5.13246	0.4154
PITPNA	6.97869	0.426201
PITPNA-AS1	7.58552	0.651077

PITPNB	8.98796	0.496524
PJA1	7.93395	0.559275
PJA2	9.28801	0.590191
PKD2	6.23567	0.882381
PKHD1L1	6.6068	1.04396
PKIG	5.57399	0.936935
PKM	7.17278	0.457222
PKN1	6.20713	0.426674
PKN2	5.95077	0.584135
PKP4	5.7578	0.65722
PLA2G12A	6.93642	0.529459
PLA2G15	5.4634	0.492036
PLA2G16	6.99035	0.74508
PLA2G2D	5.14521	0.47635
PLA2G4A	5.78536	1.23883
PLAA	5.50749	0.500487
PLAC8	9.17232	1.60291
PLAGL2	5.49833	0.565663
PLAUR	4.95797	0.550716
PLBD1	5.30578	1.16795
PLBD2	6.09906	0.434371
PLCB3	5.91202	0.427681
PLCG2	6.45013	0.578332
PLCL2	6.26369	0.512304
PLCXD1	6.21357	0.408348
PLCXD2	5.29668	0.530374
PLD3	7.80868	0.7045
PLD4	4.97466	0.832878
PLD6	5.30982	0.448109
PLEC	6.16879	0.406718
PLEK	7.17645	1.29893
PLEKHA2	6.23257	0.761498
PLEKHA4	4.90226	0.414461
PLEKHA7	4.90457	0.817016
PLEKHF2	7.27749	1.31788
PLEKHG2	5.75931	0.407301
PLEKHG4	5.52753	0.609544
PLEKHM3	4.93658	0.441287
PLEKHN1	7.00891	0.419783
PLEKHO1	5.69952	1.06709
PLGRKT	7.2257	0.63843
PLIN2	5.83975	0.483752
PLIN3	7.39625	0.44318
PLIN4	5.85486	0.484777
PLK1	5.25276	0.447056
PLK1S1	6.02241	0.427617
PLK2	5.01104	1.16908

PLOD1	6.39798	0.50866
PLOD3	8.55164	0.586228
PLP2	10.2497	0.731236
PLRG1	6.34858	0.633835
PLSCR1	5.63239	0.79444
PLSCR3	7.49268	0.445814
PLTP	5.94049	1.38776
PLXNB2	5.56975	0.467513
PLXNC1	6.95266	0.601416
PM20D2	6.65383	0.623797
PMAIP1	7.93932	2.49542
PMEPA1	6.9318	0.856739
PMF1	6.21097	0.625262
PMF1-BGLAP	5.2343	0.958823
PMM1	6.31381	0.421642
PMM2	7.15159	0.48866
PMPCA	8.14261	0.441338
PMS2P1	6.63818	0.408421
PMS2P5	6.26658	0.460177
PMS2P8	6.42329	0.437093
PMVK	8.24325	0.633447
PNISR	7.95934	0.63516
PNKP	7.51027	0.469034
PNMA1	6.16596	1.15124
PNN	8.447	0.450301
PNOC	8.88122	1.06263
PNP	8.56943	1.15482
PNPLA4	5.62873	0.462843
PNPLA6	6.3619	0.440599
PNPLA8	8.76454	0.578646
PNPO	6.56893	0.473108
PNPT1	8.39481	0.656918
PNRC1	10.4695	0.442788
PNRC2	7.82797	0.664298
POC1B	7.39029	0.655264
POC1B-GALNT4	5.43221	0.585714
POC5	6.94189	0.641603
PODXL	5.06878	0.60165
PODXL2	4.80754	0.508092
POGK	5.76859	0.565434
POGLUT1	7.44069	0.682531
POGZ	6.50777	0.418955
POLA1	5.55597	0.732359
POLB	5.99603	0.429283
POLD1	6.06758	0.503562
POLD2	7.64213	0.622971
POLD4	7.39022	0.440891

POLDIP2	7.25843	0.412339
POLE	5.58462	0.416156
POLE2	4.92908	0.893871
POLE3	8.21713	0.735309
POLG2	6.53737	0.554138
POLK	5.94065	0.459741
POLM	5.56283	0.481207
POLQ	4.9452	0.745347
POLR1B	6.00662	0.448035
POLR1C	6.65959	0.535496
POLR1D	8.08812	0.68615
POLR2D	6.42136	0.454796
POLR2E	8.02198	0.470772
POLR2G	8.55869	0.603758
POLR2H	8.30873	0.513215
POLR2I	8.66932	0.419071
POLR2J	7.88794	0.494671
POLR2J2	7.11284	0.463492
POLR2J3	7.28041	0.571769
POLR2J4	5.94661	0.427486
POLR2K	8.51382	0.504657
POLR2M	6.19508	0.417484
POLR3C	6.43596	0.622896
POLR3D	6.3184	0.504303
POLR3GL	7.90092	0.574689
POM121C	7.71207	0.472802
POMC	5.36078	0.577799
POMGNT2	6.17708	0.552925
POMP	7.32239	0.550963
POMT1	5.90108	0.463268
POMZP3	6.00452	0.743461
PON2	6.45618	0.678999
POP5	9.14055	0.541064
POP7	7.36628	0.570767
POR	6.03247	0.439333
POT1	6.61273	0.690856
POTEM	5.17681	0.93127
POU2F2	6.6515	0.456827
PP7080	5.77067	0.547369
PPA1	11.1711	0.784025
PPAP2A	6.89275	0.830876
PPAP2B	5.66604	1.08661
PPAPDC1B	9.73469	0.7423
PPAPDC2	5.85175	0.909226
PPAPDC3	5.3099	0.550674
PPAT	7.01466	0.68566
PPBP	6.98425	2.29772

PPCDC	7.71888	0.845057
PPDPF	7.32705	0.741157
PPFIA1	6.30385	0.409389
PPFIBP2	7.31217	0.698848
PPIA	10.1414	0.403541
PPIB	12.2559	0.48093
PPID	6.38271	0.669036
PPIF	7.6369	0.621348
PPIG	7.12531	0.56687
PPIH	7.49941	0.497427
PPIL1	7.46828	0.789525
PPIL3	8.45876	0.650144
PPIL4	5.48722	0.787254
PPIP5K1	5.33847	0.405999
PPIP5K2	7.72612	0.616001
PPM1A	6.16827	0.504666
PPM1B	7.36871	0.529614
PPM1D	5.3888	0.459885
PPM1K	6.04395	0.570575
PPM1M	6.30502	0.454942
PPOX	5.60216	0.685207
PPP1CA	9.31437	0.450535
PPP1CB	8.2378	0.460193
PPP1CC	11.0261	0.540465
PPP1R10	6.65683	0.567292
PPP1R12A	7.10372	0.599259
PPP1R14B	7.82513	0.597974
PPP1R14D	4.89379	0.401606
PPP1R15A	9.21068	1.13032
PPP1R15B	9.72611	0.619948
PPP1R16B	5.8521	0.785219
PPP1R18	7.40146	0.605228
PPP1R2	7.72753	0.464936
PPP1R21	6.14852	0.78502
PPP1R26	6.6698	0.67579
PPP1R35	7.08162	0.495368
PPP1R3D	5.22834	0.552276
PPP1R3E	6.1548	0.52021
PPP1R8	7.83608	0.584406
PPP2CA	6.68916	0.425895
PPP2R1B	5.58436	0.48181
PPP2R5C	7.16808	0.532566
PPP2R5E	7.12257	0.579262
PPP3CA	8.05237	0.641501
PPP3CB	7.95913	0.453905
PPP3R1	5.68961	0.609634
PPP4C	7.82759	0.474675

PPP4R1	6.99058	0.65488
PPP4R2	6.20038	0.451343
PPP6C	8.20942	0.462
PPP6R3	7.57977	0.453283
PPRC1	6.82469	0.526873
PPT1	9.85729	0.498453
PPTC7	7.72891	0.628953
PPWD1	5.71172	0.450711
PQLC3	7.04189	0.462984
PRADC1	7.07146	0.666356
PRAF2	6.58585	0.657412
PRAM1	5.18386	0.46419
PRB1	6.64575	0.439797
PRC1	6.24321	1.17666
PRCC	6.47972	0.860719
PRDM1	8.82474	0.524773
PRDM12	5.44505	0.417917
PRDM15	5.8368	0.770413
PRDM2	5.94839	0.415211
PRDM5	5.32528	0.877029
PRDX1	10.3921	0.742606
PRDX3	6.85858	0.417168
PRDX4	12.2145	0.737642
PRDX5	9.74505	0.483778
PRDX6	9.54305	0.558467
PREB	8.55301	0.670705
PRELID1	8.82237	0.490585
PREPL	6.5212	0.463314
PREX1	5.51407	0.745399
PRG2	6.71407	1.63968
PRG3	5.1983	0.983561
PRICKLE1	4.93184	0.453584
PRICKLE2	5.38892	0.798654
PRICKLE4	10.4631	0.412488
PRIM1	6.98288	0.980482
PRIMPOL	6.32554	0.646136
PRKAB2	5.14015	0.517965
PRKACB	6.61244	0.874261
PRKAG1	7.96922	0.41892
PRKAR1A	7.86963	0.539782
PRKCB	5.91797	1.01218
PRKCD	7.77075	0.761526
PRKCI	6.31119	0.48224
PRKCSH	8.99297	0.435325
PRKCZ	6.31493	0.580045
PRKD2	8.12727	0.55887
PRKD3	7.31629	1.26237

PRKDC	6.05485	0.528064
PRKRA	7.59768	0.646867
PRKRIR	8.84739	0.624993
PRM2	5.47177	0.46462
PRMT3	5.6796	1.01116
PRMT5	7.32913	0.578211
PRMT7	5.99001	0.412108
PRMT9	6.32316	0.551544
PRNP	7.95972	0.791298
PRO2852	7.38822	0.899207
PRODH	5.85032	0.491324
PRORSD1P	4.8989	0.726726
PROSC	6.07345	0.402894
PROSER1	6.31355	0.666287
PRPF19	8.16802	0.478095
PRPF3	6.89373	0.611713
PRPF38A	5.95748	0.533047
PRPF38B	7.1933	0.583383
PRPF39	6.55211	0.752713
PRPF4	7.8172	0.550211
PRPF4B	7.72165	0.511268
PRPF6	6.70306	0.42458
PRPF8	8.62338	0.484497
PRPS1	7.93406	0.67541
PRPS2	6.94039	0.824501
PRPSAP2	8.37293	0.547479
PRR11	7.80274	0.648627
PRR13	8.56696	0.437839
PRR14	6.92654	0.424523
PRR15	5.9035	1.43842
PRR24	6.33727	0.453049
PRR3	5.75353	0.499875
PRR34-AS1	8.01291	1.47959
PRR5	5.02391	0.70405
PRR7	5.46768	0.56168
PRRC1	8.50254	0.529969
PRRC2C	7.92135	0.582743
PRSS16	6.12762	0.835469
PRUNE	5.78571	0.468956
PSAP	11.1489	0.577355
PSAPL1	5.30841	0.523798
PSAT1	9.03935	1.25347
PSEN2	6.61926	0.516175
PSENEN	8.15208	0.496461
PSIP1	6.72887	0.501795
PSMA1	10.4281	0.52287
PSMA2	7.04977	0.414662

PSMA4	9.37785	0.718027
PSMA6	8.96169	0.557181
PSMA7	8.60407	0.558968
PSMB10	8.78299	0.605143
PSMB3	10.2627	0.485179
PSMB4	9.90357	0.445949
PSMB5	8.83179	0.534959
PSMB6	9.32667	0.531405
PSMB7	8.24815	0.526633
PSMB8	8.84848	0.650678
PSMB8-AS1	6.69865	0.448787
PSMB9	9.56186	0.612548
PSMC1	9.13411	0.468244
PSMC2	7.68161	0.550476
PSMC3	8.82699	0.514967
PSMC3IP	5.19311	0.469416
PSMC4	8.20407	0.485487
PSMC5	9.28093	0.455763
PSMC6	8.80523	0.717791
PSMD1	8.2851	0.447157
PSMD10	8.73176	0.663409
PSMD11	7.34303	0.473822
PSMD12	7.7675	0.636206
PSMD13	7.66431	0.443866
PSMD14	9.54568	0.657007
PSMD2	8.69948	0.465648
PSMD3	7.3033	0.492913
PSMD4	8.4309	0.464305
PSMD5-AS1	5.40358	0.628676
PSMD6-AS2	5.94392	0.431814
PSMD7	8.1375	0.586984
PSMD8	9.11342	0.46033
PSME1	10.8991	0.448455
PSME2	11.2072	0.649592
PSME3	6.90866	0.482697
PSME4	6.49033	0.55312
PSMG1	7.27773	0.816275
PSMG2	9.52322	0.582125
PSMG3	7.06622	0.524522
PSPC1	5.73911	0.409251
PTAR1	6.21802	0.505578
PTBP3	6.61731	0.551887
PTCD2	6.40071	0.645633
PTCD3	6.40333	0.468048
PTCH1	4.89849	0.535915
PTDSS1	8.83721	0.623017
PTEN	6.6758	0.430014

PTENP1	8.72167	0.686615
PTER	6.82256	0.639182
PTGER4	5.11153	0.981692
PTGES3	11.1501	0.456647
PTGR2	6.01314	0.405648
PTGS1	4.90969	0.459744
PTGS2	5.68114	1.92476
PTK2B	7.42252	0.590664
PTMA	11.363	0.403433
PTOV1	7.10294	0.525091
PTP4A1	7.91352	0.534767
PTP4A2	9.61865	0.570027
PTP4A3	7.18255	1.44425
PTPLAD1	8.87786	0.607549
PTPLAD2	5.30442	0.725008
PTPLB	7.19199	0.726319
PTPMT1	6.92434	0.414339
PTPN18	5.57921	0.450427
PTPN6	8.53512	0.636643
PTPN7	6.10902	0.481795
PTPRCAP	7.40434	1.29059
PTPRG	5.28462	1.10494
PTPRK	5.46615	1.12978
PTPRM	5.14716	0.710329
PTPRN2	5.41026	0.838165
PTRH2	7.97466	0.628272
PTRHD1	8.08048	0.696642
PTS	7.91546	0.603972
PTTG1	8.42525	1.10685
PTTG1IP	9.41864	0.494273
PUF60	7.93858	0.474197
PURB	6.31206	0.400397
PUS1	6.90305	0.510779
PUS3	7.59509	0.6582
PUS7	6.77343	0.970512
PUS7L	5.79167	0.622489
PUSL1	6.20194	0.458902
PVRIG	4.84269	0.598308
PWAR6	5.83985	0.986542
PWP1	7.99662	0.450781
PXDN	5.02893	1.18327
PXK	5.39227	0.440788
PXMP2	6.08653	0.547355
PXN-AS1	5.58356	0.447983
PXYLP1	5.18535	0.440568
PYCARD	7.03515	0.77844
PYCARDOS	5.23104	0.440989

PYCR1	7.93853	0.481337
PYCR2	7.57459	0.647642
PYCRL	5.3809	0.40796
PYROXD1	6.46442	0.532842
PYROXD2	5.53416	0.454416
PYURF	8.96235	0.483837
PYY2	6.13682	0.452897
QARS	10.0963	0.584079
QKI	5.83966	0.511262
QPCT	9.94204	2.02414
QPCTL	5.86101	0.402317
QPRT	7.19054	0.757213
QRSL1	5.06507	0.473792
QSER1	6.26889	0.446565
QSOX2	6.70185	0.43929
QTRT1	7.70259	0.530046
R3HCC1	7.47336	0.443659
R3HDM2	6.96922	0.529033
RAB10	9.06891	0.485188
RAB11A	7.07942	0.530669
RAB11FIP1	6.6411	1.02543
RAB11FIP2	7.29795	0.823643
RAB11FIP5	6.17892	0.494358
RAB12	5.32779	0.408989
RAB13	7.7268	1.27603
RAB15	5.53842	0.664166
RAB1B	7.47077	0.405004
RAB22A	6.53352	0.447467
RAB24	8.52887	0.717349
RAB26	5.77495	0.700522
RAB27A	7.72894	0.79205
RAB28	6.34813	0.545234
RAB29	6.34433	0.916482
RAB2B	8.08764	0.575614
RAB30	7.16795	0.900258
RAB30-AS1	6.23881	0.773492
RAB31	6.02083	1.34244
RAB33B	4.8797	0.593743
RAB34	6.11464	1.29391
RAB36	6.55782	0.82521
RAB39B	5.37195	1.14407
RAB3A	6.20127	0.413008
RAB3B	5.0127	0.774754
RAB3D	6.61798	0.407623
RAB3GAP1	6.20711	0.408791
RAB3GAP2	6.32849	0.429906
RAB4A	6.4089	1.10102

RAB5B	6.58916	0.564827
RAB6C	9.19956	0.513371
RAB8A	9.53313	0.598274
RAB8B	6.48458	0.955111
RAB9A	8.54678	0.562698
RABAC1	10.7446	0.682467
RABEP1	5.81457	0.439854
RABEPK	5.68225	0.408367
RABGAP1L	6.47968	0.551237
RABGGTA	6.54812	0.44657
RABGGTB	8.3128	0.71341
RABIF	6.40854	0.605485
RABL2A	5.57043	0.511018
RABL2B	5.57043	0.511018
RABL3	5.46651	0.449075
RAC1	7.50175	0.475635
RAC2	9.52232	0.736899
RACGAP1	5.89146	1.29848
RAD1	6.19385	0.452693
RAD17	8.66488	0.490934
RAD21	8.61026	0.666647
RAD23A	8.58324	0.400164
RAD23B	7.70762	0.453625
RAD50	6.16315	0.508382
RAD51-AS1	6.59397	0.687048
RAD51AP1	5.32829	1.1521
RAD51C	6.56341	0.622287
RAD9A	6.48232	0.412723
RAF1	7.38417	0.475736
RALA	7.44331	0.61217
RALB	6.36808	0.507749
RALBP1	7.00264	0.47744
RALGDS	7.4656	0.469399
RALGPS2	7.42393	0.921952
RALY	7.99589	0.650362
RALY-AS1	6.20437	0.647726
RAMP2	5.22378	0.406023
RAN	9.35826	0.760213
RANBP2	7.61297	0.623461
RANBP6	7.67803	0.791761
RANBP9	6.68964	0.479472
RAP1B	10.8031	0.432101
RAP1GAP2	5.58735	1.03104
RAP1GDS1	6.25058	0.476227
RAP2A	5.32335	0.419456
RAP2B	5.21472	0.4027
RAP2C	7.57266	0.809181

RAPGEF1	6.54394	0.479129
RAPGEF2	7.5083	0.911995
RAPGEF4	4.89988	1.27079
RAPGEF6	5.81439	0.411742
RARRES3	7.28018	1.22597
RARS	8.6894	0.481131
RASA1	8.40081	0.642195
RASA2	8.67941	0.634324
RASA3	5.7132	0.409633
RASA4	6.0675	0.70025
RASA4B	6.0675	0.70025
RASA4CP	6.3396	0.623429
RASAL3	6.97088	0.503549
RASD1	7.40011	1.17701
RASGRP1	6.24206	2.35821
RASGRP3	6.06235	1.03916
RASSF1-AS1	5.65231	0.413804
RASSF2	6.03172	0.91937
RASSF3	5.07043	0.695506
RASSF5	8.02234	0.713188
RASSF6	6.3003	1.8951
RAVER1	6.48756	0.416298
RB1	6.68409	0.929488
RB1CC1	8.18083	0.633915
RBBP4	7.36775	0.519859
RBBP5	6.19988	0.768273
RBBP7	9.18012	0.513213
RBBP8	7.63069	0.830534
RBCK1	7.24981	0.436704
RBFOX2	6.43425	0.499564
RBKS	6.63513	0.511095
RBL2	7.65109	0.583883
RBM12	6.90037	0.573396
RBM12B	5.21393	0.674781
RBM14	6.25617	0.437676
RBM18	6.43287	0.520743
RBM22	8.15201	0.465869
RBM23	6.96702	0.490842
RBM25	7.61695	0.665675
RBM27	7.6922	0.406529
RBM3	6.82276	0.554762
RBM33	5.55939	0.476458
RBM34	5.97921	0.53902
RBM38	6.55462	0.707923
RBM39	8.80493	0.503902
RBM41	5.9273	0.63877
RBM42	6.73892	0.517446

RBM47	6.53577	0.444128
RBM48	5.4846	0.446909
RBM4B	5.77776	0.659486
RBM5	7.02696	0.481708
RBM6	6.94392	0.480385
RBM7	6.2212	0.670384
RBM8A	6.81475	0.48036
RBMS1	8.58919	0.810549
RBMX2	6.38378	0.542449
RBMXL1	6.40148	0.749312
RBP7	5.02459	0.512625
RBPJ	7.07416	0.496077
RBX1	9.12195	0.503785
RC3H1	6.06402	0.629237
RC3H2	6.12498	0.422811
RCBTB2	5.94573	0.734179
RCC2	7.98067	0.64457
RCN1	7.95824	0.659116
RCN2	9.11606	0.501723
RCN3	5.74382	0.587543
RCOR1	5.74523	0.421837
RCOR3	6.03011	0.622865
RCSD1	7.54795	1.0012
RDH13	5.59926	0.488807
RDH14	8.10177	0.605981
RDX	6.00752	0.499514
REC114	5.31796	0.583784
RECQL	7.34532	0.726733
REEP2	6.58121	0.40125
REEP3	5.4894	0.694595
REEP5	9.77922	0.604571
RELA	7.28087	0.43602
RELB	6.24809	0.62619
RELL1	5.28089	0.623134
RELL2	6.53333	0.468151
RELN	6.19405	1.75584
REM2	5.66677	0.48468
REPS1	5.78953	0.411797
RETN	5.34714	0.713626
RETSAT	6.20902	0.447141
REV3L	6.31055	0.742708
REXO2	6.48281	0.511519
RFC1	7.58327	0.49156
RFC2	5.57769	0.545223
RFC4	7.458	0.888626
RFC5	5.0704	0.720287
RFESD	5.01008	0.566513

RFK	7.09729	0.66785
RFNG	5.87504	0.414233
RFWD2	6.74326	0.552042
RFX5	7.92146	0.522906
RFX7	5.86232	0.779251
RFXANK	8.13516	0.469613
RGCC	6.07764	1.14103
RGL1	6.1871	1.05259
RGL2	7.23887	0.496178
RGPD1	6.72709	0.894293
RGPD2	6.72709	0.894293
RGPD3	6.89664	0.775231
RGPD4	6.89664	0.775231
RGPD5	6.89664	0.775231
RGPD6	6.89664	0.775231
RGPD8	6.89664	0.775231
RGS1	7.37269	1.32478
RGS16	5.37297	0.653591
RGS18	5.3028	1.52322
RGS19	7.99539	0.499939
RGS2	10.5596	1.51616
RHBDD2	7.40511	0.551944
RHBDF2	6.93718	0.47942
RHEBL1	5.39744	0.47925
RHNO1	5.91695	0.656864
RHOB	6.22661	0.572709
RHOBTB2	5.48644	0.401741
RHOBTB3	5.2239	0.685718
RHOC	5.02751	0.403458
RHOG	6.97761	0.506424
RHOH	8.58314	1.21033
RHOQ	8.84674	0.758402
RHOV	5.47773	0.439139
RHPN1	6.5469	0.463914
RHPN2	4.83197	0.740963
RIC3	5.59842	0.575029
RICTOR	6.96568	0.742169
RILP	6.76655	0.413201
RILPL2	7.02729	0.736864
RIN1	6.46233	0.414518
RINT1	7.13129	0.552821
RIOK1	6.7844	0.67908
RIOK2	6.13664	0.72396
RIPK2	6.42857	0.439166
RIT1	5.36295	0.562863
RLF	6.08549	0.588164
RMDN1	5.71349	0.451601

RMDN3	7.99905	0.48111
RMI1	5.9992	0.982783
RMI2	5.28398	0.704555
RMND1	5.57543	0.629585
RMND5A	6.28193	0.418388
RNA45S5	6.62231	0.774795
RNASE2	6.29129	1.51405
RNASE3	6.28513	1.30752
RNASE4	5.88971	1.33098
RNASE6	6.04701	2.20692
RNASEH1	7.08023	0.449006
RNASEH1-AS1	4.83824	0.47576
RNASEH2A	7.17832	0.795654
RNASEK	10.507	0.561856
RNASEK-C17orf49	9.16864	0.564148
RNASEL	5.12555	0.501023
RND1	6.35483	0.555286
RND3	6.38096	2.57587
RNF10	7.05459	0.467095
RNF103	7.82589	0.414332
RNF103-CHMP3	7.75013	0.519004
RNF11	8.59942	0.964008
RNF111	6.97436	0.753269
RNF113A	8.57865	0.588754
RNF114	7.95818	0.436007
RNF115	6.61078	0.405266
RNF123	6.54086	0.411622
RNF126	6.70153	0.40145
RNF13	9.56986	0.447725
RNF135	7.70554	0.730706
RNF138	6.28232	0.579683
RNF139	8.57374	0.551609
RNF139-AS1	5.23656	0.707528
RNF14	5.75792	0.530802
RNF145	6.04995	0.468274
RNF146	6.52868	0.713506
RNF148	4.90626	0.752831
RNF149	8.19807	0.818316
RNF157	5.11286	0.531795
RNF168	5.6877	0.538263
RNF169	6.55163	0.536074
RNF181	9.53539	0.453355
RNF185	7.20594	0.441111
RNF19A	7.58966	0.602967
RNF19B	4.80467	0.473705
RNF20	8.34657	0.652959
RNF213	6.15196	0.53858

RNF38	7.09478	0.543987
RNF4	8.60257	0.430121
RNF44	6.95933	0.499527
RNF5P1	7.70542	0.621661
RNFT1	5.61442	0.604206
RNGTT	7.10757	0.939425
RNMT	5.80611	0.426592
RNMTL1	6.69786	0.492127
RNPC3	6.86525	0.620616
RNPEP	7.66763	0.627097
ROBO1	4.88099	1.62938
ROBO3	5.04692	0.550448
ROCK2	6.08463	0.480221
ROGDI	5.09979	0.462866
ROMO1	8.72919	0.497149
RP1-130G2.1	5.71914	0.531501
RP1-151F17.2	6.89871	0.926682
RP1-170O19.17	4.99053	0.439527
RP1-30M3.5	5.44265	0.589935
RP1-39G22.7	6.35723	0.713906
RP1-80B9.2	5.01371	0.441022
RP11-108M9.4	7.59733	0.740596
RP11-1094M14.11	5.56085	0.570538
RP11-111M22.3	7.07742	0.814707
RP11-111M22.4	5.12923	0.592667
RP11-1191J2.5	5.00664	0.430322
RP11-119F7.5	5.09805	0.838468
RP11-11N9.4	5.13685	1.1988
RP11-121C2.2	5.74977	0.647751
RP11-124L9.5	5.32477	1.19873
RP11-1277A3.3	5.03685	0.421078
RP11-134L10.1	5.13932	0.492247
RP11-138A9.1	8.66341	1.22327
RP11-174G6.5	5.68216	0.668818
RP11-182L21.5	5.77017	0.564931
RP11-190A12.8	5.29478	0.538262
RP11-196G18.24	6.22233	1.03798
RP11-199F11.2	6.23538	0.821322
RP11-209A2.1	10.8107	0.505698
RP11-212P7.2	5.18045	0.49051
RP11-226L15.5	5.33174	0.888287
RP11-258C19.7	5.17812	0.468375
RP11-271C24.3	7.53537	0.954767
RP11-284F21.10	5.52274	0.60478
RP11-285F7.2	6.48068	0.526687
RP11-28F1.2	7.43142	1.09822
RP11-295G20.2	4.98387	0.48289

RP11-301O19.1	4.83073	0.415149
RP11-305K5.1	7.31546	0.701756
RP11-326I11.3	5.03486	0.578618
RP11-326I11.5	5.22181	0.693444
RP11-332H14.2	6.10602	0.526361
RP11-339B21.15	5.50398	0.471941
RP11-348P10.2	5.04598	0.573832
RP11-355B11.2	5.27671	0.677448
RP11-390E23.6	5.46963	0.443827
RP11-391M1.4	6.08434	0.874106
RP11-395B7.2	5.87523	0.463163
RP11-395B7.7	6.33995	0.548152
RP11-403P17.3	6.87893	0.474142
RP11-403P17.4	8.96146	0.531017
RP11-410L14.2	6.5173	0.57923
RP11-426C22.5	5.39714	0.401145
RP11-429B14.4	4.87458	0.409784
RP11-432J9.6	5.05849	0.434405
RP11-436D10.3	5.12874	0.431193
RP11-464F9.20	5.59187	0.499915
RP11-469M7.1	5.94118	0.677768
RP11-473I1.9	6.28366	0.494445
RP11-480A16.1	6.43219	0.484939
RP11-488L18.10	7.20834	0.828995
RP11-489E7.4	6.67207	0.794666
RP11-500C11.3	5.09966	0.451213
RP11-50B3.4	6.6128	0.635848
RP11-521D12.1	4.93851	0.410811
RP11-522D2.1	4.90834	0.919506
RP11-532F12.5	6.10293	0.86047
RP11-533E19.7	5.32908	0.627555
RP11-539L10.3	4.99306	0.514594
RP11-53O19.3	5.00682	0.51835
RP11-554J4.1	6.2636	0.847518
RP11-568N6.1	7.20585	1.18871
RP11-589P10.5	5.12809	0.435952
RP11-613M5.1	5.53205	0.635011
RP11-617F23.1	6.15721	0.66621
RP11-680F8.4	4.90634	0.865572
RP11-686D22.8	4.83437	0.48851
RP11-727A23.11	4.85552	0.539759
RP11-769O8.3	6.14913	0.885336
RP11-792A8.4	4.93237	0.49695
RP11-796E2.4	6.52276	0.55517
RP11-843B15.2	5.86985	0.402252
RP11-846E15.2	7.48293	0.845999
RP11-97C16.1	6.28912	0.995058

RP13-20L14.1	4.94473	0.489194
RP13-258O15.1	12.1453	0.433302
RP13-270P17.3	5.17534	0.558886
RP3-334F4.1	6.5307	0.438954
RP3-486D24.1	11.3524	0.57389
RP3-507I15.1	10.9001	0.528275
RP3-508I15.21	6.57349	0.49985
RP3-525N10.2	6.04209	1.55422
RP4-545K15.5	5.27773	0.538239
RP4-555D20.2	5.84182	1.45981
RP4-595K12.1	11.5148	0.593624
RP4-635E18.8	7.07235	0.581243
RP4-710M16.1	7.4249	0.497586
RP4-758J24.5	5.65225	0.600784
RP4-773N10.4	5.13883	0.45781
RP4-781L3.1	8.21091	0.789391
RP4-798A10.7	5.24746	0.552486
RP5-1092A3.4	5.88387	1.12156
RP5-1136G13.2	5.39328	0.746774
RP5-1157M23.2	5.99317	0.48171
RP5-882O7.1	13.2265	0.40142
RP5-894A10.6	5.04963	0.408926
RP5-930J4.4	6.18666	0.489011
RP5-935K16.1	5.7698	0.619958
RPA1	6.43676	0.477993
RPA2	7.43626	0.673445
RPA3	8.64029	0.725495
RPA3OS	6.56169	0.768681
RPAP1	6.30618	0.423144
RPAP2	6.32267	0.446779
RPAP3	6.22813	0.66933
RPE	5.44165	0.462391
RPEL1	5.51244	0.508452
RPF2	8.1691	0.712563
RPH3A	5.81194	1.17669
RPIA	7.42702	0.722316
RPL10L	5.82778	0.562662
RPL14	10.7238	0.447424
RPL15	9.90508	0.41735
RPL18A	9.37195	0.463133
RPL18AP16	8.39517	0.663909
RPL18P10	5.96665	0.448781
RPL22	11.6127	0.447855
RPL22L1	9.52471	0.949579
RPL23	9.06808	0.480086
RPL26L1	9.09651	0.534844
RPL26P37	12.1453	0.433302

RPL27AP	6.8042	0.557556
RPL28	10.0048	0.44455
RPL29	13.2057	0.474322
RPL29P7	6.21806	0.773712
RPL31	8.07246	0.601471
RPL35P8	6.12106	0.559904
RPL36	11.8932	0.717759
RPL36A	10.0027	0.465963
RPL36A-HNRNPH2	10.7468	0.533665
RPL36AL	11.7725	0.623673
RPL37	8.08864	0.623811
RPL38	10.3423	0.437705
RPL39L	6.67839	0.776368
RPL4	13.1731	0.415466
RPL7AL2	6.92313	0.560563
RPL7AP10	7.95575	0.723232
RPL7AP71	5.50804	0.426761
RPL7L1	8.75252	0.520422
RPN1	10.9661	0.536865
RPN2	12.2109	0.554779
RPP21	7.68922	0.423906
RPP25	6.59002	0.618981
RPP25L	8.30813	0.490258
RPP38	6.9187	0.502679
RPP40	6.90086	0.912408
RPPH1	7.6657	0.889235
RPRD1A	6.76049	0.567734
RPRD2	6.34773	0.417152
RPRM	4.87606	0.758614
RPS10-NUDT3	7.06858	0.525702
RPS10L	9.52566	0.497391
RPS10P2	6.45824	0.704717
RPS16P5	6.94241	1.04109
RPS17P5	8.91126	0.669895
RPS19BP1	8.25095	0.432553
RPS2	12.0635	0.644486
RPS21	10.44	0.47309
RPS23	10.698	0.619529
RPS24	8.86598	0.450728
RPS26	11.8815	0.696551
RPS26P11	11.8815	0.696551
RPS27	10.6163	0.619756
RPS27L	8.77963	0.491236
RPS3	13.0105	0.411269
RPS4XP2	12.29	0.495006
RPS4XP3	5.54011	0.61397
RPS4Y1	8.72175	2.7538

RPS6KA1	5.43646	0.515372
RPS6KA2	5.41717	0.542808
RPS6KA3	6.40351	0.604936
RPS6KA5	4.8984	0.495945
RPS6KB1	7.03891	0.450826
RPS6KC1	6.33289	0.869805
RPS6KL1	5.53653	0.401623
RPSA	13.2859	0.42065
RPSAP19	13.2859	0.42065
RPSAP58	13.2859	0.42065
RPSAP9	13.2859	0.42065
RPUSD2	5.45216	0.465369
RPUSD4	7.46392	0.683246
RRAGA	8.32741	0.597989
RRAGD	8.85466	1.00344
RRAS	5.01807	0.613936
RRAS2	4.946	1.79621
RRBP1	6.97918	0.658667
RRM1	7.32219	0.760667
RRM2	7.75502	1.8736
RRM2B	5.95327	0.777113
RRN3	6.3614	0.484408
RRN3P2	5.53219	0.44959
RRNAD1	6.41342	0.449232
RRP15	5.91528	0.757
RRP1B	7.38808	0.445233
RRP9	6.056	0.462348
RRS1	8.13916	0.644362
RSAD1	7.28783	0.468823
RSAD2	5.92961	1.76458
RSBN1	6.42757	0.666982
RSBN1L	6.26643	0.472339
RSF1	6.25472	0.623529
RSL1D1	7.25923	0.670981
RSL24D1	10.2776	0.707798
RSPH1	5.02324	0.63522
RSPRY1	6.76024	0.566366
RSRC1	5.34423	0.548855
RSRC2	8.78857	0.411154
RSRP1	8.87125	0.666845
RTBDN	5.00953	0.41085
RTCA	6.42333	0.458519
RTCB	8.02747	0.617562
RTN2	5.3399	0.585144
RTN3	8.96188	0.642617
RTN4	9.24495	0.663147
RTN4IP1	5.24198	0.54123

RTP4	6.0374	1.11654
RTP5	5.12589	0.417078
RUFY3	6.35346	0.923585
RUNX1-IT1	6.11084	1.16081
RUNX3	6.49529	0.540432
RUSC1	6.62527	0.608972
RUSC1-AS1	5.31833	0.52158
RUVBL1	7.51963	0.812367
RWDD1	6.91186	0.404201
RWDD2A	6.00402	0.514477
RWDD2B	6.97637	0.527528
RWDD3	6.81564	0.615868
RWDD4	6.95536	0.581565
RXRA	6.05362	0.444703
RYBP	7.56104	0.517433
RYK	6.76545	0.56655
S100A10	6.73855	1.12457
S100A11	7.48406	1.15035
S100A11P1	6.43016	0.752001
S100A12	6.83922	2.04425
S100A4	8.38558	1.85232
S100A6	6.06753	0.806321
S100A8	7.39348	1.62626
S100A9	8.30298	1.58593
S100P	5.16437	1.14835
S100PBP	7.179	0.728184
S100Z	4.80002	1.05455
S1PR4	7.25558	0.596464
SAAL1	7.11656	0.626527
SAC3D1	6.333	0.795895
SACM1L	8.49351	0.775402
SAE1	6.44342	0.411546
SALL2	5.03346	0.51066
SALL4	5.17665	0.463335
SAMD11	6.43296	0.493615
SAMD12	5.49101	0.521565
SAMD8	5.09951	0.443223
SAMD9	6.02106	1.04263
SAMD9L	7.31086	1.10344
SAMHD1	6.23515	0.608961
SAMSN1	5.85783	1.33285
SAP130	6.31888	0.540209
SAP18	7.4847	0.486509
SAPCD1	5.25198	0.484336
SAPCD1-AS1	5.13735	0.437848
SAPCD2	6.69987	0.477532
SAR1A	7.60697	0.492576

SAR1B	7.76017	0.48
SARAF	10.8831	0.666703
SARS	7.31387	0.733246
SARS2	5.28281	0.428522
SART3	6.17486	0.40831
SASH3	7.36577	1.12714
SAT1	8.44587	0.703997
SAT2	8.152	0.509646
SATB1	5.3196	1.21868
SAV1	5.62944	0.444013
SBDS	8.95995	0.617902
SBDSP1	8.95995	0.617902
SBF2	5.26684	0.534838
SC5D	5.45125	0.516289
SCAF11	7.14968	0.405276
SCAF4	5.75165	0.567802
SCAF8	7.57615	0.828632
SCAMP1	6.72431	0.515194
SCAMP1-AS1	6.2426	0.647299
SCAMP3	8.61057	0.567998
SCAMP5	6.57912	0.414996
SCAND1	8.10462	0.490336
SCAP	7.1805	0.441911
SCAPER	6.8549	0.758338
SCARB2	7.70729	0.711259
SCARNA15	6.94691	0.600085
SCARNA16	5.28603	0.687087
SCARNA17	5.74237	0.622821
SCCPDH	5.50617	0.916474
SCD	5.39506	0.590466
SCD5	4.8567	0.456287
SCFD1	7.06877	0.524816
SCFD2	6.88288	0.464564
SCGB3A1	5.6097	0.420981
SCN3A	5.18117	2.03028
SCNM1	8.15987	0.50158
SCNN1B	5.97627	0.993951
SCO1	6.54007	0.587909
SCO2	7.595	0.685305
SCOC	7.54464	0.635022
SCP2	8.7621	0.712613
SCPEP1	6.10282	0.5605
SCRIB	7.01929	0.447564
SCRN1	7.71694	0.791441
SCT	5.26876	0.671426
SCYL2	8.32596	1.28021
SCYL3	5.48363	0.53807

SDAD1	6.2675	0.438918
SDC1	11.0832	0.693309
SDCBP	8.13729	1.23313
SDE2	7.25807	0.764122
SDF2	7.48715	0.471681
SDF2L1	9.64147	0.660178
SDF4	8.90704	0.509799
SDHA	8.16521	0.585534
SDHAF2	7.03723	0.541862
SDHAP1	7.61524	0.464088
SDHAP2	7.61524	0.464088
SDHC	6.58121	0.479924
SDR39U1	8.32585	0.488088
SDSL	5.00316	0.489534
SEC11A	10.4139	0.544664
SEC11C	9.15214	0.680221
SEC13	10.0952	0.42071
SEC14L1	8.25311	0.431814
SEC14L1P1	5.31836	0.522338
SEC16A	8.20432	0.493905
SEC22B	8.56556	0.56328
SEC23A	6.99553	0.549359
SEC23B	8.6185	0.558717
SEC23IP	5.48592	0.428671
SEC24A	7.63654	0.676521
SEC24B	8.23117	0.552992
SEC24C	7.70812	0.418913
SEC24D	6.31502	0.740174
SEC31A	9.64034	0.56723
SEC31B	6.31417	0.53169
SEC61A1	10.1457	0.436079
SEC61B	9.74458	0.61113
SEC61G	10.6761	0.52461
SEC62	9.34544	0.711742
SEC63	7.1143	0.581588
SECISBP2	8.74316	0.412721
SECISBP2L	7.64349	0.905773
SECTM1	5.0892	0.802642
SEH1L	6.75223	0.787192
SEL1L	9.24232	0.770521
SEL1L3	7.75935	0.885987
SELENBP1	5.10488	0.570713
SELK	11.2788	0.451788
SELL	5.97011	1.88713
SELM	9.45493	1.05526
SELPLG	7.51104	0.828316
SELT	9.92085	0.53144

SEMA4A	7.71495	0.839634
SEMA4B	6.96826	0.591037
SEMA4D	6.20238	0.736229
SENCR	6.35231	0.565592
SENP3-EIF4A1	9.71863	0.462955
SENP6	5.97506	0.582925
SENP7	4.93503	0.466047
15-Sep	10.2823	0.634534
SEPHS1	6.59954	0.548519
SEPHS2	7.48218	0.736632
SEPN1	5.95731	0.437061
SEPP1	6.03635	0.997552
1-Sep	5.74042	0.832615
10-Sep	7.35874	1.84011
3-Sep	5.02284	0.474777
5-Sep	5.01982	0.44952
SEPT5-GP1BB	5.48274	0.40636
6-Sep	6.79132	0.963592
8-Sep	5.35914	0.587316
9-Sep	6.41819	0.423206
SEPW1	8.43366	0.498238
SERBP1	8.63461	0.58121
SERF1A	6.63545	0.511953
SERF1B	6.63545	0.511953
SERF2-C15ORF63	8.8786	0.521978
SERINC1	9.97905	0.64267
SERINC3	8.81345	0.417717
SERP1	9.97078	0.413115
SERPINB1	6.5954	0.68761
SERPINB9	5.11018	1.06556
SERPINB9P1	4.92629	0.559481
SERPINH1	5.50781	0.493314
SERPINI1	7.78974	1.49047
SERTAD1	6.57788	0.556599
SERTAD2	8.07356	0.750629
SERTAD3	6.43761	0.513962
SESN1	7.36384	0.902237
SESN2	6.86792	0.928363
SET	10.5174	0.442537
SETBP1	7.54265	0.952559
SETD2	5.90394	0.509254
SETD6	6.71779	0.575265
SETD7	6.14384	0.519012
SETD9	5.5345	0.507421
SETP4	9.67876	0.898185
SETSIP	10.4298	0.442628
SETX	7.08574	0.601768

SF1	6.34458	0.528793
SF3A2	6.05097	0.575437
SF3A3	6.36178	0.57511
SF3B1	9.10052	0.405284
SF3B4	7.60015	0.743885
SF3B5	10.0347	0.606935
SFMBT2	6.37169	0.490442
SFN	5.83189	0.531212
SFR1	5.38502	0.709385
SFT2D2	6.64389	0.537793
SFXN1	5.63013	0.533337
SFXN3	5.58767	0.445166
SFXN4	7.15835	0.411118
SGK1	8.55374	1.37654
SGK3	6.11444	0.671584
SGK494	4.83347	0.568404
SGMS1	4.97752	0.712504
SGSH	6.02566	0.479381
SH2B2	8.10143	0.791864
SH2B3	5.26015	0.753531
SH2D2A	5.18868	0.487753
SH3BGR	5.18136	0.567564
SH3BGRL	9.42108	0.595836
SH3BGRL2	6.21706	1.19096
SH3BGRL3	8.20906	0.545579
SH3BP5	7.76476	1.24151
SH3BP5-AS1	6.24504	0.470367
SH3D21	5.70019	0.527273
SH3GLB1	8.60997	0.560853
SH3KBP1	9.25901	0.497454
SH3TC1	4.95795	0.464022
SH3YL1	6.39784	0.416498
SHC1	8.27349	0.701108
SHCBP1	5.82612	1.38542
SHFM1	9.61842	0.466117
SHISA2	4.89174	1.97443
SHISA5	8.57113	0.445941
SHMT2	7.91989	0.897811
SHOC2	7.67873	0.661907
SHPRH	5.34928	0.955167
SHQ1	6.76249	0.587332
SIAH1	5.37716	0.477551
SIAH2	7.44775	0.480406
SIDT1	7.36366	1.02108
SIDT2	7.24839	0.423786
SIGIRR	6.54685	0.780985
SIGMAR1	7.83195	0.651414

SIK1	8.70743	0.710512
SIK3	6.01152	0.424854
SIKE1	5.02274	0.539153
SIL1	9.07463	0.552203
SIMC1	5.42707	1.00556
SIPA1	5.69434	0.440931
SIRPA	4.81522	0.417542
SIRT1	6.75657	0.715633
SIT1	6.48665	0.795945
SIVA1	6.85859	0.402241
SKA2	6.36004	0.964656
SKAP1	6.03199	0.906718
SKAP2	5.71391	0.526594
SKIL	5.63416	0.429284
SKIV2L2	6.62139	0.413966
SKP1	9.41295	0.450292
SKP2	5.38882	0.619925
SLA	5.66601	0.899276
SLAIN1	5.88335	0.873821
SLAIN2	5.60486	0.443329
SLAMF1	7.02543	1.68321
SLAMF6	6.41907	0.772507
SLAMF7	10.9643	0.686401
SLBP	8.00675	0.752123
SLC10A3	7.25611	0.625883
SLC12A2	5.68287	0.639857
SLC12A6	6.38817	0.661091
SLC12A8	5.04868	0.810562
SLC12A9	5.99677	0.408547
SLC15A2	6.83451	0.971877
SLC15A3	5.267	0.657663
SLC16A1	5.59041	0.608078
SLC16A14	6.81846	1.76711
SLC16A3	5.77719	0.517674
SLC16A6	6.30894	1.10838
SLC16A7	6.13985	0.914228
SLC17A5	6.70554	0.683977
SLC17A9	6.71833	0.611034
SLC18B1	5.43296	0.958247
SLC19A2	4.83628	0.915779
SLC1A4	8.46569	0.55993
SLC1A5	8.20089	0.643654
SLC20A1	7.87506	0.804649
SLC22A15	5.14119	0.953861
SLC22A17	4.95265	0.499901
SLC22A18	6.82254	0.62289
SLC22A31	6.53567	0.630106

SLC25A1	5.70561	0.516716
SLC25A11	6.5523	0.486607
SLC25A12	6.51412	0.549959
SLC25A14	7.02205	0.517927
SLC25A19	7.38525	0.474281
SLC25A20	7.49741	0.767813
SLC25A22	6.58184	0.423025
SLC25A23	6.42427	0.476885
SLC25A25	6.28603	0.439401
SLC25A26	7.30686	0.455766
SLC25A3	8.30327	0.42495
SLC25A32	8.055	0.687529
SLC25A33	7.04059	0.687482
SLC25A36	7.77093	0.653615
SLC25A37	4.99788	0.539105
SLC25A38	7.93419	0.484242
SLC25A39	7.78563	0.527647
SLC25A40	6.37922	0.49268
SLC25A43	4.98711	1.06008
SLC25A44	6.45473	0.457783
SLC25A45	5.54608	0.477119
SLC25A46	8.69048	0.628381
SLC25A5	11.0337	0.618688
SLC25A6	11.2819	0.622145
SLC26A11	7.34559	0.530797
SLC26A2	6.76756	0.631791
SLC26A6	5.7382	0.457053
SLC27A3	6.40318	0.907865
SLC29A1	5.86405	0.491245
SLC2A10	4.88664	1.60255
SLC2A11	5.98564	0.447266
SLC2A5	5.72719	0.98268
SLC2A6	5.62276	0.462965
SLC30A1	6.33346	0.52954
SLC30A5	6.68023	0.517651
SLC30A7	7.28284	0.619866
SLC30A9	6.59063	0.514483
SLC31A1	5.88811	0.401826
SLC31A2	6.56588	0.706429
SLC33A1	7.49008	0.580486
SLC35A1	8.07056	0.660978
SLC35A2	6.01073	0.415434
SLC35A3	6.67489	0.643091
SLC35A4	7.87728	0.458224
SLC35A5	8.43739	0.670029
SLC35B1	9.43601	0.625169
SLC35B2	7.38075	0.49095

SLC35B3	6.84964	0.516357
SLC35B4	5.77253	0.626753
SLC35D1	5.98278	0.464674
SLC35D2	5.32798	0.524179
SLC35E2	7.07155	0.507128
SLC35E2B	7.07155	0.507128
SLC35F1	5.70502	0.907068
SLC35F2	7.24605	0.888136
SLC35G2	4.99662	0.789143
SLC37A3	5.63991	0.647821
SLC38A1	7.25602	0.510733
SLC38A2	10.5377	0.714519
SLC38A5	7.23656	0.853169
SLC38A9	6.07743	0.53737
SLC39A1	5.92733	0.450315
SLC39A10	5.97407	0.92099
SLC39A11	6.01372	0.520708
SLC39A14	5.80884	0.438289
SLC39A4	6.65156	1.02961
SLC39A6	7.61703	0.698654
SLC39A7	7.27608	0.647075
SLC39A8	6.19707	0.489541
SLC3A2	8.66652	0.685248
SLC40A1	5.92982	1.05535
SLC41A1	6.72026	0.50891
SLC41A2	5.39621	0.62568
SLC41A3	7.02744	0.458487
SLC43A1	6.14911	0.702373
SLC44A1	8.07978	0.760964
SLC46A3	5.27945	0.988881
SLC47A1	6.4378	1.79677
SLC4A1	4.87318	0.497352
SLC4A1AP	6.82776	0.481561
SLC4A2	6.37924	0.435821
SLC4A3	4.93607	0.427814
SLC50A1	7.53247	0.675932
SLC52A2	7.06937	0.577047
SLC5A11	5.17623	0.410085
SLC5A3	5.52466	0.530999
SLC5A6	7.06999	0.52803
SLC6A9	4.84197	0.539513
SLC7A1	6.08105	0.42222
SLC7A11	4.85078	0.894761
SLC7A5	7.44116	1.2376
SLC7A5P1	5.36874	0.4194
SLC7A6	6.20655	0.429548
SLC7A6OS	5.6143	0.431898

SLC7A7	8.09865	1.09964
SLC8A3	4.8542	0.483705
SLC9A3R1	7.90915	0.54868
SLC9A6	7.22806	0.785582
SLC9A8	5.61535	0.495685
SLC9A9	5.71897	0.564478
SLC9B2	5.81052	0.677711
SLCO3A1	5.39937	0.886385
SLCO4A1	5.62947	0.50654
SLCO5A1	5.48344	0.89821
SLFN11	6.45508	1.08521
SLFN13	5.3354	0.701271
SLFN5	5.62039	0.859515
SLIRP	9.15132	0.662853
SLK	6.17607	0.684917
SLMO2	6.90931	0.542956
SLPI	5.52913	0.536165
SLTM	8.69184	0.490316
SLU7	7.94934	0.517028
SLX1B-SULT1A4	7.52912	0.47243
SLX4IP	5.68432	0.540691
SMA4	6.12568	0.855268
SMA5	5.87095	1.09868
SMAD1	6.08965	2.00704
SMAD2	6.23903	0.422976
SMAD3	5.48356	0.48043
SMAD5	6.60166	0.637117
SMAD7	5.90977	1.17563
SMAGP	5.86428	0.912712
SMAP1	8.37974	0.678451
SMAP2	8.19327	1.1897
SMARCA5	6.8402	0.452102
SMARCAD1	7.22946	0.628293
SMARCAL1	6.26753	0.425574
SMARCC1	6.98913	0.519064
SMC2	5.861	0.896403
SMC3	6.36734	0.61845
SMC4	7.40867	0.778579
SMC5	5.38128	0.468392
SMC6	6.16063	0.521928
SMCHD1	6.42975	0.622334
SMCO4	6.712	1.09213
SMEK1	6.86432	0.580335
SMG1	6.20894	0.650432
SMG1P1	5.90619	0.661313
SMG1P2	7.27906	0.652523
SMG1P3	5.58748	0.517352

SMG1P5	7.34549	0.615271
SMG1P7	5.98226	0.909193
SMG7	6.07277	0.459197
SMG8	7.33377	0.74569
SMIM1	5.87364	0.453799
SMIM13	4.84803	0.474796
SMIM14	5.57093	0.837831
SMIM15	9.07202	0.584472
SMIM19	8.99331	0.577763
SMIM20	8.29127	0.530745
SMIM24	5.00516	0.417725
SMN1	6.45094	0.79348
SMN2	6.45094	0.79348
SMNDC1	8.01758	0.597473
SMOC1	7.02986	0.737162
SMOX	5.75335	0.443995
SMPD2	6.42131	0.433895
SMPD3	4.96817	0.41189
SMPD4	6.54243	0.478868
SMPDL3B	6.17974	0.638551
SMS	5.94773	0.640694
SMYD3	7.26962	1.07656
SNAI1	5.1934	0.544914
SNAP23	6.56113	0.540817
SNAP29	6.13722	0.420755
SNAP47	7.65759	0.630506
SNAPC1	5.77225	0.637268
SNAPC3	5.0811	0.497422
SNAPIN	8.28036	0.633065
SNCA	5.25953	0.499672
SND1-IT1	7.37224	0.75814
SNF8	8.27262	0.415293
SNHG1	10.3579	0.744911
SNHG12	6.98337	0.565306
SNHG15	7.70465	0.577097
SNHG16	8.77841	0.714813
SNHG17	6.44868	0.540816
SNHG18	5.20699	0.504328
SNHG19	8.22586	1.11893
SNHG20	5.28603	0.687087
SNHG5	11.4678	1.08264
SNHG6	11.5802	0.521396
SNHG7	6.36968	0.485909
SNHG8	9.22646	0.708583
SNHG9	6.1513	0.813622
SNIP1	5.66073	0.441534
SNN	5.38004	0.65571

SNORA1	8.83222	0.80499
SNORA11D	5.37218	1.05267
SNORA11E	5.37218	1.05267
SNORA16A	6.98337	0.565306
SNORA17	6.36968	0.485909
SNORA18	8.83222	0.80499
SNORA21	7.69348	0.432615
SNORA24	9.22646	0.708583
SNORA28	8.73188	1.08578
SNORA29	6.99147	0.557052
SNORA32	8.83222	0.80499
SNORA4	10.3277	0.543255
SNORA40	8.83222	0.80499
SNORA44	6.98337	0.565306
SNORA48	10.5157	0.552191
SNORA56	8.43967	0.685712
SNORA5B	6.62495	0.489659
SNORA6	13.2859	0.42065
SNORA61	6.98337	0.565306
SNORA62	13.2859	0.42065
SNORA63	10.3277	0.543255
SNORA64	12.0635	0.644486
SNORA67	9.14369	0.419904
SNORA72	4.84381	0.567726
SNORA76C	8.57204	0.702111
SNORA78	6.30758	0.495781
SNORA8	8.83222	0.80499
SNORA81	10.3277	0.543255
SNORA9	7.70465	0.577097
SNORD10	10.5157	0.552191
SNORD104	8.57204	0.702111
SNORD110	7.98352	0.525796
SNORD16	13.1731	0.415466
SNORD18A	13.1731	0.415466
SNORD18B	13.1731	0.415466
SNORD18C	13.1731	0.415466
SNORD19B	9.50692	0.591487
SNORD1A	8.77841	0.714813
SNORD1C	8.77841	0.714813
SNORD2	10.3277	0.543255
SNORD22	10.3579	0.744911
SNORD23	11.0444	0.701814
SNORD25	10.3579	0.744911
SNORD26	10.3579	0.744911
SNORD27	10.3579	0.744911
SNORD28	10.3579	0.744911
SNORD29	10.3579	0.744911

SNORD30	10.3579	0.744911
SNORD31	10.3579	0.744911
SNORD38B	12.5469	0.407493
SNORD3A	10.7878	1.33264
SNORD3B-1	10.7878	1.33264
SNORD3B-2	10.7878	1.33264
SNORD3C	10.7878	1.33264
SNORD3D	10.7878	1.33264
SNORD44	9.99816	0.764737
SNORD45A	8.24294	0.819725
SNORD45B	8.24294	0.819725
SNORD45C	8.24294	0.819725
SNORD47	9.99816	0.764737
SNORD4A	8.45735	0.608232
SNORD5	8.83222	0.80499
SNORD50A	9.66519	0.800465
SNORD50B	9.66519	0.800465
SNORD55	12.5469	0.407493
SNORD57	7.98352	0.525796
SNORD74	9.99816	0.764737
SNORD76	9.99816	0.764737
SNORD77	9.99816	0.764737
SNORD79	9.99816	0.764737
SNORD80	9.99816	0.764737
SNORD81	9.99816	0.764737
SNORD84	7.57275	0.550332
SNORD86	7.98352	0.525796
SNORD87	11.5802	0.521396
SNORD88C	7.28158	0.619069
SNORD89	6.56136	0.746375
SNORD95	9.77596	0.472575
SNORD96A	9.77596	0.472575
SNRNP25	7.92719	0.617045
SNRNP27	8.14024	0.472746
SNRNP40	6.72016	0.670324
SNRNP48	5.02828	0.623779
SNRNP70	7.216	0.5038
SNRPA1	8.20961	0.444875
SNRPB	8.88742	0.501509
SNRPD1	7.89566	0.635289
SNRPD2	11.4423	0.49924
SNRPD3	9.26255	0.527206
SNRPE	7.14583	0.48639
SNRPF	8.584	0.647555
SNRPG	10.0839	0.627766
SNUPN	7.57269	0.520386
SNURF	8.09762	0.625009

SNW1	7.83828	0.578465
SNX1	6.17849	0.42927
SNX10	6.43287	1.15064
SNX13	5.09929	0.425266
SNX14	6.84371	0.513562
SNX18	6.37272	0.494915
SNX27	6.24876	0.417355
SNX3	9.57583	0.497164
SNX30	5.55069	0.723707
SNX4	6.39779	0.559085
SNX5	6.75639	0.462741
SNX6	7.89592	0.586704
SNX8	5.23521	0.407183
SNX9	4.8398	1.36629
SOAT1	5.83709	0.646442
SOCS2	5.38545	0.793456
SOCS3	5.40024	0.730763
SOCS4	5.09191	0.531868
SOCS5	6.11016	0.618576
SOD1	10.6695	0.543859
SOD2	5.69819	0.6283
SOD3	6.22809	0.492239
SOHLH1	5.92934	0.450642
SON	9.17226	0.495629
SORD	4.92196	0.695462
SORT1	5.96549	0.927809
SOS1	6.31028	0.477666
SOWAHC	5.54663	1.45257
SOX15	5.95479	0.403091
SOX4	5.22682	1.03957
SP1	5.9826	0.494767
SP100	6.01716	0.520018
SP110	7.50111	0.700034
SP140	7.14699	0.723462
SP4	6.01092	0.448012
SPACA3	4.86842	1.02766
SPAG1	5.03413	0.823374
SPAG4	9.05414	1.30784
SPAG5	6.51188	0.765934
SPAG5-AS1	5.2624	0.434249
SPAG7	8.60317	0.411055
SPAG9	6.08602	0.467393
SPANXA1	4.90249	0.481478
SPANXA2	4.90249	0.481478
SPAST	5.07093	0.594118
SPATA20	7.43233	0.557155
SPATA21	5.36206	0.44105

SPATA2L	6.13334	0.429031
SPATA5L1	5.32442	0.498106
SPATA7	5.08896	0.465948
SPATC1L	6.30805	0.735754
SPATS2	7.36595	0.502753
SPATS2L	5.15861	0.571284
SPCS1	12.4857	0.407376
SPCS2	10.6587	0.402638
SPCS3	10.5964	0.536715
SPDL1	5.14067	0.748723
SPDYE1	5.34776	0.483352
SPDYE2	6.02343	0.438114
SPDYE2B	5.34776	0.483352
SPDYE5	5.34776	0.483352
SPDYE6	5.34776	0.483352
SPECC1L	6.26399	0.4564
SPECC1L-ADORA2A	6.77997	0.634214
SPEF2	5.20847	0.625513
SPEM1	4.99925	0.488474
SPEN	6.83747	0.534768
SPG20	4.87686	0.43053
SPHAR	6.4089	1.10102
SPIN1	6.49641	0.657021
SPIN2B	6.14275	0.444846
SPINK2	5.64843	1.16404
SPINT1	5.66605	0.482313
SPINT2	7.86542	1.77971
SPN	6.78324	0.445278
SPNS1	6.69217	0.428149
SPOCK2	6.36966	0.571777
SPON2	5.42922	0.685286
SPOP	6.78561	0.441374
SPOPL	6.8169	0.556773
SPPL2A	6.74117	0.712758
SPR	6.5162	0.484182
SPRED3	7.04238	0.410728
SPRR2B	5.1769	0.410475
SPRY1	5.41123	0.556796
SPRY4	4.87232	0.405458
SPRYD3	7.34953	0.416221
SPRYD4	6.03181	0.416903
SPSB2	5.89166	0.411631
SPTBN2	5.81208	0.530398
SPTSSA	7.46356	0.681436
SPTY2D1	6.70159	0.62065
SQLE	5.32445	0.625515
SQRDL	9.43925	0.591198

SQSTM1	7.40408	0.418479
SRA1	8.10401	0.488977
SRBD1	6.64657	0.644571
SRCRB4D	5.96375	0.410758
SRD5A3	5.13324	0.59622
SREK1	5.95238	0.508173
SREK1IP1	6.50322	0.530503
SRFBP1	5.37086	0.731366
SRGAP2	5.1416	0.526764
SRGAP2B	5.02697	0.472645
SRGAP2C	5.21728	0.549039
SRGN	10.5518	0.622162
SRI	7.33481	0.536896
SRM	8.91668	0.736213
SRP14	11.4767	0.474383
SRP19	7.4779	0.407676
SRP54	8.78341	0.661144
SRP68	9.21823	0.425528
SRP72	8.18578	0.409352
SRP9	10.8447	0.723405
SRPK1	7.27111	0.533797
SRPK2	6.06192	0.510149
SRPR	10.3123	0.498405
SRPRB	9.23801	0.440783
SRRD	6.57588	0.549725
SRRM1	8.24372	0.430518
SRSF10	7.3884	0.46847
SRSF11	7.05104	0.634635
SRSF2	10.1927	0.425978
SRSF3	8.2422	0.45569
SRSF4	6.15099	0.454202
SRSF5	8.91545	0.633719
SRSF6	8.28009	0.777615
SRSF7	8.65834	0.51031
SRSF8	7.57911	0.473352
SRSF9	9.05713	0.444176
SRXN1	6.97828	0.471807
SS18	7.16523	0.496204
SS18L1	6.20568	0.655723
SS18L2	9.04978	0.500046
SSB	8.2807	0.597313
SSBP1	7.74918	0.520207
SSBP2	4.8688	0.543522
SSBP3	5.76869	0.584451
SSBP3-AS1	4.94112	0.405865
SSBP4	6.71395	0.555956
SSC5D	6.50293	0.499965

SSFA2	5.33467	0.426131
SSNA1	7.29772	0.415772
SSPN	5.23749	0.701475
SSR1	9.80551	0.662024
SSR2	11.4356	0.479315
SSR3	9.71653	0.624899
SSR4	13.4108	0.645517
SSRP1	7.52781	0.432759
SSSCA1	8.21477	0.542167
SSX2	4.99153	0.725098
SSX5	5.13379	0.437763
SSX7	5.13379	0.437763
ST13	8.40919	0.54472
ST14	5.19616	0.624408
ST3GAL1	5.08942	0.697814
ST3GAL5	8.18482	0.68539
ST3GAL6	6.667	0.837051
ST5	5.21628	0.71516
ST6GAL1	7.45865	0.622949
ST6GALNAC4	7.24194	0.483544
ST7	6.36975	0.442949
ST7-OT3	6.36975	0.442949
ST8SIA4	6.24034	0.940067
ST8SIA6-AS1	5.25209	0.980482
STAG1	6.8033	0.414411
STAG2	8.57685	0.697713
STAG3L1	6.13622	0.564654
STAG3L2	6.27325	0.591426
STAG3L3	6.0724	0.504136
STAG3L4	5.21396	0.479163
STAG3L5P-PVRIG2P-PILRB	7.8853	0.714721
STAM	6.68871	0.67587
STAM2	5.46291	0.415977
STAP1	8.10747	1.81785
STAP2	5.12849	0.45825
STARD10	6.10416	0.45777
STARD3NL	8.04902	0.618424
STARD4	6.71855	0.770255
STARD5	7.71941	0.894532
STARD9	5.79204	0.505077
STAT1	8.22322	0.961961
STAT2	7.1208	0.472444
STAT3	7.13299	0.536447
STAT4	6.60319	1.16062
STAT6	5.94417	0.430063
STAU1	8.75612	0.465717
STEAP1B	5.59996	0.955013

STEAP3	5.02787	0.476282
STIL	5.05548	0.71513
STIM2	5.91211	0.414596
STIP1	7.88225	0.58248
STK10	6.40354	0.413406
STK11IP	5.90273	0.400128
STK17A	5.78964	0.760502
STK17B	6.62561	1.07912
STK25	5.77202	0.521063
STK38L	6.03904	0.605409
STK4	6.23289	0.50345
STMN1	5.90209	0.447142
STOM	6.82651	0.783351
STOML2	8.70996	0.597904
STRA13	8.01585	0.434991
STRADB	7.07707	0.642518
STRAP	7.77564	0.625611
STRBP	7.85137	0.666171
STRIP1	6.03227	0.460662
STRN3	5.72368	0.55384
STRN4	5.68526	0.425838
STS	5.42415	0.823377
STT3A	9.99984	0.732045
STT3B	10.6036	0.784807
STX10	6.73531	0.402088
STX11	5.59481	0.866218
STX12	7.30007	0.540938
STX16	6.97555	0.52744
STX17	6.35522	0.454043
STX3	5.17368	0.406649
STX5	6.62177	0.479505
STX7	5.72037	0.525777
STXBP3	5.61168	0.635213
STXBP6	5.10966	1.01589
STYX	5.3455	0.637381
SUB1	10.2964	0.436726
SUCLA2	6.80596	0.990158
SUCLG2	8.64069	0.568909
SUCO	8.26868	0.650697
SUDS3	5.97883	0.677925
SUGT1	5.8768	0.407884
SULF2	8.09153	2.39303
SULT1A1	7.20677	0.474063
SULT1A2	7.06583	0.494258
SUMF1	7.40278	0.565586
SUMF2	9.02159	0.704121
SUMO1	7.29826	0.407837

SUMO4	8.00182	0.442874
SUN2	7.03003	0.456051
SUPT16H	7.03626	0.648354
SUPT3H	5.14766	0.672322
SUPV3L1	6.48789	0.586245
SURF2	5.95803	0.437983
SURF4	9.76181	0.40115
SURF6	6.59694	0.405032
SUSD1	5.03144	0.614416
SUSD3	5.72486	0.486441
SUV39H1	5.60041	0.445652
SUV39H2	4.86093	0.539009
SUV420H1	6.71249	0.494178
SUZ12P1	5.70555	0.539937
SVIP	8.56259	0.606111
SWI5	7.03915	0.442349
SWSAP1	5.53255	0.422469
SWT1	6.43389	0.561374
SYAP1	8.07738	0.45449
SYK	4.93212	1.02005
SYNCRIP	7.50889	0.469829
SYNE2	4.84263	0.519447
SYNE3	6.33966	0.607148
SYNGR1	6.09171	0.559473
SYNGR2	6.3934	0.449749
SYNJ2BP	5.50445	0.726903
SYNJ2BP-COX16	5.47597	0.659731
SYNM	5.49381	1.35636
SYNRG	6.62153	0.458063
SYPL1	7.79696	1.3844
SYT1	5.74134	1.56387
SYTL1	7.78501	0.997052
SYVN1	8.89844	0.542338
TAB2	7.3969	0.707928
TACC1	5.39715	0.525464
TACC3	5.7431	0.630856
TAF11	6.76749	0.479182
TAF12	7.576	0.542623
TAF13	5.30529	0.52216
TAF15	6.38975	0.553474
TAF1A	5.02524	0.828324
TAF1A-AS1	4.91491	0.642741
TAF1D	7.83804	0.757106
TAF2	7.19664	0.415897
TAF4B	6.91654	0.954745
TAF5	5.23899	0.67549
TAF6	7.17126	0.452253

TAF7	9.9041	0.654705
TAF9	9.71182	0.583363
TAF9B	6.44924	0.470622
TAGAP	6.82472	0.940412
TAGLN2	7.37337	0.961249
TALDO1	9.33669	0.507521
TANGO2	6.37026	0.400997
TANK	6.27521	0.513785
TAOK3	6.62019	0.451605
TAP1	8.72802	0.632767
TAP2	6.44548	0.416536
TAPBP	7.81781	0.483602
TAPBPL	8.76832	0.759796
TAPT1-AS1	5.60593	0.775993
TARBP1	7.0563	0.672033
TARDBP	7.84796	0.570306
TARS2	6.8063	0.424864
TAS2R43	5.35379	0.638985
TATDN1	8.05634	0.649488
TATDN3	6.252	0.666548
TAX1BP1	8.53661	0.497088
TAX1BP3	5.92055	0.606799
TBC1D1	5.2927	0.453447
TBC1D10B	6.60187	0.482812
TBC1D10C	6.73731	0.731492
TBC1D14	7.6162	0.442729
TBC1D15	6.24307	0.528867
TBC1D23	7.04827	0.62473
TBC1D27	7.05349	0.886161
TBC1D3	7.00544	0.473434
TBC1D30	4.92563	0.775709
TBC1D3C	7.00544	0.473434
TBC1D3F	7.00544	0.473434
TBC1D3H	7.00544	0.473434
TBC1D3P1-DHX40P1	6.14807	0.715527
TBC1D9	8.66666	1.12883
TBCA	11.02	0.573761
TBCB	7.89628	0.586552
TBCC	8.35763	0.510387
TBCCD1	6.09626	0.622416
TBCE	6.27011	0.619188
TBCEL	6.9645	1.18073
TBCK	7.12082	0.711695
TBK1	7.29424	0.468343
TBL1X	5.80404	0.505998
TBL1XR1	6.95876	0.584402
TBP	6.01207	0.420235

TBPL1	7.59944	0.684073
TBX21	4.87986	0.54264
TBXAS1	5.54089	1.18232
TCEA3	4.99397	0.678295
TCEAL1	6.50221	0.676169
TCEAL3	7.98878	0.815139
TCEAL4	7.93277	0.807143
TCEAL8	7.94863	0.8685
TCEB1	7.37371	0.596741
TCEB3-AS1	6.61293	0.565961
TCERG1	6.12398	0.539311
TCF12	6.38479	0.547042
TCF15	6.06178	0.561052
TCF19	5.59398	0.906191
TCF4	8.1733	1.67134
TCFL5	4.86228	0.702312
TCIRG1	8.07357	0.60871
TCL1A	5.22267	0.459462
TCN2	6.33574	0.749195
TCP1	6.99147	0.557052
TCTA	6.95529	0.509466
TCTEX1D2	5.21859	0.714289
TCTN1	6.44365	0.432151
TCTN3	8.03052	0.452504
TDG	6.91375	0.562907
TDP2	8.44517	0.754179
TDRD3	5.48437	0.433133
TDRD7	7.16813	0.705547
TDRG1	5.50173	0.416519
TEC	4.96426	0.447346
TEFM	5.93454	0.632724
TEKT4P2	7.04277	0.694091
TEN1	6.30427	0.497284
TEP1	5.23977	0.434533
TERF1	6.89778	0.581803
TERF2IP	8.40083	0.603615
TES	7.29847	0.448562
TESK2	6.99523	0.56594
TET1	4.83218	0.761142
TET2	5.25666	0.645896
TEX10	5.97233	0.521758
TEX101	4.95157	0.500575
TEX2	7.27301	0.415568
TEX30	5.25606	0.554411
TFAM	6.29431	0.515597
TFAP2A-AS1	5.36856	0.562398
TFCP2	6.09252	0.424341

TFDP1	6.06724	0.649894
TFDP2	5.56892	0.437411
TFEB	6.41818	0.462006
TFG	6.75112	0.449944
TFPT	6.31118	0.417404
TFRC	6.64949	0.411439
TG	5.26067	0.522507
TGDS	5.78011	0.679184
TGFA	4.87333	0.673322
TGFBI	5.47402	1.0796
TGFBR1	5.36918	0.530165
TGFBR2	5.56053	0.814908
TGIF2	6.24055	0.52084
TGIF2-C20orf24	10.386	0.410734
TGOLN2	7.16149	0.432532
THAP1	5.28201	0.760034
THAP5	5.26663	0.514724
THAP7	7.65419	0.430428
THAP9-AS1	7.94497	0.64526
THEG	4.81341	0.447464
THEM4	6.69154	0.651035
THEM6	5.14482	0.462607
THEMIS2	6.99034	1.12255
THG1L	7.0635	0.922467
THOC1	7.56531	0.483139
THOC2	7.03224	0.628488
THOC3	7.14163	0.724943
THOC6	5.78245	0.421063
THOC7	9.16122	0.611133
THOP1	5.9776	0.479805
THRIL	6.35762	0.76255
THSD1	5.34423	0.568436
THTPA	7.54925	0.506247
THUMPD1	7.88119	0.556345
THUMPD2	6.147	0.503811
THUMPD3	6.67412	0.419252
THUMPD3-AS1	5.37618	0.532288
THY1	5.48293	0.516642
THYN1	7.75414	0.67269
TIA1	6.68089	0.494039
TIAM1	5.44454	0.863365
TICAM1	6.65355	0.448638
TICAM2	5.76709	0.512313
TIFA	6.38502	0.64419
TIGD1	6.48822	0.731022
TIGD2	6.31203	0.799942
TIMELESS	5.42104	0.409021

TIMM10	8.49951	0.602649
TIMM10B	6.9244	0.554727
TIMM17A	6.35668	0.417861
TIMM21	7.13748	0.69088
TIMM23B	7.64007	0.480323
TIMM44	6.67181	0.421644
TIMM8B	7.93967	0.699988
TIMM9	7.29311	0.582643
TIMMDC1	8.61544	0.493664
TIMP1	8.96998	2.0257
TIMP2	6.87162	1.32279
TIPARP	9.27252	0.871086
TIPIN	6.00446	1.00163
TIPRL	6.41211	0.72449
TISP43	4.92248	0.429384
TJP1	6.47893	1.69366
TK1	5.44752	0.763216
TKT	6.94878	0.481388
TLCD1	5.03368	0.507602
TLDC2	4.82987	0.433972
TLE1	5.41488	0.901933
TLE3	7.02677	0.591449
TLE4	6.31369	0.668748
TLR1	6.52368	1.13326
TLR10	6.50334	1.44068
TLR2	4.90369	0.491464
TLR5	4.80589	0.415014
TLR6	5.8205	0.619751
TLR9	5.81222	0.651499
TM2D1	5.85717	0.411253
TM2D2	8.33427	0.632856
TM2D3	6.91978	0.813045
TM7SF2	6.98321	0.472446
TM9SF2	10.5433	0.696526
TM9SF4	8.10087	0.457873
TMBIM1	6.90224	0.46861
TMBIM6	10.6511	0.524131
TMC4	5.30067	0.576994
TMC6	6.2301	1.16381
TMC8	5.34024	0.762047
TMCC3	6.05022	1.00655
TMCO1	9.65141	0.598706
TMCO3	4.81088	0.400876
TMCO6	5.53372	0.464546
TMED10	9.16061	0.548318
TMED2	10.7194	0.703034
TMED5	6.8887	0.433823

TMED7	8.02942	0.779156
TMED7-TICAM2	6.89825	0.542396
TMED9	10.581	0.438428
TMEFF1	4.84889	1.59101
TMEM101	6.69144	0.489643
TMEM106B	7.36996	0.546086
TMEM107	6.76083	0.74022
TMEM109	8.24949	0.455978
TMEM11	7.4538	0.407798
TMEM115	6.25642	0.434003
TMEM117	5.67388	0.534154
TMEM120A	7.29718	0.542357
TMEM123	9.87443	1.25096
TMEM126A	9.46076	0.623147
TMEM128	6.4921	0.675928
TMEM133	5.81193	1.05755
TMEM135	6.63704	0.457053
TMEM138	8.03438	0.547324
TMEM140	6.53158	0.816672
TMEM147	9.80922	0.586213
TMEM14A	8.29637	0.827783
TMEM14B	9.65943	0.507972
TMEM14C	10.0541	0.5492
TMEM151A	5.10177	0.451536
TMEM154	7.26373	1.15177
TMEM156	5.70475	1.52082
TMEM160	6.53303	0.506374
TMEM161B	6.21561	0.413827
TMEM161B-AS1	5.75891	0.442847
TMEM164	6.14292	0.585681
TMEM167A	9.88469	0.599025
TMEM167B	8.18312	0.651756
TMEM168	7.0109	0.717352
TMEM173	6.42155	1.1142
TMEM175	6.80758	0.410654
TMEM176A	5.83635	0.76104
TMEM176B	4.81461	0.602727
TMEM179	4.94583	0.446793
TMEM179B	8.64465	0.436963
TMEM181	6.86339	0.760196
TMEM183A	6.74953	0.469352
TMEM183B	6.74953	0.469352
TMEM184B	8.09239	0.892667
TMEM184C	7.53586	0.513936
TMEM185A	5.69071	0.570794
TMEM185B	5.18089	0.511737
TMEM186	6.5147	0.498639

TMEM187	6.05091	0.609867
TMEM19	7.29096	0.658862
TMEM192	8.2217	0.515237
TMEM194A	5.85618	0.735896
TMEM2	5.70772	0.775295
TMEM203	7.83767	0.571924
TMEM205	10.0072	0.606049
TMEM208	9.31253	0.586045
TMEM209	6.13156	0.460332
TMEM214	8.50523	0.528213
TMEM216	7.23142	0.580763
TMEM220	6.34456	1.26941
TMEM230	10.5288	0.43688
TMEM243	10.3956	0.668891
TMEM245	6.96897	0.474182
TMEM248	8.23426	0.427871
TMEM251	7.00029	0.880289
TMEM254	5.64881	0.410096
TMEM255B	4.95161	0.429157
TMEM256	8.68057	0.596883
TMEM256-PLSCR3	7.49268	0.445814
TMEM258	11.8947	0.452269
TMEM260	6.72526	0.587155
TMEM261	6.65883	0.444758
TMEM30A	8.16603	0.742999
TMEM33	6.85131	0.490464
TMEM37	5.09131	0.44071
TMEM38A	5.71161	0.496221
TMEM38B	7.0282	0.631502
TMEM39B	5.965	0.525259
TMEM41A	6.84847	0.482788
TMEM41B	7.84655	0.66087
TMEM42	7.12638	0.471329
TMEM44-AS1	6.28083	0.541829
TMEM45A	8.13615	1.78105
TMEM5	7.2492	0.550418
TMEM50A	8.95087	0.436377
TMEM50B	7.2849	0.425774
TMEM51	5.87129	0.680719
TMEM55A	5.77133	0.613679
TMEM55B	7.22154	0.479306
TMEM56	5.09757	0.979051
TMEM57	6.96513	0.480106
TMEM59	8.44974	0.521345
TMEM60	7.5966	0.600012
TMEM64	6.41044	0.814291
TMEM68	5.08409	0.642377

TMEM69	7.3912	0.767705
TMEM87B	6.84116	0.675638
TMEM9	7.66029	0.65055
TMEM97	6.72303	0.828776
TMEM99	5.93267	0.798907
TMEM9B	8.33651	0.624633
TMF1	6.61017	0.553368
TMOD3	4.92022	0.452585
TMPO	5.94041	0.78514
TMPPE	8.82448	0.68948
TMSB10	12.1772	0.567062
TMSB15A	4.81914	0.895595
TMSB4X	10.3285	2.02011
TMTC2	6.60861	0.707151
TMTC4	5.02709	0.479212
TMUB1	6.78019	0.403159
TMX1	9.39108	0.619381
TMX2	9.40472	0.496506
TMX3	5.7208	0.595501
TMX4	6.74091	0.894416
TNFAIP2	5.0929	0.491403
TNFAIP3	9.23042	1.508
TNFAIP8	8.00905	1.01725
TNFAIP8L1	5.44832	0.603438
TNFAIP8L2-SCNM1	8.15987	0.50158
TNFRSF10A	6.13816	0.524402
TNFRSF10B	5.54268	0.66402
TNFRSF10D	4.94065	0.426705
TNFRSF12A	6.27726	0.42221
TNFRSF13B	7.8975	0.778293
TNFRSF13C	5.99215	0.48353
TNFRSF14	8.22468	0.631216
TNFRSF17	12.3689	0.606543
TNFRSF18	5.11198	0.929734
TNFRSF1A	5.3834	0.550845
TNFRSF1B	5.57631	0.409529
TNFRSF4	5.0958	0.436256
TNFSF10	7.4288	1.62525
TNFSF12	4.86546	0.41091
TNFSF12-TNFSF13	5.43662	0.511751
TNFSF13	5.66946	0.65005
TNFSF13B	5.12452	0.902031
TNFSF8	5.18376	1.09104
TNIP1	7.9934	0.729643
TNKS2	5.63014	0.439409
TNPO3	6.96036	0.42285
TNRC6B	6.76407	0.571335

TNRC6C-AS1	4.93021	0.436038
TNS3	6.832	0.635795
TOB1	7.9637	1.08802
TOMM20	10.3598	0.622846
TOMM22	7.18751	0.418146
TOMM34	6.1946	0.458754
TOMM40L	5.83001	0.51137
TOMM5	10.0948	0.566473
TOMM6	10.4631	0.412488
TOMM7	11.5499	0.514434
TOMM70A	7.9679	0.679755
TOP1	9.0748	0.632326
TOP2B	8.84177	0.56332
TOPBP1	7.56489	0.776293
TOPORS	7.85777	0.823617
TOPORS-AS1	7.71439	0.507627
TOR1AIP1	5.94104	0.467777
TOR1B	7.60785	0.582899
TOR3A	8.05819	0.6421
TP53	5.84064	0.814126
TP53BP2	6.46339	0.710303
TP53I3	5.72427	0.573728
TP53INP1	7.47033	0.540871
TP53INP2	6.4396	0.794209
TP53RK	5.85661	0.716376
TP53TG1	6.18666	0.565329
TP73-AS1	4.94966	0.477956
TPBG	4.8189	0.958171
TPD52	9.13198	0.63853
TPD52L1	4.94079	0.747753
TPD52L2	7.13587	0.462069
TPI1	7.46493	0.420891
TPK1	5.46589	0.836654
TPM4	7.06065	0.629958
TPP1	7.65993	0.483612
TPP2	6.31987	0.527498
TPR	6.83269	0.5689
TPRA1	6.11986	0.512042
TPRG1L	7.36205	0.607206
TPRKB	8.57409	0.574471
TPST1	5.24999	0.495912
TPST2	7.74085	1.07791
TPX2	5.63614	1.12995
TRA2A	7.58841	0.579614
TRA2B	8.25277	0.407824
TRABD2A	5.10478	0.518471
TRAF3	6.27048	0.515754

TRAF3IP3	5.8617	0.975652
TRAF6	6.08493	0.409655
TRAK2	6.69195	0.630256
TRAM1	10.9808	0.413962
TRAM2	7.85603	0.60426
TRAM2-AS1	5.90487	0.470544
TRANK1	6.54101	0.846795
TRAPPC1	7.90781	0.62979
TRAPPC10	6.21348	0.509319
TRAPPC12	7.04396	0.455668
TRAPPC2P1	7.74703	0.638789
TRAPPC4	6.68084	0.522342
TRAPPC5	7.98098	0.48021
TRAPPC6A	8.11463	0.518829
TRAPPC6B	6.39961	0.729753
TRAPPC8	8.96332	0.562539
TRAT1	5.94277	1.96658
TRBC1	5.06107	0.531492
TREML2	5.61767	0.532894
TRG-AS1	7.10794	1.30297
TRIAP1	8.30598	0.516814
TRIB1	8.05308	0.584504
TRIB2	5.86852	1.08177
TRIB3	5.85021	0.684494
TRIM13	4.94229	0.47154
TRIM17	5.06031	0.407531
TRIM21	5.98823	0.553469
TRIM22	6.91117	1.96623
TRIM23	5.37314	0.666536
TRIM24	5.40493	0.4375
TRIM27	7.72821	0.556041
TRIM28	8.57377	0.634457
TRIM32	5.10167	0.403712
TRIM33	6.19367	0.480947
TRIM37	6.12284	0.456948
TRIM38	7.39572	0.470076
TRIM41	6.93466	0.485524
TRIM44	7.31318	0.466156
TRIM47	6.24162	0.564206
TRIM52	7.58567	0.655019
TRIM56	6.9531	0.49386
TRIM59	5.27092	0.842886
TRIM68	5.27964	0.536129
TRIM69	6.73969	0.570748
TRIML2	5.05496	0.40507
TRIP11	4.90392	0.409745
TRIP12	6.95948	0.517563

TRIP13	5.41	1.04643
TRIP4	7.01457	0.582632
TRIP6	5.40824	0.484056
TRIT1	6.64248	0.558719
TRMT10C	7.48902	0.715723
TRMT11	7.39981	0.799756
TRMT112	11.2861	0.438782
TRMT12	6.71325	0.558925
TRMT13	6.22846	0.873188
TRMT1L	5.6137	0.762961
TRMT2B	5.62138	0.427868
TRMT5	7.6777	0.501047
TRMT6	6.6858	0.513095
TRMT61B	6.9543	0.681181
TRNT1	6.80658	0.720704
TROAP	5.81125	0.521619
TROVE2	6.69468	0.457525
TRPM2	5.98894	0.423801
TRPM4	5.4361	1.71731
TRPV2	5.73093	0.495444
TRPV4	5.29319	0.45202
TRRAP	6.51729	0.616294
TRUB2	7.54102	0.450268
TSC1	7.29506	0.473157
TSC22D3	8.65411	0.61087
TSEN15	7.86098	0.735662
TSEN2	5.7302	0.508549
TSEN34	7.65852	0.493446
TSG101	8.77826	0.443558
TSHR	5.2464	1.11803
TSHZ1	5.43622	0.752392
TSN	7.24136	0.472291
TSNAX	7.6051	0.813181
TSPAN13	10.1056	1.01191
TSPAN31	8.49384	0.51424
TSPAN32	5.73048	0.414684
TSPAN33	6.33197	0.461282
TSPAN4	5.1714	0.497602
TSPAN5	5.53079	0.666882
TSPAN7	6.30692	2.01171
TSPO	7.66047	0.800568
TSPYL2	7.86838	0.56879
TSPYL4	7.027	0.924873
TSPYL5	5.58659	1.25681
TSR1	6.45626	0.44815
TSSC1	6.87137	0.413362
TST	7.45425	0.683621

TSTA3	7.53986	0.499104
TSTD1	9.31466	0.650906
TTC1	8.12751	0.489964
TTC13	7.6914	0.729875
TTC14	6.88607	0.599065
TTC19	8.43588	0.499498
TTC28	5.1041	0.622831
TTC3	8.73101	0.559963
TTC31	6.15556	0.48284
TTC32	7.6458	0.546564
TTC37	7.41652	0.648936
TTC3P1	8.73101	0.559963
TTC4	6.42091	0.557092
TTC8	5.08175	0.832504
TTC9C	5.28703	0.434234
TTF1	6.57214	0.551298
TTI1	6.48969	0.620466
TTI2	5.97902	0.606594
TTLL1	5.17552	0.412221
TTLL12	6.3939	0.463902
TTLL7	5.93228	0.949495
TTLL7-IT1	4.98781	0.802972
TTPAL	6.10169	0.430641
TTYH2	5.39614	0.42017
TUBA1A	5.96142	1.64418
TUBA1B	9.82612	0.584036
TUBA1C	10.7824	0.643507
TUBA4A	8.9446	0.523858
TUBB	9.73277	0.801352
TUBB2A	6.27199	0.959626
TUBB2B	5.07054	0.608203
TUBB3	8.21443	0.490627
TUBB4B	9.2525	0.583638
TUBD1	5.15535	0.513752
TUBG1	7.21969	0.885895
TUBG2	5.68688	0.650312
TUBGCP4	6.32935	0.461582
TUBGCP5	5.53824	0.506484
TUFT1	6.76781	1.04886
TUSC1	6.48866	1.45595
TUSC5	5.79012	0.466595
TVP23B	7.94166	0.733812
TVP23C	5.68033	0.51007
TVP23C-CDRT4	5.81023	0.723584
TWISTNB	5.03938	0.571034
TWSG1	6.85329	0.824804
TXLNG	6.07503	0.458624

TXLNGY	6.08478	2.33633
TXN	9.27676	0.702608
TXNDC11	9.81671	0.648998
TXNDC12	7.59876	0.477544
TXNDC15	10.0572	0.557401
TXNDC16	6.20727	0.817659
TXNDC17	7.22315	0.405992
TXNDC9	6.04376	0.516522
TXNIP	12.4994	0.63787
TXNL1	6.96305	0.435933
TYMP	5.51566	0.514041
TYMS	6.2978	0.953398
TYROBP	6.13097	1.11429
TYW1	7.69994	0.428737
TYW3	7.68482	0.635026
U2AF1	7.77163	0.511906
U2AF1L4	6.87808	0.474448
U2SURP	6.97993	0.533293
U50535	7.74081	0.788714
U91328.2	5.68561	0.49635
UAP1	11.1609	0.866591
UBA2	7.50087	0.423593
UBA3	8.4214	0.53492
UBA5	8.8858	0.449954
UBA6	5.40113	0.428063
UBA7	7.42395	0.634991
UBAC1	6.93939	0.471358
UBAC2	5.52399	0.552136
UBALD1	6.00565	0.443035
UBALD2	8.80391	0.760151
UBAP2	8.21316	0.548054
UBB	13.0423	0.424283
UBBP1	7.38706	0.517166
UBBP4	7.38706	0.517166
UBE2A	8.16001	0.674679
UBE2C	6.02266	1.09449
UBE2D2	7.58467	0.409171
UBE2E1	10.7886	0.578355
UBE2E2	7.73515	0.694065
UBE2E3	9.62615	0.52413
UBE2G1	9.07654	0.505776
UBE2J1	10.4167	0.461474
UBE2K	7.81236	0.481282
UBE2L6	9.06829	0.651595
UBE2M	7.88105	0.594416
UBE2N	8.53791	0.627067
UBE2Q2	8.43822	0.834602

UBE2Q2L	5.62329	0.472706
UBE2QL1	7.89207	1.37209
UBE2R2	7.51554	0.452045
UBE2S	7.69844	0.841765
UBE2T	5.88965	1.25502
UBE2V2P3	5.58914	0.428778
UBE4A	8.96283	0.605986
UBFD1	6.15806	0.603767
UBL3	7.42138	0.645097
UBL4A	7.06529	0.496038
UBL5	10.4033	0.4351
UBL7	7.69971	0.586855
UBL7-AS1	5.55021	0.58648
UBLCP1	7.49652	0.769548
UBP1	7.7079	0.55249
UBQLN1	8.27108	0.439855
UBQLN2	6.59235	0.60962
UBR1	6.40704	0.560219
UBR5	7.1573	0.689357
UBR7	6.76884	0.75176
UBTD2	5.8798	0.639015
UBXN11	5.41247	0.418496
UBXN2A	7.7806	0.451392
UBXN2B	6.47633	0.513817
UBXN4	8.07175	0.40083
UBXN7	6.86291	0.754626
UBXN8	6.90941	0.757703
UCHL1	5.91605	1.60533
UCHL3	8.49549	0.702096
UCHL5	5.83252	0.438133
UCK2	6.66102	0.622971
UCN2	5.12422	0.432279
UCP2	8.78468	0.777672
UEVLD	5.23676	0.453147
UFC1	10.7294	0.526412
UFD1L	9.40915	0.417502
UFL1	6.77558	0.52315
UFM1	8.52831	0.687245
UFSP1	5.78554	0.408668
UFSP2	7.93056	0.496557
UGCG	7.91221	0.70154
UGDH	6.90526	0.720291
UGP2	7.3429	0.48919
UGT2B17	6.73441	2.17234
UGT8	5.42109	1.2554
UHMK1	7.58854	0.763296
UHRF1	5.29119	1.07186

UHRF2	8.72313	0.46945
ULK3	7.4175	0.626686
UMPS	5.44302	0.419939
UNC13B	7.58436	0.671544
UNC50	8.0538	0.444995
UNC93B1	6.36638	0.501246
UNG	6.3389	0.922862
UPF2	6.91532	0.493571
UPF3A	6.84369	0.592742
UPF3B	5.67444	0.411018
UPK3BL	7.28041	0.571769
UPP1	5.32912	0.679348
UPRT	5.95182	0.604296
UQCR10	7.87351	0.427199
UQCRB	8.42652	0.449019
UQCRC1	9.06654	0.471462
UQCRC2	7.51414	0.449401
UQCRFS1	10.6233	0.487809
UQCRH	11.5229	0.5162
UQCRHL	11.5229	0.5162
UQCRQ	11.0052	0.481577
URB2	6.33025	0.53068
URGCP	6.29907	0.436183
URGCP-MRPS24	10.6221	0.674739
UROD	6.56419	0.40529
USE1	7.41573	0.570435
USMG5	11.5449	0.409612
USO1	9.84608	0.515443
USP1	8.05102	0.802608
USP10	7.04042	0.472616
USP11	8.24688	0.590816
USP12	5.06749	0.521645
USP13	5.73455	0.485907
USP14	8.37977	0.483822
USP15	6.30357	0.42135
USP16	8.76382	0.50303
USP18	5.4086	1.07107
USP20	6.06904	0.433669
USP21	6.35228	0.503448
USP24	5.92931	0.463815
USP25	4.95384	0.484342
USP28	6.1234	0.467183
USP3	8.05314	0.539275
USP30	5.57375	0.520809
USP30-AS1	5.93908	0.477306
USP32P2	7.18767	0.937451
USP33	6.18883	0.438887

USP34	7.25972	0.451116
USP37	4.94294	0.417295
USP38	6.4926	0.588865
USP40	5.97335	0.513023
USP47	6.68532	0.447403
USP48	7.04859	0.458403
USP54	5.04592	0.449679
USP6NL	5.30365	0.676015
USP6NL-IT1	5.30365	0.676015
USP8	7.20297	0.477245
USPL1	5.72136	0.580723
UTP14A	6.33641	0.554191
UTP14C	7.06049	0.662687
UTP15	4.97103	0.455852
UTP18	6.09567	0.413137
UTP20	6.17483	0.523532
UTP23	5.98948	0.506356
UTP3	8.11537	0.9957
UTRN	6.78859	0.716666
UVRAG	7.2225	0.70047
UXS1	6.67799	0.694311
UXT	10.4425	0.436384
VAMP1	6.20441	0.572098
VAMP2	6.66081	0.551169
VAMP3	8.37113	0.517082
VAMP4	6.74839	0.600416
VAMP7	8.75306	0.823668
VAMP8	8.91425	0.560803
VARS	6.83264	0.562816
VARS2	8.28125	0.583276
VASP	7.06119	0.504418
VAT1	7.49043	0.418918
VAV1	7.31836	0.541914
VAV3	4.86823	0.610926
VBP1	8.89458	0.776919
VCL	4.89994	0.44721
VCP	9.52638	0.419499
VCPKMT	6.97487	0.673797
VCX2	5.53477	0.501089
VCY	6.02824	0.624345
VCY1B	6.02824	0.624345
VDAC1	7.19686	0.460655
VDAC2	10.0315	0.426153
VDAC3	7.57345	0.535152
VDR	6.49759	0.462329
VEGFA	6.91177	0.620508
VEGFB	5.95959	0.536652

VEZF1	6.75366	0.647818
VEZT	5.89647	0.527396
VGLL4	6.9554	0.434237
VILL	6.80911	0.565246
VIM	9.77558	1.02548
VIMP	11.0237	0.597143
VIPAS39	5.64475	0.405103
VIPR1	5.62975	0.427419
VKORC1	9.16329	0.619918
VKORC1L1	5.17224	0.557918
VMP1	7.38789	1.15071
VNN2	5.19008	0.85236
VOPP1	9.985	0.744346
VPREB3	5.39793	1.75621
VPS13A	5.33071	0.462794
VPS13B	5.51871	0.475464
VPS13C	6.17634	0.493244
VPS25	7.63323	0.492493
VPS28	8.90819	0.435084
VPS35	7.03408	0.546459
VPS36	5.07423	0.473959
VPS37A	5.74471	0.756331
VPS37B	7.9143	0.771189
VPS4B	7.22198	0.879735
VPS51	9.63405	0.745013
VPS54	7.70636	0.438342
VPS8	5.14024	0.432794
VPS9D1	5.19224	0.430913
VRK1	7.45005	0.93639
VRK2	7.30026	0.616729
VSTM2L	5.33778	0.421059
VTA1	6.25513	0.581205
VTI1B	7.01054	0.53844
VWA5A	5.88798	0.774055
VWA8	5.86011	0.549216
VWCE	5.45214	0.501361
WAC-AS1	7.48761	0.457185
WAPAL	6.31105	0.41144
WARS	9.24893	1.10939
WARS2	4.90508	0.41004
WASF2	6.50904	0.551091
WBP1	7.91836	0.437136
WBP11	6.9905	0.473464
WBP1L	7.19971	0.505847
WBP2	6.9334	0.665128
WBP4	4.88733	0.493987
WBP5	7.19884	1.39018

WDFY1	6.82562	0.657178
WDFY2	5.2024	0.474444
WDPCP	6.34365	0.425145
WDR11	6.84207	0.773502
WDR18	6.77719	0.546329
WDR24	5.7196	0.449013
WDR26	6.62352	0.471797
WDR3	5.14852	0.547027
WDR34	6.14604	0.707089
WDR36	5.66414	0.655412
WDR4	6.19497	0.464916
WDR41	6.47647	0.402857
WDR43	5.91444	0.744895
WDR45	8.2354	0.550432
WDR46	7.5345	0.437084
WDR47	5.40352	0.565671
WDR48	7.11098	0.40379
WDR52	5.76116	0.727159
WDR53	5.6145	0.475614
WDR54	8.00968	0.57058
WDR5B	4.80872	0.684339
WDR61	7.19107	0.409103
WDR64	5.67708	0.995109
WDR70	6.99842	0.493562
WDR73	7.16216	0.401332
WDR75	6.75778	0.625433
WDR82	8.4043	0.56739
WDR83OS	8.96655	0.405928
WDR89	4.97231	0.426596
WDR92	6.88365	0.462117
WDSUB1	5.96802	0.679094
WDYHV1	7.52721	0.756718
WEE1	5.69844	1.02353
WFDC12	5.68773	0.409597
WFS1	7.34339	0.629517
WHAMM	7.31503	0.613764
WHSC1	5.74887	1.3235
WIPF1	7.61325	0.596837
WIPI1	9.61285	0.6865
WNT10A	5.4684	0.525751
WNT10B	6.14286	0.626011
WNT11	5.33159	0.545486
WNT4	5.08093	0.41989
WNT5A	6.26598	1.91881
WNT5B	5.07635	0.441269
WRB	8.63547	0.751997
WRN	5.83437	0.560283

WSB1	6.17493	0.639451
WSB2	6.28894	1.1086
WWC3	7.07373	0.660108
WWOX	5.65821	0.416664
WWP1	6.08897	0.505229
XAB2	5.45121	0.459528
XAF1	6.69005	1.36481
XAGE-4	6.19224	0.523914
XAGE1B	5.89805	1.06669
XAGE1E	5.89805	1.06669
XAGE3	5.08637	0.70427
XBP1	8.52675	0.439534
XIAP	7.07011	0.538365
XK	6.15391	1.47977
XPA	6.81514	0.543408
XPC	7.97669	0.402517
XPO1	8.26908	0.63764
XPO4	5.92944	0.460617
XPO5	6.56222	0.424761
XPO7	6.2536	0.490201
XPOT	8.21809	0.799905
XPR1	5.68988	0.600497
XRCC2	6.31694	0.407888
XRCC3	5.16092	0.41619
XRCC4	6.056	0.715275
XRCC6BP1	4.86894	0.433215
XRN1	6.75174	0.591214
XRN2	7.29182	0.435365
XRRA1	6.04965	0.416448
XXbac-BPG32J3.18	5.13735	0.437848
Y16709	11.6054	0.548575
YAE1D1	6.19408	0.849685
YBEY	6.65038	0.521488
YBX1	8.97893	0.411912
YBX3	5.79071	1.10934
YDJC	6.40505	0.40475
YEATS4	5.13865	0.665483
YES1	5.4992	0.578031
YIF1A	8.973	0.551242
YIPF1	7.90635	0.611834
YIPF3	8.02505	0.584046
YIPF5	8.46085	0.578332
YIPF6	6.96319	0.465171
YKT6	6.97039	0.531406
YLPM1	6.22699	0.405155
YOD1	5.95074	0.848918
YPEL2	5.33749	0.550021

YPEL3	7.19995	0.616148
YPEL5	11.2605	0.744115
YRDC	7.00329	0.429931
YTHDC1	5.99855	0.455403
YTHDC2	5.81586	0.612402
YTHDF2	9.03088	0.410029
YTHDF3	7.40501	0.72724
YTHDF3-AS1	6.48065	0.401803
YWHAB	9.37453	0.587858
YWHAE	8.68085	0.715044
YWHAG	9.43556	0.771813
YWHAH	5.17244	0.417302
YWHAQ	10.5352	0.425569
YWHAZ	8.92864	0.500217
YY1AP1	8.50763	0.550272
ZADH2	5.22419	0.447774
ZAK	4.8142	0.492364
ZBED3	6.05927	0.732049
ZBED5-AS1	7.62825	0.685178
ZBED6	5.18608	0.630235
ZBED6CL	5.77025	0.49653
ZBP1	7.88443	0.684821
ZBTB11	6.70897	0.506047
ZBTB18	4.93428	0.796093
ZBTB2	5.8174	0.696678
ZBTB20	7.70495	0.971279
ZBTB21	7.51466	0.694476
ZBTB24	5.50062	0.510356
ZBTB25	5.96081	0.612972
ZBTB26	5.67643	0.734727
ZBTB33	5.59831	0.433533
ZBTB34	5.45681	0.62559
ZBTB38	9.19773	0.623242
ZBTB4	7.44503	0.509698
ZBTB40	6.14782	0.536353
ZBTB41	5.031	0.867305
ZBTB43	6.65646	0.636371
ZBTB44	6.20145	0.56708
ZBTB48	5.92872	0.437235
ZBTB5	6.97939	0.516958
ZBTB8A	6.08553	1.55468
ZBTB8OS	8.58509	0.494515
ZBTB9	5.28472	0.484975
ZC2HC1A	4.85088	0.447599
ZC3H11A	7.74884	0.553931
ZC3H12A	7.27017	1.0192
ZC3H13	5.77893	0.426174

ZC3H14	5.59564	0.405264
ZC3H6	4.94588	0.445618
ZC3H7A	7.14053	0.530012
ZC3H8	5.72618	0.503475
ZC3HAV1	7.50531	0.49311
ZC3HC1	6.75705	0.560065
ZCCHC10	6.27842	0.597822
ZCCHC11	5.95086	0.493254
ZCCHC24	6.03934	0.590331
ZCCHC6	7.06172	0.543601
ZCCHC7	7.99453	0.667149
ZCCHC8	6.36389	0.471807
ZCCHC9	7.48899	0.520135
ZDBF2	4.80716	0.724768
ZDHHC11	5.77052	0.633644
ZDHHC11B	5.69169	0.808223
ZDHHC12	6.65011	0.463955
ZDHHC13	5.54051	0.526643
ZDHHC14	5.39596	0.714674
ZDHHC16	7.4926	0.501497
ZDHHC17	6.2805	0.501371
ZDHHC2	5.6417	0.569705
ZDHHC20	6.48221	0.455071
ZDHHC23	6.3069	0.812286
ZDHHC6	7.88623	0.474546
ZDHHC9	5.89333	0.436347
ZEB1	5.43129	0.465939
ZFAND1	8.28793	0.731825
ZFAND2A	7.46723	0.616009
ZFAND2B	6.84451	0.424819
ZFAND6	8.77834	0.629522
ZFAS1	8.58116	0.706561
ZFAT	6.77552	0.53103
ZFC3H1	6.05635	0.437941
ZFP3	5.66457	0.851399
ZFP36	10.7074	0.806254
ZFP36L1	5.07509	0.500013
ZFP36L2	7.82504	0.791968
ZFP62	7.94704	0.70573
ZFP90	5.18486	0.438072
ZFP91	7.90873	0.464177
ZFR	7.00262	0.549852
ZFYVE21	5.58322	0.476313
ZFYVE26	5.41256	0.469818
ZGPAT	7.10246	0.442676
ZHX1	5.70163	0.718348
ZHX2	6.18175	0.544421

ZKSCAN1	6.45281	0.540247
ZKSCAN4	5.31111	0.501601
ZKSCAN8	5.3223	0.416009
ZMAT1	4.8734	0.727267
ZMAT2	8.31266	0.525818
ZMAT3	6.64403	0.765421
ZMIZ1	5.24702	0.517891
ZMPSTE24	9.26456	0.525881
ZMYM4	6.62629	0.501944
ZMYM6NB	7.7141	0.727956
ZMYND19	6.97531	0.53033
ZMYND8	6.51181	0.438429
ZNF10	5.16357	0.524701
ZNF101	5.57873	0.620648
ZNF106	6.86322	0.522753
ZNF12	7.1714	0.612113
ZNF121	8.21711	0.897224
ZNF124	4.92019	0.552064
ZNF131	5.97376	0.428311
ZNF134	5.82365	0.538702
ZNF136	7.22388	0.604705
ZNF14	5.63452	0.575413
ZNF140	6.0678	0.823614
ZNF143	6.41353	0.560413
ZNF146	8.12397	0.604341
ZNF177	5.01762	0.930809
ZNF184	6.07004	0.721755
ZNF185	5.29888	0.694648
ZNF189	7.76363	0.687294
ZNF195	6.81357	0.777122
ZNF20	5.22395	0.459492
ZNF200	5.05966	0.597635
ZNF202	5.08583	0.505905
ZNF207	7.07622	0.441951
ZNF211	6.5806	0.514519
ZNF215	6.18139	0.638601
ZNF217	6.83808	1.19807
ZNF22	7.81289	0.660207
ZNF222	5.27913	0.637499
ZNF224	5.45983	0.457407
ZNF227	5.43719	0.899149
ZNF23	5.48482	0.645312
ZNF232	6.13336	0.801598
ZNF236	6.25693	0.474321
ZNF24	6.52507	0.445999
ZNF251	5.12203	0.448655
ZNF26	5.2962	0.566217

ZNF260	4.90829	0.812842
ZNF264	5.38253	0.422023
ZNF266	7.65845	0.870387
ZNF274	7.02408	0.603552
ZNF275	9.19191	0.882044
ZNF277	5.51499	0.453875
ZNF280B	4.84913	0.474569
ZNF281	7.86457	0.755461
ZNF286B	5.48451	0.628599
ZNF296	5.90312	0.53059
ZNF302	6.42571	0.741336
ZNF304	4.89091	0.606201
ZNF317	5.91765	0.508
ZNF318	5.64309	0.431734
ZNF32	6.80716	0.67642
ZNF320	6.0387	0.511681
ZNF322	5.82974	0.710738
ZNF324	6.18831	0.412926
ZNF326	4.85121	0.404782
ZNF329	5.34969	1.00497
ZNF330	7.32544	0.469453
ZNF331	7.04897	0.876585
ZNF34	5.17972	0.402556
ZNF35	5.7784	0.420756
ZNF354A	6.05364	0.569447
ZNF367	5.51072	1.05686
ZNF37A	5.72669	0.637281
ZNF37BP	5.11235	0.504601
ZNF395	6.19806	0.607279
ZNF398	6.08766	0.425489
ZNF410	7.39087	0.541975
ZNF419	6.59701	0.404435
ZNF420	5.45706	0.753054
ZNF426	5.56137	0.765934
ZNF430	5.1063	0.522269
ZNF432	6.74356	0.955921
ZNF45	5.64485	0.608603
ZNF451	6.01526	0.420644
ZNF510	5.36918	0.411361
ZNF512	7.0526	0.604134
ZNF512B	6.16532	0.505857
ZNF514	5.26641	0.798977
ZNF518A	5.73081	0.446849
ZNF526	4.9245	0.437042
ZNF532	6.29888	0.81771
ZNF548	5.7291	0.420574
ZNF550	5.33255	0.521238

ZNF558	7.02263	1.04475
ZNF559	6.52618	1.15287
ZNF559-ZNF177	5.01762	0.930809
ZNF561	5.58187	0.665398
ZNF562	5.42262	0.429142
ZNF564	5.58485	0.533198
ZNF565	5.72822	0.650847
ZNF574	6.18337	0.439479
ZNF580	7.56068	0.576997
ZNF581	7.07843	0.709071
ZNF587	6.00647	0.452913
ZNF587B	6.44368	0.613639
ZNF593	7.84771	0.599266
ZNF600	6.50961	1.23709
ZNF615	5.37784	0.578015
ZNF622	7.86067	0.567377
ZNF625	4.80744	0.406378
ZNF625-ZNF20	5.42103	0.495959
ZNF630	5.293	0.505922
ZNF638	7.44098	0.515157
ZNF638-IT1	7.19746	0.628489
ZNF639	6.27328	0.612148
ZNF644	5.95444	0.697317
ZNF652	7.2074	0.59357
ZNF655	7.35691	0.516177
ZNF665	7.11138	0.427779
ZNF669	5.68348	0.41574
ZNF689	5.76134	0.477501
ZNF691	5.46762	0.444498
ZNF692	7.15102	0.55488
ZNF697	5.01791	0.54835
ZNF700	6.86051	0.649694
ZNF706	8.71219	0.609789
ZNF711	5.78697	1.20744
ZNF721	7.65176	0.421383
ZNF764	5.11915	0.494226
ZNF765	5.13228	0.40444
ZNF766	6.9005	0.572459
ZNF767P	6.34694	0.532048
ZNF770	6.19324	0.450339
ZNF776	6.81118	0.763007
ZNF786	5.21849	0.445729
ZNF791	8.87412	0.621822
ZNF800	6.4062	0.435692
ZNF804A	5.19475	1.10643
ZNF805	5.40008	0.485012
ZNF827	6.47931	0.586745

ZNF830	6.97804	0.482733
ZNF84	5.59687	0.575104
ZNF844	5.3085	0.483387
ZNF862	6.03389	0.473199
ZNFX1	6.68674	0.552443
ZNHIT1	8.30381	0.47928
ZNHIT3	7.50965	0.581703
ZNHIT6	5.0327	0.429739
ZNRF3	4.84717	0.692537
ZP3	6.10724	0.772488
ZPR1	7.18842	0.52562
ZRANB1	6.33898	0.423083
ZRANB2	8.36358	0.551517
ZRSR1	5.06115	0.417653
ZRSR2	7.00416	0.456443
ZSCAN16	5.80424	0.627072
ZSCAN18	5.49683	0.66152
ZSCAN21	6.97419	0.594368
ZSCAN26	5.82009	0.525315
ZSCAN29	6.84841	0.515627
ZSWIM6	7.95713	0.926783
ZUFSP	6.18671	0.729159
ZW10	7.0272	0.56122
ZWILCH	6.50183	0.967975
ZWINT	6.86796	1.60078
ZYG11B	6.51443	0.587287
ZYX	6.09952	0.463719
ZZZ3	6.53467	0.466755

**Supplementary Table 3**. Cis-CNV regulated genes among informative genes that were included in the construction of M3CN.	
CNV nodes	cis correlation
cnv_AACS	0.405309
cnv_AAGAB	0.486504
cnv_AARS	0.464855
cnv_AASDHPPT	0.653572
cnv_ABCB10	0.581071
cnv_ABCC10	0.468254
cnv_ABCC5	0.433533
cnv_ABCD3	0.448692
cnv_ABCF1	0.508879
cnv_ABHD12	0.304139
cnv_ABHD14A-ACY1	0.30642
cnv_ABHD17A	0.347014
cnv_ABHD17B	0.278979
cnv_ABHD17C	0.394307
cnv_ABHD2	0.351613
cnv_ABHD3	0.450606
cnv_ABRACL	0.366501
cnv_ABT1	0.301709
cnv_ACAD8	0.481047
cnv_ACAD9	0.304929
cnv_ACADM	0.272964
cnv_ACADVL	0.494441
cnv_ACAT2	0.349605
cnv_ACBD3	0.645312
cnv_ACBD6	0.640252
cnv_ACCS	0.298495
cnv_ACD	0.295316
cnv_ACIN1	0.300399
cnv_ACLY	0.397286
cnv_ACOT1	0.472335
cnv_ACOT13	0.376645
cnv_ACOT2	0.470682
cnv_ACOT9	0.273689
cnv_ACP2	0.467623
cnv_ACP6	0.562524
cnv_ACTG1P4	0.378793
cnv_ACTL6A	0.265642
cnv_ACTR10	0.5203
cnv_ACTR2	0.325967
cnv_ACTR3	0.347733
cnv_ACTR3B	0.44066
cnv_ACTR5	0.422306

cnv_ACTR6	0.288353
cnv_ACYP1	0.344065
cnv_ADA	0.349232
cnv_ADAM10	0.37409
cnv_ADAM17	0.228357
cnv_ADAM19	0.288286
cnv_ADAM28	0.375772
cnv_ADAMTS15	0.346596
cnv_ADAR	0.554624
cnv_ADAT1	0.481875
cnv_ADAT2	0.388267
cnv_ADCY7	0.465385
cnv_ADIPOR1	0.515931
cnv_ADPGK	0.543499
cnv_ADRM1	0.388445
cnv_ADSS	0.576519
cnv_AEN	0.457536
cnv_AFF4	0.432651
cnv_AFTPH	0.282892
cnv_AGA	0.274674
cnv_AGFG1	0.282371
cnv_AGK	0.424871
cnv_AGL	0.413617
cnv_AGO2	0.441181
cnv_AGO3	0.335839
cnv_AGPAT1	0.389216
cnv_AGPAT5	0.35451
cnv_AGTPBP1	0.502839
cnv_AHCYL1	0.558452
cnv_AHI1	0.560647
cnv_AIDA	0.546842
cnv_AIFM1	0.469096
cnv_AIG1	0.591005
cnv_AIMP2	0.498398
cnv_AIP	0.474771
cnv_AK3	0.514163
cnv_AK6	0.428984
cnv_AKAP11	0.555876
cnv_AKAP13	0.380256
cnv_AKAP2	0.484705
cnv_AKAP9	0.448229
cnv_AKIRIN1	0.284807
cnv_AKIRIN2	0.401915
cnv_AKR1A1	0.42493
cnv_AKR1B1	0.214743
cnv_AKR7A2	0.412195
cnv_AKT1S1	0.330677

cnv_AKTIP	0.432998
cnv_ALAS1	0.426586
cnv_ALDH16A1	0.298584
cnv_ALDH6A1	0.500476
cnv_ALDH9A1	0.550599
cnv_ALDOA	0.50371
cnv_ALG11	0.309505
cnv_ALG13	0.538801
cnv_ALG14	0.426565
cnv_ALG2	0.350611
cnv_ALG3	0.471373
cnv_ALG5	0.517556
cnv_ALG6	0.271196
cnv_ALG9	0.532258
cnv_ALKBH3	0.490069
cnv_ALKBH5	0.379945
cnv_ALKBH8	0.610078
cnv_AMD1	0.459699
cnv_AMMECR1	0.399989
cnv_AMPD2	0.473225
cnv_AMY1A	0.333749
cnv_AMY1B	0.333749
cnv_AMY2A	0.333749
cnv_AMY2B	0.378793
cnv_AMZ2	0.427645
cnv_AMZ2P1	0.3774
cnv_ANAPC15	0.461484
cnv_ANAPC4	0.376365
cnv_ANAPC5	0.492902
cnv_ANG	0.221263
cnv_ANGEL2	0.570567
cnv_ANKHD1-EIF4EBP3	0.462344
cnv_ANKIB1	0.475869
cnv_ANKRA2	0.34253
cnv_ANKRD12	0.396824
cnv_ANKRD13C	0.445858
cnv_ANKRD17	0.404129
cnv_ANKRD27	0.227059
cnv_ANKRD36BP2	0.255014
cnv_ANKRD46	0.446084
cnv_ANKRD49	0.603328
cnv_ANKS1A	0.341102
cnv_ANP32A	0.507631
cnv_ANP32B	0.469854
cnv_ANP32E	0.727473
cnv_ANTXR2	0.216197
cnv_ANXA11	0.203579

cnv_ANXA2R	0.360475
cnv_ANXA5	0.3049
cnv_ANXA7	0.369753
cnv_AP1G1	0.511954
cnv_AP1M1	0.31323
cnv_AP1S1	0.457525
cnv_AP2B1	0.267871
cnv_AP2M1	0.389855
cnv_AP2S1	0.30415
cnv_AP3M1	0.182804
cnv_AP5M1	0.560423
cnv_APBA3	0.282188
cnv_APBB1IP	0.224066
cnv_APBB3	0.429259
cnv_APC	0.551786
cnv_APH1A	0.683731
cnv_API5	0.410824
cnv_APITD1-CORT	0.345588
cnv_APMAP	0.493418
cnv_APOBEC3B	0.190053
cnv_APOBEC3C	0.370332
cnv_APOBEC3G	0.349744
cnv_APOL6	0.231585
cnv_APPL1	0.399038
cnv_APPL2	0.282005
cnv_AQR	0.47707
cnv_ARCN1	0.540558
cnv_ARF3	0.402742
cnv_ARF4	0.358719
cnv_ARF5	0.470993
cnv_ARF6	0.362496
cnv_ARFGAP3	0.490142
cnv_ARFGEF1	0.516551
cnv_ARFIP1	0.228285
cnv_ARFIP2	0.445477
cnv_ARGLU1	0.725251
cnv_ARHGAP17	0.312113
cnv_ARHGAP18	0.381854
cnv_ARHGAP21	0.390021
cnv_ARHGAP30	0.418658
cnv_ARHGAP4	0.376273
cnv_ARHGDIB	0.296622
cnv_ARHGEF2	0.579049
cnv_ARHGEF3	0.241577
cnv_ARHGEF6	0.395561
cnv_ARID2	0.253307
cnv_ARID4A	0.38919

cnv_ARIH1	0.555851
cnv_ARL1	0.55449
cnv_ARL14EP	0.367956
cnv_ARL2	0.455453
cnv_ARL2BP	0.457977
cnv_ARL5A	0.364702
cnv_ARL5B	0.26312
cnv_ARL6IP1	0.462064
cnv_ARL6IP6	0.215659
cnv_ARL8A	0.406191
cnv_ARL8B	0.385673
cnv_ARMC1	0.511708
cnv_ARMC10	0.566215
cnv_ARMC8	0.359516
cnv_ARMCX3	0.34887
cnv_ARMCX5	0.400676
cnv_ARMCX5-GPRASP2	0.246432
cnv_ARPC1A	0.521622
cnv_ARPC3	0.413602
cnv_ARPC5	0.575343
cnv_ARPC5L	0.374803
cnv_ARPP19	0.497713
cnv_ARRDC3	0.27343
cnv_ASAH1	0.597046
cnv_ASB16-AS1	0.353189
cnv_ASB8	0.392433
cnv_ASCC2	0.389062
cnv_ASCC3	0.4888
cnv_ASH1L	0.481618
cnv_ASH1L-AS1	0.496994
cnv_ASH2L	0.502969
cnv_ASL	0.362304
cnv_ASNA1	0.394943
cnv_ASNS	0.37684
cnv_ASXL1	0.434525
cnv_ATF1	0.274612
cnv_ATF2	0.313197
cnv_ATF6	0.398463
cnv_ATF7IP2	0.2225
cnv_ATG12	0.496483
cnv_ATG14	0.515615
cnv_ATG2B	0.486442
cnv_ATG3	0.411119
cnv_ATG4A	0.44413
cnv_ATM	0.447034
cnv_ATOX1	0.249684
cnv_ATP10B	0.287214

cnv_ATP10D	0.52826
cnv_ATP11B	0.4774
cnv_ATP11C	0.434268
cnv_ATP13A1	0.315413
cnv_ATP13A3	0.387799
cnv_ATP2B4	0.360492
cnv_ATP2C1	0.270936
cnv_ATP5D	0.397006
cnv_ATP5G1	0.40924
cnv_ATP5I	0.408998
cnv_ATP5J	0.44534
cnv_ATP5L	0.641732
cnv_ATP6AP1	0.544612
cnv_ATP6AP2	0.215467
cnv_ATP6V0A1	0.220693
cnv_ATP6V0B	0.496212
cnv_ATP6V0C	0.437786
cnv_ATP6V0D1	0.522573
cnv_ATP6V0E1	0.512692
cnv_ATP6V1A	0.445755
cnv_ATP6V1B2	0.471956
cnv_ATP6V1C1	0.595015
cnv_ATP6V1E1	0.465121
cnv_ATP6V1E2	0.254241
cnv_ATP6V1F	0.653541
cnv_ATP6V1G1	0.390482
cnv_ATP6V1G2-DDX39B	0.376443
cnv_ATP8A2	0.316263
cnv_ATP8B2	0.607331
cnv_ATPAF1	0.506486
cnv_ATRAID	0.377755
cnv_ATXN10	0.366215
cnv_ATXN1L	0.591556
cnv_ATXN2	0.387399
cnv_AUH	0.568279
cnv_AUP1	0.292747
cnv_AURKAPS1	0.68623
cnv_AVEN	0.596711
cnv_AZIN1	0.557545
cnv_B4GALT4	0.339269
cnv_B4GALT5	0.340755
cnv_BABAM1	0.543188
cnv_BAG1	0.53513
cnv_BAG4	0.507985
cnv_BAG5	0.407979
cnv_BAK1	0.37797
cnv_BANF1	0.345846

cnv_BANK1	0.307555
cnv_BARD1	0.222059
cnv_BBS2	0.35108
cnv_BBS4	0.338813
cnv_BCAP31	0.456244
cnv_BCAR3	0.318084
cnv_BCAS2	0.596524
cnv_BCAS3	0.337439
cnv_BCKDHB	0.558297
cnv_BCKDK	0.480135
cnv_BCL10	0.436003
cnv_BCL2	0.396792
cnv_BCL2L2-PABPN1	0.433371
cnv_BCL7B	0.521703
cnv_BCL7C	0.35946
cnv_BCLAF1	0.463521
cnv_BCR	0.339351
cnv_BDP1	0.324818
cnv_BET1L	0.404683
cnv_BEX2	0.244539
cnv_BICD1	0.320944
cnv_BICD2	0.27562
cnv_BIRC2	0.787944
cnv_BLCAP	0.409316
cnv_BLNK	0.294264
cnv_BLOC1S1	0.372886
cnv_BLOC1S4	0.261505
cnv_BLOC1S5	0.421691
cnv_BLOC1S6	0.421542
cnv_BNIP2	0.383847
cnv_BNIP3	0.254107
cnv_BNIP3L	0.633795
cnv_BOD1	0.552383
cnv_BOD1L1	0.352562
cnv_BOLA1	0.576298
cnv_BOLA2	0.465602
cnv_BORA	0.425961
cnv_BPGM	0.353885
cnv_BPNT1	0.673442
cnv_BRAF	0.389462
cnv_BRAT1	0.512766
cnv_BRCA1	0.392828
cnv_BRCC3	0.471427
cnv_BRD2	0.463743
cnv_BRI3BP	0.206726
cnv_BRIX1	0.46991
cnv_BRMS1	0.569377

cnv_BRMS1L	0.423011
cnv_BROX	0.636173
cnv_BSDC1	0.511394
cnv_BST2	0.365823
cnv_BTBD1	0.43002
cnv_BTBD10	0.405673
cnv_BTK	0.333931
cnv_BTLA	0.388635
cnv_BTN3A1	0.28304
cnv_BUD13	0.621263
cnv_BZW2	0.425343
cnv_C11orf1	0.512309
cnv_C11orf24	0.298153
cnv_C11orf54	0.405693
cnv_C11orf57	0.665381
cnv_C11orf68	0.382576
cnv_C11orf71	0.416787
cnv_C12orf29	0.317934
cnv_C12orf4	0.354277
cnv_C12orf57	0.322273
cnv_C14orf166	0.576291
cnv_C14orf2	0.470957
cnv_C14orf28	0.326285
cnv_C15orf39	0.395081
cnv_C15orf57	0.388374
cnv_C15orf61	0.539556
cnv_C16orf54	0.289994
cnv_C16orf72	0.3775
cnv_C16orf91	0.374417
cnv_C17orf49	0.407024
cnv_C17orf75	0.23681
cnv_C19orf12	0.643554
cnv_C19orf24	0.449798
cnv_C19orf43	0.639866
cnv_C19orf48	0.344671
cnv_C19orf53	0.600281
cnv_C19orf60	0.325116
cnv_C19orf66	0.261986
cnv_C19orf70	0.472542
cnv_C1D	0.24388
cnv_C1GALT1	0.356298
cnv_C1GALT1C1	0.47729
cnv_C1orf106	0.500951
cnv_C1orf122	0.428655
cnv_C1orf131	0.522087
cnv_C1orf174	0.379989
cnv_C1orf21	0.426549

cnv_C1orf27	0.547351
cnv_C1orf43	0.611358
cnv_C1orf52	0.61147
cnv_C1orf53	0.358831
cnv_C1QTNF3-AMACR	0.43209
cnv_C20orf24	0.466394
cnv_C21orf33	0.423965
cnv_C22orf39	0.481621
cnv_C2CD2	0.25707
cnv_C2CD5	0.341795
cnv_C2orf69	0.297806
cnv_C3orf38	0.342831
cnv_C4A	0.295311
cnv_C4B	0.295311
cnv_C4orf3	0.389597
cnv_C5orf22	0.429981
cnv_C5orf24	0.453336
cnv_C5orf30	0.359403
cnv_C5orf56	0.434961
cnv_C6orf1	0.380518
cnv_C6orf120	0.497216
cnv_C6orf136	0.450984
cnv_C6orf226	0.241938
cnv_C6orf62	0.428794
cnv_C7orf31	0.454985
cnv_C7orf55-LUC7L2	0.474026
cnv_C8orf44-SGK3	0.380972
cnv_C8orf59	0.487514
cnv_C8orf76	0.563352
cnv_C8orf82	0.371885
cnv_C9orf78	0.454667
cnv_CAAP1	0.422182
cnv_CABLES1	0.254861
cnv_CACYBP	0.709723
cnv_CADM1	0.309565
cnv_CALU	0.461197
cnv_CAMK2D	0.278759
cnv_CAMLG	0.541606
cnv_CAMSAP2	0.374398
cnv_CAND1	0.3888
cnv_CAP1	0.430058
cnv_CAPN2	0.401361
cnv_CAPN7	0.283175
cnv_CAPRIN1	0.583206
cnv_CAPZA2	0.49911
cnv_CARD16	0.314882
cnv_CASC3	0.362371

cnv_CASP1	0.427293
cnv_CASP4	0.537979
cnv_CASP7	0.278277
cnv_CAST	0.555607
cnv_CAT	0.259006
cnv_CBFA2T2	0.34506
cnv_CBFB	0.465768
cnv_CBL	0.408746
cnv_CBLL1	0.294345
cnv_CBR1	0.283024
cnv_CBX1	0.425063
cnv_CBX3	0.444465
cnv_CBX5	0.274532
cnv_CBX7	0.315027
cnv_CCDC102A	0.336454
cnv_CCDC107	0.418437
cnv_CCDC115	0.207203
cnv_CCDC117	0.345491
cnv_CCDC12	0.441487
cnv_CCDC125	0.427082
cnv_CCDC126	0.336977
cnv_CCDC167	0.305124
cnv_CCDC25	0.547793
cnv_CCDC28A	0.536968
cnv_CCDC50	0.289314
cnv_CCDC51	0.285772
cnv_CCDC59	0.482773
cnv_CCDC6	0.327137
cnv_CCDC69	0.364861
cnv_CCDC84	0.404193
cnv_CCDC86	0.136227
cnv_CCM2	0.453213
cnv_CCNC	0.618138
cnv_CCND1	0.332941
cnv_CCNDBP1	0.682487
cnv_CCNE1	0.435974
cnv_CCNE2	0.285106
cnv_CCNG1	0.493846
cnv_CCNH	0.426406
cnv_CCNI	0.597624
cnv_CCNT1	0.332576
cnv_CCNYL1	0.348628
cnv_CCP110	0.534336
cnv_CCPG1	0.295178
cnv_CCT3	0.622443
cnv_CCT5	0.33544
cnv_CCT6A	0.415161

cnv_CCT6P1	0.405785
cnv_CCT6P3	0.442842
cnv_CCT8	0.435807
cnv_CCZ1B	0.557163
cnv_CD164	0.525486
cnv_CD27	0.276572
cnv_CD274	0.324336
cnv_CD320	0.367046
cnv_CD44	0.296733
cnv_CD46	0.487203
cnv_CD53	0.425025
cnv_CD55	0.467198
cnv_CD63	0.38145
cnv_CDC23	0.289329
cnv_CDC25B	0.224303
cnv_CDC26	0.441106
cnv_CDC27	0.347604
cnv_CDC37L1	0.48483
cnv_CDC40	0.423962
cnv_CDC42	0.317016
cnv_CDC42SE1	0.600326
cnv_CDC42SE2	0.421823
cnv_CDC73	0.605294
cnv_CDCA7L	0.395829
cnv_CDIPT	0.595937
cnv_CDK2	0.274922
cnv_CDK2AP1	0.2708
cnv_CDK4	0.351556
cnv_CDK5	0.365047
cnv_CDK5RAP3	0.38947
cnv_CDK7	0.396077
cnv_CDK8	0.36008
cnv_CDKN1B	0.479395
cnv_CDKN2AIP	0.46401
cnv_CDRT4	0.308099
cnv_CDV3	0.402652
cnv_CDYL	0.366881
cnv_CEBPB	0.312252
cnv_CEBPZOS	0.374786
cnv_CECR1	0.351044
cnv_CENPB	0.346848
cnv_CENPC	0.280614
cnv_CENPJ	0.358981
cnv_CENPM	0.257993
cnv_CEP120	0.357135
cnv_CEP170	0.512345
cnv_CEP192	0.488529

cnv_CEP290	0.315141
cnv_CEP350	0.531447
cnv_CEP57	0.580877
cnv_CEP72	0.394335
cnv_CEP85L	0.422272
cnv_CEPT1	0.47931
cnv_CERK	0.311858
cnv_CERS2	0.739964
cnv_CETN2	0.484827
cnv_CFL2	0.32885
cnv_CGGBP1	0.278709
cnv_CGRRF1	0.558313
cnv_CHCHD2	0.474085
cnv_CHCHD3	0.528392
cnv_CHCHD4	0.423287
cnv_CHCHD7	0.383448
cnv_CHD1	0.389463
cnv_CHD1L	0.692034
cnv_CHD2	0.333817
cnv_CHD8	0.539641
cnv_CHD9	0.50353
cnv_CHEK1	0.383855
cnv_CHIC2	0.262112
cnv_CHKA	0.352134
cnv_CHMP1B	0.518359
cnv_CHMP2B	0.2745
cnv_CHMP4A	0.586072
cnv_CHMP4C	0.319666
cnv_CHMP5	0.336836
cnv_CHMP7	0.498365
cnv_CHORDC1	0.426272
cnv_CHP1	0.504599
cnv_CHPF	0.300688
cnv_CHPF2	0.0877621
cnv_CHRAC1	0.51262
cnv_CHRNB1	0.321683
cnv_CHST12	0.421222
cnv_CHST15	0.327164
cnv_CHSY3	0.411708
cnv_CHTOP	0.480923
cnv_CHURC1	0.516284
cnv_CIB1	0.316067
cnv_CIPC	0.473552
cnv_CITED2	0.437509
cnv_CKAP4	0.424659
cnv_CKAP5	0.338555
cnv_CKLF	0.482982

cnv_CKLF-CMTM1	0.478744
cnv_CKMT2-AS1	0.307932
cnv_CKS1B	0.520835
cnv_CKS2	0.378475
cnv_CLASP2	0.269425
cnv_CLCC1	0.630459
cnv_CLCN3	0.365334
cnv_CLDND1	0.28851
cnv_CLIC1	0.444077
cnv_CLIC4	0.266996
cnv_CLINT1	0.347494
cnv_CLK2	0.526105
cnv_CLK4	0.448596
cnv_CLMN	0.485753
cnv_CLN3	0.329864
cnv_CLN5	0.567015
cnv_CLNS1A	0.674269
cnv_CLOCK	0.394111
cnv_CLPP	0.458407
cnv_CLPTM1	0.319028
cnv_CLPX	0.470421
cnv_CLSTN1	0.312566
cnv_CMC1	0.314836
cnv_CMC2	0.51096
cnv_CMC4	0.643322
cnv_CMSS1	0.417864
cnv_CMTR1	0.38913
cnv_CNEP1R1	0.265879
cnv_CNOT10	0.336094
cnv_CNOT11	0.376184
cnv_CNOT6L	0.449088
cnv_CNOT7	0.633039
cnv_CNOT8	0.333645
cnv_CNPPD1	0.318051
cnv_CNPY3	0.521233
cnv_COA4	0.471029
cnv_COA6	0.607776
cnv_COG2	0.677992
cnv_COG3	0.582871
cnv_COG5	0.331699
cnv_COG7	0.513684
cnv_COIL	0.345076
cnv_COL24A1	0.316158
cnv_COLCA2	0.309228
cnv_COLGALT1	0.361206
cnv_COMMD10	0.475785
cnv_COMMD2	0.356159

cnv_COMMD9	0.425542
cnv_COPA	0.696179
cnv_COPS2	0.562307
cnv_COPS3	0.35432
cnv_COPS5	0.454797
cnv_COPS6	0.660388
cnv_COPS7A	0.407912
cnv_COQ10A	0.338358
cnv_COQ2	0.186655
cnv_COQ4	0.474996
cnv_COQ5	0.362888
cnv_COX14	0.334555
cnv_COX15	0.237541
cnv_COX16	0.536461
cnv_COX17	0.322661
cnv_COX20	0.516372
cnv_COX5A	0.630498
cnv_COX6B1	0.367997
cnv_COX6C	0.465747
cnv_COX7A2	0.45931
cnv_COX7C	0.684745
cnv_COX8A	0.360574
cnv_CPEB4	0.417632
cnv_CPNE1	0.437439
cnv_CPNE3	0.44135
cnv_CPOX	0.278528
cnv_CPT1A	0.3082
cnv_CPT2	0.46477
cnv_CRBN	0.434841
cnv_CREB1	0.265665
cnv_CREB3	0.371766
cnv_CREB3L4	0.602312
cnv_CREBL2	0.491234
cnv_CREBRF	0.313786
cnv_CREBZF	0.483706
cnv_CREG1	0.281462
cnv_CRIPAK	0.467673
cnv_CRIPT	0.342205
cnv_CRK	0.470919
cnv_CRKL	0.168264
cnv_CRTC3	0.338026
cnv_CRYL1	0.388508
cnv_CRYZ	0.302713
cnv_CRYZL1	0.504651
cnv_CSDE1	0.691141
cnv_CSE1L	0.414471
cnv_CSGALNACT1	0.504485

cnv_CSGALNACT2	0.244885
cnv_CSK	0.299533
cnv_CSNK1A1	0.526158
cnv_CSNK1G3	0.364134
cnv_CSNK2A1	0.399017
cnv_CSNK2A2	0.410144
cnv_CSPP1	0.447205
cnv_CSRP1	0.384015
cnv_CSTF2T	0.246915
cnv_CSTF3	0.501337
cnv_CTBS	0.485083
cnv_CTDNEP1	0.418992
cnv_CTDSP2	0.321146
cnv_CTDSPL2	0.404307
cnv_CTH	0.300575
cnv_CTNNB1	0.250513
cnv_CTNS	0.304639
cnv_CTR9	0.291681
cnv_CTSA	0.337601
cnv_CTSB	0.456027
cnv_CTSC	0.483637
cnv_CTSK	0.50118
cnv_CTSO	0.450225
cnv_CTSZ	0.233077
cnv_CUL4B	0.471838
cnv_CUL5	0.591445
cnv_CUTA	0.316639
cnv_CUTC	0.265279
cnv_CUX1	0.35954
cnv_CWC15	0.698793
cnv_CWC22	0.282735
cnv_CWC27	0.42402
cnv_CXorf40A	0.629072
cnv_CXorf40B	0.654354
cnv_CXXC1	0.337309
cnv_CXXC4	0.209305
cnv_CXXC5	0.305934
cnv_CYB561A3	0.342973
cnv_CYB5A	0.268113
cnv_CYB5D1	0.374124
cnv_CYB5R3	0.380257
cnv_CYB5R4	0.304444
cnv_CYBB	0.18628
cnv_CYC1	0.424255
cnv_CYCS	0.36572
cnv_CYLD	0.439513
cnv_CYP20A1	0.212453

cnv_CYP2R1	0.296133
cnv_CYP51A1	0.362263
cnv_CYSTM1	0.424719
cnv_CYTH1	0.247322
cnv_DAAM1	0.328376
cnv_DAD1	0.341895
cnv_DALRD3	0.332367
cnv_DAPP1	0.418306
cnv_DARS2	0.705846
cnv_DBF4	0.513345
cnv_DBNL	0.341988
cnv_DCAF10	0.401379
cnv_DCAF12	0.294535
cnv_DCAF13	0.578669
cnv_DCAF16	0.308848
cnv_DCP1A	0.277964
cnv_DCP2	0.38936
cnv_DCPS	0.480983
cnv_DCTD	0.509109
cnv_DCTN1	0.284457
cnv_DCTN3	0.582602
cnv_DCTN4	0.507864
cnv_DCTN6	0.618866
cnv_DCUN1D1	0.407471
cnv_DCUN1D2	0.319974
cnv_DCUN1D4	0.245039
cnv_DCUN1D5	0.6187
cnv_DDB2	0.296604
cnv_DDHD1	0.416097
cnv_DDHD2	0.546226
cnv_DDIAS	0.366889
cnv_DDIT3	0.344536
cnv_DDOST	0.487995
cnv_DDRGK1	0.358362
cnv_DDX17	0.329447
cnv_DDX18	0.421801
cnv_DDX24	0.586987
cnv_DDX39A	0.446099
cnv_DDX39B	0.376443
cnv_DDX41	0.38904
cnv_DDX46	0.355936
cnv_DDX47	0.453298
cnv_DDX5	0.190937
cnv_DDX50	0.33061
cnv_DDX58	0.45918
cnv_DDX59	0.557655
cnv_DECR1	0.379855

cnv_DEDD	0.591718
cnv_DEDD2	0.425356
cnv_DEF6	0.403183
cnv_DEGS1	0.55345
cnv_DENND1B	0.428172
cnv_DENND2D	0.409141
cnv_DENND4A	0.55189
cnv_DENND4B	0.481827
cnv_DENND5B	0.338057
cnv_DENND6A	0.373728
cnv_DEPTOR	0.296988
cnv_DERA	0.42886
cnv_DERL1	0.422329
cnv_DERL2	0.564199
cnv_DET1	0.321635
cnv_DEXI	0.356611
cnv_DFFB	0.420713
cnv_DGAT1	0.337231
cnv_DGCR6	0.426306
cnv_DGKA	0.243682
cnv_DGKQ	0.352269
cnv_DHCR7	0.258217
cnv_DHPS	0.541672
cnv_DHRS13	0.358919
cnv_DHRS4	0.421343
cnv_DHRS4L2	0.416551
cnv_DHTKD1	0.314602
cnv_DHX16	0.54113
cnv_DHX29	0.445761
cnv_DHX33	0.377811
cnv_DHX40	0.452639
cnv_DHX9	0.480576
cnv_DIABLO	0.307515
cnv_DIEXF	0.429075
cnv_DIMT1	0.420131
cnv_DIS3	0.532867
cnv_DIS3L	0.524829
cnv_DKC1	0.444263
cnv_DKFZP586I1420	0.330636
cnv_DLAT	0.523907
cnv_DLD	0.444269
cnv_DLEU1	0.37138
cnv_DLG1	0.334746
cnv_DLST	0.52287
cnv_DMAP1	0.424751
cnv_DMRT2	0.242439
cnv_DMTF1	0.402398

cnv_DMXL1	0.408633
cnv_DMXL2	0.324619
cnv_DNAAF2	0.24005
cnv_DNAJA1	0.420285
cnv_DNAJA2	0.380011
cnv_DNAJA3	0.437082
cnv_DNAJB14	0.299986
cnv_DNAJB4	0.419416
cnv_DNAJB6	0.265393
cnv_DNAJC14	0.36503
cnv_DNAJC15	0.526888
cnv_DNAJC17	0.324452
cnv_DNAJC18	0.540199
cnv_DNAJC19	0.441024
cnv_DNAJC24	0.395026
cnv_DNAJC3	0.454425
cnv_DNASE1L1	0.554814
cnv_DNASE2	0.448684
cnv_DNTTIP1	0.494514
cnv_DNTTIP2	0.419681
cnv_DOCK11	0.320254
cnv_DOCK7	0.380859
cnv_DOCK8	0.356726
cnv_DOK3	0.383861
cnv_DOLK	0.454682
cnv_DONSON	0.376455
cnv_DPAGT1	0.43277
cnv_DPF2	0.551392
cnv_DPH3	0.389037
cnv_DPM1	0.368396
cnv_DPM2	0.413063
cnv_DPM3	0.540182
cnv_DPP3	0.40934
cnv_DPP8	0.462703
cnv_DPY19L4	0.415545
cnv_DRAM2	0.588872
cnv_DRAP1	0.281191
cnv_DRG1	0.393981
cnv_DROSHA	0.527934
cnv_DSN1	0.382996
cnv_DTL	0.436419
cnv_DTNBP1	0.464287
cnv_DTWD1	0.424868
cnv_DTX3L	0.33411
cnv_DUS4L	0.470849
cnv_DUSP11	0.281223
cnv_DUSP12	0.688586

cnv_DUSP14	0.245912
cnv_DUSP22	0.323775
cnv_DUSP23	0.3494
cnv_DUSP28	0.354721
cnv_DUSP7	0.300115
cnv_DUT	0.285434
cnv_DXO	0.374724
cnv_DYNC1LI1	0.459837
cnv_DYNLT1	0.560864
cnv_DYNLT3	0.261809
cnv_DYRK4	0.431173
cnv_DYX1C1-CCPG1	0.321277
cnv_DZIP3	0.393795
cnv_E2F3	0.344121
cnv_EAF1	0.401101
cnv_EBAG9	0.422903
cnv_EBPL	0.448649
cnv_ECD	0.319662
cnv_ECHDC1	0.494891
cnv_ECI2	0.360201
cnv_ECSIT	0.506725
cnv_EDEM2	0.315681
cnv_EDEM3	0.604456
cnv_EED	0.534727
cnv_EEF1E1	0.4456
cnv_EEF1E1-BLOC1S5	0.376508
cnv_EEF2	0.481303
cnv_EEF2K	0.326789
cnv_EFCAB7	0.285239
cnv_EFNA4	0.285977
cnv_EFR3A	0.416638
cnv_EGLN1	0.677046
cnv_EHD3	0.216484
cnv_EI24	0.483517
cnv_EID1	0.518664
cnv_EIF1AD	0.369366
cnv_EIF1AX	0.356381
cnv_EIF1B	0.263392
cnv_EIF2A	0.460324
cnv_EIF2AK1	0.590642
cnv_EIF2AK4	0.347899
cnv_EIF2B2	0.400705
cnv_EIF2D	0.446865
cnv_EIF2S1	0.469471
cnv_EIF3B	0.587512
cnv_EIF3F	0.585945
cnv_EIF3G	0.587023

cnv_EIF3J	0.509233
cnv_EIF3J-AS1	0.316842
cnv_EIF3M	0.487112
cnv_EIF4E3	0.234303
cnv_EIF4G1	0.380027
cnv_EIF5	0.529968
cnv_EIF5A	0.299447
cnv_EIF6	0.39491
cnv_ELF1	0.505164
cnv_ELF2	0.353032
cnv_ELF4	0.380691
cnv_ELFN1-AS1	0.311244
cnv_ELL2	0.487732
cnv_ELMO1	0.437127
cnv_ELOVL7	0.464334
cnv_ELP3	0.443974
cnv_ELP4	0.451526
cnv_ELP5	0.482153
cnv_EMB	0.293367
cnv_EMC2	0.43891
cnv_EMC6	0.509633
cnv_EMC7	0.490066
cnv_EMC8	0.658096
cnv_EMC9	0.49806
cnv_EMD	0.500009
cnv_EME1	0.344211
cnv_EMG1	0.427865
cnv_EML6	0.26212
cnv_ENDOG	0.418428
cnv_ENKD1	0.314315
cnv_ENOSF1	0.298123
cnv_ENSA	0.697636
cnv_ENTPD4	0.658345
cnv_ENY2	0.542499
cnv_EP300	0.326687
cnv_EPB41L4A	0.654249
cnv_EPB41L4A-AS1	0.601895
cnv_EPC1	0.363379
cnv_EPHX2	0.264087
cnv_EPM2AIP1	0.279816
cnv_EPRS	0.697429
cnv_EPS15	0.464711
cnv_EPSTI1	0.364201
cnv_ERAP2	0.338474
cnv_ERCC1	0.53784
cnv_ERCC3	0.286555
cnv_ERCC4	0.308829

cnv_ERCC5	0.645413
cnv_ERGIC1	0.344984
cnv_ERGIC2	0.371087
cnv_ERH	0.559348
cnv_ERI3	0.376068
cnv_ERLIN2	0.54174
cnv_ERMARD	0.615533
cnv_ERMP1	0.305822
cnv_ERN1	0.264806
cnv_ERP29	0.41491
cnv_ESF1	0.223416
cnv_ESYT1	0.433362
cnv_ESYT2	0.363668
cnv_ETF1	0.497772
cnv_ETFA	0.387636
cnv_ETHE1	0.32412
cnv_ETNK1	0.339004
cnv_ETV3	0.351953
cnv_ETV6	0.301149
cnv_EWSR1	0.199687
cnv_EXD2	0.593864
cnv_EXO1	0.455998
cnv_EXOC1	0.196601
cnv_EXOC2	0.492585
cnv_EXOC5	0.396364
cnv_EXOC8	0.440456
cnv_EXOSC3	0.350261
cnv_EXOSC6	0.38001
cnv_EXOSC8	0.390095
cnv_EXT1	0.401029
cnv_EXT2	0.430049
cnv_EZH2	0.337071
cnv_EZR	0.44271
cnv_F12	0.34206
cnv_F2R	0.442246
cnv_F8	0.472105
cnv_F8A1	0.603871
cnv_FADD	0.298669
cnv_FAF2	0.38814
cnv_FAM102B	0.291383
cnv_FAM103A1	0.341367
cnv_FAM104A	0.410827
cnv_FAM111A	0.387712
cnv_FAM114A1	0.293331
cnv_FAM118B	0.618686
cnv_FAM120B	0.592729
cnv_FAM122A	0.288248

cnv_FAM122B	0.599574
cnv_FAM126B	0.234794
cnv_FAM127A	0.385752
cnv_FAM127B	0.353641
cnv_FAM129A	0.330427
cnv_FAM134A	0.332829
cnv_FAM134C	0.350345
cnv_FAM13B	0.408435
cnv_FAM149A	0.398072
cnv_FAM160A2	0.458224
cnv_FAM162A	0.366167
cnv_FAM174A	0.447402
cnv_FAM175B	0.308989
cnv_FAM177A1	0.2429
cnv_FAM179B	0.450463
cnv_FAM193A	0.403
cnv_FAM199X	0.499042
cnv_FAM206A	0.385988
cnv_FAM20B	0.535695
cnv_FAM210A	0.416098
cnv_FAM210B	0.179275
cnv_FAM213A	0.308104
cnv_FAM214A	0.586568
cnv_FAM217B	0.225894
cnv_FAM219A	0.379372
cnv_FAM220A	0.401123
cnv_FAM221A	0.436186
cnv_FAM228B	0.220848
cnv_FAM229B	0.351268
cnv_FAM32A	0.483596
cnv_FAM35A	0.145906
cnv_FAM46C	0.333409
cnv_FAM50A	0.601062
cnv_FAM53C	0.524788
cnv_FAM58A	0.47125
cnv_FAM63B	0.422913
cnv_FAM65A	0.308375
cnv_FAM72A	0.413282
cnv_FAM72C	0.481001
cnv_FAM72D	0.45419
cnv_FAM76B	0.549169
cnv_FAM8A1	0.41776
cnv_FAM96A	0.505197
cnv_FAM96B	0.571592
cnv_FAM98A	0.345615
cnv_FAM98B	0.281671
cnv_FAM98C	0.417244

cnv_FANCF	0.31182
cnv_FANCG	0.441439
cnv_FAR1	0.324418
cnv_FARSA	0.379686
cnv_FASTKD3	0.347189
cnv_FASTKD5	0.394074
cnv_FBL	0.436988
cnv_FBXL20	0.281514
cnv_FBXL4	0.450986
cnv_FBXL5	0.471911
cnv_FBXO11	0.210773
cnv_FBXO16	0.61637
cnv_FBXO22	0.667395
cnv_FBXO25	0.519175
cnv_FBXO28	0.567881
cnv_FBXO30	0.405416
cnv_FBXO33	0.458176
cnv_FBXO34	0.412185
cnv_FBXO38	0.422351
cnv_FBXO45	0.458673
cnv_FBXO46	0.319426
cnv_FBXO6	0.275601
cnv_FBXO7	0.468768
cnv_FBXW11	0.37633
cnv_FCF1	0.526471
cnv_FCHSD2	0.529269
cnv_FDFT1	0.535057
cnv_FDPS	0.672072
cnv_FDX1	0.581798
cnv_FDXACB1	0.614009
cnv_FECH	0.363481
cnv_FEM1B	0.407735
cnv_FER	0.398008
cnv_FERMT3	0.427386
cnv_FGFR1OP2	0.357839
cnv_FH	0.73244
cnv_FHOD1	0.2519
cnv_FIBP	0.420797
cnv_FICD	0.383141
cnv_FIG4	0.41718
cnv_FIP1L1	0.270462
cnv_FIS1	0.507235
cnv_FKBP11	0.396827
cnv_FKBP1A	0.338275
cnv_FKTN	0.298727
cnv_FLAD1	0.658665
cnv_FLI1	0.471316

cnv_FLJ20021	0.297108
cnv_FLJ32255	0.272707
cnv_FLNA	0.295577
cnv_FLVCR1	0.465176
cnv_FLVCR1-AS1	0.35061
cnv_FMR1	0.492763
cnv_FNBP4	0.467905
cnv_FNDC3A	0.507072
cnv_FNDC3B	0.405236
cnv_FNIP1	0.264563
cnv_FNTA	0.462281
cnv_FOS	0.403904
cnv_FOXO1	0.382215
cnv_FOXO3	0.492646
cnv_FOXRED1	0.462024
cnv_FTO	0.577153
cnv_FTSJ3	0.337792
cnv_FUBP1	0.368299
cnv_FUBP3	0.328768
cnv_FUCA2	0.457038
cnv_FUK	0.452203
cnv_FUOM	0.269186
cnv_FUS	0.471991
cnv_FUT8	0.33719
cnv_FUT8-AS1	0.340235
cnv_FXN	0.284447
cnv_FYN	0.239953
cnv_FYTTD1	0.498313
cnv_G3BP1	0.283154
cnv_G3BP2	0.357599
cnv_G6PC3	0.33316
cnv_G6PD	0.419686
cnv_GAB1	0.375232
cnv_GABARAP	0.577856
cnv_GABARAPL1	0.27566
cnv_GABARAPL2	0.626559
cnv_GABPA	0.379917
cnv_GABPB1-AS1	0.465734
cnv_GABPB2	0.460321
cnv_GALC	0.366936
cnv_GALK2	0.301096
cnv_GALNT1	0.395365
cnv_GALNT2	0.401154
cnv_GANAB	0.269406
cnv_GAPVD1	0.427138
cnv_GARS	0.475068
cnv_GART	0.540294

cnv_GAS5	0.371431
cnv_GATAD1	0.351075
cnv_GATAD2B	0.374502
cnv_GATSL2	0.268627
cnv_GBA	0.543051
cnv_GBAP1	0.584785
cnv_GBAS	0.316155
cnv_GBE1	0.2731
cnv_GBF1	0.257456
cnv_GBP1	0.226459
cnv_GBP3	0.31823
cnv_GBP5	0.324144
cnv_GCDH	0.40253
cnv_GCH1	0.344906
cnv_GCLC	0.343867
cnv_GCOM1	0.552698
cnv_GCSH	0.395839
cnv_GDAP2	0.486093
cnv_GDE1	0.395334
cnv_GDI1	0.644814
cnv_GDI2	0.323588
cnv_GFM1	0.41398
cnv_GFM2	0.372692
cnv_GFPT1	0.327926
cnv_GGCT	0.466868
cnv_GGH	0.246544
cnv_GGPS1	0.558541
cnv_GHITM	0.312958
cnv_GID4	0.409601
cnv_GIMAP2	0.369208
cnv_GINM1	0.531295
cnv_GINS1	0.203945
cnv_GKAP1	0.257567
cnv_GLA	0.432891
cnv_GLB1	0.408391
cnv_GLCCI1	0.480505
cnv_GLE1	0.443232
cnv_GLG1	0.609258
cnv_GLIPR1	0.221199
cnv_GLIS3	0.274968
cnv_GLOD4	0.367121
cnv_GLRX	0.474558
cnv_GLRX2	0.514137
cnv_GLRX5	0.46612
cnv_GLTP	0.318701
cnv_GLTSCR1L	0.395867
cnv_GLTSCR2	0.415896

cnv_GM2A	0.379649
cnv_GMCL1	0.23661
cnv_GMDS	0.50756
cnv_GMFB	0.477045
cnv_GMPPA	0.366544
cnv_GMPR2	0.497871
cnv_GNA13	0.320644
cnv_GNAI3	0.387959
cnv_GNB1	0.445662
cnv_GNB2	0.388578
cnv_GNB5	0.347464
cnv_GNE	0.276213
cnv_GNG5	0.632868
cnv_GNL3	0.396787
cnv_GNPAT	0.684482
cnv_GNPNAT1	0.260171
cnv_GNPTG	0.392705
cnv_GNS	0.366103
cnv_GOLGA1	0.347779
cnv_GOLGA4	0.385942
cnv_GOLGA5	0.457813
cnv_GOLGA6L4	0.345397
cnv_GOLGA6L5P	0.342593
cnv_GOLGA6L9	0.367506
cnv_GOLGA7	0.565629
cnv_GOLGA8CP	0.423001
cnv_GOLGA8DP	0.388852
cnv_GOLGA8EP	0.407955
cnv_GOLGA8F	0.423924
cnv_GOLGA8G	0.41609
cnv_GOLGA8N	0.427042
cnv_GOLGB1	0.270197
cnv_GOLIM4	0.296525
cnv_GOLPH3	0.253677
cnv_GOLPH3L	0.428861
cnv_GOLT1B	0.454886
cnv_GON4L	0.497702
cnv_GOPC	0.501381
cnv_GORAB	0.517358
cnv_GORASP2	0.415857
cnv_GOT2	0.537561
cnv_GPAA1	0.388884
cnv_GPALPP1	0.520152
cnv_GPANK1	0.494549
cnv_GPATCH1	0.345983
cnv_GPBP1	0.505944
cnv_GPBP1L1	0.57902

cnv_GPD1L	0.266548
cnv_GPHN	0.397989
cnv_GPI	0.329598
cnv_GPN3	0.346172
cnv_GPR155	0.20835
cnv_GPR89A	0.31795
cnv_GPR89B	0.734981
cnv_GPRASP1	0.248542
cnv_GPRASP2	0.253026
cnv_GPS2	0.520868
cnv_GPT2	0.25281
cnv_GRHPR	0.623467
cnv_GRK6	0.501013
cnv_GRPEL2	0.246508
cnv_GSE1	0.426572
cnv_GSKIP	0.389687
cnv_GSPT1	0.41594
cnv_GSR	0.553611
cnv_GSTA4	0.157214
cnv_GSTK1	0.521768
cnv_GSTM1	0.416343
cnv_GSTM2	0.406884
cnv_GSTM4	0.363734
cnv_GSTZ1	0.539871
cnv_GTF2A1	0.35711
cnv_GTF2A2	0.507279
cnv_GTF2B	0.484707
cnv_GTF2E2	0.589303
cnv_GTF2F1	0.520003
cnv_GTF2F2	0.518015
cnv_GTF2H1	0.5008
cnv_GTF2H2	0.408229
cnv_GTF2H2B	0.307807
cnv_GTF2H2C	0.341793
cnv_GTF2H3	0.270295
cnv_GTF2H4	0.467647
cnv_GTF2I	0.468917
cnv_GTF2IP1	0.496232
cnv_GTF3A	0.595976
cnv_GTF3C6	0.399511
cnv_GTPBP8	0.433902
cnv_GUCD1	0.505313
cnv_GUSBP3	0.332372
cnv_GVINP1	0.372642
cnv_GYG1	0.496949
cnv_GYS1	0.26664
cnv_GZF1	0.335202

cnv_H2AFV	0.505641
cnv_H3F3A	0.268922
cnv_H3F3B	0.227131
cnv_HACL1	0.46395
cnv_HADHB	0.219037
cnv_HARS	0.620063
cnv_HAT1	0.294497
cnv_HAUS1	0.490975
cnv_HAUS4	0.365776
cnv_HAUS7	0.503427
cnv_HBS1L	0.535297
cnv_HCLS1	0.402863
cnv_HDAC1	0.331748
cnv_HDDC2	0.552594
cnv_HDDC3	0.493723
cnv_HDGF	0.6771
cnv_HDHD2	0.50178
cnv_HEATR1	0.603255
cnv_HEATR3	0.239212
cnv_HEBP1	0.274753
cnv_HECA	0.518922
cnv_HECTD1	0.461257
cnv_HECTD3	0.370287
cnv_HEIH	0.394194
cnv_HERC1	0.405292
cnv_HERC5	0.388548
cnv_HERPUD1	0.493737
cnv_HERPUD2	0.397034
cnv_HESX1	0.312163
cnv_HEY2	0.409345
cnv_HIBADH	0.501392
cnv_HIGD1A	0.380201
cnv_HIGD2A	0.645567
cnv_HILPDA	0.349297
cnv_HINFP	0.723831
cnv_HINT1	0.583421
cnv_HINT2	0.47838
cnv_HIPK2	0.435336
cnv_HIPK3	0.267422
cnv_HIST1H2BN	0.425894
cnv_HLA-A	0.245182
cnv_HMBOX1	0.594301
cnv_HMBS	0.553078
cnv_HMCES	0.276199
cnv_HMG20A	0.433382
cnv_HMGB1	0.539421
cnv_HMGB3	0.299877

cnv_HMGCS1	0.310195
cnv_HMGN1	0.467687
cnv_HMGN2	0.358002
cnv_HMGN3	0.490781
cnv_HMGN4	0.45224
cnv_HMGXB4	0.242518
cnv_HNRNPA3	0.31365
cnv_HNRNPAB	0.291421
cnv_HNRNPH2	0.362103
cnv_HNRNPR	0.352896
cnv_HNRNPU	0.539284
cnv_HOMER1	0.43426
cnv_HOOK1	0.350491
cnv_HOOK2	0.384807
cnv_HP1BP3	0.538557
cnv_HPRT1	0.440843
cnv_HPS3	0.360366
cnv_HPS5	0.396366
cnv_HS2ST1	0.409654
cnv_HSBP1	0.340292
cnv_HSD17B11	0.376623
cnv_HSD17B7	0.351577
cnv_HSD17B8	0.418457
cnv_HSDL1	0.362795
cnv_HSF2	0.35563
cnv_HSH2D	0.294628
cnv_HSP90AA1	0.49834
cnv_HSP90AB1	0.463813
cnv_HSP90B1	0.323028
cnv_HSPA14	0.290136
cnv_HSPA4	0.468694
cnv_HSPA9	0.340405
cnv_HSPB11	0.414
cnv_HSPH1	0.443886
cnv_HTATIP2	0.30212
cnv_HTATSF1	0.527227
cnv_HYI	0.420995
cnv_HYKK	0.412654
cnv_HYLS1	0.459116
cnv_IARS	0.297799
cnv_IARS2	0.733738
cnv_IBTK	0.448402
cnv_ICAM2	0.394969
cnv_ICAM3	0.408716
cnv_ICAM4	0.279366
cnv_ICE1	0.498548
cnv_ICE2	0.605242

cnv_IDE	0.302639
cnv_IDH2	0.468253
cnv_IDH3A	0.565162
cnv_IDH3B	0.536985
cnv_IDH3G	0.517671
cnv_IDS	0.611713
cnv_IER3IP1	0.517039
cnv_IER5	0.283453
cnv_IFFO2	0.282748
cnv_IFI16	0.496499
cnv_IFI30	0.299898
cnv_IFITM1	0.300837
cnv_IFITM2	0.302698
cnv_IFNAR1	0.360714
cnv_IFNAR2	0.410493
cnv_IFNGR2	0.450839
cnv_IFNLR1	0.281734
cnv_IFRD2	0.355684
cnv_IFT172	0.329524
cnv_IFT22	0.28673
cnv_IFT46	0.524231
cnv_IFT88	0.309215
cnv_IGF2R	0.592175
cnv_IGIP	0.352071
cnv_IGSF8	0.382401
cnv_IK	0.511541
cnv_IKBKB	0.535376
cnv_IKZF3	0.208016
cnv_IL10RB	0.549023
cnv_IL17RA	0.321125
cnv_IL6R	0.409492
cnv_ILF2	0.65458
cnv_ILF3	0.447111
cnv_IMMP1L	0.372182
cnv_IMMP2L	0.308112
cnv_IMP3	0.425781
cnv_IMPA1	0.428037
cnv_IMPA2	0.284209
cnv_IMPACT	0.313944
cnv_IMPAD1	0.502075
cnv_IMPDH1	0.374632
cnv_IMPDH2	0.310126
cnv_ING2	0.361554
cnv_ING3	0.413544
cnv_INO80E	0.460408
cnv_INPP5A	0.316184
cnv_INSIG1	0.282025

cnv_INTS2	0.34221
cnv_INTS5	0.352955
cnv_INTS6	0.494181
cnv_INTS6-AS1	0.388653
cnv_INTS8	0.522418
cnv_IP6K2	0.475741
cnv_IPO4	0.383792
cnv_IPO5	0.545216
cnv_IPO7	0.484711
cnv_IPO8	0.345002
cnv_IQCB1	0.296661
cnv_IQCH-AS1	0.487748
cnv_IQGAP2	0.279163
cnv_IRAK1	0.470247
cnv_IREB2	0.481189
cnv_IRF1	0.302775
cnv_IRF2BP2	0.389231
cnv_IRF2BPL	0.461673
cnv_IRF5	0.384079
cnv_IRF7	0.377425
cnv_IRF9	0.523065
cnv_ISCA1	0.48017
cnv_ISCA2	0.462828
cnv_ISCU	0.215849
cnv_ISG20	0.427278
cnv_ISG20L2	0.612051
cnv_ISL2	0.442122
cnv_ISY1-RAB43	0.419354
cnv_ITCH	0.365767
cnv_ITGAE	0.414933
cnv_ITGAL	0.444306
cnv_ITM2B	0.557206
cnv_IVNS1ABP	0.471102
cnv_JADE1	0.48322
cnv_JADE2	0.305288
cnv_JAGN1	0.417543
cnv_JAK1	0.39792
cnv_JAK2	0.315356
cnv_JDP2	0.260252
cnv_JKAMP	0.428712
cnv_JMJD1C	0.381707
cnv_JMY	0.494743
cnv_JOSD1	0.370057
cnv_JRKL	0.554344
cnv_JTB	0.687944
cnv_JUN	0.32639
cnv_KANSL1-AS1	0.18907

cnv_KARS	0.587542
cnv_KAT2B	0.240823
cnv_KAT5	0.575392
cnv_KAT6B	0.274688
cnv_KAT7	0.29433
cnv_KATNA1	0.437762
cnv_KATNBL1	0.404961
cnv_KBTBD2	0.415034
cnv_KBTBD3	0.557423
cnv_KBTBD4	0.529759
cnv_KCNA3	0.27941
cnv_KCNN3	0.286924
cnv_KCTD13	0.470425
cnv_KCTD20	0.450083
cnv_KCTD21	0.249055
cnv_KCTD3	0.529163
cnv_KCTD6	0.433573
cnv_KCTD9	0.41289
cnv_KDELR1	0.316332
cnv_KDELR2	0.460879
cnv_KDM1A	0.337441
cnv_KDM2B	0.256521
cnv_KDM3B	0.302072
cnv_KDM5A	0.31413
cnv_KDM5B	0.25444
cnv_KDM6A	0.428414
cnv_KEAP1	0.35431
cnv_KHNYN	0.471501
cnv_KIAA0040	0.376507
cnv_KIAA0232	0.375929
cnv_KIAA0430	0.462166
cnv_KIAA0513	0.322456
cnv_KIAA0753	0.395091
cnv_KIAA0907	0.409985
cnv_KIAA0930	0.35911
cnv_KIAA1024	0.4207
cnv_KIAA1143	0.49781
cnv_KIAA1191	0.559306
cnv_KIAA1468	0.536891
cnv_KIAA1551	0.250493
cnv_KIF13B	0.546653
cnv_KIF21A	0.27461
cnv_KIF21B	0.445633
cnv_KIF22	0.520945
cnv_KIF3B	0.250861
cnv_KIFAP3	0.602331
cnv_KIZ	0.312466

cnv_KLC2	0.421761
cnv_KLHDC2	0.441862
cnv_KLHDC3	0.479464
cnv_KLHDC9	0.472332
cnv_KLHL12	0.616176
cnv_KLHL2	0.225022
cnv_KLHL20	0.622949
cnv_KLHL21	0.43515
cnv_KLHL24	0.231867
cnv_KLHL26	0.349331
cnv_KLHL28	0.497353
cnv_KLHL6	0.38348
cnv_KLHL7	0.413742
cnv_KMT2A	0.440016
cnv_KNSTRN	0.362959
cnv_KNTC1	0.365936
cnv_KPNA1	0.49449
cnv_KPNA3	0.492234
cnv_KPNA5	0.461111
cnv_KPNB1	0.294408
cnv_KPTN	0.353737
cnv_KRAS	0.322975
cnv_KRCC1	0.266102
cnv_KRIT1	0.331334
cnv_KRT10	0.196698
cnv_KTN1	0.60347
cnv_L3HYPDH	0.389491
cnv_LACTB	0.357772
cnv_LAGE3	0.546529
cnv_LAMC1	0.307621
cnv_LAMP1	0.470075
cnv_LAMP2	0.488973
cnv_LAMTOR1	0.561261
cnv_LAMTOR2	0.617996
cnv_LAMTOR4	0.601933
cnv_LAMTOR5	0.60348
cnv_LAPTM4A	0.227811
cnv_LARP1B	0.426978
cnv_LARP4	0.280813
cnv_LASP1	0.248912
cnv_LATS2	0.293276
cnv_LBR	0.498781
cnv_LCMT1	0.565742
cnv_LCMT2	0.257419
cnv_LCOR	0.291291
cnv_LCORL	0.347877
cnv_LDLRAP1	0.464638

cnv_LEAP2	0.317595
cnv_LEO1	0.555544
cnv_LEPROTL1	0.527239
cnv_LGALS1	0.119896
cnv_LGALS8	0.511685
cnv_LGALSL	0.323163
cnv_LGMN	0.230787
cnv_LHPP	0.380193
cnv_LHX4-AS1	0.325377
cnv_LIG4	0.430285
cnv_LIME1	0.38609
cnv_LIMS1	0.257818
cnv_LIMS3	0.251222
cnv_LIN52	0.395614
cnv_LIN7C	0.433575
cnv_LINC00094	0.27306
cnv_LINC00294	0.32804
cnv_LINC00339	0.288995
cnv_LINC00526	0.4014
cnv_LINC00528	0.295865
cnv_LINC00667	0.479437
cnv_LINC00888	0.261108
cnv_LINC00893	0.312397
cnv_LINC00909	0.380592
cnv_LINC00969	0.331341
cnv_LIX1L	0.324126
cnv_LMAN1	0.457838
cnv_LMAN2L	0.281401
cnv_LMBRD1	0.496073
cnv_LMBRD2	0.273064
cnv_LMNA	0.24525
cnv_LMNB2	0.300691
cnv_LMO4	0.512862
cnv_LNP1	0.487089
cnv_LNPEP	0.389139
cnv_LNX2	0.48358
cnv_LOC100049716	0.348249
cnv_LOC100129518	0.49511
cnv_LOC100147773	0.448503
cnv_LOC100270804	0.27854
cnv_LOC100287497	0.313217
cnv_LOC100506548	0.504459
cnv_LOC100506844	0.349494
cnv_LOC100506990	0.227706
cnv_LOC100507431	0.352204
cnv_LOC100507670	0.390766
cnv_LOC101926963	0.307882

cnv_LOC101927178	0.243736
cnv_LOC101927204	0.517515
cnv_LOC101927420	0.263701
cnv_LOC101927974	0.277724
cnv_LOC101928000	0.336432
cnv_LOC101928111	0.257855
cnv_LOC101928143	0.609712
cnv_LOC101929243	0.20169
cnv_LOC102606465	0.403416
cnv_LOC102724532	0.280336
cnv_LOC150776	0.365869
cnv_LOC374443	0.250264
cnv_LOC440434	0.273815
cnv_LOC643733	0.29163
cnv_LOC728613	0.310264
cnv_LOC730098	0.280718
cnv_LONP2	0.622894
cnv_LONRF1	0.386894
cnv_LPIN2	0.320603
cnv_LPXN	0.291813
cnv_LRBA	0.260418
cnv_LRCH1	0.494033
cnv_LRMP	0.288985
cnv_LRP10	0.280074
cnv_LRRC37B	0.286258
cnv_LRRC40	0.282717
cnv_LRRC42	0.461126
cnv_LRRC47	0.525363
cnv_LRRC58	0.421029
cnv_LRRC8C	0.389612
cnv_LRRD1	0.376679
cnv_LRRFIP1	0.226859
cnv_LRWD1	0.507124
cnv_LSG1	0.46148
cnv_LSM1	0.719977
cnv_LSM10	0.483969
cnv_LSM14A	0.316016
cnv_LSM2	0.491041
cnv_LSM4	0.457137
cnv_LSM5	0.518862
cnv_LSM7	0.591808
cnv_LSM8	0.37545
cnv_LTN1	0.363857
cnv_LTV1	0.508741
cnv_LUC7L2	0.44888
cnv_LUC7L3	0.366406
cnv_LUZP6	0.329053

cnv_LYN	0.339194
cnv_LYPLA1	0.368032
cnv_LYPLAL1	0.39487
cnv_LYRM1	0.371535
cnv_LYRM4	0.487844
cnv_LYRM7	0.345424
cnv_LYSMD2	0.270439
cnv_LYSMD4	0.352449
cnv_LZIC	0.471893
cnv_LZTFL1	0.288547
cnv_M6PR	0.519709
cnv_MAD2L1BP	0.296129
cnv_MAD2L2	0.257207
cnv_MAEA	0.476684
cnv_MAF1	0.446681
cnv_MAGED4	0.134954
cnv_MAGED4B	0.15239
cnv_MAGOH	0.413863
cnv_MAK16	0.407441
cnv_MALT1	0.509489
cnv_MAML1	0.367417
cnv_MAML2	0.369971
cnv_MAN1A1	0.287455
cnv_MAN1A2	0.43277
cnv_MAN1C1	0.206309
cnv_MAN2A1	0.399321
cnv_MAN2A2	0.27684
cnv_MAN2B2	0.388654
cnv_MANBA	0.441101
cnv_MANEA	0.299582
cnv_MAP1LC3B	0.555766
cnv_MAP2K3	0.319758
cnv_MAP2K4	0.465678
cnv_MAP3K13	0.424079
cnv_MAP3K14	0.281274
cnv_MAP3K4	0.593587
cnv_MAP3K5	0.322329
cnv_MAP3K7	0.514991
cnv_MAP4K5	0.443075
cnv_MAP7D1	0.362867
cnv_MAPK1	0.40418
cnv_MAPK13	0.275383
cnv_MAPK1IP1L	0.633735
cnv_MAPK8	0.317297
cnv_MAPKAPK5	0.429187
cnv_MAPRE1	0.403775
cnv_MARCH5	0.305029

cnv_MARCKS	0.194273
cnv_MARS	0.443865
cnv_MAT2B	0.333054
cnv_MATN1-AS1	0.354333
cnv_MAX	0.531758
cnv_MBD4	0.391951
cnv_MBIP	0.269745
cnv_MBNL2	0.476215
cnv_MBNL3	0.4565
cnv_MBTPS1	0.511945
cnv_MCC	0.440528
cnv_MCCC1	0.405653
cnv_MCL1	0.374435
cnv_MCM3	0.243815
cnv_MCM4	0.285522
cnv_MCM5	0.166512
cnv_MCM7	0.420983
cnv_MCOLN1	0.338904
cnv_MCPH1	0.583487
cnv_MCRS1	0.404255
cnv_MCTP2	0.403637
cnv_MCUR1	0.526241
cnv_MDC1	0.345891
cnv_MDH2	0.574965
cnv_MDM4	0.416189
cnv_MDN1	0.43695
cnv_MDP1	0.493447
cnv_MEA1	0.487281
cnv_MECP2	0.50711
cnv_MED1	0.33099
cnv_MED10	0.484217
cnv_MED11	0.428137
cnv_MED13	0.289069
cnv_MED17	0.574097
cnv_MED19	0.62894
cnv_MED20	0.312989
cnv_MED23	0.568743
cnv_MED26	0.333896
cnv_MED29	0.263629
cnv_MED30	0.478932
cnv_MED31	0.437742
cnv_MED4	0.442279
cnv_MED6	0.601193
cnv_MED7	0.366412
cnv_MED8	0.368271
cnv_MEF2A	0.457531
cnv_MEIS2	0.437338

cnv_MEN1	0.41515
cnv_MEPCE	0.437132
cnv_METTL1	0.308742
cnv_METTL13	0.555501
cnv_METTL18	0.458495
cnv_METTL23	0.564338
cnv_METTL25	0.427605
cnv_METTL2B	0.32732
cnv_METTL3	0.440301
cnv_METTL4	0.495352
cnv_MEX3C	0.455135
cnv_MFAP1	0.37589
cnv_MFF	0.311878
cnv_MFSD1	0.244987
cnv_MFSD8	0.297927
cnv_MGAT1	0.420871
cnv_MGAT2	0.288773
cnv_MGEA5	0.322908
cnv_MGRN1	0.316187
cnv_MIA3	0.465159
cnv_MIB1	0.42147
cnv_MICAL1	0.303415
cnv_MICB	0.31916
cnv_MICU1	0.361173
cnv_MICU2	0.502314
cnv_MID2	0.365619
cnv_MIER1	0.43163
cnv_MIER3	0.46179
cnv_MIF4GD	0.481218
cnv_MIOS	0.494095
cnv_MIR1236	0.572949
cnv_MIR1248	0.289148
cnv_MIR1304	0.624193
cnv_MIR186	0.391047
cnv_MIR21	0.265494
cnv_MIR3620	0.701172
cnv_MIR3652	0.356592
cnv_MIR3656	0.413773
cnv_MIR3658	0.463462
cnv_MIR4467	0.507124
cnv_MIR4647	0.454904
cnv_MIR4657	0.419182
cnv_MIR4707	0.365776
cnv_MIR5193	0.302292
cnv_MIR6132	0.553929
cnv_MIR6513	0.259442
cnv_MIR664B	0.443859

cnv_MIR6741	0.554376
cnv_MIR6748	0.337674
cnv_MIR6751	0.295562
cnv_MIR6758	0.429584
cnv_MIR6778	0.240418
cnv_MIR6800	0.255378
cnv_MIR6805	0.416576
cnv_MIR6824	0.349402
cnv_MIR6831	0.429259
cnv_MIR6837	0.350428
cnv_MIR6840	0.476836
cnv_MIR6845	0.293476
cnv_MIR6866	0.362371
cnv_MIR6878	0.756168
cnv_MIR6883	0.292209
cnv_MIR6884	0.394183
cnv_MIR6890	0.384153
cnv_MIS12	0.402901
cnv_MIS18A	0.349826
cnv_MIS18BP1	0.414227
cnv_MKKS	0.457008
cnv_MKLN1	0.355714
cnv_MKNK2	0.285749
cnv_MKRN1	0.339512
cnv_MLEC	0.39299
cnv_MLF2	0.453483
cnv_MLH1	0.326708
cnv_MLKL	0.390299
cnv_MLLT11	0.381603
cnv_MMGT1	0.57339
cnv_MNAT1	0.439694
cnv_MOAP1	0.580605
cnv_MOB1A	0.293481
cnv_MOCS2	0.370987
cnv_MON1B	0.532632
cnv_MON2	0.34458
cnv_MORC1	0.323059
cnv_MORC3	0.39432
cnv_MORC4	0.546786
cnv_MORF4L1	0.560824
cnv_MORF4L2	0.342926
cnv_MOSPD1	0.472787
cnv_MPC1	0.596264
cnv_MPC2	0.664353
cnv_MPHOSPH6	0.42418
cnv_MPHOSPH8	0.57505
cnv_MPLKIP	0.337023

cnv_MPP1	0.231686
cnv_MPP5	0.410812
cnv_MPV17	0.439278
cnv_MPV17L2	0.276351
cnv_MPZL1	0.444215
cnv_MR1	0.488007
cnv_MRFAP1	0.365606
cnv_MRI1	0.263325
cnv_MROH7-TTC4	0.317319
cnv_MRPL11	0.451219
cnv_MRPL13	0.421965
cnv_MRPL14	0.392382
cnv_MRPL16	0.417104
cnv_MRPL17	0.375334
cnv_MRPL18	0.596164
cnv_MRPL2	0.391094
cnv_MRPL21	0.4933
cnv_MRPL22	0.538694
cnv_MRPL24	0.666644
cnv_MRPL3	0.309073
cnv_MRPL32	0.386474
cnv_MRPL34	0.575653
cnv_MRPL36	0.517916
cnv_MRPL39	0.428931
cnv_MRPL4	0.503075
cnv_MRPL40	0.453399
cnv_MRPL43	0.401849
cnv_MRPL46	0.474529
cnv_MRPL48	0.409505
cnv_MRPL49	0.451203
cnv_MRPL50	0.475559
cnv_MRPL51	0.530248
cnv_MRPL53	0.303765
cnv_MRPL54	0.609045
cnv_MRPL57	0.477669
cnv_MRPL9	0.782719
cnv_MRPS10	0.419645
cnv_MRPS14	0.631631
cnv_MRPS17	0.396796
cnv_MRPS18A	0.459197
cnv_MRPS18B	0.523078
cnv_MRPS21	0.368311
cnv_MRPS22	0.407819
cnv_MRPS24	0.5337
cnv_MRPS25	0.456675
cnv_MRPS28	0.386319
cnv_MRPS30	0.390149

cnv_MRPS31	0.619222
cnv_MRPS31P5	0.474549
cnv_MRPS33	0.506556
cnv_MRPS34	0.343995
cnv_MRPS36	0.614476
cnv_MRPS6	0.342371
cnv_MRPS7	0.430944
cnv_MRRF	0.60123
cnv_MRS2	0.475036
cnv_MSANTD2	0.463646
cnv_MSANTD3	0.41337
cnv_MSANTD4	0.538411
cnv_MSH5	0.420744
cnv_MSH5-SAPCD1	0.421133
cnv_MSL2	0.269359
cnv_MSRB1	0.251652
cnv_MSTO1	0.666017
cnv_MSTO2P	0.620909
cnv_MT1E	0.191611
cnv_MT1F	0.219856
cnv_MT1X	0.284404
cnv_MTA2	0.256242
cnv_MTAP	0.330149
cnv_MTCH1	0.460844
cnv_MTCH2	0.39226
cnv_MTDH	0.383675
cnv_MTERF3	0.390018
cnv_MTF2	0.413508
cnv_MTFMT	0.557956
cnv_MTFP1	0.305571
cnv_MTFR1	0.424706
cnv_MTG1	0.201148
cnv_MTHFD1	0.485063
cnv_MTHFD1L	0.35568
cnv_MTMR1	0.590509
cnv_MTMR12	0.42772
cnv_MTMR2	0.56905
cnv_MTMR4	0.366777
cnv_MTMR9	0.520747
cnv_MTO1	0.517772
cnv_MTR	0.589551
cnv_MTRR	0.351978
cnv_MTUS1	0.351321
cnv_MTX1	0.657746
cnv_MTX3	0.326418
cnv_MUS81	0.506379
cnv_MUT	0.430071

cnv_MXI1	0.267832
cnv_MYBL2	0.380455
cnv_MYCBP2	0.714055
cnv_MYD88	0.407129
cnv_MYEOV	0.271423
cnv_MYH9	0.380926
cnv_MYL12A	0.398044
cnv_MYL12B	0.614043
cnv_MYL6B	0.365026
cnv_MYO18A	0.261109
cnv_MYO5A	0.243217
cnv_MZF1	0.450338
cnv_MZT1	0.540553
cnv_MZT2A	0.33577
cnv_N4BP1	0.492784
cnv_N4BP2	0.216575
cnv_N4BP2L2	0.620038
cnv_NAA10	0.492986
cnv_NAA16	0.504011
cnv_NAA20	0.45156
cnv_NAA35	0.460005
cnv_NAA38	0.403091
cnv_NAA40	0.566439
cnv_NAA50	0.414661
cnv_NAE1	0.409893
cnv_NAGA	0.264405
cnv_NAGK	0.282185
cnv_NAGPA	0.332354
cnv_NAPB	0.369241
cnv_NAPG	0.489151
cnv_NARS	0.504019
cnv_NARS2	0.424331
cnv_NASP	0.267878
cnv_NAT1	0.312189
cnv_NAT10	0.410199
cnv_NBEA	0.254151
cnv_NBN	0.455445
cnv_NBPF11	0.358722
cnv_NBPF12	0.331494
cnv_NBPF15	0.351275
cnv_NBPF9	0.38696
cnv_NCAPD2	0.300341
cnv_NCAPD3	0.459411
cnv_NCBP1	0.317824
cnv_NCBP2	0.357464
cnv_NCF1	0.280544
cnv_NCF1B	0.254643

cnv_NCF1C	0.272106
cnv_NCK1	0.397908
cnv_NCK1-AS1	0.352993
cnv_NCKAP1L	0.382991
cnv_NCOA1	0.257553
cnv_NCOA2	0.383422
cnv_NCOA7	0.438236
cnv_NDFIP1	0.426527
cnv_NDFIP2	0.647698
cnv_NDNF	0.330233
cnv_NDUFA1	0.523881
cnv_NDUFA12	0.484498
cnv_NDUFA13	0.315782
cnv_NDUFA3	0.42011
cnv_NDUFA4	0.689606
cnv_NDUFA5	0.379984
cnv_NDUFA8	0.568373
cnv_NDUFA9	0.461893
cnv_NDUFAF1	0.397839
cnv_NDUFAF2	0.501196
cnv_NDUFAF3	0.389798
cnv_NDUFAF6	0.417507
cnv_NDUFB10	0.503428
cnv_NDUFB5	0.493438
cnv_NDUFB6	0.467805
cnv_NDUFB9	0.58096
cnv_NDUFC1	0.428903
cnv_NDUFS2	0.648187
cnv_NDUFS3	0.512933
cnv_NDUFS4	0.623902
cnv_NDUFS6	0.654426
cnv_NDUFV2	0.4761
cnv_NDUFV3	0.527613
cnv_NECAP1	0.418434
cnv_NEDD1	0.306787
cnv_NEDD8	0.536224
cnv_NEDD8-MDP1	0.493447
cnv_NEK3	0.465687
cnv_NEK4	0.313221
cnv_NEK7	0.514744
cnv_NELFCD	0.507494
cnv_NELFE	0.572775
cnv_NEMF	0.547218
cnv_NEO1	0.310597
cnv_NES	0.490276
cnv_NEU1	0.412038
cnv_NFE2L1	0.269377

cnv_NFKB1	0.319643
cnv_NFS1	0.342004
cnv_NFU1	0.33161
cnv_NFX1	0.409554
cnv_NGLY1	0.317221
cnv_NGRN	0.474463
cnv_NHLRC3	0.381686
cnv_NHP2	0.390138
cnv_NIP7	0.311245
cnv_NIPA1	0.373204
cnv_NIPA2	0.539305
cnv_NIPAL2	0.297777
cnv_NIPBL	0.359292
cnv_NIT2	0.410388
cnv_NKAP	0.533942
cnv_NLRC5	0.303243
cnv_NMB	0.370197
cnv_NMD3	0.456952
cnv_NME3	0.351393
cnv_NME6	0.359407
cnv_NME7	0.289279
cnv_NNT	0.403008
cnv_NNT-AS1	0.339889
cnv_NOL11	0.256725
cnv_NOL7	0.548983
cnv_NOL8	0.537626
cnv_NOM1	0.370832
cnv_NOP10	0.480762
cnv_NOP16	0.370176
cnv_NOSIP	0.374551
cnv_NPAT	0.535039
cnv_NPC2	0.349591
cnv_NPEPPS	0.432001
cnv_NPM1	0.460418
cnv_NPTN	0.390019
cnv_NPTN-IT1	0.3124
cnv_NR1H2	0.324462
cnv_NR2C2	0.288743
cnv_NR3C1	0.258331
cnv_NRBP1	0.382863
cnv_NRBP2	0.292941
cnv_NRG2	0.615146
cnv_NSA2	0.588145
cnv_NSDHL	0.600561
cnv_NSMAF	0.407333
cnv_NSMCE1	0.344211
cnv_NSUN2	0.562798

cnv_NSUN5	0.519376
cnv_NSUN5P1	0.468129
cnv_NSUN5P2	0.478084
cnv_NT5C2	0.329354
cnv_NT5C3B	0.310797
cnv_NT5DC1	0.554432
cnv_NTAN1	0.341129
cnv_NTPCR	0.652549
cnv_NUBP1	0.43397
cnv_NUBPL	0.343139
cnv_NUCKS1	0.481812
cnv_NUDCD1	0.454222
cnv_NUDT15	0.537705
cnv_NUDT18	0.301043
cnv_NUDT2	0.46504
cnv_NUDT21	0.375929
cnv_NUDT3	0.473584
cnv_NUDT9	0.477494
cnv_NUMB	0.576216
cnv_NUP133	0.560064
cnv_NUP153	0.405188
cnv_NUP155	0.383177
cnv_NUP160	0.5931
cnv_NUP205	0.387812
cnv_NUP210	0.401217
cnv_NUP43	0.442107
cnv_NUP85	0.392762
cnv_NUP88	0.422271
cnv_NUP98	0.557814
cnv_NUPL2	0.422965
cnv_NUS1	0.520727
cnv_NVL	0.65483
cnv_NXT1	0.414736
cnv_NXT2	0.404382
cnv_OARD1	0.472805
cnv_OCIAD2	0.389539
cnv_OCRL	0.455994
cnv_ODF2	0.392222
cnv_OFD1	0.228593
cnv_OGFOD1	0.551189
cnv_OGFRL1	0.210658
cnv_OIP5-AS1	0.383357
cnv_OMA1	0.457738
cnv_OPA1	0.320362
cnv_OPN3	0.661695
cnv_ORAI3	0.328794
cnv_ORC3	0.500939

cnv_ORC5	0.444406
cnv_ORMDL1	0.253281
cnv_ORMDL2	0.389269
cnv_OS9	0.370807
cnv_OSBP	0.54595
cnv_OSBPL8	0.409398
cnv_OSBPL9	0.526483
cnv_OSER1	0.281285
cnv_OSGEP	0.427179
cnv_OST4	0.393014
cnv_OSTC	0.290433
cnv_OSTF1	0.314234
cnv_OSTM1	0.467905
cnv_OTUD1	0.304107
cnv_OTUD6B	0.38549
cnv_OTUD6B-AS1	0.526705
cnv_OXA1L	0.518946
cnv_OXNAD1	0.442588
cnv_OXSM	0.424092
cnv_OXSR1	0.408191
cnv_P2RX4	0.166753
cnv_P2RX5-TAX1BP3	0.412712
cnv_P4HA1	0.406235
cnv_P4HTM	0.327767
cnv_PAAF1	0.483137
cnv_PABPC1L	0.392647
cnv_PABPN1	0.404326
cnv_PAFAH1B1	0.371484
cnv_PAFAH1B3	0.339961
cnv_PAIP1	0.441185
cnv_PAK1	0.479231
cnv_PAK1IP1	0.471515
cnv_PAK2	0.286603
cnv_PALB2	0.463187
cnv_PALM2-AKAP2	0.47238
cnv_PAM	0.422119
cnv_PAN2	0.307758
cnv_PAN3	0.613985
cnv_PANX1	0.525209
cnv_PAPD5	0.364649
cnv_PAPD7	0.429564
cnv_PAPOLA	0.585759
cnv_PAPSS1	0.340833
cnv_PARK7	0.392163
cnv_PARM1	0.326083
cnv_PARN	0.498431
cnv_PARP1	0.675536

cnv_PARP16	0.554506
cnv_PARP2	0.485038
cnv_PARP3	0.372123
cnv_PARP4	0.430353
cnv_PARP9	0.315583
cnv_PAXBP1	0.49729
cnv_PAXIP1	0.26375
cnv_PAXIP1-AS1	0.195176
cnv_PBXIP1	0.341079
cnv_PCAT6	0.353777
cnv_PCBP1	0.31529
cnv_PCBP2	0.316279
cnv_PCF11	0.492183
cnv_PCGF6	0.283301
cnv_PCID2	0.571296
cnv_PCM1	0.591219
cnv_PCMT1	0.657446
cnv_PCNP	0.249609
cnv_PCYOX1	0.246268
cnv_PDCD10	0.337295
cnv_PDCD2L	0.372087
cnv_PDCD5	0.418746
cnv_PDCD6IP	0.354084
cnv_PDCD7	0.393709
cnv_PDCL	0.391288
cnv_PDE8A	0.42747
cnv_PDF	0.40165
cnv_PDHB	0.457976
cnv_PDHX	0.482093
cnv_PDIA6	0.267638
cnv_PDP1	0.355279
cnv_PDS5A	0.307115
cnv_PDSS2	0.513381
cnv_PDZD8	0.240071
cnv_PEA15	0.309768
cnv_PEBP1	0.256968
cnv_PEF1	0.420906
cnv_PEMT	0.319613
cnv_PER1	0.292209
cnv_PET100	0.482106
cnv_PEX1	0.453628
cnv_PEX11B	0.314856
cnv_PEX19	0.648764
cnv_PEX2	0.479446
cnv_PEX3	0.539551
cnv_PEX7	0.6489
cnv_PFDN2	0.653463

cnv_PFN1	0.257096
cnv_PGAP2	0.330812
cnv_PGGT1B	0.348331
cnv_PGM1	0.423118
cnv_PGM3	0.429742
cnv_PGM5-AS1	0.412782
cnv_PGRMC1	0.3995
cnv_PHACTR4	0.413675
cnv_PHAX	0.359681
cnv_PHB	0.468352
cnv_PHC3	0.276061
cnv_PHF10	0.534772
cnv_PHF11	0.561242
cnv_PHF13	0.342489
cnv_PHF14	0.410532
cnv_PHF6	0.346489
cnv_PHKB	0.4704
cnv_PHLDA1	0.234606
cnv_PHLPP2	0.237805
cnv_PHTF1	0.263722
cnv_PHYH	0.282494
cnv_PI4KA	0.17285
cnv_PI4KAP1	0.468486
cnv_PI4KAP2	0.432099
cnv_PIAS1	0.314749
cnv_PIAS3	0.388428
cnv_PIBF1	0.324622
cnv_PICALM	0.524546
cnv_PIGA	0.222658
cnv_PIGB	0.433012
cnv_PIGC	0.61073
cnv_PIGH	0.56162
cnv_PIGK	0.51008
cnv_PIGM	0.541473
cnv_PIGN	0.465801
cnv_PIGP	0.378548
cnv_PIGU	0.419325
cnv_PIGY	0.392593
cnv_PIK3C2A	0.439346
cnv_PIK3CB	0.340013
cnv_PIK3R2	0.324167
cnv_PIKFYVE	0.276631
cnv_PILRB	0.476836
cnv_PIN1	0.511812
cnv_PINK1	0.437863
cnv_PINLYP	0.27431
cnv_PIP4K2A	0.200282

cnv_PIP5K1A	0.653113
cnv_PIP5K1B	0.333284
cnv_PITPNA	0.566856
cnv_PITPNA-AS1	0.261643
cnv_PITPNB	0.520895
cnv_PJA1	0.179371
cnv_PJA2	0.35575
cnv_PKD2	0.340876
cnv_PKN2	0.517446
cnv_PLA2G15	0.296226
cnv_PLA2G4A	0.410914
cnv_PLAA	0.419961
cnv_PLCG2	0.433831
cnv_PLCL2	0.309861
cnv_PLEKHA2	0.492206
cnv_PLGRKT	0.537603
cnv_PLIN3	0.38736
cnv_PLOD3	0.43993
cnv_PLP2	0.222999
cnv_PLXNC1	0.247844
cnv_PMF1	0.482681
cnv_PMM1	0.343883
cnv_PMM2	0.526186
cnv_PMS2P1	0.605188
cnv_PMS2P5	0.412444
cnv_PMVK	0.594099
cnv_PNISR	0.537339
cnv_PNKP	0.424849
cnv_PNMA1	0.303656
cnv_PNN	0.49157
cnv_PNOC	0.526685
cnv_PNPO	0.383566
cnv_PNRC1	0.490963
cnv_POC5	0.423596
cnv_POGK	0.606553
cnv_POGLUT1	0.446959
cnv_POGZ	0.372451
cnv_POLB	0.408744
cnv_POLD2	0.54001
cnv_POLDIP2	0.378471
cnv_POLE3	0.320431
cnv_POLK	0.393987
cnv_POLM	0.415207
cnv_POLR1C	0.355107
cnv_POLR1D	0.640403
cnv_POLR2G	0.507722
cnv_POLR2H	0.511579

cnv_POLR2I	0.300398
cnv_POLR2J	0.485981
cnv_POLR2J2	0.385595
cnv_POLR2J3	0.377797
cnv_POLR2K	0.4765
cnv_POLR2M	0.552619
cnv_POLR3C	0.486996
cnv_POLR3D	0.316964
cnv_POLR3GL	0.359483
cnv_POM121C	0.5096
cnv_POMP	0.42938
cnv_POMZP3	0.293538
cnv_POP5	0.436911
cnv_POP7	0.400331
cnv_POR	0.299284
cnv_POT1	0.34549
cnv_POU2F2	0.283702
cnv_PP7080	0.305009
cnv_PPBP	0.247986
cnv_PPCDC	0.58658
cnv_PPFIA1	0.569648
cnv_PPFIBP2	0.280026
cnv_PPIA	0.543454
cnv_PPIG	0.364268
cnv_PPIL1	0.27971
cnv_PPIL4	0.476031
cnv_PPIP5K1	0.352254
cnv_PPIP5K2	0.334005
cnv_PPM1A	0.566517
cnv_PPM1K	0.493529
cnv_PPM1M	0.251927
cnv_PPOX	0.47068
cnv_PPP1CA	0.32415
cnv_PPP1CB	0.352204
cnv_PPP1R12A	0.300417
cnv_PPP1R14D	0.311068
cnv_PPP1R15B	0.458666
cnv_PPP1R18	0.266862
cnv_PPP1R21	0.24943
cnv_PPP1R35	0.550003
cnv_PPP1R3E	0.372511
cnv_PPP1R8	0.284219
cnv_PPP2CA	0.408888
cnv_PPP2R1B	0.605833
cnv_PPP2R5C	0.572661
cnv_PPP2R5E	0.541889
cnv_PPP3CA	0.279141

cnv_PPP3R1	0.260904
cnv_PPP4C	0.636869
cnv_PPP4R1	0.307358
cnv_PPP4R2	0.468283
cnv_PPP6C	0.482692
cnv_PPP6R3	0.522752
cnv_PPTC7	0.325087
cnv_PPWD1	0.325193
cnv_PRADC1	0.22469
cnv_PRCC	0.576589
cnv_PRDM2	0.401678
cnv_PRDM5	0.293625
cnv_PRDX1	0.328246
cnv_PRDX6	0.558298
cnv_PREB	0.32468
cnv_PRELID1	0.625662
cnv_PRICKLE2	0.317601
cnv_PRICKLE4	0.551954
cnv_PRIM1	0.274096
cnv_PRKAB2	0.687241
cnv_PRKACB	0.342839
cnv_PRKAR1A	0.22489
cnv_PRKCSH	0.273551
cnv_PRKCZ	0.352874
cnv_PRKD2	0.312266
cnv_PRKDC	0.438325
cnv_PRKRA	0.271217
cnv_PRMT3	0.363012
cnv_PRMT7	0.369473
cnv_PRMT9	0.238721
cnv_PRORSD1P	0.264752
cnv_PROSC	0.637781
cnv_PROSER1	0.600044
cnv_PRPF19	0.447301
cnv_PRPF3	0.390038
cnv_PRPF38B	0.521453
cnv_PRPF39	0.309882
cnv_PRPF4	0.40776
cnv_PRPF4B	0.462558
cnv_PRPF8	0.582916
cnv_PRPS1	0.496858
cnv_PRPSAP2	0.433325
cnv_PRR13	0.248695
cnv_PRR14	0.492113
cnv_PRR7	0.449839
cnv_PRRC2C	0.492073
cnv_PRSS16	0.301968

cnv_PSAP	0.388585
cnv_PSMA1	0.487315
cnv_PSMA2	0.545273
cnv_PSMA4	0.424959
cnv_PSMA6	0.369477
cnv_PSMA7	0.460052
cnv_PSMB10	0.415057
cnv_PSMB3	0.255258
cnv_PSMB4	0.758832
cnv_PSMB6	0.522202
cnv_PSMB7	0.353162
cnv_PSMB8	0.270562
cnv_PSMC1	0.347201
cnv_PSMC3	0.520878
cnv_PSMC3IP	0.315282
cnv_PSMC5	0.346248
cnv_PSMC6	0.415825
cnv_PSMD10	0.438152
cnv_PSMD11	0.298947
cnv_PSMD12	0.221697
cnv_PSMD13	0.486525
cnv_PSMD2	0.407629
cnv_PSMD3	0.352463
cnv_PSMD4	0.799316
cnv_PSMD6-AS2	0.275947
cnv_PSMD7	0.511025
cnv_PSMD8	0.277988
cnv_PSME1	0.557235
cnv_PSME3	0.280825
cnv_PSME4	0.279483
cnv_PSMG1	0.486484
cnv_PSMG2	0.482448
cnv_PSMG3	0.556109
cnv_PSPC1	0.528511
cnv_PTCD2	0.340501
cnv_PTCH1	0.435041
cnv_PTDSS1	0.406978
cnv_PTEN	0.303879
cnv_PTGES3	0.404145
cnv_PTGR2	0.560907
cnv_PTK2B	0.438342
cnv_PTP4A1	0.462372
cnv_PTP4A2	0.461472
cnv_PTPMT1	0.5542
cnv_PTPN6	0.325616
cnv_PTRH2	0.296798
cnv_PTS	0.490103

cnv_PTTG1IP	0.371813
cnv_PUF60	0.510938
cnv_PURB	0.441272
cnv_PUS3	0.545263
cnv_PUS7	0.302078
cnv_PUS7L	0.3629
cnv_PWP1	0.352188
cnv_PXK	0.331191
cnv_PXN-AS1	0.33275
cnv_PYCR2	0.554376
cnv_PYROXD1	0.451484
cnv_PYURF	0.392593
cnv_QARS	0.384153
cnv_QKI	0.606763
cnv_QRSL1	0.442058
cnv_QSER1	0.482205
cnv_QTRT1	0.487985
cnv_R3HCC1	0.626107
cnv_RAB10	0.269414
cnv_RAB11A	0.448157
cnv_RAB11FIP1	0.411821
cnv_RAB11FIP5	0.356562
cnv_RAB12	0.400829
cnv_RAB1B	0.42555
cnv_RAB22A	0.279193
cnv_RAB24	0.463403
cnv_RAB27A	0.32139
cnv_RAB28	0.371627
cnv_RAB29	0.452527
cnv_RAB30	0.366214
cnv_RAB30-AS1	0.348132
cnv_RAB33B	0.311294
cnv_RAB39B	0.259999
cnv_RAB3B	0.283102
cnv_RAB3GAP2	0.690601
cnv_RAB4A	0.484465
cnv_RAB5B	0.377696
cnv_RAB8A	0.278386
cnv_RAB8B	0.32421
cnv_RABEP1	0.433958
cnv_RABEPK	0.475607
cnv_RABGAP1L	0.424864
cnv_RABIF	0.621411
cnv_RABL3	0.396866
cnv_RAC1	0.60982
cnv_RAD1	0.440648
cnv_RAD17	0.337348

cnv_RAD21	0.528646
cnv_RAD23A	0.455881
cnv_RAD23B	0.345745
cnv_RAD50	0.366636
cnv_RAD51-AS1	0.310053
cnv_RAD51C	0.338923
cnv_RAD9A	0.360815
cnv_RAF1	0.486688
cnv_RALA	0.447901
cnv_RALBP1	0.503228
cnv_RALGPS2	0.54916
cnv_RALY	0.291042
cnv_RALY-AS1	0.340408
cnv_RANBP6	0.331948
cnv_RANBP9	0.489752
cnv_RAP1B	0.356035
cnv_RAP1GAP2	0.198993
cnv_RAP2A	0.292204
cnv_RAP2B	0.181101
cnv_RAP2C	0.503876
cnv_RAPGEF1	0.304188
cnv_RAPGEF6	0.337233
cnv_RARS	0.526206
cnv_RASA2	0.39161
cnv_RASA4	0.383021
cnv_RASA4B	0.377699
cnv_RASSF5	0.456458
cnv_RB1	0.517429
cnv_RB1CC1	0.357595
cnv_RBBP4	0.360643
cnv_RBBP5	0.391444
cnv_RBBP7	0.160558
cnv_RBBP8	0.447137
cnv_RBCK1	0.433273
cnv_RBL2	0.513901
cnv_RBM12B	0.276216
cnv_RBM18	0.529929
cnv_RBM22	0.573745
cnv_RBM23	0.37634
cnv_RBM25	0.556733
cnv_RBM33	0.371912
cnv_RBM34	0.62218
cnv_RBM39	0.380106
cnv_RBM41	0.432977
cnv_RBM47	0.34381
cnv_RBM4B	0.330089
cnv_RBM5	0.366909

cnv_RBM6	0.288123
cnv_RBM7	0.6203
cnv_RBM8A	0.311498
cnv_RBMS1	0.182488
cnv_RBMX2	0.548642
cnv_RBMXL1	0.464896
cnv_RBPJ	0.439087
cnv_RBX1	0.342734
cnv_RC3H1	0.304305
cnv_RC3H2	0.478063
cnv_RCBTB2	0.418408
cnv_RCN1	0.285877
cnv_RCN2	0.471413
cnv_RCOR1	0.471779
cnv_RCOR3	0.621829
cnv_RDX	0.601777
cnv_REEP3	0.270474
cnv_REEP5	0.517788
cnv_RELA	0.462735
cnv_RELL2	0.324535
cnv_REPS1	0.659812
cnv_REV3L	0.406797
cnv_REXO2	0.500392
cnv_RFC1	0.350312
cnv_RFC2	0.327129
cnv_RFC5	0.29304
cnv_RFESD	0.352542
cnv_RFK	0.284354
cnv_RFWD2	0.670013
cnv_RFX5	0.464747
cnv_RFX7	0.428867
cnv_RFXANK	0.581327
cnv_RGCC	0.352599
cnv_RGPD3	0.296688
cnv_RGPD4	0.304167
cnv_RHBDD2	0.456049
cnv_RHNO1	0.329553
cnv_RHOB	0.203802
cnv_RHOBTB2	0.38197
cnv_RHOG	0.402345
cnv_RHOH	0.323727
cnv_RIC3	0.313944
cnv_RICTOR	0.309972
cnv_RINT1	0.482923
cnv_RIOK1	0.514869
cnv_RIOK2	0.447351
cnv_RIPK2	0.381544

cnv_RIT1	0.396725
cnv_RMDN1	0.434622
cnv_RMDN3	0.525337
cnv_RMI2	0.268667
cnv_RMND1	0.58346
cnv_RNASE4	0.15416
cnv_RNASEH1	0.210495
cnv_RNASEK	0.490792
cnv_RNASEK-C17orf49	0.407024
cnv_RNF10	0.405375
cnv_RNF11	0.455165
cnv_RNF111	0.372548
cnv_RNF113A	0.446672
cnv_RNF114	0.505215
cnv_RNF115	0.480303
cnv_RNF123	0.374337
cnv_RNF126	0.322468
cnv_RNF13	0.407153
cnv_RNF138	0.55554
cnv_RNF139	0.565907
cnv_RNF14	0.458745
cnv_RNF145	0.350436
cnv_RNF146	0.473684
cnv_RNF168	0.389514
cnv_RNF169	0.546472
cnv_RNF181	0.257947
cnv_RNF185	0.40811
cnv_RNF19A	0.388623
cnv_RNF20	0.273256
cnv_RNF38	0.447363
cnv_RNF4	0.369307
cnv_RNF44	0.42857
cnv_RNFT1	0.246126
cnv_RNMT	0.524735
cnv_RNPC3	0.446521
cnv_RNPEP	0.640638
cnv_ROCK2	0.271496
cnv_ROMO1	0.38587
cnv_RPA1	0.308942
cnv_RPA3	0.468678
cnv_RPAP1	0.363269
cnv_RPAP2	0.454666
cnv_RPL14	0.313419
cnv_RPL15	0.410854
cnv_RPL18A	0.488691
cnv_RPL22L1	0.357874
cnv_RPL26L1	0.307365

cnv_RPL28	0.416576
cnv_RPL29	0.395876
cnv_RPL36	0.455884
cnv_RPL36A	0.421401
cnv_RPL36A-HNRNPH2	0.417915
cnv_RPL36AL	0.42813
cnv_RPL37	0.528042
cnv_RPL4	0.56777
cnv_RPL7L1	0.447539
cnv_RPN2	0.374032
cnv_RPP21	0.599296
cnv_RPP25L	0.590519
cnv_RPP40	0.348806
cnv_RPRD1A	0.521356
cnv_RPRD2	0.592994
cnv_RPS10-NUDT3	0.465939
cnv_RPS19BP1	0.381282
cnv_RPS23	0.444721
cnv_RPS27L	0.479049
cnv_RPS3	0.535095
cnv_RPS6KA5	0.414241
cnv_RPS6KB1	0.336375
cnv_RPS6KC1	0.415319
cnv_RPSA	0.366613
cnv_RPSAP58	0.305843
cnv_RPUSD2	0.31152
cnv_RPUSD4	0.582715
cnv_RRAGA	0.411279
cnv_RRM2B	0.51051
cnv_RRNAD1	0.472239
cnv_RRP15	0.555495
cnv_RRP1B	0.53199
cnv_RSAD1	0.404991
cnv_RSBN1	0.480542
cnv_RSF1	0.588058
cnv_RSL24D1	0.55734
cnv_RSPRY1	0.429698
cnv_RSRC1	0.377768
cnv_RSRP1	0.363276
cnv_RTCA	0.573081
cnv_RTCB	0.476403
cnv_RTN4	0.315185
cnv_RTN4IP1	0.368095
cnv_RUNX3	0.359361
cnv_RUSC1	0.532671
cnv_RUSC1-AS1	0.50246
cnv_RUVBL1	0.328748

cnv_RWDD1	0.623321
cnv_RWDD2A	0.493923
cnv_RWDD2B	0.486619
cnv_RWDD3	0.565062
cnv_RYBP	0.298767
cnv_S100A4	0.421138
cnv_S100A6	0.341811
cnv_S100PBP	0.286156
cnv_S1PR4	0.261799
cnv_SAAL1	0.514066
cnv_SAC3D1	0.349087
cnv_SACM1L	0.382612
cnv_SAE1	0.316843
cnv_SAMD8	0.315671
cnv_SAP130	0.292109
cnv_SAP18	0.679863
cnv_SAPCD1	0.421133
cnv_SAR1A	0.353378
cnv_SAR1B	0.394324
cnv_SARAF	0.683535
cnv_SARS	0.490438
cnv_SART3	0.27541
cnv_SASH3	0.323013
cnv_SAT2	0.647787
cnv_SBDS	0.329575
cnv_SBDSP1	0.275999
cnv_SBF2	0.439417
cnv_SC5D	0.396799
cnv_SCAF11	0.225478
cnv_SCAF4	0.321794
cnv_SCAF8	0.505698
cnv_SCAMP1	0.316629
cnv_SCAMP1-AS1	0.406112
cnv_SCAMP3	0.65244
cnv_SCAMP5	0.32203
cnv_SCAND1	0.477298
cnv_SCAP	0.365701
cnv_SCAPER	0.409912
cnv_SCARB2	0.235267
cnv_SCARNA15	0.347592
cnv_SCFD1	0.476149
cnv_SCNM1	0.660972
cnv_SCO1	0.294767
cnv_SCP2	0.326715
cnv_SCRIB	0.462204
cnv_SCRN1	0.237323
cnv_SCYL3	0.573223

cnv_SDAD1	0.282975
cnv_SDCBP	0.227679
cnv_SDE2	0.576253
cnv_SDF2	0.289153
cnv_SDF2L1	0.309852
cnv_SDF4	0.411837
cnv_SDHA	0.322927
cnv_SDHAF2	0.438993
cnv_SDHC	0.75914
cnv_SDR39U1	0.5828
cnv_SEC11A	0.483324
cnv_SEC11C	0.477838
cnv_SEC13	0.412099
cnv_SEC14L1	0.415557
cnv_SEC14L1P1	0.393744
cnv_SEC23A	0.365119
cnv_SEC23B	0.353556
cnv_SEC24A	0.376381
cnv_SEC24B	0.472024
cnv_SEC24C	0.261073
cnv_SEC24D	0.348041
cnv_SEC31A	0.371171
cnv_SEC61A1	0.340217
cnv_SEC61G	0.489372
cnv_SEC63	0.615338
cnv_SECISBP2	0.518138
cnv_SEH1L	0.413107
cnv_SEL1L	0.279088
cnv_SEMA4B	0.246895
cnv_SEMA4D	0.338809
cnv_SENP6	0.513915
cnv_SEPHS1	0.206544
cnv_SEPHS2	0.541398
cnv_SEPT6	0.381383
cnv_SERF2-C15ORF63	0.491361
cnv_SERINC1	0.655615
cnv_SERINC3	0.536083
cnv_SERP1	0.394277
cnv_SERPINB1	0.261282
cnv_SERPINI1	0.304879
cnv_SET	0.491084
cnv_SETBP1	0.318552
cnv_SETD2	0.417774
cnv_SETD6	0.349712
cnv_SETD9	0.407454
cnv_SETX	0.325738
cnv_SF1	0.246474

cnv_SF3A3	0.346976
cnv_SF3B1	0.414871
cnv_SF3B4	0.623494
cnv_SF3B5	0.545359
cnv_SFR1	0.268195
cnv_SFT2D2	0.549595
cnv_SFXN1	0.360015
cnv_SGK3	0.370207
cnv_SH3BGRL	0.140954
cnv_SH3BGRL3	0.371908
cnv_SH3BP5-AS1	0.347564
cnv_SH3GLB1	0.723547
cnv_SH3TC1	0.19528
cnv_SHC1	0.511619
cnv_SHISA2	0.300307
cnv_SHISA5	0.381583
cnv_SHOC2	0.307809
cnv_SHPRH	0.4686
cnv_SHQ1	0.379714
cnv_SIAH1	0.450939
cnv_SIDT1	0.309307
cnv_SIDT2	0.390332
cnv_SIGMAR1	0.427114
cnv_SIK3	0.601449
cnv_SIKE1	0.315557
cnv_SIL1	0.401393
cnv_SIPA1	0.293147
cnv_SIVA1	0.30341
cnv_SKA2	0.258165
cnv_SKIV2L2	0.476934
cnv_SKP1	0.432378
cnv_SKP2	0.383983
cnv_SLAIN1	0.304154
cnv_SLAIN2	0.442139
cnv_SLBP	0.24795
cnv_SLC10A3	0.532254
cnv_SLC12A9	0.482189
cnv_SLC17A5	0.379454
cnv_SLC18B1	0.481933
cnv_SLC19A2	0.53099
cnv_SLC1A4	0.240357
cnv_SLC25A1	0.375111
cnv_SLC25A11	0.369108
cnv_SLC25A14	0.595147
cnv_SLC25A19	0.319483
cnv_SLC25A23	0.360499
cnv_SLC25A26	0.390133

cnv_SLC25A32	0.523415
cnv_SLC25A37	0.423514
cnv_SLC25A38	0.421674
cnv_SLC25A39	0.312451
cnv_SLC25A40	0.376378
cnv_SLC25A43	0.2712
cnv_SLC25A44	0.533325
cnv_SLC25A45	0.44273
cnv_SLC25A46	0.390388
cnv_SLC25A5	0.426557
cnv_SLC2A11	0.349083
cnv_SLC30A7	0.571799
cnv_SLC30A9	0.323054
cnv_SLC31A1	0.282827
cnv_SLC31A2	0.304276
cnv_SLC35A1	0.459941
cnv_SLC35A3	0.528498
cnv_SLC35A4	0.424547
cnv_SLC35A5	0.408631
cnv_SLC35B1	0.302144
cnv_SLC35B2	0.454904
cnv_SLC35B3	0.4744
cnv_SLC35B4	0.424137
cnv_SLC35D1	0.333769
cnv_SLC35F2	0.561713
cnv_SLC37A3	0.308723
cnv_SLC38A9	0.427019
cnv_SLC39A1	0.364516
cnv_SLC39A11	0.280419
cnv_SLC39A14	0.359275
cnv_SLC39A4	0.396549
cnv_SLC39A6	0.534902
cnv_SLC39A7	0.403861
cnv_SLC39A8	0.370704
cnv_SLC40A1	0.31163
cnv_SLC41A1	0.4179
cnv_SLC41A2	0.253939
cnv_SLC41A3	0.539935
cnv_SLC46A3	0.378296
cnv_SLC4A2	0.28298
cnv_SLC50A1	0.437573
cnv_SLC7A1	0.429942
cnv_SLC7A5	0.295769
cnv_SLC7A6	0.459546
cnv_SLC7A6OS	0.368834
cnv_SLC7A7	0.19164
cnv_SLC9A6	0.531888

cnv_SLC9A8	0.335691
cnv_SLIRP	0.508445
cnv_SLK	0.345525
cnv_SLTM	0.525633
cnv_SLU7	0.507287
cnv_SLX4IP	0.285181
cnv_SMA4	0.297147
cnv_SMAD2	0.676951
cnv_SMAD5	0.267732
cnv_SMAP1	0.630446
cnv_SMARCA5	0.364419
cnv_SMARCC1	0.264489
cnv_SMC5	0.336554
cnv_SMC6	0.214968
cnv_SMCHD1	0.465749
cnv_SMG1	0.401256
cnv_SMG1P2	0.444425
cnv_SMG1P5	0.417824
cnv_SMG7	0.46627
cnv_SMG8	0.257276
cnv_SMIM13	0.369801
cnv_SMIM15	0.30697
cnv_SMIM19	0.511275
cnv_SMIM20	0.421637
cnv_SMPD4	0.394998
cnv_SNAP23	0.410062
cnv_SNAP29	0.123209
cnv_SNAP47	0.645183
cnv_SNAPC1	0.337177
cnv_SNAPC3	0.316868
cnv_SNAPIN	0.695862
cnv_SNF8	0.542477
cnv_SNHG1	0.360378
cnv_SNHG15	0.486477
cnv_SNHG9	0.410917
cnv_SNORA1	0.624193
cnv_SNORA11D	0.134954
cnv_SNORA11E	0.15239
cnv_SNORA18	0.624193
cnv_SNORA28	0.477033
cnv_SNORA29	0.57213
cnv_SNORA32	0.624193
cnv_SNORA4	0.289148
cnv_SNORA40	0.624193
cnv_SNORA56	0.443162
cnv_SNORA5B	0.543743
cnv_SNORA6	0.366613

cnv_SNORA62	0.366613
cnv_SNORA63	0.289148
cnv_SNORA72	0.31967
cnv_SNORA78	0.409387
cnv_SNORA8	0.624193
cnv_SNORA81	0.289148
cnv_SNORA9	0.486477
cnv_SNORD16	0.56777
cnv_SNORD18A	0.56777
cnv_SNORD18B	0.56777
cnv_SNORD18C	0.56777
cnv_SNORD19B	0.386934
cnv_SNORD2	0.289148
cnv_SNORD22	0.360378
cnv_SNORD23	0.400264
cnv_SNORD25	0.360378
cnv_SNORD26	0.360378
cnv_SNORD27	0.360378
cnv_SNORD28	0.360378
cnv_SNORD29	0.360378
cnv_SNORD30	0.360378
cnv_SNORD31	0.360378
cnv_SNORD44	0.359526
cnv_SNORD47	0.359526
cnv_SNORD5	0.624193
cnv_SNORD50A	0.346822
cnv_SNORD50B	0.346822
cnv_SNORD74	0.359526
cnv_SNORD76	0.359526
cnv_SNORD77	0.359526
cnv_SNORD79	0.359526
cnv_SNORD80	0.359526
cnv_SNORD81	0.359526
cnv_SNORD84	0.376443
cnv_SNORD88C	0.344671
cnv_SNRNP25	0.318389
cnv_SNRNP27	0.260767
cnv_SNRNP48	0.282275
cnv_SNRNP70	0.474481
cnv_SNRPD1	0.462339
cnv_SNRPD2	0.328864
cnv_SNRPD3	0.459148
cnv_SNRPE	0.552563
cnv_SNUPN	0.53317
cnv_SNURF	0.383046
cnv_SNW1	0.551538
cnv_SNX1	0.513548

cnv_SNX13	0.353853
cnv_SNX14	0.466981
cnv_SNX27	0.446815
cnv_SNX3	0.71046
cnv_SNX4	0.236738
cnv_SNX6	0.399506
cnv_SOAT1	0.298934
cnv_SOCS4	0.421058
cnv_SOCS5	0.260956
cnv_SOD1	0.42359
cnv_SOD2	0.514583
cnv_SON	0.566596
cnv_SORD	0.392298
cnv_SORT1	0.324387
cnv_SP1	0.253953
cnv_SP4	0.267661
cnv_SPAG5-AS1	0.27047
cnv_SPAG7	0.503242
cnv_SPAG9	0.373996
cnv_SPATA5L1	0.41413
cnv_SPCS2	0.385704
cnv_SPECC1L	0.423241
cnv_SPEF2	0.37417
cnv_SPG20	0.368064
cnv_SPHAR	0.484465
cnv_SPINK2	0.210217
cnv_SPINT1	0.347749
cnv_SPOP	0.279257
cnv_SPRY1	0.300574
cnv_SPRYD3	0.318747
cnv_SPSB2	0.328191
cnv_SPTY2D1	0.40634
cnv_SQLE	0.48382
cnv_SQRDL	0.549538
cnv_SQSTM1	0.306905
cnv_SRA1	0.621915
cnv_SREK1	0.429135
cnv_SREK1IP1	0.391285
cnv_SRGAP2	0.328578
cnv_SRGAP2C	0.195071
cnv_SRGN	0.260767
cnv_SRI	0.274373
cnv_SRP14	0.27803
cnv_SRP19	0.433239
cnv_SRP54	0.361094
cnv_SRP68	0.457958
cnv_SRP72	0.354471

cnv_SRP9	0.439601
cnv_SRPK1	0.385441
cnv_SRPK2	0.419745
cnv_SRPRB	0.419981
cnv_SRRD	0.413308
cnv_SRRM1	0.330941
cnv_SRSF11	0.581374
cnv_SRSF3	0.338753
cnv_SRSF4	0.32475
cnv_SRSF5	0.639305
cnv_SRSF7	0.301763
cnv_SRSF8	0.423345
cnv_SRSF9	0.58159
cnv_SS18	0.474939
cnv_SS18L1	0.369809
cnv_SS18L2	0.410248
cnv_SSB	0.320812
cnv_SSBP1	0.432236
cnv_SSBP3	0.358931
cnv_SSBP4	0.404324
cnv_SSFA2	0.24164
cnv_SSR1	0.526048
cnv_SSR2	0.489572
cnv_SSR3	0.415778
cnv_SSR4	0.238263
cnv_SSRP1	0.398988
cnv_SSSCA1	0.491263
cnv_ST3GAL1	0.396851
cnv_ST7	0.554954
cnv_ST7-OT3	0.507997
cnv_ST8SIA4	0.407467
cnv_STAG1	0.252818
cnv_STAG2	0.503269
cnv_STAG3L1	0.381504
cnv_STAG3L2	0.356313
cnv_STAG3L3	0.360596
cnv_STAG3L4	0.54823
cnv_STAG3L5P-PVRIG2P-PILRB	0.476836
cnv_STAM	0.255207
cnv_STAP2	0.309409
cnv_STARD3NL	0.339006
cnv_STARD5	0.357124
cnv_STARD9	0.333576
cnv_STAT2	0.36607
cnv_STEAP1B	0.388038
cnv_STIM2	0.445773
cnv_STIP1	0.331013

cnv_STK10	0.303857
cnv_STK17B	0.297388
cnv_STK25	0.314501
cnv_STK4	0.28517
cnv_STOM	0.302572
cnv_STOML2	0.457447
cnv_STRAP	0.495788
cnv_STRIP1	0.400824
cnv_STRN3	0.338755
cnv_STT3B	0.203656
cnv_STX10	0.411049
cnv_STX11	0.38235
cnv_STX16	0.368571
cnv_STX17	0.517143
cnv_STX5	0.287381
cnv_STX7	0.486685
cnv_STXBP3	0.415501
cnv_SUB1	0.304727
cnv_SUCLA2	0.348553
cnv_SUCLG2	0.292203
cnv_SUCO	0.375962
cnv_SUDS3	0.28518
cnv_SUGT1	0.598944
cnv_SUMF2	0.355163
cnv_SUMO1	0.229873
cnv_SUN2	0.17171
cnv_SUPT16H	0.44365
cnv_SURF2	0.293308
cnv_SURF4	0.3182
cnv_SVIP	0.360227
cnv_SWSAP1	0.325058
cnv_SWT1	0.458292
cnv_SYAP1	0.345033
cnv_SYNCRIP	0.487385
cnv_SYNE2	0.303321
cnv_SYNGR2	0.266078
cnv_SYNJ2BP	0.509526
cnv_SYNJ2BP-COX16	0.532026
cnv_SYTL1	0.29856
cnv_SYVN1	0.295562
cnv_TAB2	0.452192
cnv_TACC1	0.361534
cnv_TAF11	0.407816
cnv_TAF12	0.357451
cnv_TAF13	0.350918
cnv_TAF1A	0.484503
cnv_TAF1A-AS1	0.393167

cnv_TAF1D	0.60696
cnv_TAF2	0.597471
cnv_TAF6	0.300851
cnv_TAF7	0.416078
cnv_TAF9	0.392725
cnv_TAGAP	0.30368
cnv_TAGLN2	0.545521
cnv_TALDO1	0.285688
cnv_TANGO2	0.380256
cnv_TANK	0.26039
cnv_TAP1	0.303173
cnv_TAP2	0.36378
cnv_TAPBP	0.263581
cnv_TAPBPL	0.356445
cnv_TARBP1	0.366395
cnv_TARDBP	0.247535
cnv_TARS2	0.756168
cnv_TATDN3	0.55175
cnv_TAX1BP1	0.427301
cnv_TAX1BP3	0.427453
cnv_TBC1D1	0.302219
cnv_TBC1D10B	0.396232
cnv_TBC1D15	0.320687
cnv_TBC1D23	0.241854
cnv_TBC1D3	0.261712
cnv_TBC1D30	0.229032
cnv_TBC1D3C	0.274911
cnv_TBC1D3H	0.24173
cnv_TBC1D9	0.311759
cnv_TBCA	0.454892
cnv_TBCC	0.345252
cnv_TBCCD1	0.396794
cnv_TBCE	0.695041
cnv_TBCEL	0.379389
cnv_TBCK	0.284376
cnv_TBK1	0.217339
cnv_TBPL1	0.57677
cnv_TCEAL1	0.414287
cnv_TCEAL3	0.326201
cnv_TCEAL4	0.315241
cnv_TCEAL8	0.343213
cnv_TCERG1	0.282544
cnv_TCF12	0.467349
cnv_TCFL5	0.291054
cnv_TCN2	0.29326
cnv_TCP1	0.57213
cnv_TCTN1	0.336786

cnv_TDG	0.276922
cnv_TDP2	0.332261
cnv_TDRD3	0.493152
cnv_TDRD7	0.423468
cnv_TEKT4P2	0.25333
cnv_TERF1	0.452328
cnv_TERF2IP	0.643455
cnv_TESK2	0.435657
cnv_TET1	0.187053
cnv_TET2	0.332838
cnv_TEX10	0.55232
cnv_TEX30	0.222113
cnv_TFDP2	0.423692
cnv_TFG	0.38358
cnv_TFPT	0.386678
cnv_TFRC	0.385332
cnv_TGDS	0.579774
cnv_TGIF2-C20orf24	0.466394
cnv_TGOLN2	0.310617
cnv_THAP1	0.322273
cnv_THAP7	0.193288
cnv_THEM4	0.560171
cnv_THEMIS2	0.283619
cnv_THG1L	0.605267
cnv_THOC1	0.477594
cnv_THOC2	0.483059
cnv_THOC7	0.452046
cnv_THOP1	0.304843
cnv_THRIL	0.261404
cnv_THSD1	0.46788
cnv_THTPA	0.355325
cnv_THUMPD1	0.477103
cnv_THUMPD3	0.390508
cnv_THYN1	0.5092
cnv_TICAM2	0.363897
cnv_TIFA	0.334874
cnv_TIMELESS	0.327821
cnv_TIMM10	0.452909
cnv_TIMM10B	0.58286
cnv_TIMM17A	0.659246
cnv_TIMM21	0.456114
cnv_TIMM23B	0.336932
cnv_TIMM44	0.441368
cnv_TIMM8B	0.557607
cnv_TIMM9	0.575608
cnv_TIMMDC1	0.365475
cnv_TIPIN	0.329517

cnv_TIPRL	0.609536
cnv_TK1	0.245805
cnv_TKT	0.325789
cnv_TLR1	0.367891
cnv_TLR6	0.392909
cnv_TM2D1	0.534714
cnv_TM2D2	0.481067
cnv_TM7SF2	0.312034
cnv_TM9SF2	0.584199
cnv_TM9SF4	0.340217
cnv_TMBIM1	0.255172
cnv_TMBIM6	0.438407
cnv_TMCO1	0.611574
cnv_TMCO6	0.388931
cnv_TMED10	0.571243
cnv_TMED2	0.478469
cnv_TMED5	0.671399
cnv_TMED9	0.312111
cnv_TMEM106B	0.457525
cnv_TMEM107	0.314647
cnv_TMEM115	0.261246
cnv_TMEM120A	0.360304
cnv_TMEM123	0.687589
cnv_TMEM126A	0.619491
cnv_TMEM128	0.290256
cnv_TMEM133	0.506902
cnv_TMEM138	0.547408
cnv_TMEM147	0.252064
cnv_TMEM14A	0.382534
cnv_TMEM14B	0.626498
cnv_TMEM14C	0.510286
cnv_TMEM161B	0.635751
cnv_TMEM161B-AS1	0.474303
cnv_TMEM167A	0.37781
cnv_TMEM167B	0.579349
cnv_TMEM179B	0.335739
cnv_TMEM181	0.586451
cnv_TMEM183A	0.642249
cnv_TMEM185A	0.565076
cnv_TMEM185B	0.270741
cnv_TMEM187	0.491162
cnv_TMEM192	0.40862
cnv_TMEM2	0.253077
cnv_TMEM208	0.523031
cnv_TMEM209	0.439699
cnv_TMEM214	0.350064
cnv_TMEM216	0.487499

cnv_TMEM220	0.289804
cnv_TMEM230	0.498344
cnv_TMEM243	0.33919
cnv_TMEM245	0.349952
cnv_TMEM248	0.461491
cnv_TMEM251	0.37745
cnv_TMEM254	0.228941
cnv_TMEM256	0.405884
cnv_TMEM260	0.565068
cnv_TMEM261	0.449045
cnv_TMEM30A	0.553528
cnv_TMEM41A	0.401836
cnv_TMEM41B	0.365437
cnv_TMEM42	0.352903
cnv_TMEM44-AS1	0.346878
cnv_TMEM5	0.40004
cnv_TMEM50A	0.396944
cnv_TMEM50B	0.294127
cnv_TMEM55A	0.307268
cnv_TMEM55B	0.513279
cnv_TMEM59	0.441741
cnv_TMEM60	0.299603
cnv_TMEM64	0.380844
cnv_TMEM68	0.376769
cnv_TMEM87B	0.371055
cnv_TMEM9	0.629921
cnv_TMEM9B	0.347245
cnv_TMF1	0.334649
cnv_TMOD3	0.303051
cnv_TMPO	0.274279
cnv_TMPPE	0.408391
cnv_TMSB15A	0.198783
cnv_TMTC4	0.431278
cnv_TMUB1	0.172983
cnv_TMX1	0.380985
cnv_TMX2	0.48409
cnv_TMX3	0.481789
cnv_TMX4	0.213528
cnv_TNFAIP8L2-SCNM1	0.660972
cnv_TNFRSF10A	0.390586
cnv_TNFRSF10D	0.349313
cnv_TNFSF13	0.243068
cnv_TNKS2	0.314776
cnv_TNPO3	0.542149
cnv_TNRC6B	0.414042
cnv_TOMM20	0.523069
cnv_TOMM34	0.447531

cnv_TOMM40L	0.48783
cnv_TOMM5	0.565384
cnv_TOMM6	0.551954
cnv_TOMM7	0.57086
cnv_TOP1	0.275301
cnv_TOP2B	0.28659
cnv_TOPBP1	0.356203
cnv_TOPORS	0.337368
cnv_TOPORS-AS1	0.413644
cnv_TOR1AIP1	0.529461
cnv_TOR1B	0.429112
cnv_TOR3A	0.48852
cnv_TP53	0.310123
cnv_TP53BP2	0.488404
cnv_TP53TG1	0.462592
cnv_TP73-AS1	0.200078
cnv_TPD52	0.321492
cnv_TPK1	0.459304
cnv_TPM4	0.422102
cnv_TPP2	0.442423
cnv_TPR	0.536626
cnv_TPRG1L	0.502653
cnv_TRA2B	0.287828
cnv_TRAF6	0.45588
cnv_TRAK2	0.252399
cnv_TRAM1	0.330787
cnv_TRAM2-AS1	0.279052
cnv_TRAPPC1	0.44592
cnv_TRAPPC10	0.419296
cnv_TRAPPC4	0.411068
cnv_TRAPPC5	0.314272
cnv_TRAPPC6B	0.405542
cnv_TRAPPC8	0.515252
cnv_TRIM13	0.508376
cnv_TRIM21	0.384184
cnv_TRIM22	0.328314
cnv_TRIM24	0.326587
cnv_TRIM27	0.403908
cnv_TRIM28	0.279836
cnv_TRIM33	0.623684
cnv_TRIM37	0.465133
cnv_TRIM38	0.311173
cnv_TRIM44	0.492457
cnv_TRIM56	0.304043
cnv_TRIM68	0.378048
cnv_TRIM69	0.426797
cnv_TRIP11	0.435866

cnv_TRIP4	0.409683
cnv_TRIP6	0.3724
cnv_TRMT10C	0.355875
cnv_TRMT11	0.601327
cnv_TRMT112	0.529754
cnv_TRMT12	0.493525
cnv_TRMT13	0.347155
cnv_TRMT1L	0.422079
cnv_TRMT2B	0.424473
cnv_TRMT6	0.369694
cnv_TRNT1	0.381498
cnv_TROVE2	0.704791
cnv_TRRAP	0.336316
cnv_TRUB2	0.403999
cnv_TSC1	0.264602
cnv_TSEN15	0.656561
cnv_TSEN34	0.364288
cnv_TSG101	0.562698
cnv_TSHZ1	0.435411
cnv_TSN	0.310193
cnv_TSNAX	0.580305
cnv_TSPAN31	0.373897
cnv_TSPO	0.38816
cnv_TSPYL4	0.329754
cnv_TSTA3	0.320966
cnv_TSTD1	0.543344
cnv_TTC1	0.479706
cnv_TTC13	0.589144
cnv_TTC14	0.298441
cnv_TTC19	0.556481
cnv_TTC3	0.297213
cnv_TTC37	0.503433
cnv_TTC4	0.325899
cnv_TTC8	0.335566
cnv_TTC9C	0.537098
cnv_TTF1	0.365011
cnv_TTI1	0.470174
cnv_TTI2	0.378239
cnv_TTLL7	0.421394
cnv_TTPAL	0.417476
cnv_TUBA1A	0.164901
cnv_TUBA1B	0.354631
cnv_TUBA1C	0.369749
cnv_TUBD1	0.243227
cnv_TUBG1	0.262541
cnv_TUBGCP4	0.559218
cnv_TUBGCP5	0.422197

cnv_TVP23B	0.34851
cnv_TVP23C	0.323786
cnv_TVP23C-CDRT4	0.298201
cnv_TWISTNB	0.354166
cnv_TWSG1	0.384526
cnv_TXLNG	0.370869
cnv_TXN	0.547633
cnv_TXNDC11	0.313211
cnv_TXNDC12	0.471634
cnv_TXNDC15	0.543489
cnv_TXNL1	0.552293
cnv_TYW1	0.497737
cnv_TYW3	0.42114
cnv_U2AF1	0.399404
cnv_U2AF1L4	0.293943
cnv_U2SURP	0.318518
cnv_UAP1	0.602743
cnv_UBA2	0.248508
cnv_UBA3	0.372922
cnv_UBA5	0.485327
cnv_UBA6	0.271961
cnv_UBA7	0.302292
cnv_UBAC2	0.437898
cnv_UBAP2	0.422019
cnv_UBB	0.394249
cnv_UBE2A	0.509886
cnv_UBE2D2	0.295266
cnv_UBE2E1	0.210575
cnv_UBE2E2	0.341
cnv_UBE2G1	0.327135
cnv_UBE2J1	0.410941
cnv_UBE2K	0.374611
cnv_UBE2L6	0.460022
cnv_UBE2M	0.271711
cnv_UBE2Q2	0.616212
cnv_UBE2R2	0.406845
cnv_UBE2T	0.493975
cnv_UBE4A	0.55633
cnv_UBFD1	0.449036
cnv_UBL3	0.451091
cnv_UBL4A	0.459024
cnv_UBL5	0.508151
cnv_UBL7	0.595116
cnv_UBL7-AS1	0.379843
cnv_UBLCP1	0.448382
cnv_UBP1	0.508798
cnv_UBQLN1	0.320182

cnv_UBQLN2	0.2922
cnv_UBR1	0.428471
cnv_UBR5	0.582025
cnv_UBR7	0.359622
cnv_UBXN2A	0.242323
cnv_UBXN2B	0.421783
cnv_UBXN4	0.327613
cnv_UBXN7	0.30849
cnv_UCHL3	0.596656
cnv_UCHL5	0.634841
cnv_UCK2	0.45859
cnv_UEVLD	0.349015
cnv_UFC1	0.65666
cnv_UFD1L	0.393031
cnv_UFL1	0.493854
cnv_UFM1	0.521746
cnv_UHMK1	0.634011
cnv_UHRF2	0.48426
cnv_ULK3	0.67123
cnv_UMPS	0.403564
cnv_UNC13B	0.283979
cnv_UPF2	0.312271
cnv_UPF3B	0.464183
cnv_UPK3BL	0.37583
cnv_UPP1	0.216558
cnv_UQCR10	0.276359
cnv_UQCRC1	0.385088
cnv_UQCRFS1	0.508638
cnv_UQCRQ	0.37767
cnv_URB2	0.547632
cnv_URGCP	0.393033
cnv_URGCP-MRPS24	0.528112
cnv_UROD	0.390238
cnv_USE1	0.591291
cnv_USMG5	0.428108
cnv_USO1	0.461593
cnv_USP1	0.329099
cnv_USP10	0.479714
cnv_USP12	0.390847
cnv_USP14	0.495201
cnv_USP16	0.521656
cnv_USP20	0.440082
cnv_USP21	0.552041
cnv_USP24	0.411676
cnv_USP25	0.349185
cnv_USP28	0.500536
cnv_USP3	0.497473

cnv_USP30	0.386078
cnv_USP33	0.650471
cnv_USP37	0.173429
cnv_USP47	0.458949
cnv_USP54	0.288801
cnv_USP8	0.387644
cnv_USPL1	0.647809
cnv_UTP14C	0.309505
cnv_UTP15	0.338573
cnv_UTP20	0.297075
cnv_UTP23	0.535354
cnv_UTRN	0.477448
cnv_UVRAG	0.530429
cnv_VAMP1	0.28726
cnv_VAMP2	0.381561
cnv_VAMP3	0.418394
cnv_VAMP4	0.570971
cnv_VAMP8	0.3188
cnv_VARS	0.403262
cnv_VARS2	0.592019
cnv_VAT1	0.225373
cnv_VBP1	0.561862
cnv_VCL	0.217236
cnv_VCP	0.434301
cnv_VCPKMT	0.263453
cnv_VDAC1	0.452744
cnv_VDAC3	0.401357
cnv_VEGFA	0.269158
cnv_VEZF1	0.278325
cnv_VEZT	0.379015
cnv_VGLL4	0.419781
cnv_VILL	0.249208
cnv_VIM	0.304321
cnv_VIPAS39	0.534311
cnv_VKORC1	0.264218
cnv_VMP1	0.224777
cnv_VOPP1	0.315488
cnv_VPS13A	0.31524
cnv_VPS13B	0.510795
cnv_VPS13C	0.384364
cnv_VPS25	0.366089
cnv_VPS28	0.354096
cnv_VPS35	0.349117
cnv_VPS36	0.579403
cnv_VPS37A	0.477354
cnv_VPS4B	0.472125
cnv_VPS51	0.490808

cnv_VPS54	0.275819
cnv_VPS8	0.358502
cnv_VRK2	0.242833
cnv_VTA1	0.475524
cnv_VTI1B	0.639686
cnv_VWA8	0.647272
cnv_WARS	0.303921
cnv_WASF2	0.39381
cnv_WBP1	0.302381
cnv_WBP11	0.54628
cnv_WBP1L	0.337289
cnv_WBP2	0.260133
cnv_WBP4	0.532215
cnv_WDFY2	0.367629
cnv_WDR18	0.345948
cnv_WDR26	0.553092
cnv_WDR36	0.311279
cnv_WDR4	0.43378
cnv_WDR41	0.476161
cnv_WDR46	0.552066
cnv_WDR47	0.415079
cnv_WDR48	0.345849
cnv_WDR53	0.411025
cnv_WDR61	0.564751
cnv_WDR70	0.487319
cnv_WDR73	0.450102
cnv_WDR82	0.439218
cnv_WDR83OS	0.444627
cnv_WDR89	0.263464
cnv_WDSUB1	0.26049
cnv_WFS1	0.390743
cnv_WHAMM	0.51262
cnv_WRB	0.316328
cnv_WRN	0.506606
cnv_WSB1	0.203383
cnv_WSB2	0.293957
cnv_WWOX	0.488579
cnv_WWP1	0.442669
cnv_XIAP	0.433452
cnv_XPA	0.563273
cnv_XPC	0.389663
cnv_XPO4	0.511501
cnv_XPO5	0.514215
cnv_XPO7	0.70943
cnv_XPOT	0.339011
cnv_XPR1	0.451781
cnv_XRCC4	0.602812

cnv_XRN1	0.318243
cnv_XRRA1	0.365854
cnv_YBX1	0.368346
cnv_YES1	0.43295
cnv_YIF1A	0.406069
cnv_YIPF1	0.538858
cnv_YIPF3	0.400904
cnv_YIPF5	0.478984
cnv_YKT6	0.546741
cnv_YLPM1	0.705927
cnv_YPEL3	0.423884
cnv_YTHDC2	0.307268
cnv_YTHDF2	0.444329
cnv_YTHDF3	0.435044
cnv_YWHAB	0.340212
cnv_YWHAG	0.298513
cnv_YWHAH	0.245542
cnv_YWHAQ	0.358818
cnv_YWHAZ	0.536672
cnv_YY1AP1	0.617774
cnv_ZADH2	0.496918
cnv_ZBED3	0.49709
cnv_ZBED6CL	0.465192
cnv_ZBTB11	0.26982
cnv_ZBTB2	0.332621
cnv_ZBTB21	0.333122
cnv_ZBTB24	0.282168
cnv_ZBTB25	0.348667
cnv_ZBTB26	0.350603
cnv_ZBTB33	0.47634
cnv_ZBTB34	0.301352
cnv_ZBTB38	0.29946
cnv_ZBTB4	0.525088
cnv_ZBTB41	0.40862
cnv_ZBTB44	0.502754
cnv_ZBTB5	0.405777
cnv_ZBTB8OS	0.451072
cnv_ZC2HC1A	0.246453
cnv_ZC3H11A	0.477689
cnv_ZC3H13	0.497015
cnv_ZC3H14	0.541397
cnv_ZC3H7A	0.396229
cnv_ZC3HAV1	0.313362
cnv_ZC3HC1	0.507088
cnv_ZCCHC10	0.311356
cnv_ZCCHC11	0.460989
cnv_ZCCHC6	0.373013

cnv_ZCCHC7	0.321053
cnv_ZCCHC9	0.481228
cnv_ZDHHC12	0.330143
cnv_ZDHHC13	0.392129
cnv_ZDHHC14	0.263479
cnv_ZDHHC17	0.422784
cnv_ZDHHC2	0.58611
cnv_ZDHHC20	0.397924
cnv_ZDHHC23	0.380963
cnv_ZDHHC6	0.273996
cnv_ZDHHC9	0.464964
cnv_ZFAND2A	0.508114
cnv_ZFAND2B	0.254177
cnv_ZFAND6	0.309706
cnv_ZFAT	0.264464
cnv_ZFC3H1	0.236464
cnv_ZFP62	0.443219
cnv_ZFP90	0.337901
cnv_ZFP91	0.475046
cnv_ZFYVE21	0.38836
cnv_ZFYVE26	0.576704
cnv_ZGPAT	0.419146
cnv_ZHX1	0.446649
cnv_ZKSCAN8	0.371982
cnv_ZMAT1	0.348528
cnv_ZMAT2	0.481117
cnv_ZMAT3	0.333407
cnv_ZMYM4	0.321169
cnv_ZMYND8	0.279324
cnv_ZNF101	0.429939
cnv_ZNF106	0.444243
cnv_ZNF12	0.414122
cnv_ZNF121	0.514892
cnv_ZNF131	0.583541
cnv_ZNF136	0.425022
cnv_ZNF146	0.344105
cnv_ZNF177	0.314846
cnv_ZNF195	0.430266
cnv_ZNF20	0.386712
cnv_ZNF200	0.282268
cnv_ZNF202	0.554716
cnv_ZNF211	0.367637
cnv_ZNF215	0.398896
cnv_ZNF224	0.351031
cnv_ZNF23	0.403753
cnv_ZNF24	0.583334
cnv_ZNF251	0.331021

cnv_ZNF264	0.372683
cnv_ZNF266	0.366307
cnv_ZNF275	0.398046
cnv_ZNF277	0.39234
cnv_ZNF281	0.447356
cnv_ZNF286B	0.213608
cnv_ZNF296	0.245197
cnv_ZNF302	0.303162
cnv_ZNF304	0.261305
cnv_ZNF317	0.447936
cnv_ZNF318	0.364842
cnv_ZNF326	0.359303
cnv_ZNF35	0.329526
cnv_ZNF395	0.569729
cnv_ZNF398	0.269115
cnv_ZNF410	0.570157
cnv_ZNF419	0.277808
cnv_ZNF426	0.441342
cnv_ZNF430	0.263137
cnv_ZNF451	0.212299
cnv_ZNF510	0.315684
cnv_ZNF514	0.328517
cnv_ZNF532	0.390547
cnv_ZNF548	0.403212
cnv_ZNF559-ZNF177	0.324034
cnv_ZNF561	0.499926
cnv_ZNF562	0.488595
cnv_ZNF564	0.330489
cnv_ZNF565	0.394955
cnv_ZNF574	0.32288
cnv_ZNF581	0.395767
cnv_ZNF587	0.305049
cnv_ZNF622	0.549041
cnv_ZNF625	0.490425
cnv_ZNF625-ZNF20	0.517411
cnv_ZNF644	0.458766
cnv_ZNF652	0.322551
cnv_ZNF655	0.511689
cnv_ZNF689	0.556492
cnv_ZNF691	0.269864
cnv_ZNF700	0.472803
cnv_ZNF721	0.349787
cnv_ZNF764	0.53766
cnv_ZNF770	0.467812
cnv_ZNF776	0.291376
cnv_ZNF786	0.293003
cnv_ZNF805	0.402398

cnv_ZNF844	0.305962
cnv_ZNF862	0.341917
cnv_ZNHIT1	0.50349
cnv_ZNHIT6	0.329953
cnv_ZPR1	0.602089
cnv_ZRANB2	0.469014
cnv_ZSCAN16	0.296715
cnv_ZSCAN21	0.518437
cnv_ZSCAN26	0.29941
cnv_ZSCAN29	0.516164
cnv_ZSWIM6	0.369824
cnv_ZUFSP	0.478173
cnv_ZW10	0.605897
cnv_ZWILCH	0.27851
cnv_ZYG11B	0.471404
cnv_ZZZ3	0.492597

Supplementary Table 4. Comparison of M3CN with public network/pathway database

	Observed (percent)	meanR	sdR	p-value
HumanNet	0.0795	0.011	0.0014	~0
HPRD	0.0159	0.002	0.0008	6.38×10^{-68}
STRING	0.0788	0.005	0.0012	~0
KEGG	0.3152	0.047	0.0092	3.92×10^{-187}
Hallmark	0.3715	0.089	0.0124	3.44×10^{-115}
GO	0.1327	0.048	0.0055	8.18×10^{-54}
InnateDB	0.0439	0.002	0.0042	9.69×10^{-24}

Supplementary Table 5. Comparison of prognostic signatures. Number of overlapping genes are listed in upper part, p-values from Fisher test listed in bottom.

	Burington_92	Decaux_15	Hose_50	Kassamba_22	Kuiper_92	Reme_19	Shaughnes_70	Zhan_52
Burington_92		1	0	1	3	0	1	1
Decaux_15	6.09E-02		0	0	1	0	0	0
Hose_50	1.00E+00	1.00E+00		1	6	2	3	0
Kassambara_22	8.81E-02	1.00E+00	4.88E-02		1	0	0	0
Kuiper_92	6.59E-03	6.03E-02	5.82E-08	8.72E-02		0	2	0
Reme_19	1.00E+00	1.00E+00	8.45E-04	1.00E+00	1.00E+00		0	0
Shaughnessy_70	2.39E-01	1.00E+00	4.37E-04	1.00E+00	2.97E-02	1.00E+00		0
Zhan_52	1.96E-01	1.00E+00	1.00E+00	1.00E+00	1.00E+00	1.00E+00	1.00E+00	

Supplementary Table 6. Enrichment of signature genes in chromosomes

Signatures	Chrom.	p value	number of genes	Genes
Burington_92	chr1	2.70E-02	13	MACF1,GADD45A,SARS,EIF4G3,SF3B4,STXBP3, UCK2,AGL,CD53,C1orf38,ILF2,AMPD1,PDE4B
Decaux_15	chr12	1.73E-02	3	MGST1,CPSF6,ALDH2
Dickens_6	chr13	7.35E-02	1	ITM2B
Hose_50	chr10	5.52E-03	6	CEP55,KIF11,MKI67,PGAM1,MCM10,ZWINT
Kassambara_22	chr15	3.08E-02	3	FANCI,RAD51,BLM
Kryukov_12	chr15	5.41E-02	2	RAD51,BUB1B
Kuiper_92	chr4	5.06E-02	7	ST13,GABRA4,AIMP1,PGM2,FGFR3,NCAPG,CENPE
Reme_19	chr15	1.28E-01	2	NUSAP1,EHD4
Shaughnessy_70	chr1	5.54E-05	14	CKS1B,IFI16,AHCYL1,TRIM33,CTBS,FUCA1,AIM2, KIF14,EVI5,ASPM,PSMD4,TAGLN2,ENO1,OPN3
Wu_10	chr8	3.95E-02	2	MYBL1,PTP4A3
Zhan_52	chr5	2.11E-02	5	ZNF131,VDAC1,TCERG1,LARS,SMAD5

Supplementary Table 7. Key regulators identified in M3CN for three signatures

Key regulators	signatures	p-values
NOP16	Shaughnessy_70	1.38E-07
CECR5	Shaughnessy_70	4.36E-06
CDK1	Hose_50	0
DTL	Hose_50	0
CENPA	Hose_50	1.58E-12
TROAP	Hose_50	3.12E-11
OIP5	Hose_50	1.07E-08
CENPU	Hose_50	1.16E-08
NUSAP1	Hose_50	2.95E-07
CEP78	Hose_50	2.71E-06
DNMT1	Hose_50	4.05E-06
CDKN2A	Hose_50	5.78E-06
MELK	Kuiper_92	2.83E-11
TPX2	Kuiper_92	9.65E-11
NCAPG2	Kuiper_92	2.13E-06

Supplementary Table 8. Nodes in the prognostic subnetwork.

Node name (Gene symbol)
ADSL
APOBEC3B
ASF1B
AURKB
BIRC5
BRCA1
BUB1B
C11orf48
CCNB2
CCT2
CDC20
CDC25A
CDC45
CDCA3
CDCA5
CDCA8
CDK1
CDK2
CDKN2A
CDKN3
CDT1
CECR5
CENPA
CENPE
CENPH
CENPJ
CENPM
CENPU
CENPW
CEP78
CHEK1
CHTF18
CIRH1A
CKS1B
CPSF3
CRELD2
CTNNAL1
DDIAS
DHFR
DNA2
DNMT1
DONSON
DTL
DUT

E2F2
ENO1
ESPL1
EVI2B
EXO1
EXOSC4
EZH2
FABP5
FANCI
FARSA
FEN1
FOXM1
GINS1
GMNN
H2AFZ
HAUS1
HJURP
HK2
HMGB2
HMGB3
HMGB3P1
HMMR
KARS
KIAA0101
KIF18B
KIF2C
KIF4A
KIFC1
KNSTRN
KPNA2
LMNB1
LOC100505715
LOC102288414
LRR1
MAD2L1
MARS2
MCM2
MCM3
MCM4
MCM5
MCM6
MCM7
MELK
MKI67
MRPL17
MRTO4
MSH2

MSH5
MSH5-SAPCD1
MTIF2
MYC
NCAPD3
NCAPG
NCAPG2
NDC80
NDUFV3
NIFK
NOM1
NOP16
NUDT1
NUP205
NUSAP1
OIP5
OSTC
OXCT1
PAK1IP1
PAM
PBK
PCNA
PFKM
PHF19
PHF5A
PODXL
POLA1
POLD1
POLE
POLE2
POLQ
PRC1
PRIM1
PRKCI
PSMC3IP
PTS
PTTG1
RACGAP1
RAD51AP1
RAD51C
RAN
RFC2
RFC4
RMI1
RMI2
RNASEH2A
RRM1

RRM2
RUVBL1
SAPCD1
SAPCD2
SELK
SHCBP1
SLFN13
SMC2
SNX5
SORD
SPAG5
SPDL1
SSSCA1
STIL
STMN1
SUV39H2
TACC3
TIMM8B
TK1
TMPO
TNFRSF13C
TOPBP1
TPX2
TRIP13
TROAP
TRRAP
TTLL12
TYMS
UBE2C
UBE2T
UHRF1
WDR4
WDR75
WEE1
YEATS4
ZC3H8
ZNF266
ZNF367
ZWILCH
ZWINT

Supplementary Table 9. Regulations among nodes in the prognostic subnetwork.

parent node		child node
ADSL	->	CECR5
ADSL	->	CPSF3
ADSL	->	ENO1
ADSL	->	MRTO4
ADSL	->	MTIF2
ADSL	->	OXCT1
AURKB	->	CDC20
AURKB	->	CHTF18
AURKB	->	E2F2
AURKB	->	POLD1
AURKB	->	POLE
BIRC5	->	CDT1
BIRC5	->	CENPW
BIRC5	->	KIF2C
BIRC5	->	NUDT1
BIRC5	->	TACC3
BIRC5	->	TK1
BIRC5	->	TROAP
BUB1B	->	KIF4A
BUB1B	->	MAD2L1
BUB1B	->	MCM2
BUB1B	->	MELK
BUB1B	->	OIP5
BUB1B	->	PRC1
CCNB2	->	PTTG1
CCT2	->	ADSL
CDCA3	->	CDC25A
CDCA3	->	CDCA8
CDCA3	->	CDK2
CDCA3	->	CENPA
CDCA3	->	UBE2C
CDK1	->	CCNB2
CDK1	->	CDC20
CDK1	->	CDKN3
CDK1	->	CENPE
CDK1	->	H2AFZ
CDK1	->	NCAPG
CDK1	->	NDC80
CDK1	->	TYMS
CDK1	->	UBE2C
CDT1	->	CDCA3
CENPE	->	LMNB1
CENPH	->	YEATS4
CENPU	->	ZNF367
CPSF3	->	HAUS1

DTL	->	APOBEC3B
DTL	->	GINS1
DTL	->	MCM6
DTL	->	MELK
DTL	->	UBE2T
DUT	->	ZNF266
ESPL1	->	HJURP
ESPL1	->	KIFC1
ESPL1	->	TROAP
EXOSC4	->	FABP5
EXOSC4	->	NDUFV3
EXOSC4	->	PODXL
EZH2	->	CDKN3
FARSA	->	EXOSC4
FARSA	->	RNASEH2A
GINS1	->	CHEK1
GINS1	->	CKS1B
GINS1	->	FEN1
GINS1	->	MCM2
GINS1	->	MCM3
GINS1	->	MSH2
GINS1	->	POLE2
GINS1	->	PRIM1
GINS1	->	RAD51AP1
GINS1	->	RMI2
GINS1	->	SLFN13
GINS1	->	STIL
GINS1	->	SUV39H2
GINS1	->	TPX2
H2AFZ	->	HMGB2
H2AFZ	->	LOC100505715
HAUS1	->	MSH2
HAUS1	->	RAD51AP1
HMGB3	->	HMGB3P1
HMMR	->	PTTG1
KARS	->	CIRH1A
KIAA0101	->	CCNB2
KIAA0101	->	TRIP13
KNSTRN	->	SPDL1
LOC102288414	->	C11orf48
MAD2L1	->	CENPU
MAD2L1	->	LRR1
MAD2L1	->	RFC4
MAD2L1	->	RNASEH2A
MCM2	->	CDKN2A
MCM2	->	MCM5
MCM2	->	MCM7

MCM2	->	MSH5
MCM2	->	MSH5-SAPCD1
MCM2	->	NCAPD3
MCM2	->	PAM
MCM2	->	SAPCD1
MCM3	->	GMNN
MCM4	->	POLE2
MCM4	->	RFC2
MCM6	->	CTNNAL1
MCM6	->	DNA2
MCM6	->	MAD2L1
MCM6	->	PCNA
MCM6	->	RAD51C
MELK	->	POLQ
MKI67	->	BIRC5
MKI67	->	BRCA1
MKI67	->	CDC45
MKI67	->	CDCA3
MKI67	->	CDCA5
MKI67	->	CDK1
MKI67	->	CENPM
MKI67	->	FOXM1
MKI67	->	KIF18B
MKI67	->	KIF4A
MKI67	->	MCM4
MKI67	->	TPX2
MKI67	->	WEE1
NCAPG	->	PSMC3IP
NCAPG	->	RRM1
NCAPG	->	TRIP13
NCAPG	->	ZWINT
NCAPG2	->	DHFR
NIFK	->	MARS2
NIFK	->	PAK1IP1
NIFK	->	PHF5A
NIFK	->	RAN
NIFK	->	SNX5
NIFK	->	WDR75
NIFK	->	ZC3H8
NOP16	->	CIRH1A
NOP16	->	EXOSC4
NOP16	->	NIFK
NOP16	->	PFKM
NOP16	->	TIMM8B
NUP205	->	DNMT1
NUP205	->	NOM1
NUP205	->	PRKCI

NUP205	->	TRRAP
NUSAP1	->	DHFR
NUSAP1	->	FANCI
NUSAP1	->	HMMR
NUSAP1	->	KIAA0101
OIP5	->	CENPH
OIP5	->	CENPJ
OIP5	->	DUT
OIP5	->	HMGB3
OIP5	->	SPDL1
OIP5	->	STIL
OIP5	->	ZWILCH
OSTC	->	H2AFZ
PCNA	->	MSH2
POLE2	->	DNMT1
POLE2	->	KIF2C
POLQ	->	EVI2B
PRC1	->	NUSAP1
PRIM1	->	POLA1
RAD51AP1	->	CEP78
RAD51AP1	->	DDIAS
RAD51AP1	->	POLA1
RAD51AP1	->	RACGAP1
RAD51AP1	->	SMC2
RAD51AP1	->	TMPO
RFC2	->	MCM6
RMI1	->	HMMR
RMI2	->	NCAPG2
RRM2	->	MELK
RRM2	->	NUSAP1
RRM2	->	TYMS
RUVBL1	->	EXOSC4
RUVBL1	->	SORD
SAPCD1	->	MSH5
SAPCD1	->	MSH5-SAPCD1
SMC2	->	NUP205
SMC2	->	RMI1
SMC2	->	TOPBP1
SORD	->	HK2
SORD	->	MYC
SORD	->	NOP16
SORD	->	SELK
SORD	->	TTLL12
SPAG5	->	KPNA2
SPAG5	->	POLQ
STMN1	->	SPAG5
TIMM8B	->	C11orf48

TIMM8B	->	CRELD2
TIMM8B	->	LOC102288414
TIMM8B	->	MRPL17
TIMM8B	->	PTS
TIMM8B	->	SSSCA1
TK1	->	AURKB
TK1	->	RRM2
TMPO	->	LMNB1
TOPBP1	->	FANCI
TPX2	->	CENPA
TPX2	->	EXO1
TPX2	->	HJURP
TPX2	->	SPAG5
TROAP	->	AURKB
TROAP	->	SAPCD2
TYMS	->	CENPW
TYMS	->	STMN1
UBE2T	->	DONSON
UBE2T	->	GMNN
UHRF1	->	ASF1B
WDR4	->	ADSL
ZWILCH	->	NDC80
ZWILCH	->	TNFRSF13C
ZWINT	->	ASF1B
ZWINT	->	BUB1B
ZWINT	->	DTL
ZWINT	->	NCAPG2
ZWINT	->	PBK
ZWINT	->	PHF19
ZWINT	->	SHCBP1
ZWINT	->	UHRF1
ZWINT	->	ZNF367

Supplementary Table 10. Pathway analysis of the prognostic subnetwork

PANTHER GO-Slim Biological Process	#genes in pathway	raw P-value	FDR
cell cycle (GO:0007049)	42	6.12E-26	1.10E-22
mitotic cell cycle process (GO:1903047)	25	1.30E-20	7.81E-18
mitotic cell cycle (GO:0000278)	25	1.30E-20	1.17E-17
mitotic nuclear division (GO:0140014)	23	1.84E-18	8.25E-16
DNA replication (GO:0006260)	20	3.95E-18	1.42E-15
cellular macromolecule biosynthetic process (GO:0034645)	20	2.95E-17	8.83E-15
nucleic acid metabolic process (GO:0090304)	25	5.95E-15	1.34E-12
DNA-dependent DNA replication (GO:0006261)	13	5.62E-15	1.44E-12
DNA metabolic process (GO:0006259)	16	2.13E-14	4.26E-12
cellular biosynthetic process (GO:0044249)	20	3.46E-14	6.22E-12
biosynthetic process (GO:0009058)	20	4.12E-13	6.72E-11
DNA repair (GO:0006281)	15	6.31E-11	9.44E-09
cellular response to DNA damage stimulus (GO:0006974)	16	1.27E-10	1.75E-08
chromosome organization (GO:0051276)	15	2.91E-10	3.74E-08
mitotic sister chromatid segregation (GO:0000070)	8	5.05E-09	6.05E-07
meiotic cell cycle (GO:0051321)	9	2.18E-08	2.31E-06
meiotic cell cycle process (GO:1903046)	9	2.18E-08	2.45E-06
cellular response to stress (GO:0033554)	16	3.73E-08	3.73E-06
nuclear DNA replication (GO:0033260)	5	4.32E-08	3.88E-06
reproductive process (GO:0022414)	9	4.12E-08	3.89E-06
regulation of cell cycle process (GO:0010564)	8	1.36E-07	1.11E-05
DNA biosynthetic process (GO:0071897)	8	1.36E-07	1.16E-05
regulation of cell cycle (GO:0051726)	10	1.65E-07	1.28E-05
cellular process (GO:0009987)	83	2.15E-07	1.61E-05
Unclassified (UNCLASSIFIED)	54	3.68E-07	2.65E-05

metabolic process (GO:0008152)	62	6.28E-07	4.34E-05
DNA strand elongation involved in DNA replication (GO:0006271)	4	1.39E-06	9.25E-05
meiosis I cell cycle process (GO:0061982)	6	1.54E-06	9.88E-05

Supplementary Table 11. Top biological processes enriched in the treatment response signatures

Signature	Biological process	raw p value	FDR
Bhutani_176	defense response to virus (GO:0051607)	1.55E-05	9.27E-03
Bhutani_176	cellular process (GO:0009987)	1.25E-05	2.25E-02
Mitra_42	cellular lipid catabolic process (GO:0044242)	1.88E-03	1.00E+00
Mitra_42	carbohydrate metabolic process (GO:0005975)	5.83E-03	1.00E+00
Mulligan_100	antibacterial humoral response (GO:0019731)	2.95E-04	5.30E-01
Mulligan_100	defense response to Gram-negative bacterium (GO:0050829)	2.09E-03	1.00E+00
Shaughnessy_80	proteasomal protein catabolic process (GO:0010498)	5.83E-10	5.24E-07
Shaughnessy_80	defense response to Gram-negative bacterium (GO:0050829)	2.09E-03	1.00E+00
Zhu_244	protein import into nucleus (GO:0006606)	2.80E-10	5.02E-07
Zhu_244	macromolecule metabolic process (GO:0043170)	1.95E-09	1.75E-06

Supplementary Table 12. Enrichment of response signature genes in chromosomes

Signature	chr.	p-value	num. of genes	Genes
Bhutani_176	chr1	0.02546535	23	MUC1_KCNA3_FCGR2C_JUN_MACF1_FCGR2A_FCGR2B_CRYZ_RABGAP1L_AMPD1_CR2_PBXIP1_FAIM3_RGS1_DPYD_RAB13_PEA15_RNPEP_CAPN2_B4GALT3_DDR2_TRAF3IP3_PRDX6
Mitra_42	chr22	0.01612247	4	MYH9_CERK_NCAPH2_LMF2
Mulligan_100	chrx	2.94E-05	10	GAGE4_MAGEA3_SSX2_CTAG1A_SSX1_SSX4_SLC6A8_SSX3_CTAG1B_MAGEA6
Shaughnessy_80	chr17	0.20838642	5	NMT1_BIRC5_PSMC5_PSMB3_TMC8
Zhu_244	chr4	0.00690187	2	POLR2B_AFF1

Supplementary Table 13. Top biological processes enriched in genes in the response subnetworks

Subnetworks	biology process	raw p value	FDR
subnetwork_Bhutani_176	defense response to virus (GO:0051607)	1.77E-15	3.17E-12
subnetwork_Bhutani_176	immune system process (GO:0002376)	1.47E-10	1.32E-07
subnetwork_Zhu_244	metabolic process (GO:0008152)	1.65E-06	2.97E-03
subnetwork_Zhu_244	organic substance metabolic process (GO:0071704)	4.75E-05	4.27E-02
subnetwork_Shaughnessy_80	cellular response to stress (GO:0033554)	4.92E-05	4.41E-02
subnetwork_Shaughnessy_80	autophagy of mitochondrion (GO:0000422)	3.94E-05	7.08E-02
subnetwork_Mulligan_100	antibacterial humoral response (GO:0019731)	4.11E-07	7.39E-04
subnetwork_Mulligan_100	defense response to Gram-negative bacterium (GO:0050829)	7.01E-06	6.30E-03

Supplementary Table 14. Nodes in the IMiD response subnetwork.

Node name (gene symbol)
APEX1
PRMT5
LSM12
NDUFB5
FOSB
JUN
NASP
PIGU
PHF14
PLRG1
SYNCRIP
C10orf10
CDC73
KIAA1279
PRKAR1A
CUL2
SMC4
HPRT1
NNT
SNRPD1
WDR4
HSPA14
RNF138
NEDD1
YTHDF3
GAS6
C14orf166
RP11-97C16.1
SOCS3
MDN1
LARP4
PSMA7
PPP1R15A
ASCC3
ADSL
CPSF3
NDUFAF4
MRTO4
MAT2A
NUP133
POLR1C
POLR2G
ESF1
GTF3C3
TFAM

ZFR
NOLC1
AGPS
CSTF1
NPEPPS
REEP2
MTPAP
EXOSC3
NUDT21
SQSTM1
ORC4
POT1
UMPS
DDX21
SF3A3
MIS18A
PRB1
LOC100131541
USP34
MIR6733
CCDC186
LDHA
AJ420595
FOS
PSMD1
SMC3
SCAF11
DIEXF
GEMIN2
GEMIN5
MKKS
CAND1
MLN
NUP153
CAPZA2
TAF1D
PRR7
DNAJA2
DNAJA3
ALDH18A1
OTUD1
NAA25
NIF3L1
TIMM23B
ZNF638
PUS7
ZNF638-IT1

LTV1
MBNL1
UTP20
XPO1
PCNP
BCLAF1
DNAJC25-GNG10
ZBTB41
ANKRD44
F12
BIRC6
PTP4A3
RAD21
TIA1
TROVE2
RANBP9
PA2G4
CCT2
SETD2
GNL2
PSMD14
PSMA1
PSMA6
EIF2B2
ALYREF
GLUD1
FLJ38717
NME3
FTSJ2
ALKBH3
G2E3
POU2F2
IL13RA1
AK022030
IDH1
RAB8A
WNT4
SRSF10
TXNDC16
SRBD1
MAGOH
MDM1
GNAI3
LOC100130872
PTPRG
MST4
WRN

TXLNG
C4orf46
NXT1
ZNF639
CMTM7
CNIH1
MUC1
COL9A2
RXRA
CCDC91
CCNB1IP1
SLC33A1
CASP3
DCP1B
DCBLD1
C6orf203
MTIF2
OXCT1
ENO1
CECR5
SNRNP40
C10orf2
PSMD13
PEX1
TSPAN33
C1GALT1
DHRS3
SNX14
MTMR12
KBTBD2
METAP2
HSF2
ILF3
ARMC8
TWISTNB
SRRD
TTPAL
FAM118B
C12orf73
COMMD9
KPNA3
XRN2
KNSTRN
TOMM40L
GEMIN6
ALAS1
ETF1

RPA2
APMAP
IGSF22
BAZ1A
ZMYND8
EBNA1BP2
ANKRD12
SON
DUSP1
METTL21A
CBX3
ARID2
HMGCS1
TUBB
NANP
TTC4
KEAP1
TBC1D15
TXNL1
KIAA1033
P2RX7
RP11-500C11.3
KLF11
RALA
CSNK2A2
NARS
PUS1
OAT
YTHDC2
ZDHHC17
USP14
SMCHD1
SRGAP2
LOC101060691
RBM41
DCAF16
KBTBD3
CCNC
PPP1R12A
WEE1
ALMS1
DNAJC25
GRK6
MIB1
LLPH
RAB3GAP2
SFT2D2

CORO1C
CA5BP1
RPF2
PPIH
PSMC1
DTD2

Supplementary Table 15. Regulations among nodes in the IMiD response subnetwork.

**Parent node**	**child node**
APEX1	PRMT5
PRMT5	EIF2B2
LSM12	ALYREF
LSM12	GLUD1
NDUFB5	C14orf166
FOSB	JUN
JUN	FLJ38717
NASP	C10orf10
PIGU	NME3
PHF14	FTSJ2
PHF14	ALKBH3
PLRG1	YTHDF3
PLRG1	G2E3
PLRG1	LARP4
PLRG1	BIRC6
SYNCRIP	ASCC3
C10orf10	POU2F2
C10orf10	IL13RA1
CDC73	PRKAR1A
CDC73	AK022030
CDC73	KIAA1279
KIAA1279	IDH1
PRKAR1A	RAB8A
CUL2	WNT4
CUL2	SRSF10
CUL2	TXNDC16
CUL2	NNT
CUL2	SRBD1
CUL2	MAGOH
CUL2	MDM1
CUL2	GNAI3
CUL2	LOC100130872
CUL2	CAPZA2
SMC4	PTPRG
HPRT1	MST4
NNT	ZFR
SNRPD1	WDR4
SNRPD1	APEX1
SNRPD1	DDX21
WDR4	ADSL
HSPA14	WRN
HSPA14	RNF138
HSPA14	TXLNG
HSPA14	C4orf46
HSPA14	NXT1

RNF138	ZNF639
NEDD1	CAND1
YTHDF3	MST4
GAS6	CMTM7
C14orf166	CNIH1
C14orf166	APEX1
RP11-97C16.1	BIRC6
SOCS3	PTP4A3
SOCS3	C10orf10
SOCS3	MUC1
MDN1	COL9A2
MDN1	RXRA
MDN1	CCDC91
MDN1	CCNB1IP1
LARP4	SLC33A1
LARP4	CASP3
LARP4	DCP1B
PSMA7	PSMD1
PPP1R15A	SQSTM1
ASCC3	DCBLD1
ASCC3	NDUFAF4
ASCC3	C6orf203
ADSL	MTIF2
ADSL	OXCT1
ADSL	ENO1
ADSL	MRTO4
ADSL	CECR5
ADSL	CPSF3
CPSF3	GNL2
NDUFAF4	C6orf203
MRTO4	PA2G4
MAT2A	SNRNP40
NUP133	CPSF3
NUP133	PSMD1
POLR1C	C10orf2
POLR2G	PSMD13
ESF1	GTF3C3
GTF3C3	UTP20
GTF3C3	XPO1
GTF3C3	PEX1
GTF3C3	TSPAN33
GTF3C3	G2E3
GTF3C3	LTV1
GTF3C3	C1GALT1
GTF3C3	DHRS3
TFAM	GLUD1
TFAM	HSPA14

TFAM	CUL2
ZFR	SNX14
ZFR	MTMR12
ZFR	RANBP9
ZFR	KBTBD2
ZFR	METAP2
ZFR	HSF2
ZFR	ILF3
ZFR	ARMC8
NOLC1	DDX21
AGPS	POT1
AGPS	SF3A3
AGPS	NUDT21
AGPS	ORC4
AGPS	CSTF1
AGPS	MTPAP
CSTF1	TWISTNB
CSTF1	SRRD
CSTF1	TTPAL
CSTF1	FAM118B
CSTF1	PIGU
CSTF1	C12orf73
CSTF1	COMMD9
NPEPPS	XPO1
REEP2	CUL2
MTPAP	KPNA3
EXOSC3	GEMIN5
EXOSC3	UMPS
NUDT21	DNAJA2
SQSTM1	PRR7
ORC4	XRN2
ORC4	NIF3L1
POT1	PHF14
UMPS	KNSTRN
UMPS	TOMM40L
UMPS	C6orf203
UMPS	GEMIN6
UMPS	ALAS1
DDX21	ETF1
SF3A3	RPA2
SF3A3	SNRNP40
SF3A3	APMAP
SF3A3	IGSF22
MIS18A	CSTF1
PRB1	MUC1
PRB1	MLN
PRB1	BAZ1A

PRB1	CUL2
LOC100131541	USP34
USP34	SCAF11
USP34	ZMYND8
MIR6733	EBNA1BP2
CCDC186	MBNL1
CCDC186	PCNP
CCDC186	ANKRD12
CCDC186	BCLAF1
LDHA	DDX21
AJ420595	SON
FOS	DUSP1
FOS	FOSB
PSMD1	METTL21A
SMC3	RAD21
SMC3	ESF1
SMC3	NEDD1
SMC3	CBX3
SCAF11	ARID2
DIEXF	GEMIN5
DIEXF	GEMIN2
GEMIN2	HMGCS1
GEMIN5	TUBB
GEMIN5	NANP
GEMIN5	TTC4
GEMIN5	KEAP1
GEMIN5	MAT2A
MKKS	XRN2
CAND1	TBC1D15
CAND1	TXNL1
CAND1	KIAA1033
MLN	NUP153
NUP153	P2RX7
NUP153	RP11-500C11.3
NUP153	KLF11
CAPZA2	RALA
TAF1D	BAZ1A
PRR7	F12
DNAJA2	CSNK2A2
DNAJA3	NARS
DNAJA3	PUS1
ALDH18A1	OAT
OTUD1	JUN
NAA25	MDN1
NIF3L1	DNAJA3
TIMM23B	ALDH18A1
TIMM23B	NOLC1

ZNF638	YTHDC2
ZNF638	ZDHHC17
ZNF638	USP14
ZNF638	USP34
ZNF638	SMCHD1
ZNF638	SRGAP2
ZNF638	LOC101060691
ZNF638	RBM41
ZNF638	DCAF16
ZNF638	KBTBD3
PUS7	NOLC1
PUS7	POLR1C
ZNF638-IT1	ZNF638
ZNF638-IT1	ZFR
LTV1	CCNC
MBNL1	SMC4
UTP20	NAA25
XPO1	PPP1R12A
XPO1	WEE1
XPO1	ALMS1
PCNP	SETD2
BCLAF1	DNAJC25
BCLAF1	DNAJC25-GNG10
BCLAF1	AJ420595
DNAJC25-GNG10	DNAJC25
ZBTB41	IDH1
ZBTB41	AK022030
ANKRD44	F12
ANKRD44	PTP4A3
F12	GRK6
BIRC6	MIB1
BIRC6	TIA1
BIRC6	LLPH
PTP4A3	GAS6
RAD21	TROVE2
TIA1	MDM1
TROVE2	RAB3GAP2
TROVE2	SFT2D2
TROVE2	AK022030
TROVE2	CORO1C
RANBP9	CA5BP1
PA2G4	LSM12
CCT2	PSMA6
CCT2	GNL2
CCT2	PSMA1
CCT2	ADSL
CCT2	C12orf73

SETD2	ZNF639
GNL2	RPF2
GNL2	MIR6733
GNL2	PPIH
GNL2	EBNA1BP2
PSMD14	PSMD1
PSMA1	PSMD13
PSMA6	PRMT5
PSMA6	PSMC1
PSMA6	DTD2
PSMA6	FOSB

Supplementary Table 16. Pathway analysis of subnetwork of IMiD treatment

PANTHER GO-Slim Biological Process	raw P-value	FDR
defense response to virus (GO:0051607)	2.47E-09	4.43E-06
regulation of multi-organism process (GO:0043900)	4.35E-06	2.60E-03
proteolysis involved in cellular protein catabolic process (GO:0051603)	1.26E-04	4.54E-02
cellular protein catabolic process (GO:0044257)	1.34E-04	4.02E-02
metabolic process (GO:0008152)	2.76E-06	2.48E-03
organic substance metabolic process (GO:0071704)	5.16E-05	2.32E-02

Supplementary Figures

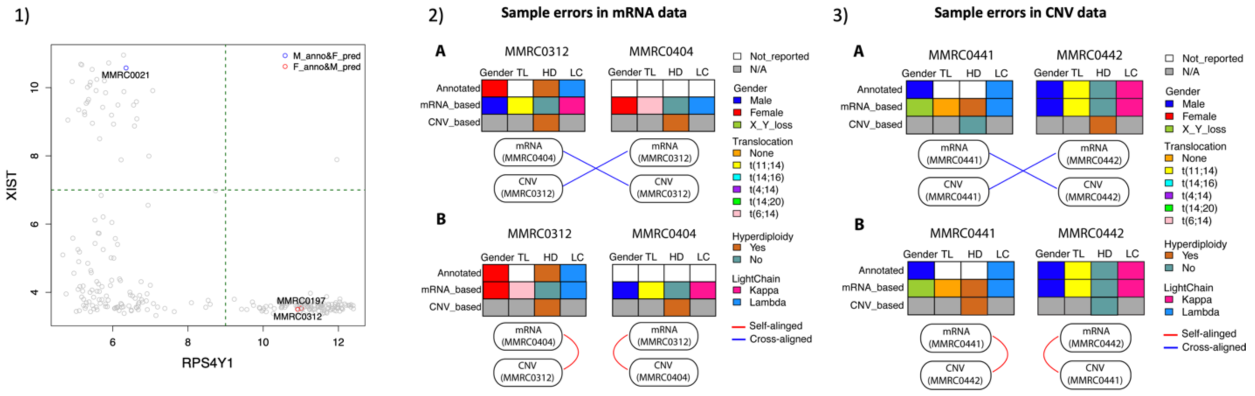

Supplementary Figure 1. Potential sample label errors in the dataset. 1)Scatter plot of expression of two sex related genes: *XIST* and *RPS4Y*; 2-3) Sample swaps identified by *pro*MODMatcher.

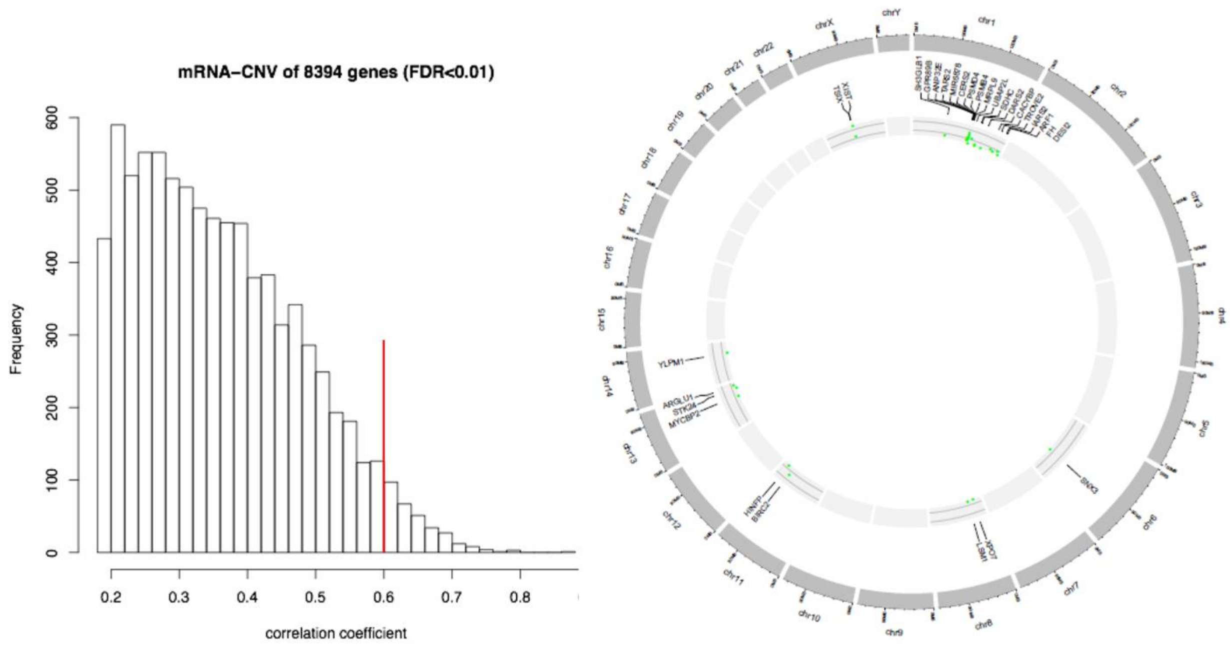

Supplementary Figure 2. Association between CNV and mRNA profiles. 8,394 genes had significant associations between their CNV and mRNA profiles at FDR < 0.01, and genes with highly correlated CNV and mRNA profiles (correlation coefficient > 0.6) were enriched in chr1q, the red line indicates the cutoff of correlation coefficient = 0.6.

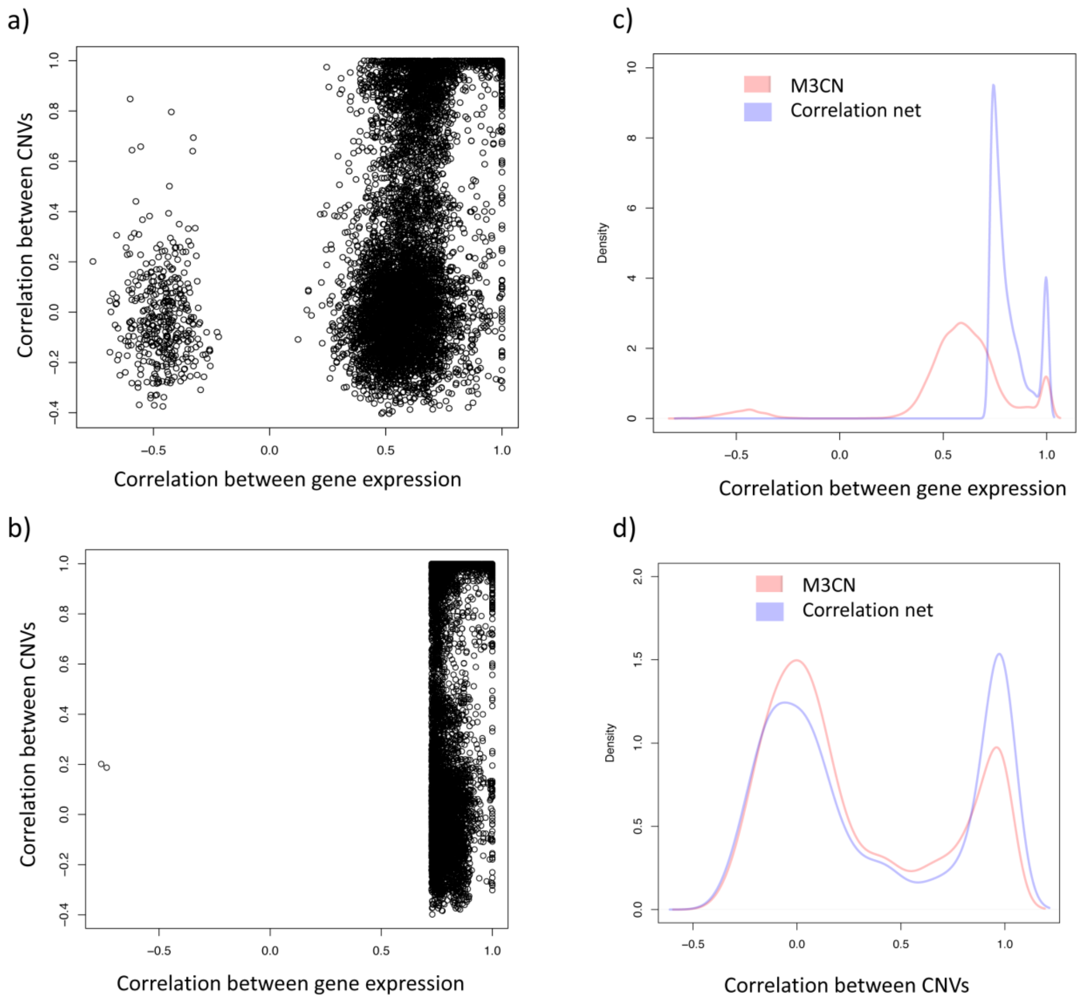

Supplementary Figure 3. Distribution of correlations between gene expression, and correlations between CNVs. A) A scatter plot of correlations between gene expression (x-axis) and CNV (y-axis) profiles for gene pairs connected in M3CN; b) a scatter plot of correlations between gene expression (x-axis) and CNV (y-axis) profiles for the most correlated gene pairs based on gene expression profiles (correlation-based network); c) histograms of gene expression correlations; d) histograms of gene CNV correlations.

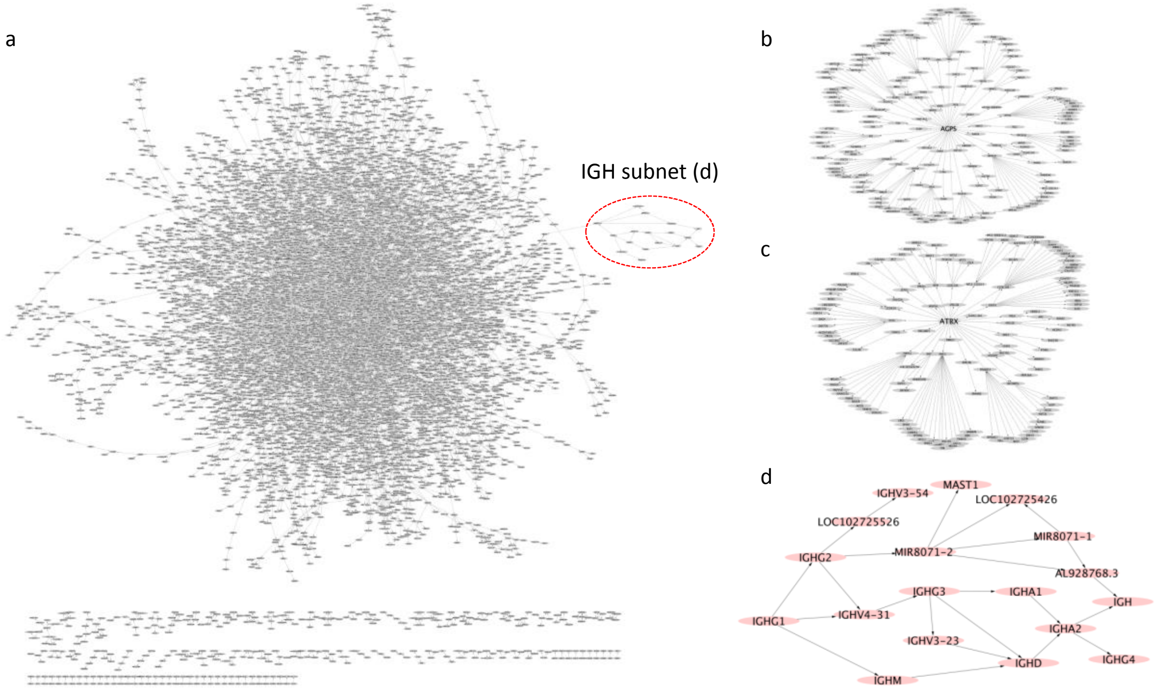

Supplementary Figure 4. The M3CN, hub genes, and IGH subnet. a) The global view of M3CN ; b) and c) the two most connected genes, *AGPS* (Alkylglycerone Phosphate Synthase) and *ATRX* (Alpha Thalassemia/Mental Retardation Syndrome X-Linked), regulated dozens of genes directly (41 and 32 respectively); d) the IGH subnet was disconnected from the rest of M3CN.

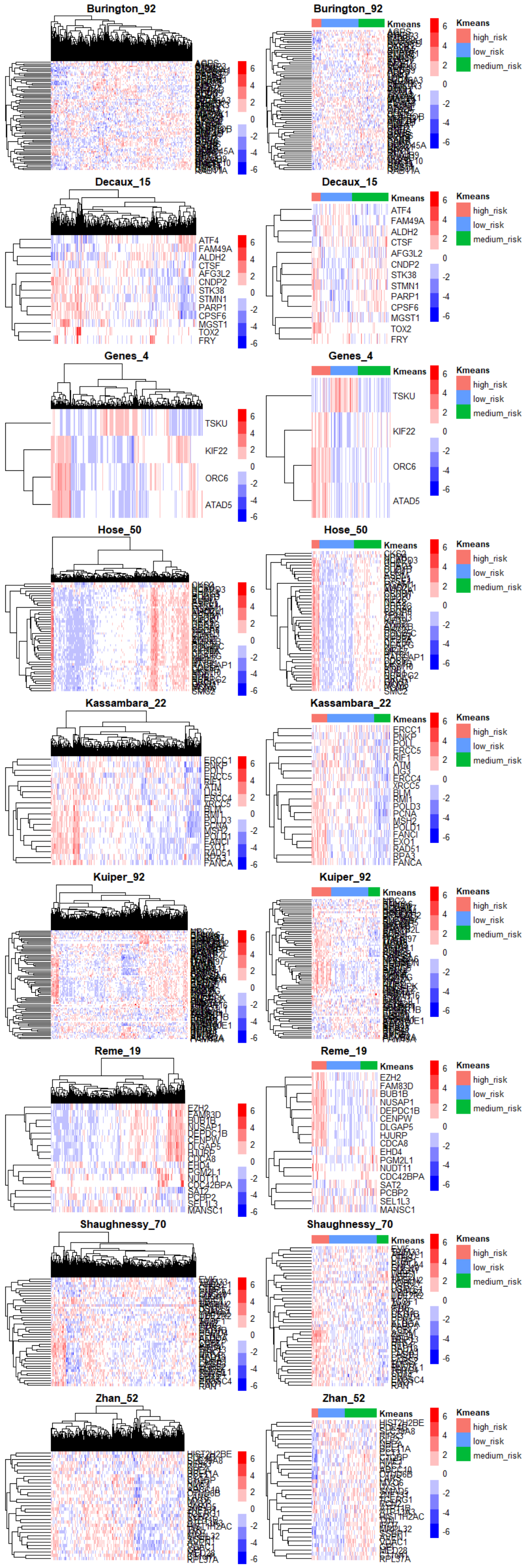
Burington_92
Kmeans
high_risk
low_risk
medium_risk
6
4
2
0
-2
-4
-6
Decaux_15
ATF4
FAM49A
ALDH2
CTSF
AFG3L2
CNDP2
STK38
STMN1
PARP1
CPSF6
MGST1
TOX2
FRY
Genes_4
TSKU
KIF22
ORC6
ATAD5
Hose_50
Kassambara_22
ERCC1
FNKP
POLL
ERCC5
RIF1
ATM
LIG3
ERCC4
XRCC5
BLM
RMI1
POLD3
PCNA
MSH2
POLD1
FANCI
EXO1
RAD51
RPA3
FANCA
Kuiper_92
Reme_19
EZH2
FAM83D
BUB1B
NUSAP1
DEPDC1B
CENPW
DLGAP5
HJURP
CDCA8
EHD4
PGM2L1
NUDT11
CDC42BPA
SAT2
PCBP2
SEL1L3
MANSC1
Shaughnessy_70
Zhan_52
HIST2H2BE
RPL37A

Supplementary Figure 5. Results of hierarchical clustering (left panels) and k-mean clustering (k=3, right panels) RNAseq data of the MMRF-CoMMpass cohort based on genes in the different prognostic signatures.

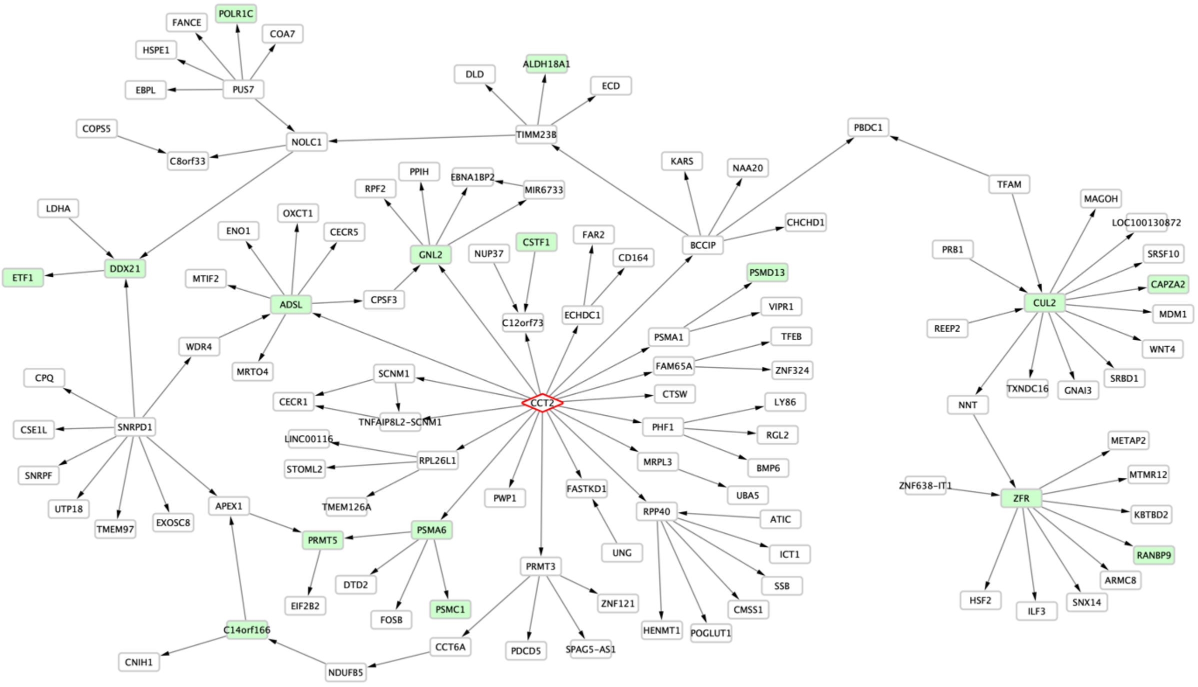

Supplementary Figure 6 The subnetwork generated based on Zhu_244 (subnetwork_Zhu_244), the diamond shape with red border indicates key regulator gene. Nodes in green are genes in Zhu_244.

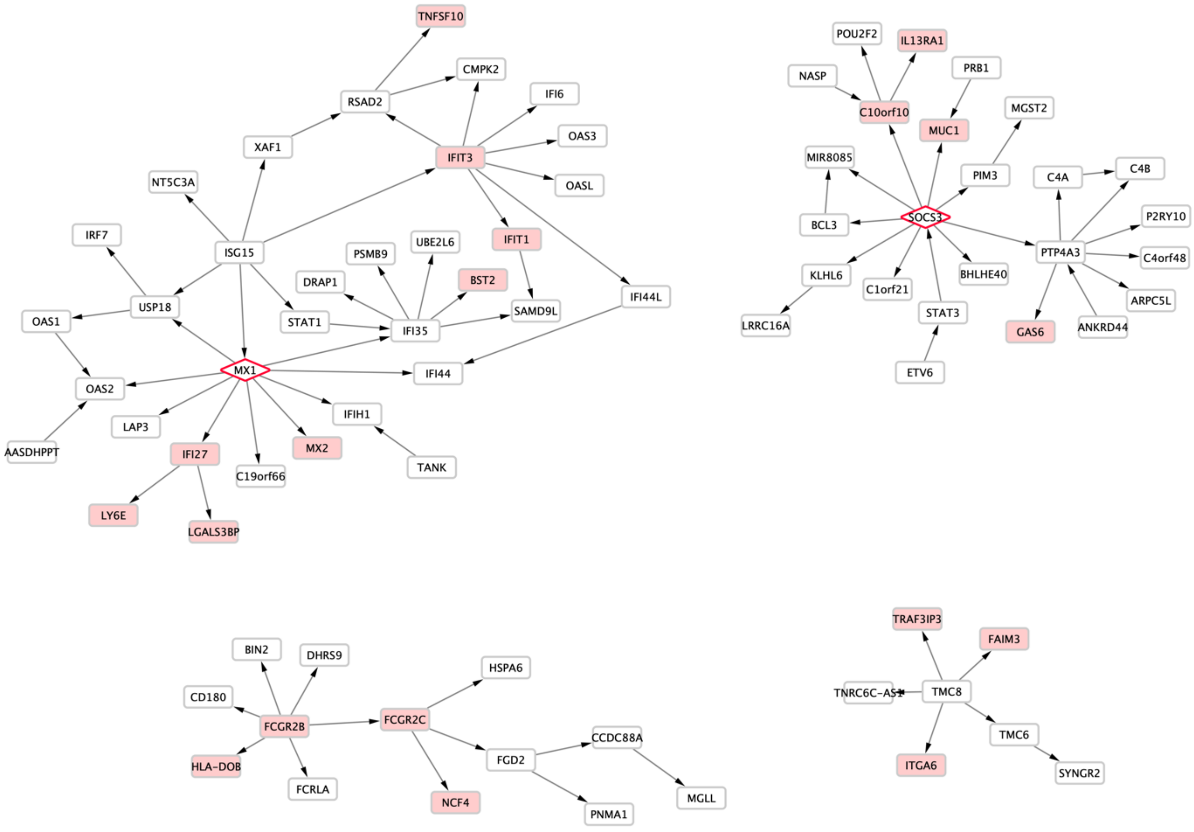

Supplementary Figure 7 The subnetwork generated based on Bhutani_176 (subnetwork_Bhutani_176). The diamond shape with red border indicates key regulator gene. Nodes in red are genes in Bhutani_176.

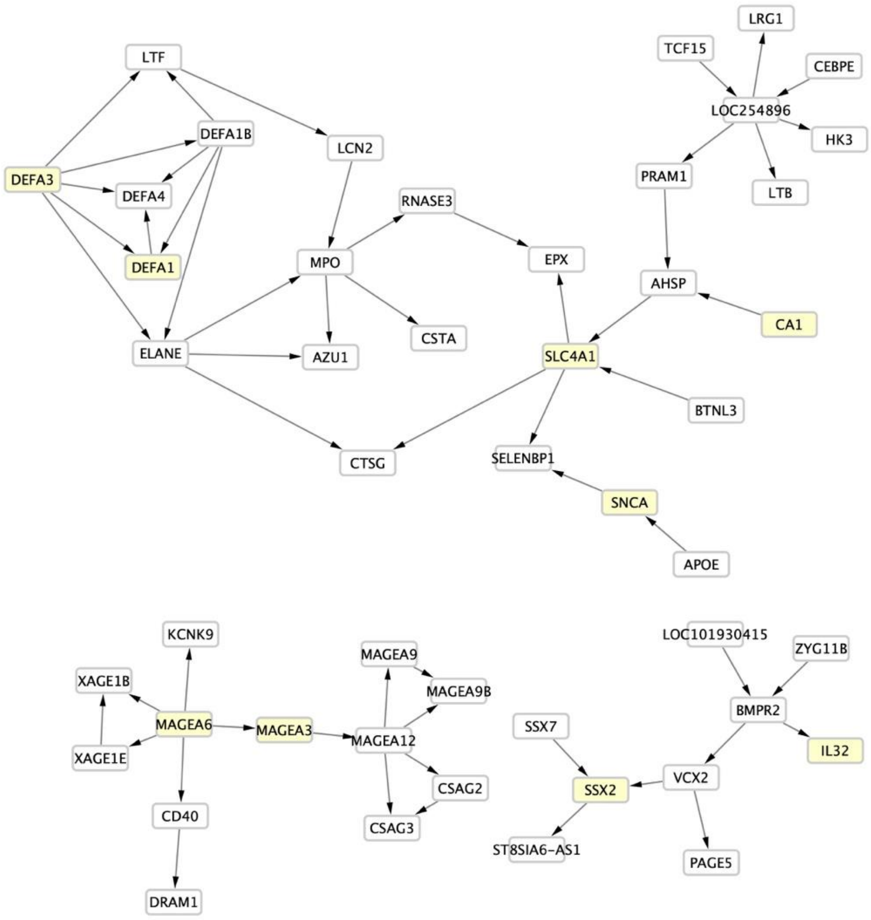

Supplementary Figure 8 subnetworks generated based on Mulligan_100 (subnetwork_Mulli-gan_100), nodes in yellow are genes in Mulligan_100

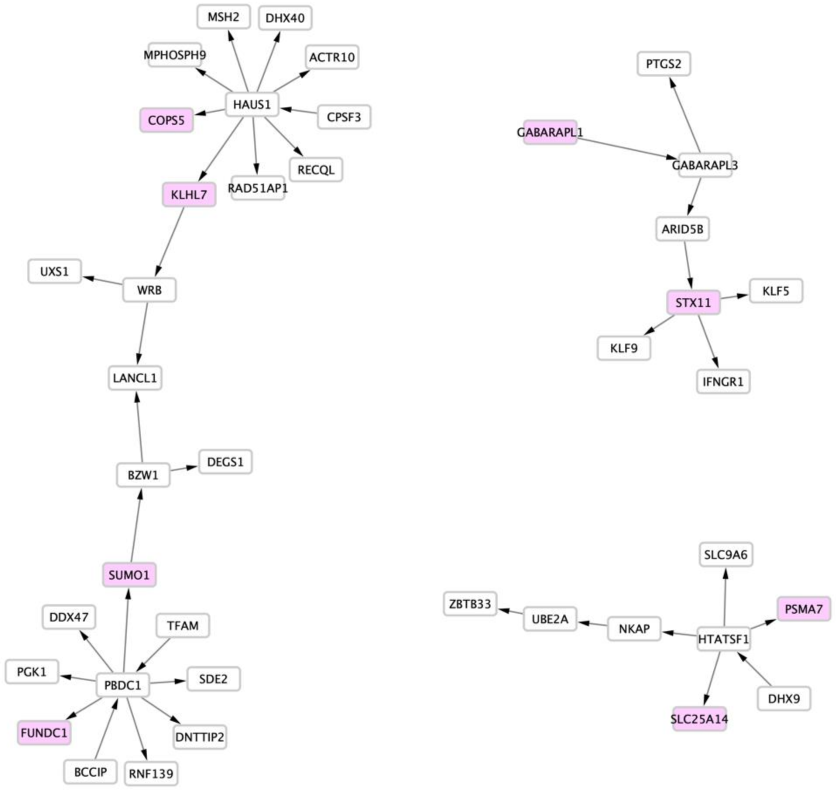

Supplementary Figure 9 The subnetworks generated based on Shaughnessy_80 (subnetwork_ Shau-ghnessy_80), nodes in pink are genes in Shaughnessy_80.

© 2019 by the authors. Submitted for possible open access publication under the terms and conditions of the Creative Commons Attribution (CC BY) license (http://creativecommons.org/licenses/by/4.0/).
